# Spatial predictive properties of built environment characteristics assessed by drop-and-spin virtual neighborhood auditing

**DOI:** 10.1186/s12942-020-00213-5

**Published:** 2020-05-29

**Authors:** Jesse J. Plascak, Mario Schootman, Andrew G. Rundle, Cathleen Xing, Adana A. M. Llanos, Antoinette M. Stroup, Stephen J. Mooney

**Affiliations:** 1 Department of Biostatistics and Epidemiology, Rutgers School of Public Health, Piscataway, NJ USA; 2 Rutgers Cancer Institute of New Jersey, New Brunswick, NJ USA; 3 Department of Clinical Analytics, SSM Health, St. Louis, MO USA; 4 Department of Epidemiology, Mailman School of Public Health, Columbia University, New York, USA; 5 New Jersey Department of Health, New Jersey State Cancer Registry, Trenton, NJ USA; 6 Department of Epidemiology, University of Washington, Seattle, WA USA

**Keywords:** Virtual neighborhood audit, Built environment, Kriging, Spatial autocorrelation, Spatial prediction

## Abstract

**Background:**

Virtual neighborhood audits have been used to visually assess characteristics of the built environment for health research. Few studies have investigated spatial predictive properties of audit item responses patterns, which are important for sampling efficiency and audit item selection. We investigated the spatial properties, with a focus on predictive accuracy, of 31 individual audit items related to built environment in a major Metropolitan region of the Northeast United States.

**Methods:**

Approximately 8000 Google Street View (GSV) scenes were assessed using the CANVAS virtual audit tool. Eleven trained raters audited the 360^°^ view of each GSV scene for 10 sidewalk-, 10 intersection-, and 11 neighborhood physical disorder-related characteristics. Nested semivariograms and regression Kriging were used to investigate the presence and influence of both large- and small-spatial scale relationships as well as the role of rater variability on audit item spatial properties (measurement error, spatial autocorrelation, prediction accuracy). Receiver Operator Curve (ROC) Area Under the Curve (AUC) based on cross-validated spatial models summarized overall predictive accuracy. Correlations between predicted audit item responses and select demographic, economic, and housing characteristics were investigated.

**Results:**

Prediction accuracy was better within spatial models of all items accounting for both small-scale and large- spatial scale variation (vs large-scale only), and further improved with additional adjustment for rater in a majority of modeled items. Spatial predictive accuracy was considered ‘Excellent’ (0.8 ≤ ROC AUC < 0.9) for full models of all but four items. Predictive accuracy was highest and improved the most with rater adjustment for neighborhood physical disorder-related items. The largest gains in predictive accuracy comparing large- + small-scale to large-scale only models were among intersection- and sidewalk-items. Predicted responses to neighborhood physical disorder-related items correlated strongly with one another and were also strongly correlated with racial-ethnic composition, socioeconomic indicators, and residential mobility.

**Conclusions:**

Audits of sidewalk and intersection characteristics exhibit pronounced variability, requiring more spatially dense samples than neighborhood physical disorder audits do for equivalent accuracy. Incorporating rater effects into spatial models improves predictive accuracy especially among neighborhood physical disorder-related items.

## Background

Characteristics of the built environment measured through visual observation have been associated with various health-related factors and outcomes, including physical activity [1, 2], obesity [3], injuries [4], violence [5], diabetes [6], and depression [7, 8]. For example, one experimental study of vacant lot “cleaning and greening”—removal of garbage and debris, repairing structures or improving yard conditions—reported lower resident perception of crime and fear for safety along with lower police-reported crime comparing experimental to control regions [5]. Other studies have found more walkable environments—presence of sidewalks, condition of sidewalks, density of destinations—associated with greater physical activity and less diabetes [1, 2, 6]. Besides physical activity, hypothesized pathways through which built environment factors might influence health include substance use and psychosocial stress [9]. Identifying specific built environment characteristics that might be associated with health outcomes is important for motivating place-based interventions of the built environment [5, 10, 11]. However, associations of many of these studies are small, underscoring the importance of well-designed measures of the built environment that maximize accuracy [12].

Neighborhood auditing (i.e., “systematic social observation”, “systematic field observation”) is a systematic method used to assess specific built environment characteristics that might influence health behaviors and outcomes [13–15]. Such audits were initially conducted by in-person observations along street segments [16], but recent and readily available residential imagery from mapping- and advertisement-based businesses such as Google (i.e., Google Street View) have led to development of virtual neighborhood audit tools and protocols [14, 17–20]. Studies comparing in-person to virtual neighborhood audits have concluded that virtual audits are reliable, valid, time-, and resource-efficient methods for assessing visual neighborhood characteristics, especially when those characteristics are conspicuous and more stable over time (e.g., sidewalk present, pedestrian signal, building conditions, etc.) [17, 19, 21–25].

Although recognizing the potential for large-scale built environment characterization, most virtual neighborhood audit studies have assessed pre-defined and small areas surrounding the residence of participants of an extant health study [21]. Few studies randomly sampled potential audit locations with the intent of generating geographically-generalizable estimates of built environment characteristics across large areas (larger than typical U.S. counties or large cities) [26]. In line with the original, in-person practice of neighborhood audits, most virtual audit tools and protocols that have been developed to date utilize street segments surrounding the residence of study participants as the sampling unit. Segment-based audits have prevailed over time despite previous studies reporting that such commonly audited built constructs as walkability and neighborhood physical disorder—indicated by presence of garbage, graffiti, poor building and yard conditions, etc.—positively spatially autocorrelate at distances up to 1000 meters [27–30]. This spatial autocorrelation, or notion that “…pairs of observations taken nearby are more alike than those taken farther apart” [31] suggests that segment-based audits might not be the most efficient sampling unit because information across a typical segment is partially redundant. Despite the few reports of spatially autocorrelated constructs, the spatial trend, autocorrelation, and predictive performance (hereafter, ‘spatial properties’) of models of individual audit items has yet to be systematically and thoroughly investigated.

A recent adaptation to virtual audits has been developed called “drop-and-spin” where observations are limited to a single 360^°^ view around a virtual scene, as opposed to traversing the entire segment [32]. This method was explicitly developed to test whether a surface of estimated built environment measures across a study region could be generated based on the spatial properties of resulting audit responses. Previously reported test–retest and inter-rater reliability of drop-and-spin auditing is similar to that of segment-based audits of identical items [32]. Moreover, the median item-location rating time of “drop-and-spin” auditing (7.3 s) is similar to that of the fastest reported segment-based method (7.9 s) [20], and twice as fast as typical times (15 s) [4, 14, 17–20, 25, 26, 33–38]. However, it is not known whether, or to what degree, drop-and-spin audit responses exhibit spatial properties that are required for accurate spatial prediction.

Estimates of the spatial properties of neighborhood audit item response patterns are important for several reasons. First, investigation of “drop-and-spin” audit item-specific spatial properties is critical for motivating whether point-based auditing might be used in place of segment-based. When audit item responses demonstrate spatial autocorrelation and high spatial predictive accuracy, point-based audits of a sample of points may characterize neighborhood conditions as accurately as a segment-based census of street segments but at lower cost. Lack of any spatial components to audit responses would indicate that drop-and-spin auditing is only useful at the point location assessed and cannot be generalized any further.

Second, investigation of spatial prediction performance of specific audit items, segment- or point-based, has yet to be investigated. Such results are important as use of such spatial prediction methods to yield estimates of built environment characteristics across epidemiologic study regions have been increasingly recommended [39–42], including characteristics assessed from virtual neighborhood audit studies [25].

Third, previous audit studies of rater-reliability indicate large variability in test–retest and inter-rater agreement of audit item responses [23, 27, 32, 43]. If this disagreement is systematic as opposed to random (e.g., one rater consistently rates the same sidewalk quality as worse than another does), then it is possible to improve spatial prediction accuracy by accounting for this source of inaccuracies in measurement [44, 45].

Fourth, observable built environment features such as pedestrian amenities are indicators of historical social processes that have distinct spatial distributions [46, 47]. Individual audit item indicators of health-relevant constructs vary at different spatial scales and understanding these variations is important to informing how best to build constructs from response patterns as well as the processes influencing these patterns. For example, number of traffic lanes and presence of sidewalks are both indicators of pedestrian-friendliness at a given location [48], but number of lanes is very street-dependent, whereas presence of sidewalks is not, and so these should not be combined into a single measure for spatial interpolation.

The purpose of this study was to investigate the spatial properties, with a focus on spatial prediction, of 31 commonly-assessed characteristics of the built environment measured using the newly developed drop-and-spin virtual neighborhood audit method. Relationships between predicted audit item responses as well as between predicted audit responses and various neighborhood characteristics were explored to inform relationships within audit item responses and between social factors and audit item responses.

## Methods

### Study sample

**Fig. 1:**
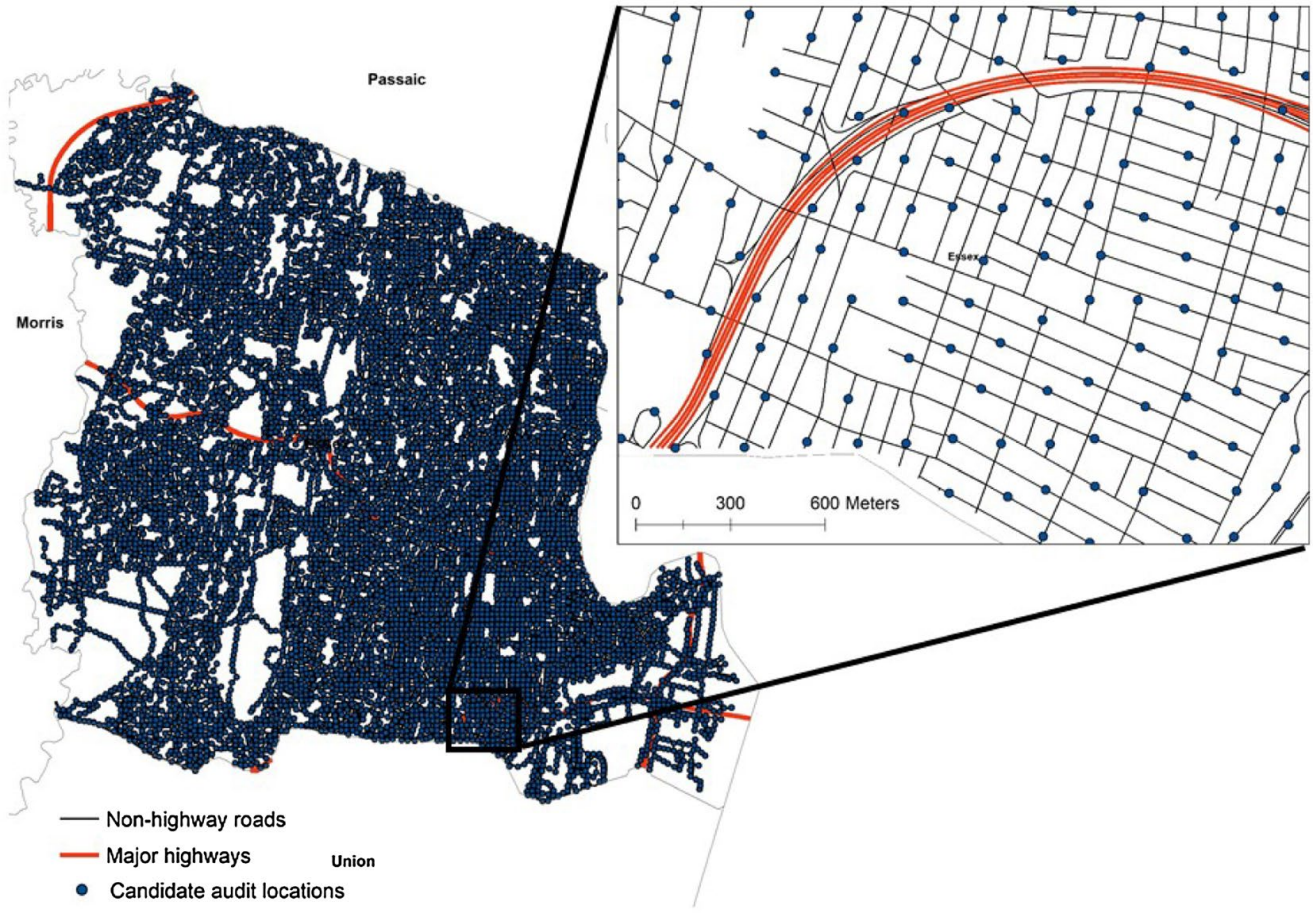
8,262 candidate neighborhood audit locations assessed for 31 different built environment characteristics, Essex County, New Jersey

Virtual neighborhood audit locations were generated across non-highway roads within Essex County, NJ. New Jersey is the most densely populated U.S. state (1195.5 people per square mile), and Essex is the most populous county in NJ (783,969) [49]. Essex County contains Newark, NJ and other densely populated urban areas to the east. Newark International airport is the area with few audit locations that is immediately southeast of the inset (Fig. 1). Numerous suburban communities and non-residential parks (i.e., indicated by no roads/audit locations) lie to the less dense western region. Details of the sampling scheme, audit training protocol, audit item prevalence, and audit item reliability have been previously described [32]. In brief, iterative GIS operations—random point generation, point-to-point near distance calculation, integration of points within a specific distance of separation, event collection, snapping to road file—were completed to generate points along non-highway roads. In order to have enough power to test for spatial autocorrelation, which studies of similar constructs have reported occur within distances of 1000 meters, the above GIS operations were repeated until the average point-to-point near distance was within 1 standard deviation of 150 meters (mean = 142 meters, standard deviation = 18 meters), resulting in 8262 total candidate audit locations (25.3 per square km) (Fig. 1).

Eleven raters were trained during in-person sessions using a standardized protocol and manual [32]. Thirty-one audit items—10 intersection-related, 10 sidewalk-related, and 11 neighborhood physical disorder-related, were assessed at each audit location. Raters were assigned audit locations at random throughout the study region. Groups of audit items were assigned to raters according to item similarity (i.e., intersection-related or sidewalk-related) or belonging to a theoretical construct (i.e., neighborhood physical disorder/aesthetics). Full audit item wording and response values are displayed in Table 1. The virtual neighborhood audit platform called CANVAS was used in this study Although numerous virtual auditing platforms exist (see Rzotkiewicz et al.), CANVAS is arguably the most frequently used platform and efficiently combines GSV scene visualization with audit items in a consistent and clear interface [14, 21]. Audit items were chosen from previous scales, or theoretically related to constructs of previous scales, that assess observable built environment characteristics related to walkability, pedestrian infrastructure, and physical disorder [13, 29, 50, 51].

**Table 1:** Audit item name, full wording, response categorization, and prevalence

Audit item (n/% yes)	Question	Response categorization
Neighborhood physical disorder-related		
Garbage (3285/41.2)	Is there garbage, litter, or broken glass in the street or on the sidewalks (≥ 5 items)?	Yes (1) vs no (2)
Abandoned cars (13/0.2)	Are there abandoned cars?	Yes (1) vs no (2)
Building conditions ≥ moderate (6349/86.3)	How would you rate the condition of most of the buildings?	Very well/Moderately well kept (1) vs fair/poor condition (2)
Yard conditions ≥ moderate (5388/87.7)	How would you rate the condition of most of the yards?	Very well/Moderately well kept (1) vs fair/poor condition (2)
Dumpster (706/8.9)	Are there dumpsters visible?	≥1 (1) vs None (2)
Graffiti (904/11.3)	Is there graffiti, or evidence of graffiti that has been painted over, on buildings, signs, or walls?	Yes (1) vs no (2)
Boarded/burned building (502/6.3)	Do you see boarded up, abandoned, or burned out buildings?	Yes (1) vs no (2)
Outdoor seating (2131/27.1)	Do you see any outdoor seating (e.g., benches, porch swings, restaurant seating)?	Yes (1) vs No (2)
Team sports (187/2.4)	Do you see any team sports equipment (e.g., street hockey nets, soccer nets, basketball hoops)?	Yes (1) vs no (2)
Yard decorations (3932/49.9)	Do you see any exterior or yard decorations (e.g. banners/flags, seasonal decorations, water fountains/statues)?	Yes (1) vs no (2)
Fences (6390/80.3)	Do you see fences separating adjoining yards/properties or separating yards from the street?	Yes (1) vs no (2)
Sidewalk-related		
Sidewalk present (5462/68.2)	What type of sidewalk or path (paved or unpaved) is there?	Sidewalk (1) vs none (2)
Complete sidewalk (4948/90.4)	Is the sidewalk complete/continuous?	Complete (1) vs incomplete (2)
Sidewalk condition (3535/64.7)	In what condition is the sidewalk or pedestrian path?	Good (1) vs fair/poor (2)
Sidewalk width (872/15.9)	How wide is the sidewalk?	≥ 4 feet (1) vs < 4 feet (2)
Sidewalk from curb distance (71/1.3)	How far is the sidewalk or path from the curb?	≥ 5 feet (1) vs < 5 feet (2)
Car obstruction (340/6.2)	Do parked cars obstruct the path?	Yes (1) vs no (2)
Garbage can obstruction (301/5.5)	Do garbage cans obstruct the path?	Yes (1) vs no (2)
Pole or sign obstruction (262/5.8)	Do poles or signs obstruct the path?	Yes (1) vs no (2)
Other obstruction (445/8.1)	Does anything else obstruct the path?	Yes (1) vs no (2)
Curb cuts (2585/52.1)	Do you see any curb cuts?	Yes (1) vs no (2)
Intersection-related		
Clear intersection (4741/60.1)	Can you clearly see an intersection?	Yes (1) vs no (2)
Pedestrian crossing sign (860/17.9)	Is there a pedestrian crossing warning sign?	Yes (1) vs no (2)
Pedestrian signal (718/15.1)	Is there a pedestrian signal?	Yes (1) vs no (2)
Pedestrian crossing marks (2975/64.0)	Consider places intended for pedestrians to cross the street. Are these places marked for pedestrian crossing?	All/some (1) vs none (2)
Type of pedestrian crosswalk marks (2076/68.4)	How is the road marked at crosswalks?	Painted solid lines (1) vs other (2)
Traffic signal type (3438/73.8)	What kind of traffic signal can you see?	Traffic signal, stop sign, yield sign (1) vs none (2)
One-way street (846/10.7)	Is the street a one-way or a two-way street?	One-way (1) vs two-way (2)
Number of lanes (1183/14.9)	How many lanes are there for cars?	Many (2 +) (1) vs few (≤ 2) (2)
Presence of highway (579/7.3)	Can you see a highway (elevated or below ground) here?	This is a highway (1) vs not a highway (2)
Highway is barrier (29/19.9)	Is the highway a barrier to walking?	Cannot be overcome (1) vs can be overcome (2)

### Statistical analysis

**Fig. 2:**
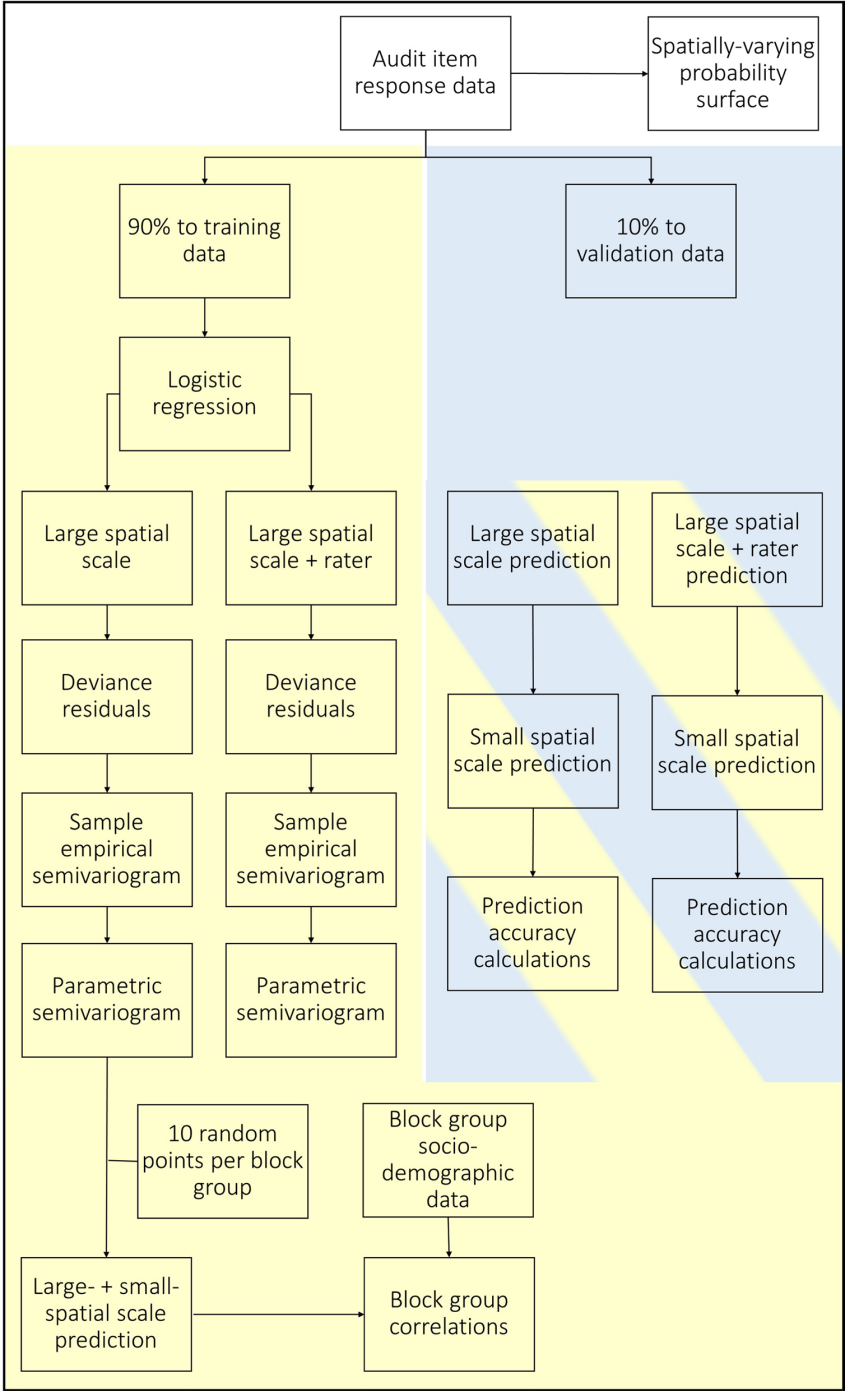
Flow chart of analysis plan to investigate the spatial properties of neighborhood audit item responses

A workflow of all statistical analyses is displayed in Fig. 2. Nonparametric, spatially varying probability surfaces of each item response = ‘Yes’/’1’ (Table 1) were created to visualize the spatial distribution of each binomially distributed item response [52]. Estimated probabilities were calculated following an isostropic, Gaussian kernel where the kernel smoothing bandwidth distance was selected via cross-validation as the distance which minimized the negative likelihood among all candidate distances [53]. Each resulting probability surface was shaded via a divergent color scheme with red and blue hue proportional to estimated probability of ‘Yes’/’1’ and white equal to the overall probability of each item. Thus, dark red areas are higher than average probability of ‘Yes’/’1’ and dark blue areas are lower than average probability of ‘Yes’/’1’.

Item-specific analysis of spatial structure and prediction accuracy with and without statistical adjustment for rater proceeded as follows. First, we divided the data into 90-10 training-validation datasets. Using the training dataset, we then fit a logistic regression model of audit item responses, adjusting for 3rd order spatial covariates only. This detrending for large spatial scale relationships is recommended to meet the spatial statistical assumption of spatial stationarity, which in this case requires that audit item response patterns depend only on their relative positioning between one another and not their absolute positioning within the study region [31]. Next, we fit the same model of audit item responses with the addition of rater as an additional covariate (i.e., rater identity as a 4-level dummy code). Adjustment for rater will allow us to investigate whether variation in audit item responses by rater influences spatial prediction performance of audit item response patterns. Deviance residuals of each model were then assessed for spatial structure. The full model with notation for the Deviance residual was as follows: 
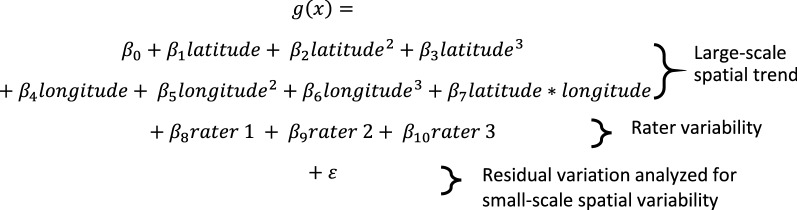
where the function *g*(**x**) is the logit of the probability of the audit response = ‘1’/’Yes’, latitude and longitude values were centered (i.e., mean = 0) in anticipation of subsequent algorithm convergence issues and to minimize polynomial estimate collinearity, rater 1–3 are dummy coded with rater 4 as the reference level, and ε represents the residual which is subsequently analyzed for small-scale spatial structure. Collectively, β 0–7 describe the large-scale spatial trend, β 8–10 rater variability, and ε variability in audit item responses not explained by 3rd order spatial trends or raters, but which could contain spatially autocorrelated audit item responses that can be used to predict unknown residuals as a function of known residual values and the distances between known and unknown values.

Experimental semivariograms of Deviance residuals within the training dataset were calculated for each audit item, with and without adjustment for rater, using identical parameters (see Additional file 1 "Supplementary methods of spatial analyses": for details of spatial analyses). Local Ordinary Kriging (OK) was performed to predict Deviance residuals from audit locations within the validation dataset based on Deviance residuals and estimated covariance parameters from theoretical semivariograms of the validation dataset. Local OK, as opposed to global OK, was performed for practical reasons; Kriging based on all points within the validation dataset was computationally prohibitive and likely statistically unnecessary given the estimated ranges that were oftentimes far shorter than 13.2 km, let alone 26.4 km (Table 2). The local OK radius was set at 1.3 km (1/10th max semivariogram distance) or the minimum distance required to include 30 training points in the prediction of the Deviance value of the specific validation location, as recommended [54]. Others have used similar “Regression Kriging” or “Kriging with External Drift” methods based on manual detrending [44, 45, 55, 56].

**Table 2:** Small-scale spatial properties of neighborhood audit item responses, Essex County, NJ

Audit item	Detrending	Theoretical semivariogram	Nugget	Partial sill/*b*	Range (km)/*p*	RMSPE	% RMSPE change (of rater adjustment)	Evidence of anisotropy
Garbage	3rd order spatial	Sine hole-Matern	0.935	0.063, 0.13	4.731, 0.871	0.3940		None
	3rd order spatial + rater	Sine hole-Matern	0.727	0.063, 0.104	4.385, 0.795	0.3376	–14.31	None
Abandoned Cars	3rd order spatial	Sine hole-Sine hole	0	0.012, 0.007	1E−6, 1E−6	0.0491		NA
	3rd order spatial + rater	Spherical-Sine hole	6.80E−04	0.16, 0.002	1E−6, 0.762	0.0489	− 0.32	NA
Building conditions ≥ moderate	3rd order spatial	Power^a^-Sine hole	0.532	1E−6, 0.0247	0.513, 2.470	0.2806		≥ Mid-range
	3rd order spatial + rater	Sine hole-sine hole	4.70E−06	0.025, 0.493	2.489, 1E−6	0.2704	− 3.63	≥ Mid-range
Yard conditions ≥ moderate	3rd order spatial	Sine hole-Matern	0.459	0.045, 0.019	2.614, 1E−6	0.2596		≥ Mid-range
	3rd order spatial + rater	Power^a^-Sine hole	0.429	1.4E−6, 0.045	0, 2.655	0.2455	− 5.44	≥ Mid-range
Dumpster	3rd order spatial	Matern-sine hole	0.501	1.0E−6, 0.007	9.052, 1.650	0.2596		≥ Mid-range
	3rd order spatial + rater	Sine hole-sine hole	0.485	0.015, 1.0E−6	0.821, 6.511	0.2567	− 1.11	≥ Mid-range
Graffiti	3rd order spatial	Sine hole-Gaussian	0.025	0.017, 0.421	1.020, 1.0E−6	0.2606		≥ Mid-range
	3rd order spatial + rater	Matern-Sine hole	0.433	6.3E−5, 0.016	10.141, 1.028	0.2566	− 1.54	≥ Mid-range
Boarded/burned building	3rd order spatial	Cubic	0.032	0.306	0.231	0.2138		≥ Mid-range
	3rd order spatial + rater	Spherical-Matern	0.004	1.0E−6, 0.333	4.085, 0.094	0.2140	0.12	≥ Mid-range
Outdoor seating	3rd order spatial	Sine hole	1.131	0.015	4.405	0.4222		None
	3rd order spatial + rater	Sine hole-spherical	0.006	0.011, 1.089	4.385, 1.0E−6	0.4078	− 3.41	None
Team sports	3rd order spatial	Power^a^-sine hole	0.1658	8.6E−5, 0.023	2, 6.4E−5	0.1352		None
	3rd order spatial + rater	Power^a^	0.186	8.70E−05	2	0.1349	− 0.22	None
Yard Decorations	3rd order spatial	Sine hole-gaussian	1.095	0.046, 0.146	4.591, 0.649	0.4208		None
	3rd order spatial + rater	Sine hole-Matern	0.023	0.048, 1.141	4.652, 0.278	0.4003	− 4.87	None
Fences	3rd order spatial	Gaussian-Exponential	0.65	0.109, 0.200	10.758, 0.435	0.3443		≥ Mid-range
	3rd order spatial + rater	Sine hole-matern	4.10E−05	0.064, 0.826	10.723, 0.351	0.3311	− 3.81	≥ Mid-range
Sidewalk present	3rd order spatial	Matern-sine hole	0	0.904, 0.069	1.869, 3.022	0.2952		≥ Mid-range
	3rd order spatial + rater	Power^a^-matern	0.558	2.70E−4, 0.381	2, 1.683	0.3035	2.8	≥ Mid-range
Complete sidewalk	3rd order spatial	Gaussian-sine hole	0.443	0.164, 0.015	7.648, 2.542	0.2512		None
	3rd order spatial + rater	Gaussian-spherical	0.432	0.107, 0.070	8.959, 10.3981	0.2511	− 0.01	None
Sidewalk condition	3rd order spatial	Gaussian-Sine hole	1.225	1.8E−6, 0.029	1.0E−6, 2.409	0.4403		None
	3rd order spatial + rater	Exponential-sine hole	1.106	1.0E−6, 0.034	4.139, 2.495	0.4297	− 2.39	None
Sidewalk width	3rd order spatial	Gaussian-exponential	0.019	0.637, 0.005	0.099, 1.0E−6	0.2870		None
	3rd order spatial + rater	Cubic-matern	0.11	1.0E−6, 0.538	4.782, 0.109	0.2779	− 3.18	None
Sidewalk from Curb Distance	3rd order spatial	Sine hole-Spherical	0.04	0.076, 0.011	1.0E−6, 3.918	0.0938		≥ Mid-range
	3rd order spatial + rater	Sine hole-exponential	0.036	0.012, 0.076	3.953, 1.0E−6	0.0936	− 0.15	≥ Mid-range
Car Obstruction	3rd order spatial	Gaussian-cubic	0.09	9.5E−4, 0.320	1.0E−6, 0.251	0.2003		≥ Mid-range
	3rd order spatial + rater	Spherical-spherical	0.006	0.368, 0.034	0.208, 1.0E−6	0.1996	− 0.32	≥ Mid-range
Garbage can obstruction	3rd order spatial	Power^a^	0.388	1.60E−04	2	0.1979		None
	3rd order spatial + rater	Power^a^-sine hole	0.372	1.4E−4, 0.014	2, 1.0E−6	0.1974	− 0.27	None
Pole or Sign obstruction	3rd order spatial	Power^a^	0.338	5.90E−04	2	0.1620		≥ Mid-range
	3rd order spatial + rater	Power^a^-sine hole	0.018	6.2E − 5, 0.298	2, 1.0E−6	0.1624	0.29	≥ Mid-range
Other obstruction	3rd order spatial	Spherical-gaussian	6.10E−04	2.2E−4, 0.495	1.0E−6, 0.067	0.2450		None
	3rd order spatial + rater	Matern-matern	2.80E−04	1.6E−4, 0.491	1.0E−6, 0.068	0.2427	− 0.93	None
Curb Cuts	3rd order spatial	Matern-sine hole	9.00E−09	1.345, 0.026	0.094, 4.131	0.4751		None
	3rd order spatial + rater	Sine hole-Power^a^	1.186	6.1E−3, 0.030	4.303, 0.360	0.4587	− 3.45	None
Clear Intersection	3rd order spatial	Sine hole-Matern	3.30E−05	0.028, 1.272	12.078, 0.409	0.4148		None
	3rd order spatial + rater	Sine hole-matern	9.20E−05	0.028, 1.271	11.815, 0.410	0.4143	− 0.12	None
Pedestrian Crossing Sign	3rd order spatial	Spherical-Cubic	0.383	1.0E−6, 0.494	6.008, 0.258	0.3739		None
	3rd order spatial + rater	Spherical-Matern	0.242	1.0E−3, 0.630	1.0E−6, 8.550	0.3747	0.2	None
Pedestrian Signal	3rd order spatial	Spherical-matern	0.063	6.8E−4, 0.713	1.0E−6, 0.148	0.3348		≥ Mid-range
	3rd order spatial + rater	Matern-matern	0.117	4.5E−3, 0.646	8.045, 0.1523	0.3326	− 0.67	≥ Mid-range
Pedestrian crossing marks	3rd order spatial	Sine hole-Matern	0.649	0.074, 0.416	8.039, 0.991	0.3634		≥ Mid-range
	3rd order spatial + rater	Sine hole-matern	0.597	0.068, 0.447	7.660, 0.988	0.3676	1.15	≥ Mid-range
Type of pedestrian crosswalk marks	3rd order spatial	Sine hole-gaussian	0.669	0.053, 0.457	3.671, 0.259	0.3582		Yes, E-SE
	3rd order spatial + rater	Sine hole-matern	0	0.052, 1.124	3.672, 0.187	0.3530	− 1.44	Yes, E-SE
Traffic signal type	3rd order spatial	Sine hole-cubic	0.8	0.119, 0.215	11.930, 0.631	0.3786		None
	3rd order spatial + rater	Matern-spherical	0.779	0.174, 0.226	11.967, 0.583	0.3763	− 0.59	None
One-way Street	3rd order spatial	Exponential-matern	0.081	1.0E−6, 0.462	6.700, 0.146	0.2397		≥ Mid-range
	3rd order spatial + rater	Sine hole-exponential	0.005	0.041, 0.491	0.182, 0.100	0.2412	0.62	≥ Mid-range
Number of lanes	3rd order spatial	Exponential-sine hole	0.493	0.188, 0.057	0.690, 2.295	0.3066		≥ Mid-range
	3rd order spatial + rater	Matern-spherical	0.22	0.364, 0.155	0.210, 3.020	0.2880	− 6.09	≥ Mid-range
Presence of highway	3rd order spatial	Power^a^-matern	0.126	1.9E−4, 0.236	2, 0.738	0.1611		≥ Mid-range
	3rd order spatial + rater	Power^a^-matern	0.126	1.9E−4, 0.236	2, 0.737	0.1616	0.31	≥ Mid-range
Highway is barrier	3rd order spatial	Cubic-sine hole	0	0.772, 0.206	0.412, 2.597	0.4451		≥ Mid-range
	3rd order spatial + rater	Sine hole-Gaussian	0.043	0.180, 0.641	2.439, 1.0E−6	0.4690	5.37	≥ Mid-range

All kriging was local, with radius set at 1.3 km (approx 1/20th of largest distance between points) or the shortest distance to include 30 neighbors in Kriging calculations

^a^ Power semivariogram *b* and *p* are not partial sill and range values and cannot be compared to values of other semivariograms

The assumption of spatial isotropy, or invariance of spatial structure as a function of direction between locations, was assessed for each set of Deviance residuals by fitting directional-specific experimental and theoretical semivariograms via the same procedures and parameters used to fit omnidirectional semivariograms. Eight separate directions, uniformly dividing the unit circle (0°/180°, 22.5°/202.5°, 45°/225°, etc.), were specified and anisotropy assessed visually based on experimental and theoretical semivariograms. Qualitative assessments of anisotropy violations were made—‘None’, ‘≥ Mid-range’, ‘Yes’—based on a combination of semivariogram behavior about the nugget, sill, and range. Comparing *within* directional semivariograms as well as *between* directional and omnidirectional semivariograms, ‘None’ indicates nearly identical semivariograms, ‘≥ Mid-range’ indicates very similar semivariogram properties within estimated ranges of the majority of semivariograms, and ‘Yes’ indicates evidence of anisotropy (with directional violation noted).

Prediction accuracy was measured by root mean squared prediction error (RMSPE) calculated from predicted and observed audit responses within the validation datasets of each audit item model (with and without adjustment for rater). Kriging-predicted audit item response residuals were calculated by back-transforming the Kriging-estimated Deviance residuals to raw residuals. Validation dataset trend components were calculated by the score method and using the model built from the training dataset (e.g., mean response, 3rd order spatial trend, and rater adjustment if applicable). The predicted audit item response of the validation dataset was obtained by summing the Kriging-predicted residual and logistic regression-scored trend component. Percent change of RMSPE comparing models adjusted for rater to those not rater-adjusted were calculated; negative% change of RMSPE indicating that adjustment for rater yielded lower audit item predictive error. Area Under the Curve (AUC) of the Receiver Operator Curve (ROC) of the validation datasets were calculated to assess the overall predictive ability of the above modeling. In these ROC AUC calculations of the validation dataset, observed audit item response was the dependent variable and predicted response probability (e.g., Kriging-predicted residual + logistic regression-scored trend) was the single independent variable measured as a continuous variable. Percent change of ROC AUC comparing models adjusted for rater to those not rater-adjusted were calculated where, contrary to above, positive% change of ROC AUC indicated that adjustment for rater yielded greater audit item predictive ability. The following interpretations of ROC AUC accuracy were used: ROC = 0.5 ‘*None*’, 0.7 ≤ ROC AUC < 0.8 ‘*Acceptable*’, 0.8 ≤ ROC AUC < 0.9 ‘*Excellent*’, ROC AUC ≥ 0.9 ‘*Outstanding*’ [57].

Lastly, correlations between predicted audit item responses and select block group-level census variables were calculated to explore relationships between various audit items, sociodemographic, and neighborhood features. Audit item response predictions were based on the full model and a separate set of 10 locations randomly generated within each of Essex County’s 671 block groups (i.e., 6710 prediction locations). As correlations were block group-level, the 10 audit item response values per a block group were treated as imputed data and analyzed within a multiple imputation framework, as has been previously done [27]. Block group-level percentage of non-Hispanic African American residents (% AA), percentage of HispanicLatinx residents /(% Latinx), percentage of non-Hispanic White residents (% NHW), percentage of residents who moved within the previous year, percentage of working-age people who walk to work, median year of homes’ construction, median gross rent, median owner occupied home value, and population density were from the 2011–2015 American Community Survey [58]. Data management and analyses were conducted within 64-bit, desktop versions of ArcGIS v10.5, SAS v9.4, and the *Spatstat* package within R v3.5.2 [59–61]. All spatial data were projected Alber’s Equidistant Conic to preserve accurate distance calculations.

## Results

**Fig. 3:**
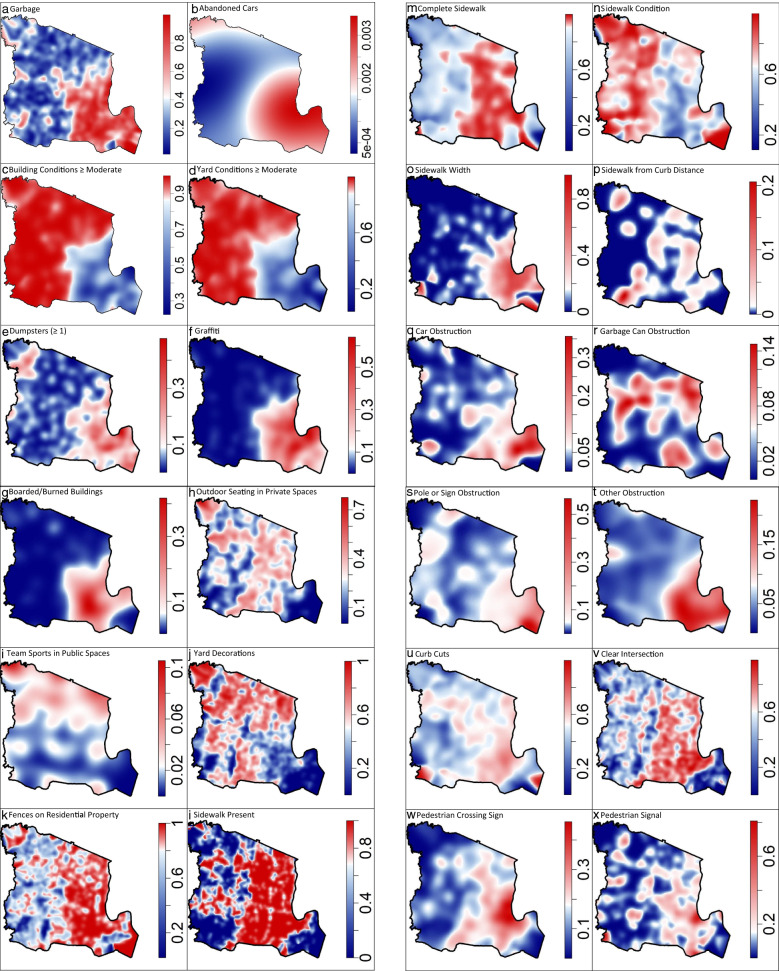

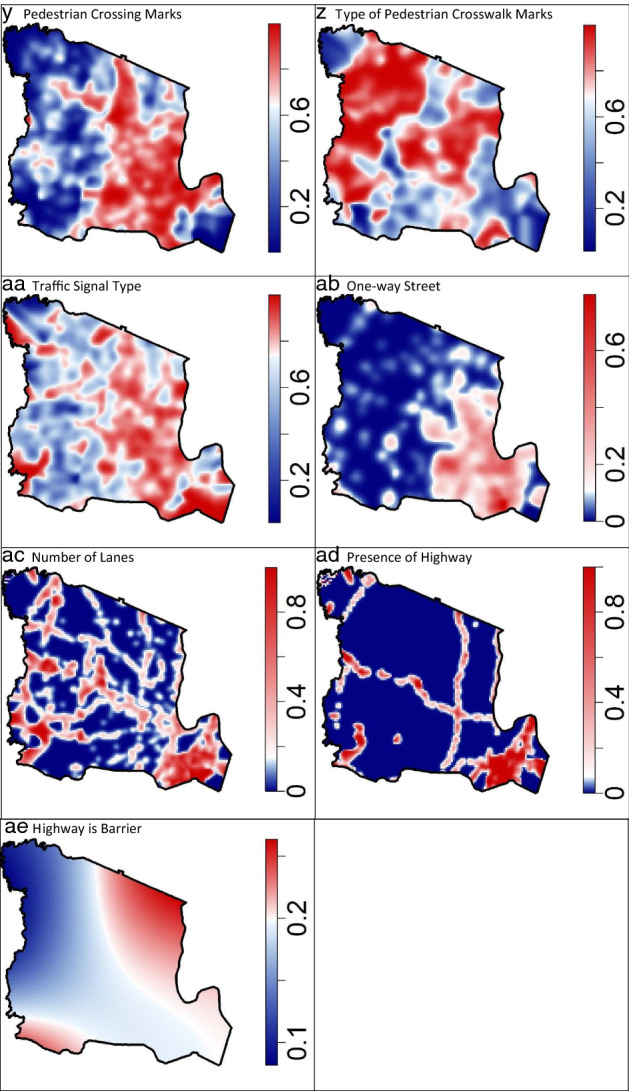
**a**–**ae** Nonparametric, spatially varying probability surfaces of each audit item response =”Yes”/”1”, Essex County, NJ^1^. ^1^ Shaded via a divergent color scheme with red and blue hue proportional to estimated probability of ‘Yes’/’1’ and white equal to the overall probability of each item (Table 1)

Spatially varying probability surfaces of each audit item’s response indicate largely unique geographic patterns across audit items. There are, however, notable trends especially when audit item response patterns are considered within item groupings. Presence of garbage, abandoned cars, < moderate building or yard conditions, dumpsters, graffiti, and boarded up/burned out buildings appear to be concentrated in the southeast portion of the study region (Newark), and hence, appear to correlate with another (Fig. 3a–g). Presence of outdoor seating, team sports in public spaces, and yard decorations tend to occur at lower than average probabilities within the southeast, but also appear to be highly variable throughout the remainder of the study region. Sidewalk presence and complete sidewalks were estimated to be higher towards the eastern portion of the region. Among areas with sidewalks, sidewalks of good condition tended to be less commonly found within the southeast. Sidewalks wider than 4 feet were more likely in the eastern portions. Sidewalks obstructed by either a car, pole or sign, or something else (besides garbage can) were more likely in the southeast of the study region. Presence of an intersection was concentrated in the more densely populated eastern section as was presence of pedestrian crossing signs, signals, and marked crosswalks.

Results of theoretical semivariogram fitting by weighted least squares regression are shown in Table 2. There are several general results worth noting from these fitted semivariograms of the detrended audit items. First, the majority of audit items were best estimated, in terms of lower sums of squares error, by nested semivariograms as opposed to a single theoretical semivariogram. The better fitting nested semivariograms indicate that audit item responses spatially autocorrelate at more than one scale. For example, the best fitting semivariograms to the 3rd order spatially-detrended response pattern of the ‘Garbage’ audit item suggests a sharp rise in semivariance from an initial nugget of 0.935 to a semivariance of 1.065 (Matern partial sill of 0.13) over a distance of 0.871 km (Matern range), then a second and flatter rise over a distance of 4.731 km (sine hole range) until the sill (1.128 = 0.935 (nugget) + 0.13 (Matern partial sill) + 0.063 (sine hole partial sill)). Second, a majority (21/31) of theoretical semivariogram nuggets of audit items additionally detrended for ‘rater’ were lower than theoretical semivariogram nuggets of audit items detrended only for 3rd order spatial relationships. As the nugget is a measure of measurement error or short-distance spatial heterogeneity, lower nuggets of rater-detrended audit item responses could indicate that rater disagreement in item responses (e.g., lower test–retest and inter-rater reliability) might increase short-distance measurement error. Third, RMSPE of rater-adjusted audit item responses were lower than RMSPE of responses adjusted for spatial trend only in 23 of 31 audit items, indicating that rater adjustment of these 23 audit items resulted in improved prediction accuracy via the cross-validation Kriging models. Fourth, several audit item experimental semivariogams best fit by nested theoretical semivariograms indicate a small nugget which coincides with a large 1st partial sill over a very small range followed by a 2nd, smaller partial sill and larger range. Audit item semivariograms characterized this way have at least one short-distance binned empirical semivariogram value with markedly low semivariance (i.e., high correlation at short distance), which leads to a fitted theoretical semivariogram that is “bent” downwards by the influence of the low variance values (Additional file 1: Figs. S1a–ae 1) and 2)). Fifth, there was no evidence of anisotropy among 13 audit items—4 neighborhood physical disorder-related, 6 sidewalk-related, 3 intersection-related—and evidence of anisotropy “≥ Mid-range” among 16 items (Additional file 1: Figs. S2a–ae 1) and 2)). As an example of potential ≥ Mid-range anisotropy, the sills (~ 0.6) and semivariogram ranges (~ 2.4–3.5 km) look nearly identical across all eight directional semivariograms of the “Building conditions ≥ Moderate” item (Additional file 1: Fig. S2c.1)) and are similar to the estimated sill (0.56) and range (1st = 0.513 km, 2nd = 2.470 km) of the omnidirectional semivariogram reported in Table 2. However, certain directional semivariances decrease as distance increases beyond these ranges (directional semivariograms of angles 0^°^/180^°^, 22.5^°^/202.5^°^, 45^°^/225^°^, 67.5^°^/247.5^°^), while others exhibit cyclical variability beyond these ranges (90^°^/270^°^, 112.5^°^/292.5^°^, 135^°^/315^°^), and one is flat beyond the range (157.5^°^/337.5^°^).

Table 3 displays the predictive accuracy of large spatial scale, small spatial scale, and rater components, as symbolized in the above equation. Regardless of rater adjustment, additional modeling of small-scale spatial relationships via Kriging results in a markedly higher ROC AUC of observed audit item responses for all audit items compared to modeling only large-scale relationships. When considering all 62 full models (e.g., large-scale + small-scale spatial modeling with or without rater adjustment), 29 models—12 neighborhood physical disorder, 9 intersection-related, 8 sidewalk-related—had ‘outstanding’ predictive accuracies (ROC AUC ≥ 0.9), indicating that 90% of observed responses for these audit items of the validation dataset could be classified as ‘Yes’/’1’ or ‘No’/’2’ based on predictions from the training dataset. Only 1 model—highway is a barrier including rater adjustment—resulted in a less than ‘Acceptable’ predictive accuracy (ROC AUC = 0.583).

**Table 3:** Prediction ability of neighborhood audit item responses by spatial scale (large/small) and rater adjustment

Audit item	Detrending	ROC AUC prediction*			
		Large-scale/Rater	+ Small-scale Kriging	%Change of rater adjustment, full equation	%Change large- vs small-scale
Garbage	3rd order spatial	0.755	0.856		13.4
	3rd order spatial + rater	0.874	0.919	7.36	5.1
Abandoned cars	3rd order spatial	0.881	0.996		13.1
	3rd order spatial + rater	0.952	0.994	− 0.2	4.4
Building conditions ≥ moderate	3rd order spatial	0.852	0.902		5.9
	3rd order spatial + rater	0.880	0.916	1.55	4.1
Yard conditions ≥ moderate	3rd order spatial	0.897	0.934		4.1
	3rd order spatial + rater	0.921	0.946	1.28	2.7
Dumpster	3rd order spatial	0.714	0.842		17.9
	3rd order spatial + rater	0.714	0.849	0.83	18.9
Graffiti	3rd order spatial	0.871	0.914		4.9
	3rd order spatial + rater	0.887	0.923	0.98	4.1
Boarded/burned building	3rd order spatial	0.851	0.914		7.4
	3rd order spatial + rater	0.850	0.915	0.11	7.6
Outdoor seating	3rd order spatial	0.562	0.735		30.8
	3rd order spatial + rater	0.679	0.767	4.35	13
Team sports	3rd order spatial	0.591	0.915		54.8
	3rd order spatial + rater	0.683	0.894	− 2.3	30.9
Yard decorations	3rd order spatial	0.667	0.825		23.7
	3rd order spatial + rater	0.708	0.852	3.27	20.3
Fences	3rd order spatial	0.710	0.884		24.5
	3rd order spatial + rater	0.736	0.897	1.47	21.9
Sidewalk present	3rd order spatial	0.814	0.950		16.7
	3rd order spatial + rater	0.814	0.943	− 0.74	15.8
Complete sidewalk	3rd order spatial	0.683	0.842		23.3
	3rd order spatial + rater	0.726	0.855	1.54	17.8
Sidewalk condition	3rd order spatial	0.662	0.771		16.5
	3rd order spatial + rater	0.697	0.775	0.52	11.2
Sidewalk width	3rd order spatial	0.830	0.897		8.1
	3rd order spatial + rater	0.831	0.901	0.45	8.4
Sidewalk from curb distance	3rd order spatial	0.586	0.978		66.9
	3rd order spatial + rater	0.724	0.914	− 6.54	26.2
Car obstruction	3rd order spatial	0.626	0.879		40.4
	3rd order spatial + rater	0.663	0.881	0.23	32.9
Garbage can obstruction	3rd order spatial	0.674	0.929		37.8
	3rd order spatial + rater	0.695	0.917	− 1.29	31.9
Pole or sign obstruction	3rd order spatial	0.592	0.900		52
	3rd order spatial + rater	0.726	0.876	− 2.67	20.7
Other obstruction	3rd order spatial	0.689	0.870		26.3
	3rd order spatial + rater	0.694	0.871	0.11	25.5
Curb cuts	3rd order spatial	0.559	0.710		27
	3rd order spatial + rater	0.669	0.716	0.85	7
Clear intersection	3rd order spatial	0.681	0.851		25
	3rd order spatial + rater	0.684	0.852	0.12	24.6
Pedestrian crossing sign	3rd order spatial	0.669	0.827		23.6
	3rd order spatial + rater	0.664	0.809	− 2.18	21.8
Pedestrian signal	3rd order spatial	0.651	0.908		39.5
	3rd order spatial + rater	0.679	0.897	− 1.21	32.1
Pedestrian crossing marks	3rd order spatial	0.731	0.871		19.2
	3rd order spatial + rater	0.725	0.860	− 1.26	18.6
Type of pedestrian crosswalk marks	3rd order spatial	0.697	0.931		33.6
	3rd order spatial + rater	0.709	0.934	0.32	31.7
traffic signal type	3rd order spatial	0.661	0.841		27.2
	3rd order spatial + rater	0.699	0.838	− 0.36	19.9
One-way street	3rd order spatial	0.789	0.931		18
	3rd order spatial + rater	0.793	0.923	− 0.86	16.4
Number of lanes	3rd order spatial	0.676	0.920		36.1
	3rd order spatial + rater	0.674	0.948	3.04	40.7
Presence of highway	3rd order spatial	0.838	0.993		18.5
	3rd order spatial + rater	0.838	0.993	0	18.5
Highway is barrier	3rd order spatial	0.563	0.833		48
	3rd order spatial + rater	0.479	0.583	− 30.01	21.7

*The area under the curve (AUC) of receiver operator curves (ROC) resulting from each item-specific logistic regression of the validation dataset was calculated where observed audit item response was the dependent variable and predicted response probability was the single independent variable measured as a continuous variable

A majority of audit items’ (18/31) predictive accuracy improved with additional adjustment for rater within the system of equations. There was variation in predictive accuracy improvement by audit item grouping with 9 of 11 neighborhood physical disorder audit items, 6 of 10 sidewalk-related, and 3 of 10 intersection-related audit items indicating improved prediction accuracy with rater adjustment. Percent improvement in ROC AUC with adjustment for rater ranges from a low of − 30.01% (Highway is Barrier, worsening prediction) to 7.36% (Garbage, improved prediction). When considering the worse predictive ability of “Highway is Barrier” as an outlier (next worse is− 6.54%), the overall average % improvement in ROC AUC with rater adjustment is 0.34%—neighborhood physical disorder-related item average = 1.7%, sidewalk-related item average = − 0.3%, intersection-related item average = − 0.6%. All 62 models that accounted for small-scale spatial variation resulted in improved spatial prediction accuracy. The largest improvements associated with small-scale spatial modeling were among sidewalk- and intersection-related items, indicating greater small-scale spatial variation in response patterns of these audit items.

**Fig. 4:**
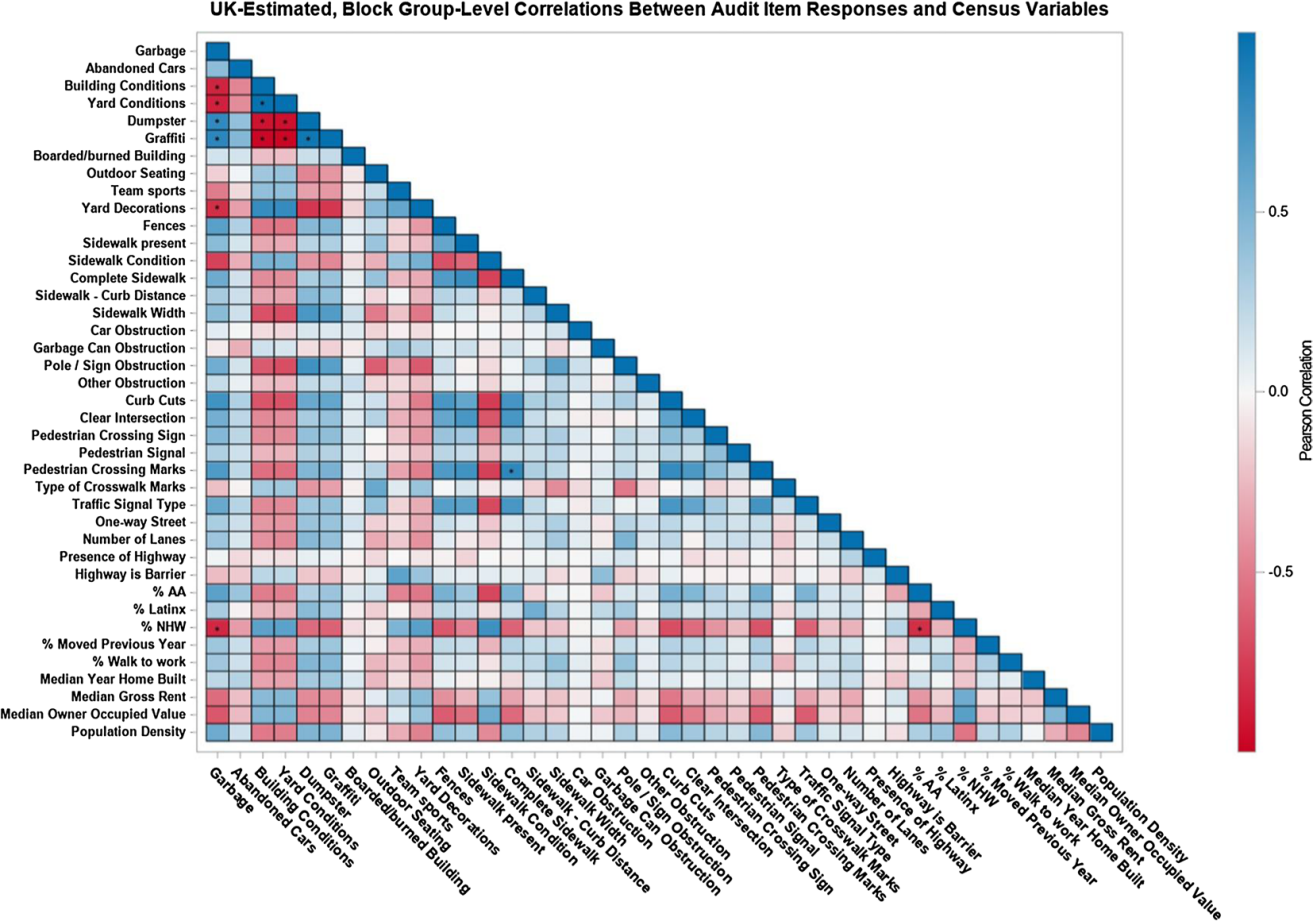
Correlation matrix of block group-level, predicted audit item responses and sociodemographic, economic, and housing characteristics^1,2^. ^1^ From the 2011–2015 American Community Survey. ^2^ Pearson correlations greater than |0.8| noted with an asterisk

Block group-level, pair-wise correlations between predicted audit item responses and sociodemographic, economic, and housing characteristics vary substantially in magnitude from near perfect correlation (r = 0.99 building conditions-yard conditions) to near zero (r = 0.03 outdoor seating-abandoned cars) (Fig. 4, Additional file 1: Table S1). Nine of the ten largest correlations (all r ≥ |0.83|) involve combinations of five neighborhood physical disorder audit items—garbage, building conditions ≥ moderate, yard conditions ≥ moderate, graffiti, dumpsters—indicating their interdependence. The ten largest correlations involving census data (|0.64| ≤ r ≤ |0.84|) include one of the three racial-ethnic composition variables with the five highest between presence of garbage and percentage non-Hispanic White (r = − 0.83), percentage non-Hispanic AA and percentage non-Hispanic White (r = − 0.81), sidewalk conditions ≥ good and percentage non-Hispanic White (r = 0.77), sidewalk conditions ≥ good and percentage non-Hispanic AA (r = − 0.71), and curb cuts and percentage non-Hispanic White (r = − 0.68). The three largest correlations involving median owner occupied home value involved the same variables—percentage non-Hispanic White (r = 0.65), garbage (r = − 0.64), and sidewalk curb cuts (r = − 0.62)—as the largest correlations involving median gross rent (percentage non-Hispanic White r = 0.56, garbage r = − 0.56, and sidewalk curb cuts r = − 0.51). The five highest correlations involving percentage of residents who moved within the previous year (|0.32| ≤ r ≤ |0.37|) were with garbage, building conditions ≥ were with building conditions ≥ moderate, yard conditions ≥ moderate, graffiti, dumpsters, and abandoned cars. The strongest correlates of the percentage of working-age people who walk to work also included dumpsters (r = 0.48), graffiti (r = 0.47), building conditions ≥ moderate (r = − 0.45), yard conditions ≥ moderate (r = − 0.44) along with sidewalk width ≥ 4 feet (r = 0.41).

## Discussion

The spatial properties (i.e., spatial trend, autocorrelation, predictive accuracy) of 32 built environment characteristics assessed via point-based virtual neighborhood audits vary by audit item, but nearly all spatial models predict audit responses with ‘outstanding’ accuracy. Sidewalk and intersection audit item responses tend to exhibit small-scale variability which indicate the need for samples that are more spatially dense compared to neighborhood physical disorder audit items. Correlations between predicted audit item response patterns and neighborhood factors indicate that block-group level neighborhood physical disorder-related items are most inter-dependent with one another as well as select sociodemographic, economic, and housing characteristics.

### Comparison with previous literature

To the best of our knowledge, our work is the first to report extensively on spatial autocorrelation and spatial prediction on a diverse set of audit items over a large spatial scale. With a few notable exceptions [25, 27–30, 62–64], neighborhood audit studies, virtual or in-person, have not reported spatial properties of audited features [25, 27–30, 62–64]. Those studies reporting any spatial properties have mainly focused on investigations of spatial autocorrelation [25, 27–30, 62, 63], with only one known study investigating spatial prediction of four neighborhood disorder audit items (reporting no predictive performance metrics) [64], and no known studies reporting spatial trend properties. Studies reporting spatial autocorrelation have tested a mix of individual audit items [28–30, 62], and neighborhood physical disorder scores from data reduction techniques that yield single values per audit location from combinations of audit item responses [25, 27]. Spatial autocorrelation ranges of neighborhood physical disorder scale scores from previous studies of four major U.S. metropolitan areas varied from 1 km to 10 km [25, 27]. These ranges from neighborhood physical disorder scores were typically larger than ranges of individual audit items of neighborhood physical disorder—presence of buildings in disrepair (0.72 km) [28], presence of parcel-level gardens (range ≈ 0.61 km) [29], block group-level garden density (range ≈ 0.40 km) [30]—observed in other studies. Two additional studies tested the spatial autocorrelation of neighborhood physical disorder scores [63], and sidewalk completeness and width [62], but did not report the distances at which values spatially correlated.

### Viability of point-based auditing

Although comparisons to the few previous studies investigating spatial properties of audit responses are difficult, this study confirms and extends previous results in various ways. All 31 audit item response patterns demonstrated the presence of spatial autocorrelation, confirming previous studies of similar audit items or constructs. This study extends previous results in finding appreciable spatial autocorrelation based on 3rd order spatially-detrended audit item response patterns; estimated Kriging parameters (i.e., nugget, partial sill, range) are from audit item response patterns that are independent of larger-scale trends across the study area. The non-zero Kriging range parameters for all audit items parallels the improved spatial prediction accuracy of models that additionally adjust for small-scale spatial variation; small-scale spatial autocorrelation exists in these audit items. Also, of note was the pattern of best-fit, nested theoretical semivariograms (opposed to single semivariograms), suggesting that small-scale spatial variation operated at least two scales. Together, these results indicate that the spatial variability of audit item response patterns spatially autocorrelate across multiple scales, suggestive of multiple processes influencing these patterns. Factors that explain multi-scale spatial variation of audit item responses are likely specific to individual audit items or audit item construct grouping (e.g., all sidewalk-related items caused by common social processes). For example, the spatial correlation of yard conditions across a region might operate at multiple scales due to variation in individual and institutional economic resources or strongly related factors (e.g., disposable income for yard care equipment, number of foreclosed/real estate owned/abandoned homes, municipal resources to care for public land, public/private disinvestment) and landscaping services (i.e., existence of such, affordability, universal servicer vs. multiple servicers coming on different days within the same street) might operate at a larger scale, whereas typical yard care practices of individuals and institutions occupying those regions or social network diffusion effects where presence of a well-kept yard influences neighbors to improve their yard (i.e., “landscape mimicry” [29, 30]) might operate more locally.

As indicated in the results, the semivariograms best fit through nesting appear to be related to instances of a few binned empirical semivariogram values with markedly lower variance (i.e., higher correlation) at short distances. One the one hand it can be argued that the high correlation of audit item responses at short distances might be expected and reflective of social processes such as those detailed above for yard conditions. On the other, more statistically problematic hand, these highly correlated observations at short distances could be indicative of raters’ characterizations of nearly-identical GSV scenes; akin to a Kriging analysis with duplicated observations which invalidates the results [65]. However, no two exact audit locations were rated more than once and less than 0.02% of semivariogram data points comprise the first two binned values of each audit item experimental semivariogram translating to very small contributions to the weighted least squares regression and fitted theoretical semivariogram. Pragmatically, it would very difficult to generate a sample of audit location points proximate enough to adequately test small-scale spatial variation while also ensuring that raters are not rating portions of the same scene more than once.

### Spatial model predictive accuracy

That approximately half of full spatial models, regardless of rater adjustment, had at least 90% predictive accuracy—and 90% of models had at least 80% accuracy —suggests that this sample of point-based audit item responses were predicted well. Echoing the above discussion on audit item response multiscale spatial variation, additional modeling of small-scale spatial variation resulted in marked improvement in prediction accuracy for all items compared to large-scale spatial prediction alone, indicating the utility of regression Kriging for spatial prediction [44, 65]. A main objective of this study was the exploration of spatial prediction performance variation due to systematic differences in rater test–retest and inter-rater agreement [32]. While a slim majority (18 of 31) of full spatial model’s predictive accuracy improved with rater adjustment, patterns of prediction improvement might exist which could aid in guiding future analyses and decisions of whether to adjust for rater. For example, an overwhelming majority of neighborhood physical disorder-related, but minority of intersection-related, audit item responses saw improved prediction accuracy with statistical adjustment for rater. Comparing these spatial predictive accuracy patterns to previous reported patterns of rater agreement reliability seems to suggest that lower reliability items (neighborhood physical disorder-related) experience greater improvement in prediction accuracy compared to higher reliability items (intersection-related) when variation in rater is accounted for in statistical models [32]. Measurement theory conventionally partitions observed variation into a component attributable to true variation, a component attributable to systematic observer error and a component attributable to random error. It follows that, for lower reliability items (i.e. items in which more observed variation is attributable to error), there is more room for spatial improvement by adjusting for rater effects. Future validity studies of these audit items should test whether item or construct validity varies with rather adjustment in greater detail. Such validation studies will be helpful in deciding whether adjusting for rater is beneficial.

### Predicted audit item response correlations

This study’s findings of moderate-strong correlations among neighborhood physical disorder audit items and weak-moderate correlations between neighborhood physical disorder items and demographic, economic, and housing characteristics corroborate previous research [3, 25, 27, 28, 63]. Neighborhood physical disorder scores have been consistently built from visually audited assessments of items similar to those measured in this study: garbage/litter, empty liquor bottles, cigarettes in the street, graffiti, defaced property, abandoned cars, building conditions, deteriorated recreational spaces, boarded/burned buildings, vacant land, barred windows [3, 25, 27, 63]. These studies have also found greater physical disorder to negatively correlate with area-level home or property value [25, 63], and positively correlate with individual-level AA race [28] and population density [27].

Although only correlational and not indicative of causal processes, relationships involving the three racial-ethnic density variables offer suggestions of areas for additional inquiry. New Jersey, and Essex County in particular, contains regions with some of the highest racial-ethnic residential segregation in the U.S. [66, 67]. High correlations suggested that block groups with higher percentages of NHW residents have less garbage, more sidewalks in good condition, and fewer curb cuts. The latter correlation might be more indicative of NHW residents’ tendency to live away from urban areas characterized by more intersections between roads and sidewalks, and hence curb cuts.

Other results of the correlation analysis deserving further attention, especially in future research involving physical disorder, is the moderate relationships between percentage of residents moving within the previous year and presence of garbage (r = 0.37), presence of graffiti (r = 0.37), building conditions ≥ moderate (r = − 0.36), yard conditions ≥ moderate (r = − 0.36), and presence of dumpsters (r = 0.32). These relationships suggest that block groups serving as destinations to greater proportions of recent residential movers are more likely to have higher physical disorder compared to areas with fewer residential movers. If holding under more rigorous analyses such results could inform research on neighborhood instability [68, 69], as well as underscore the importance of incorporating residential histories into studies involving built environment factors such as these [70–73].

Limitations of this study include the ad hoc regression Kriging method and uncertainty surrounding GSV as a reliable data source. Regression Kriging has been shown to generate estimates of the mean structure of a spatial process—large scale + small scale estimates – that are as accurate as Universal Kriging [44, 55]. However, most instances of regression Kriging involve linear, as opposed to logistic, regression of large-scale and covariate factors. No statistical methods exist within frequentist settings for Universal Kriging of binary data. Limitations of GSV data for assessment of built environment factors—unknown protocols for GSV driver routes, image acquisition, image processing, image updates; spatio-temporal patterns of image availability; suitability of environmental assessment of small or temporally variable items (e.g., garbage variation by day/time of day)—have been extensively detailed elsewhere [21, 22, 32, 74]. Of special relevance to this spatial analysis is the temporal variability of GSV scenes. An assumption of the spatial autocorrelation analyses is that temporal and spatial variability are independent of one another, which at least one previous study of spatio-temporal patterns of GSV image availability has brought into question [22]. For example, there is evidence that the GSV cars collect images in spatio-temporal batches based on whichever region the cars traverse [75], leading to spatially autocorrelated GSV image dates [22]. The influence of this relationship might could be mitigated if the spatio-temporal dependency is smooth across the study area. For example, GSV image batches that are collected and uploaded based on municipality adjacency, which would make economic sense from a transportation optimization perspective, would result in smooth changes in spatio-temporal patterns of GSV images. Regardless, these potential dependencies point to investigation of spatio-temporal prediction models of neighborhood audit responses [76], which coincide with the need mentioned above to investigate residential histories of individuals to whom GSV data will be linked in future studies. Finally, the audit items considered in this study were chosen based on their ability to be reliably observed and recorded through a standardized protocol, differentiating them from studies that prompt raters to provide their overall perception of a virtual scene’s beauty, safety, or liveliness [74, 77]. Choosing between assessing a virtual scene for identifiable visual components versus a scene’s perceived overall characteristics should not be based on whether one approach is generally superior to another, but rather the ultimate study question and planned translation of study findings. Identifying individually observable components of a virtual streetscape could motivate further studies and place-based interventions aimed at modifying the built environment as ways to improve population health [5].

## Conclusion

Specific built environment- and physical disorder-related patterns assessed using a new point-based virtual neighborhood audit method spatially autocorrelate across multiple spatial scales, both short and longer distances, indicating the potential benefit of point-based over traditional, segment-based assessment methods. An overwhelming majority of audit item spatial patterns were well-predicted by regression Kriging spatial models, albeit with mixed results for whether statistical adjustment for rater response variability improves audit item spatial prediction. Predicted audit item responses related to physical disorder—garbage, graffiti, building conditions, yard conditions, boarded/abandoned buildings, and dumpsters,—were strongly related to one another as well as distributions of racial-ethnic composition, socioeconomic indicators, and residential mobility. Among these specific items, drop-and-spin virtual neighborhood auditing is a viable alternative to segment-based methodologies.

## Supplementary information

**Additional file 1.** Supplementary methods of spatial analyses.

## Data Availability

The datasets used and/or analysed during the current study are available from the corresponding author on reasonable request.

## Abbreviations

GSV: Google Street View
U.S.: United States
NJ: New Jersey
GIS: Geographic Information System
CANVAS: Computer Assisted Neighborhood Virtual Assessment System
ROC: Receiver operator curves
AUC: Area under the curve
NA: Not applicable
OK: Ordinary Kriging
RMSPE: Root mean squared prediction error
AA: African American
NHW: Non-Hispanic White
km: Kilometer
