# Supplementary material for: Spatial predictive properties of built environment characteristics assessed by drop-and-spin virtual neighborhood auditing
Source: Int J Health Geogr. 2020 May 29;19:21. doi: 10.1186/s12942-020-00213-5 (PMC7257196; doi:10.1186/s12942-020-00213-5)
Supplement: Supplementary file 1 — Additional file 1. Supplementary methods of spatial analyses. [file 12942_2020_213_MOESM1_ESM.pdf]

Spatial predictive properties of socio-physical environment characteristics assessed by drop-and-spin virtual neighborhood auditing

Supplementary Methods

Experimental semivariograms of Deviance residuals within the training dataset were calculated with the following parameters: classical semivariance, bin size = 0.1 km, maximum distance for semivariance calculations = 13.2 km (13.2 km ≈ half the maximum distance observed between the two most distant audit locations) [1, 2]. Theoretical semivariograms were fit to experimental semivariograms via likelihood-based weighted least squares regression, where weights are proportional to the number of audit location pairings within each bin. To allow for more complex spatial structure, 2-level nesting of theoretical semivariogram models were assessed[3]. Sine hole, power, Gaussian, Matern, cubic, exponential, or spherical functional forms were considered. Models were chosen based on whichever functional form minimized the sums of square errors fit to the experimental semivariogram. Likelihood-based methods allowed for empirical comparisons of fit between numerous potential combinations of theoretical semivariograms.

Although it would have been ideal to utilize Universal Kriging (UK) for spatial and rater detrending within the system of equations that also estimate spatial covariance parameters and response prediction, no known software package performs such analyses of binomial data, with likelihood-based estimation of semivariogram model parameters, within frequentist settings. Those common software employing UK methods either detrend by ordinary least squares only (e.g., R's geoR), do not accommodate simultaneous estimation of variogram parameters via likelihood-based statistics (e.g., R's gstat), or allow these features but via complicated and computationally intensive Bayesian methods (e.g., R's geoRglm).

Supplementary Results

Figure S1.a-ae 1) (3rd order spatial detrending only) and 2)(3rd order spatial detrending + rater adjustment) experimental and best-fit theoretical omnidirectional semivariograms of neighborhood audit item responses from approximately 8000 locations, Essex County NJ.

a.1) Garbage, 3rd order spatial detrend

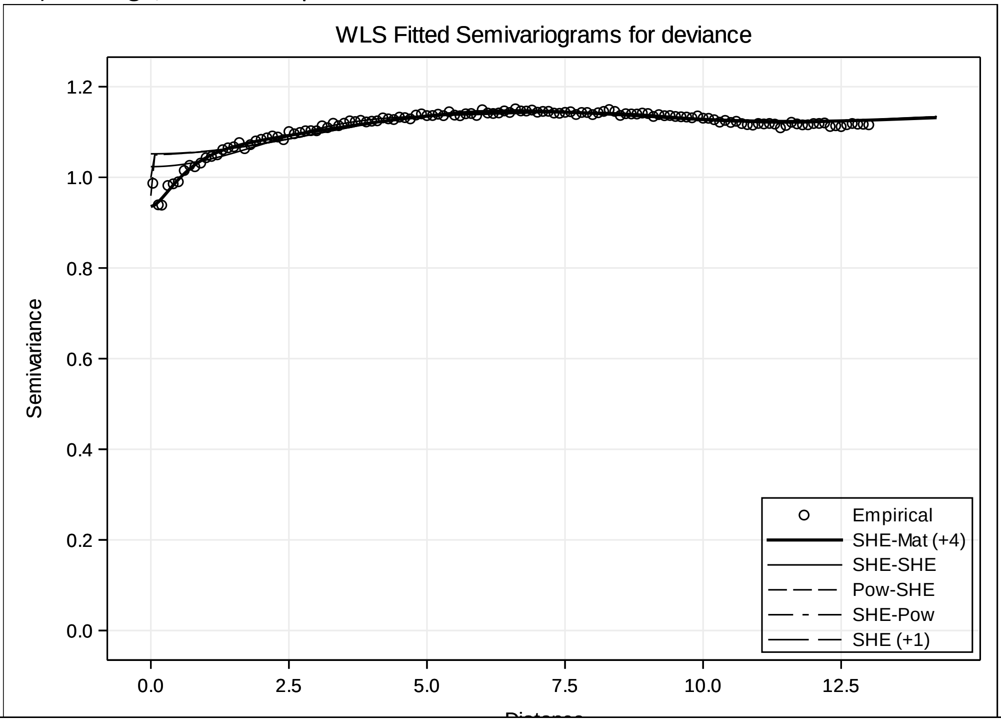

a.2) Garbage, 3rd order spatial detrend + rater adjustment

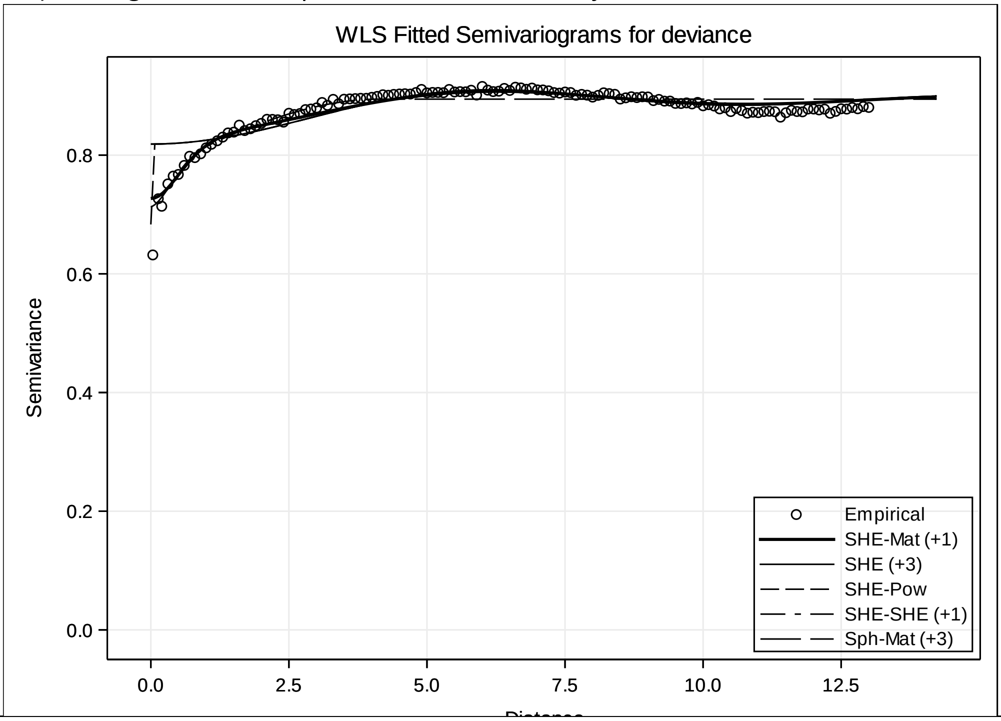

b.1) Abandoned Cars, 3rd order spatial detrend

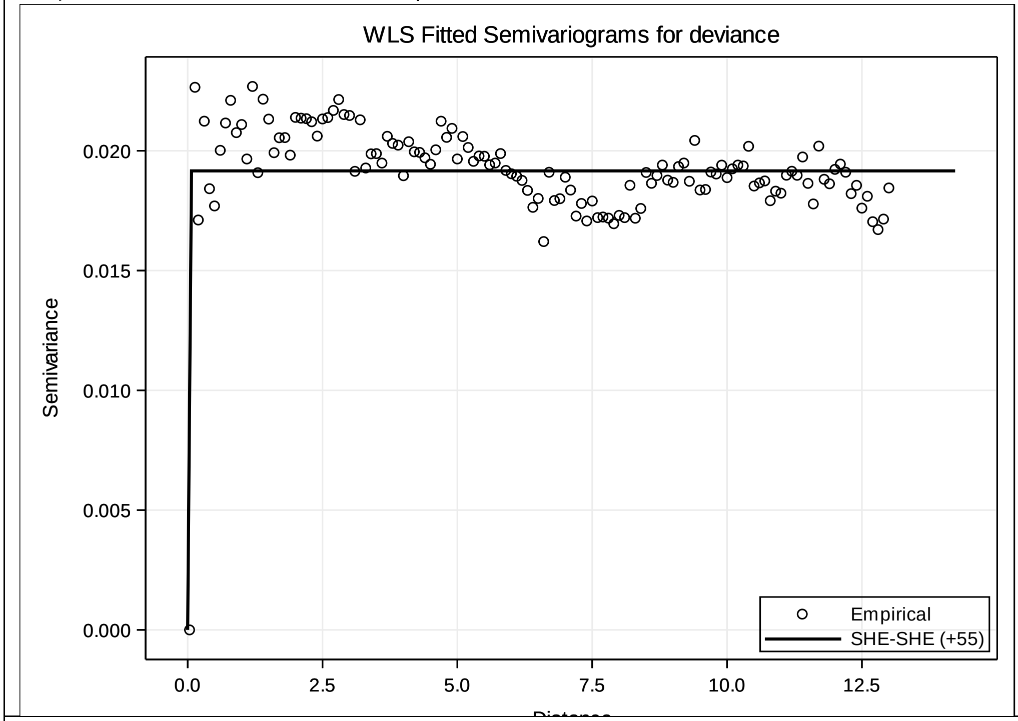

b.2) Abandoned Cars, 3rd order spatial detrend + rater adjustment

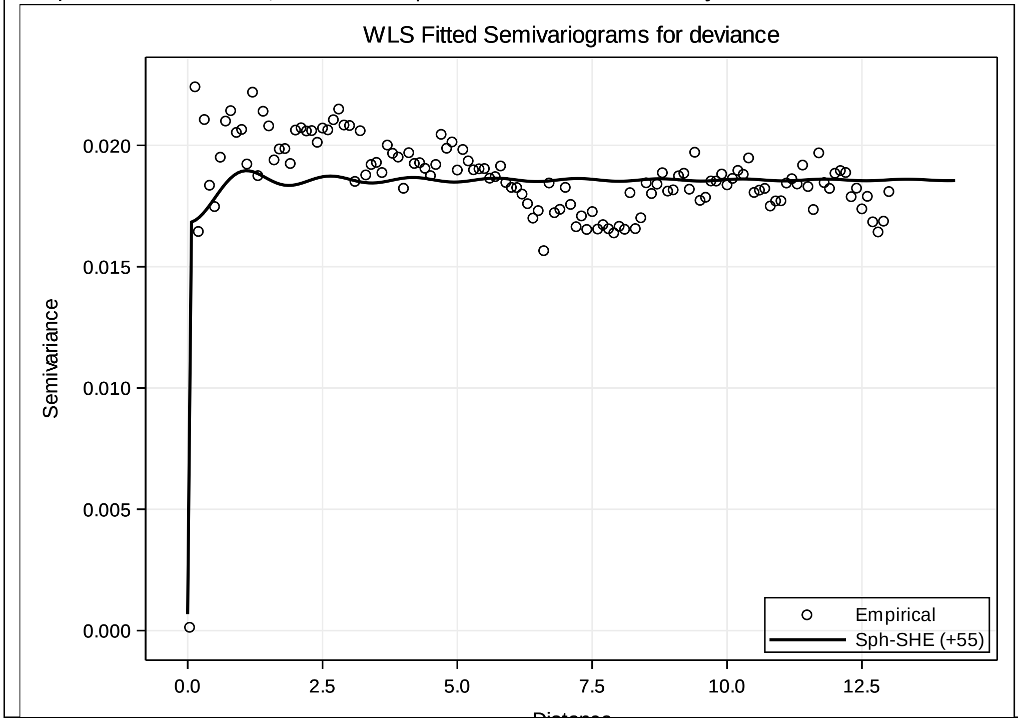

c.1) Building Conditions ≥ Moderate, 3rd order spatial detrend

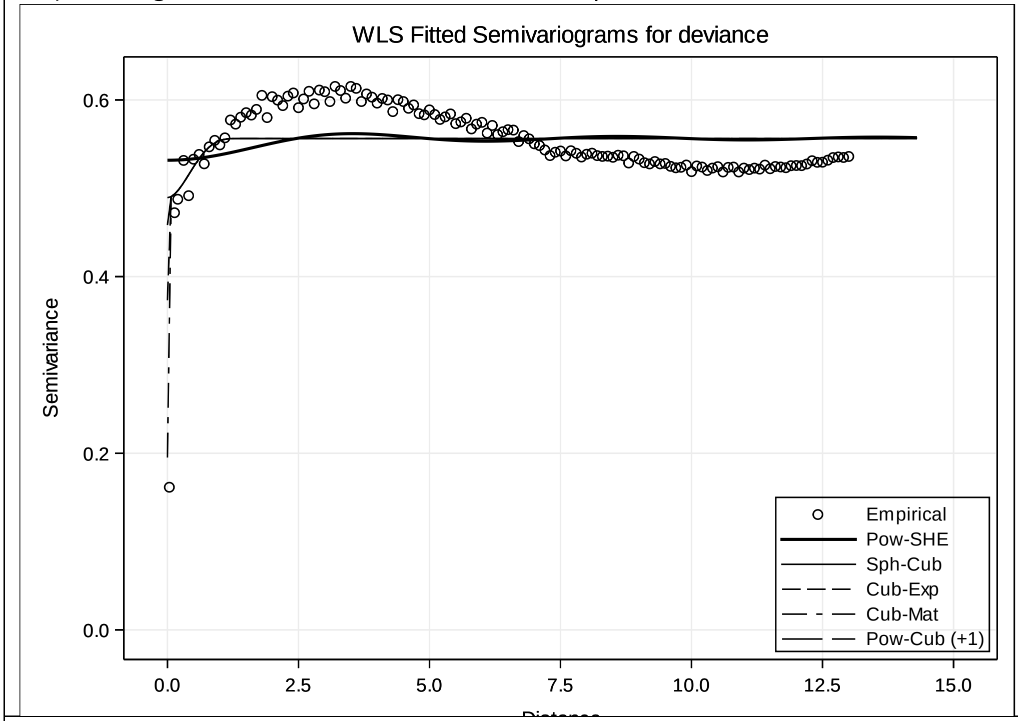

c.2) Building Conditions ≥ Moderate, 3rd order spatial detrend + rater adjustment

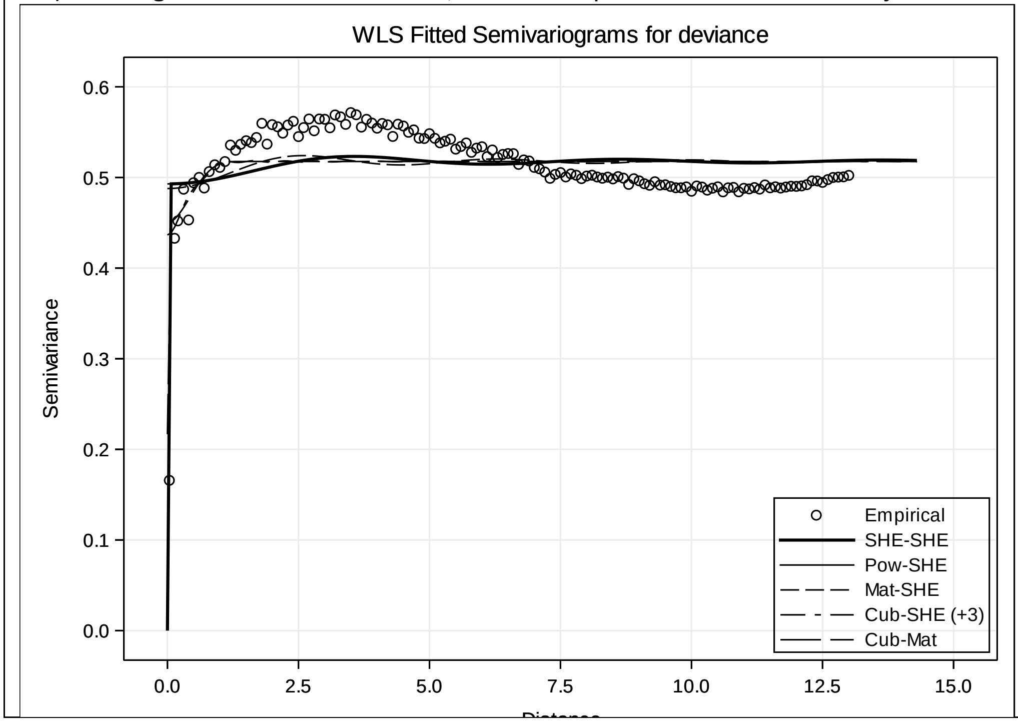

d.1) Yard Conditions ≥ Moderate, 3rd order spatial detrend

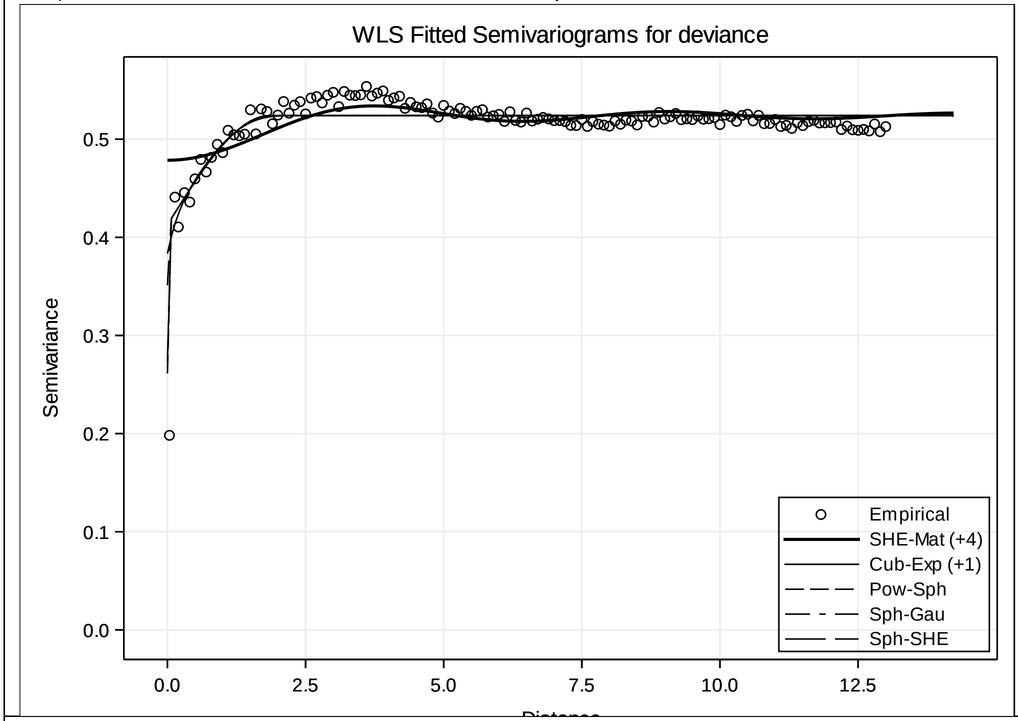

d.2) Yard Conditions ≥ Moderate, 3rd order spatial detrend + rater adjustment

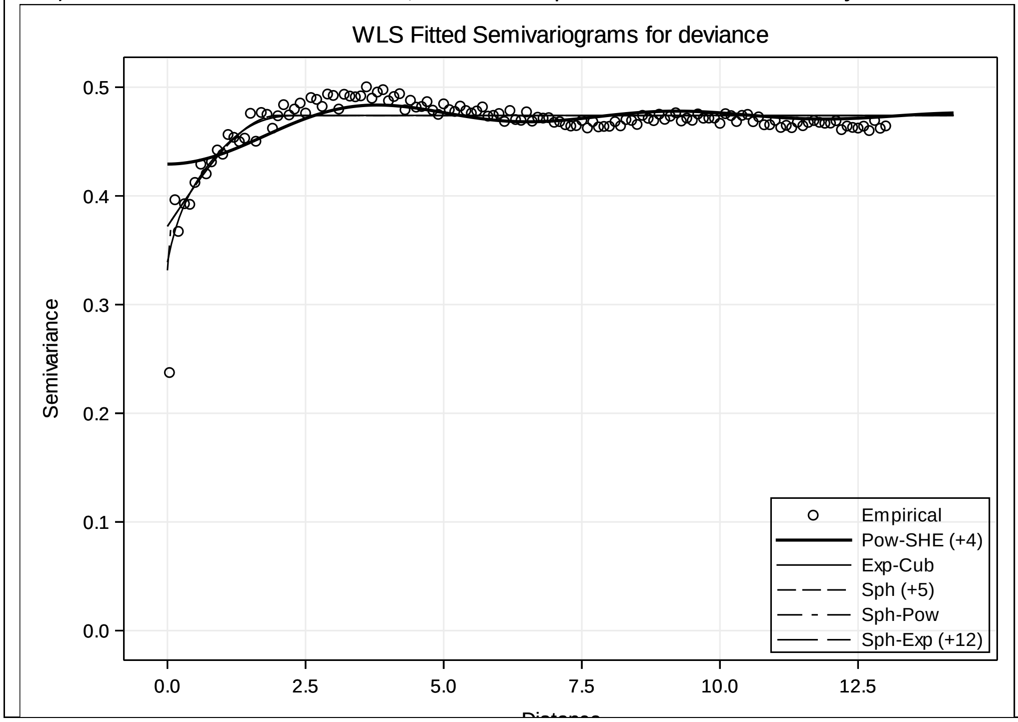

e.1) Dumpsters, 3rd order spatial detrend

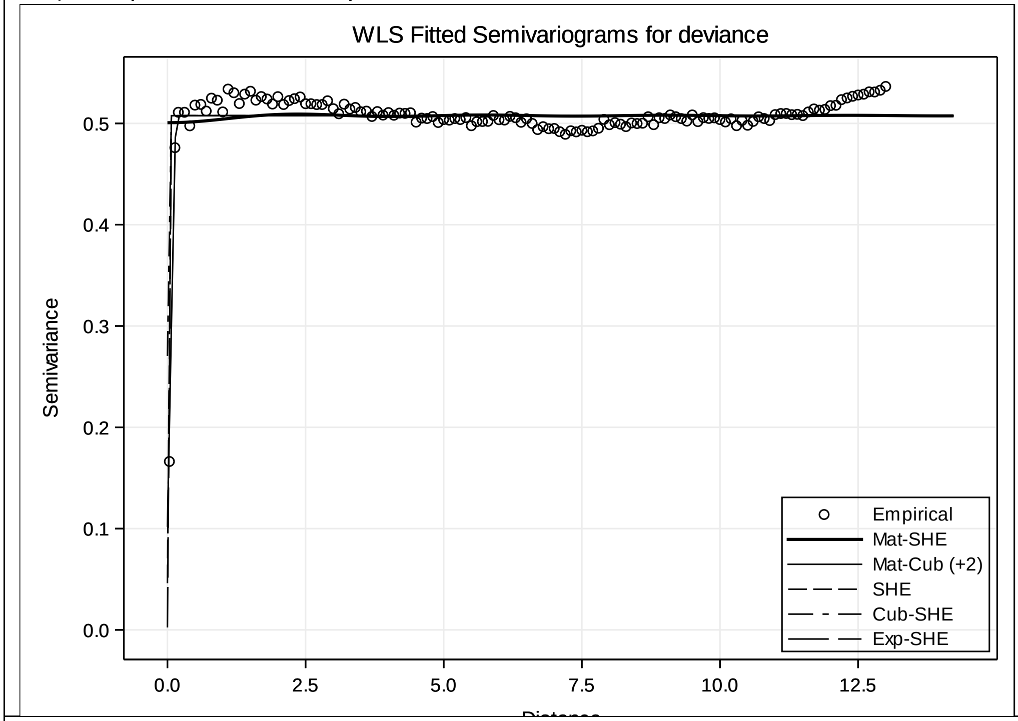

e.2) Dumpsters, 3rd order spatial detrend + rater adjustment

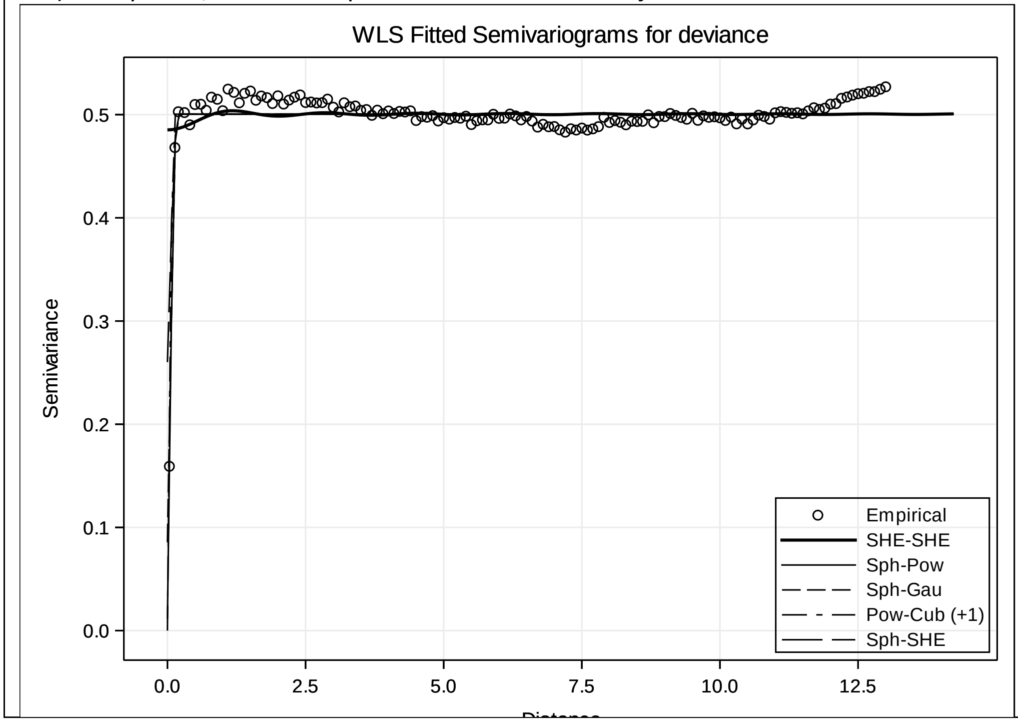

f.1) Graffiti, 3rd order spatial detrend

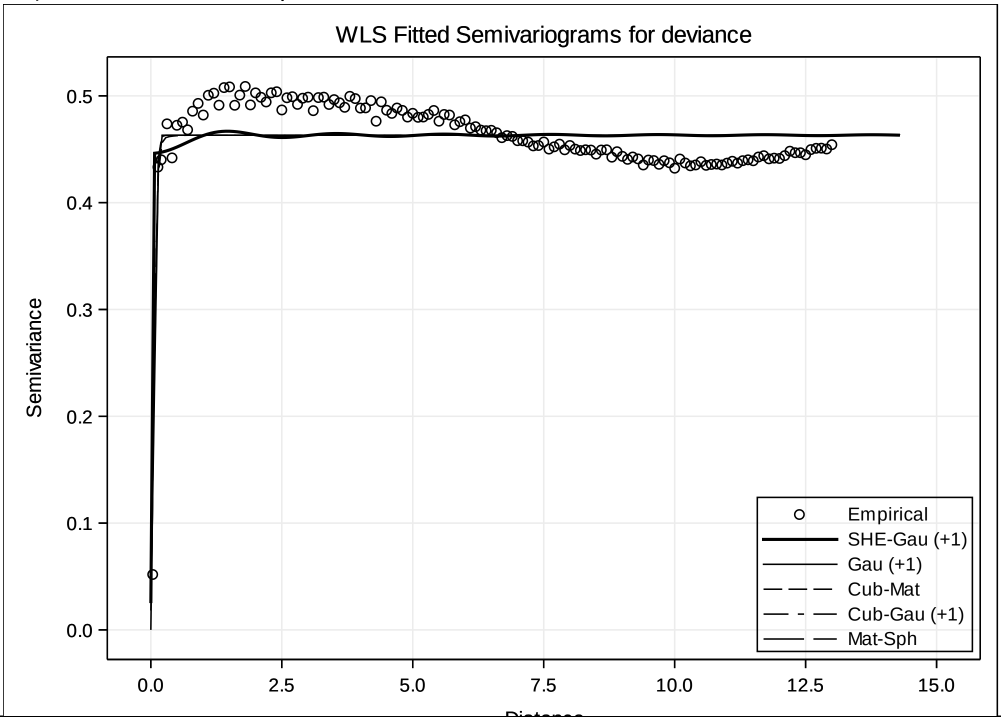

f.2) Graffiti, 3rd order spatial detrend + rater adjustment

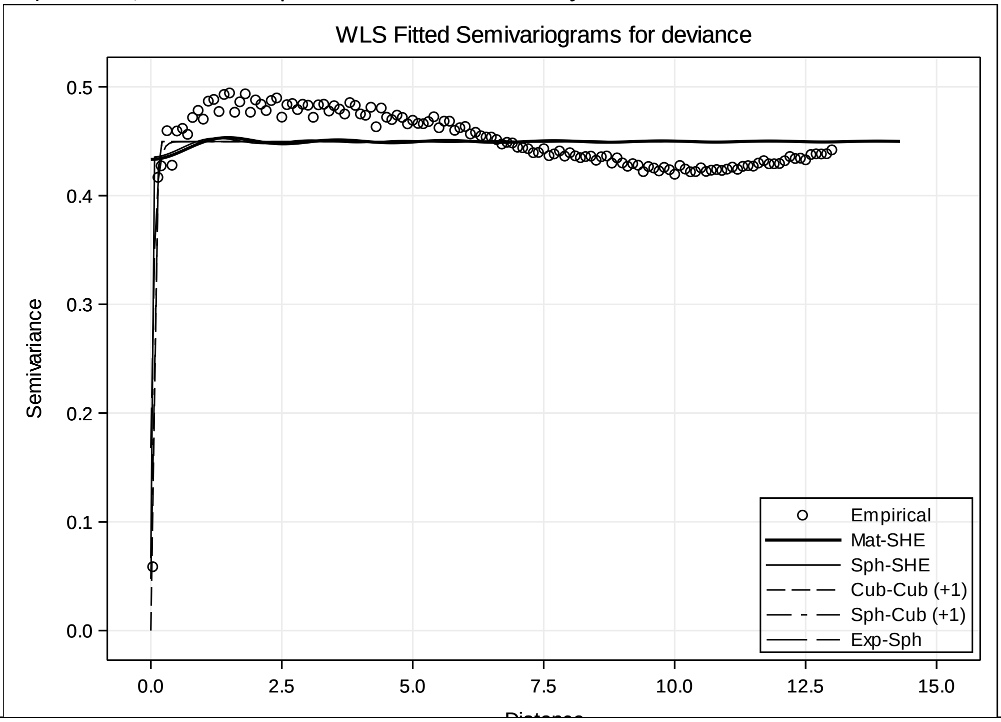

g.1) Boarded/burned Buildings, 3rd order spatial detrend

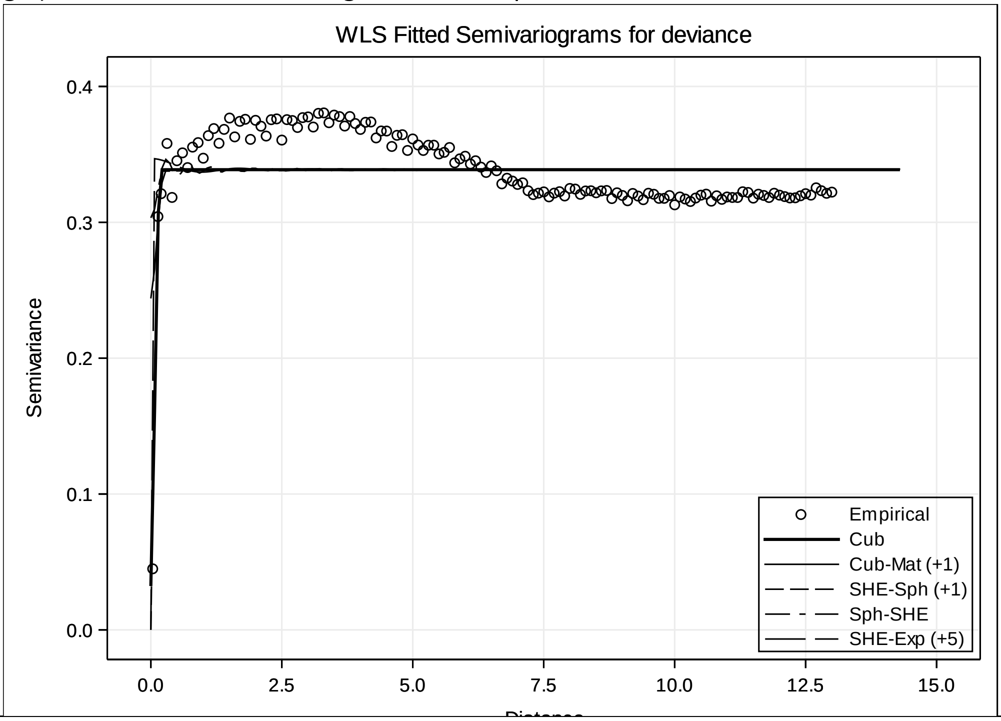

g.2) Boarded/burned Buildings, 3rd order spatial detrend + rater adjustment

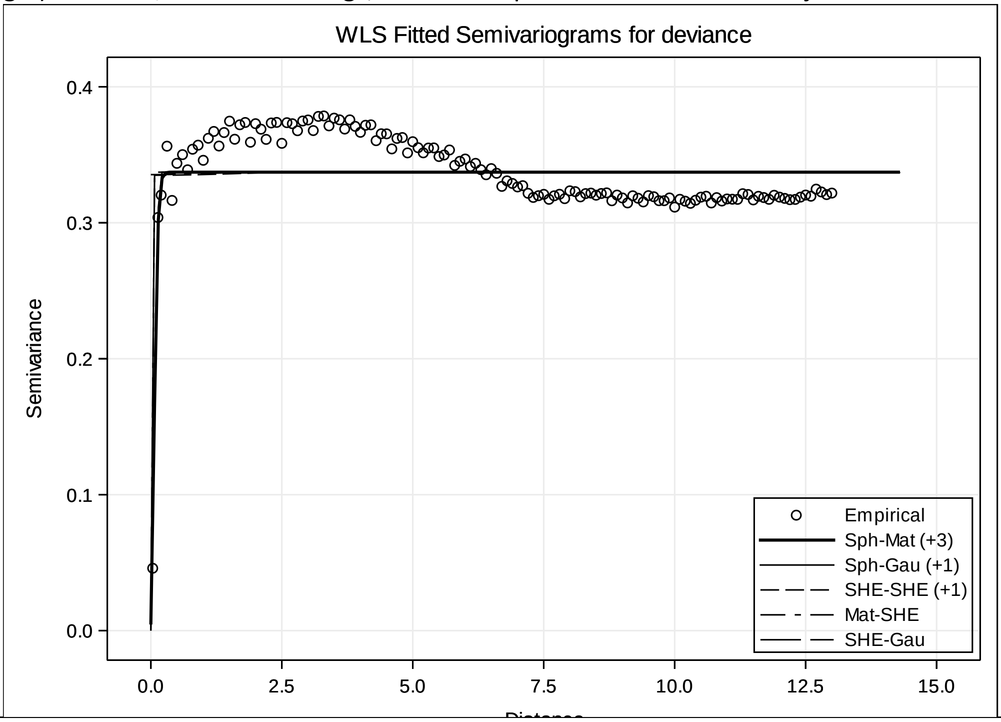

h.1) Outdoor Seating, 3rd order spatial detrend

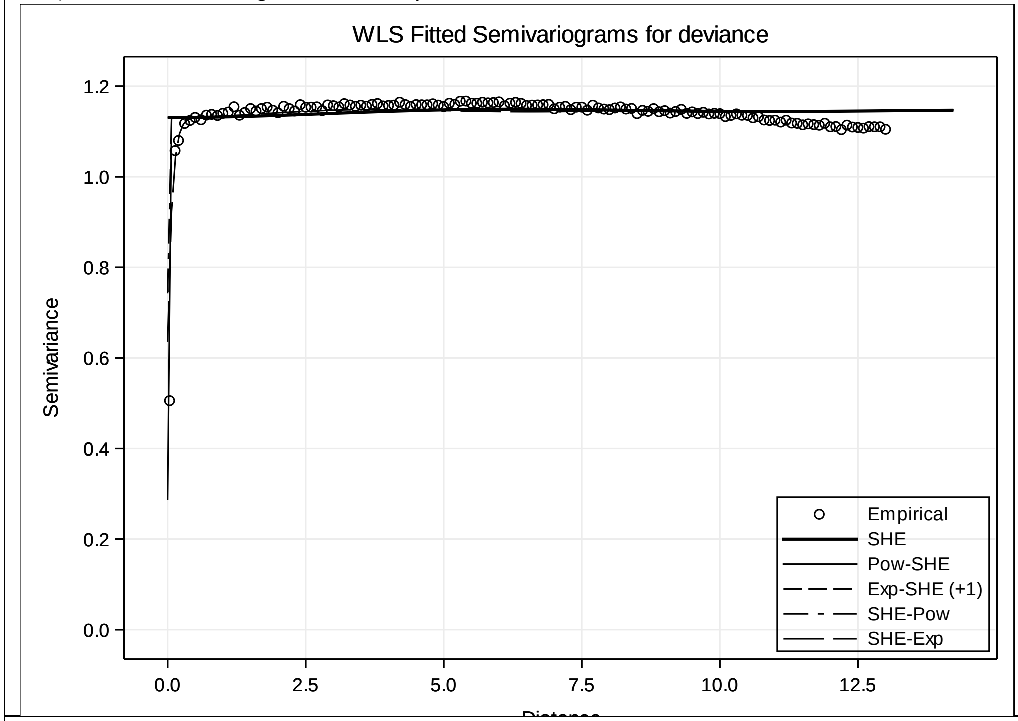

h.2) Outdoor Seating, 3rd order spatial detrend + rater adjustment

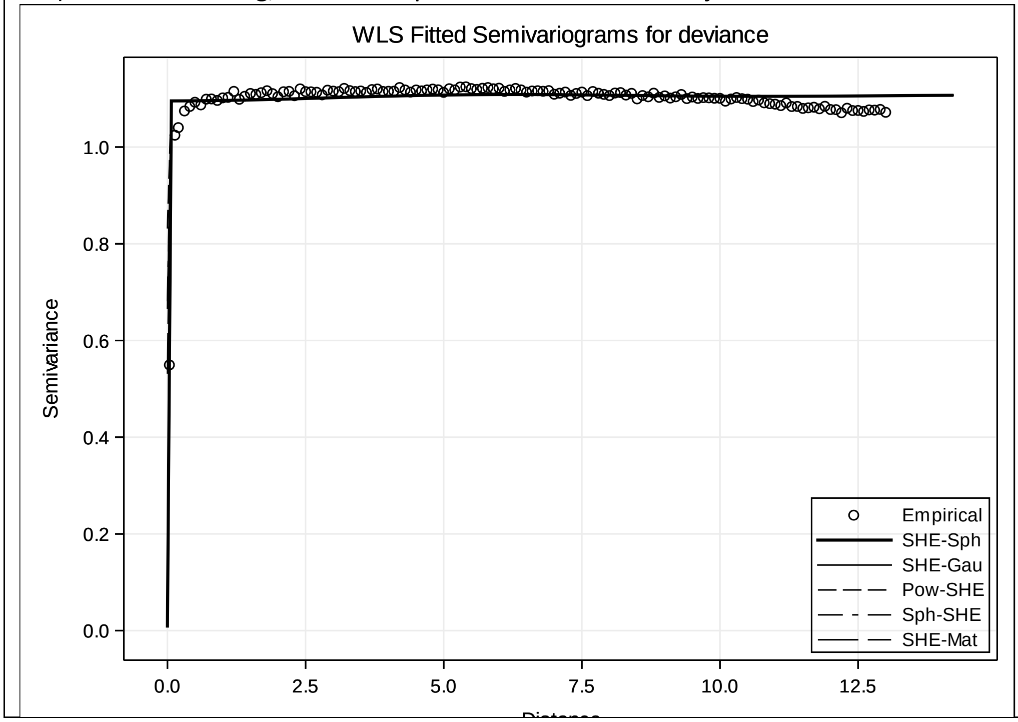

i.1) Team Sports, 3rd order spatial detrend

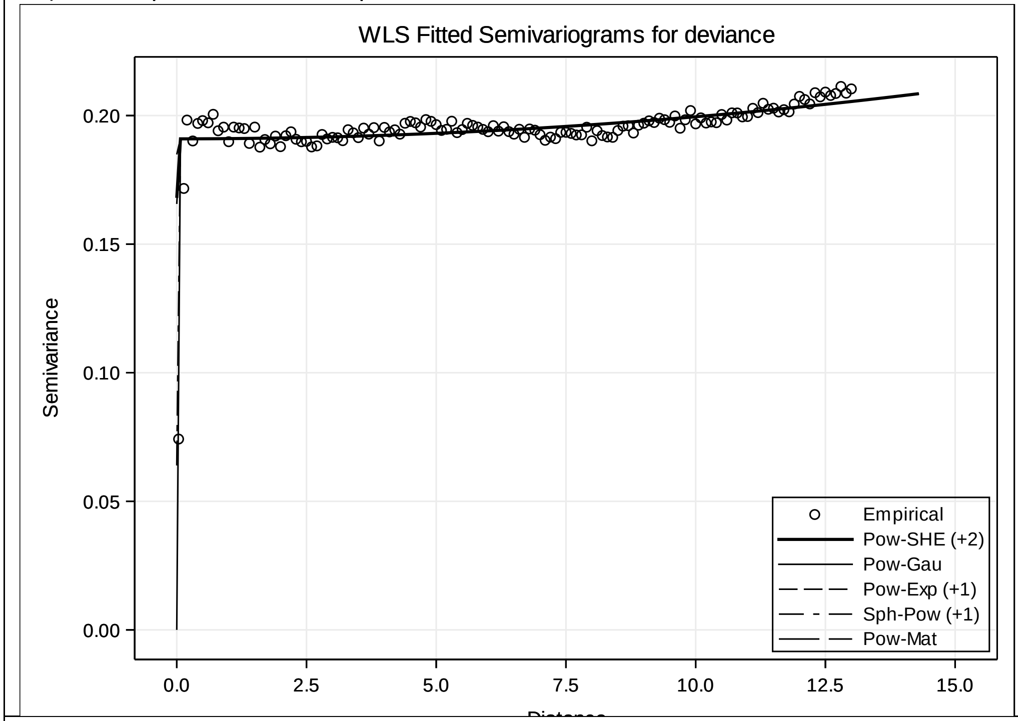

i.2) Team Sports, 3rd order spatial detrend + rater adjustment

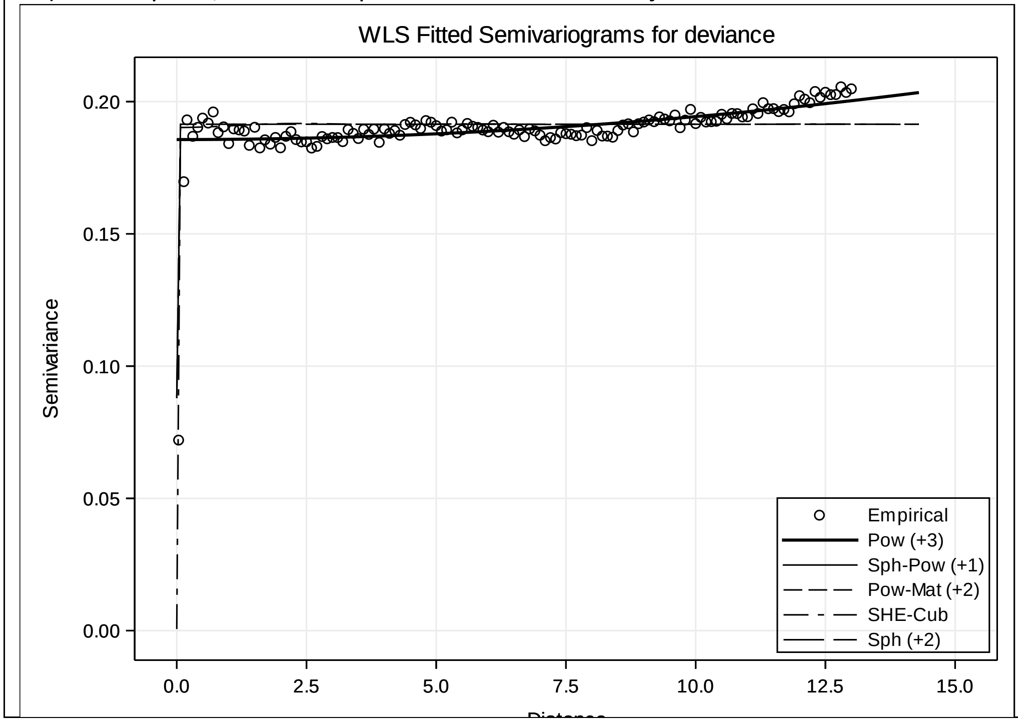

j.1) Yard Decorations, 3rd order spatial detrend

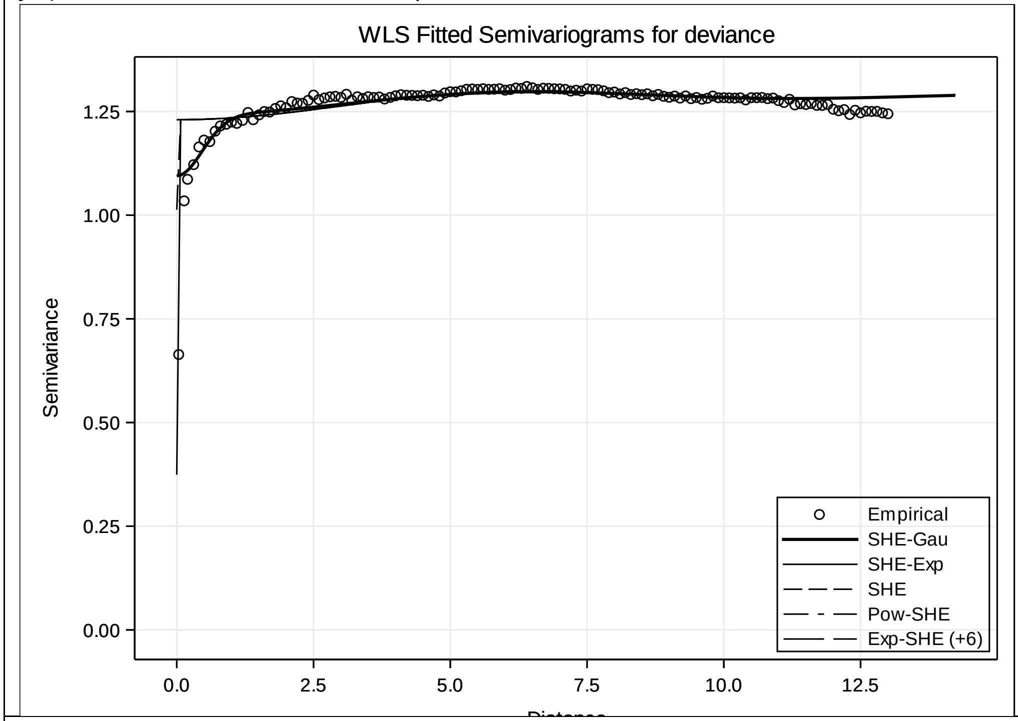

j.2) Yard Decorations, 3rd order spatial detrend + rater adjustment

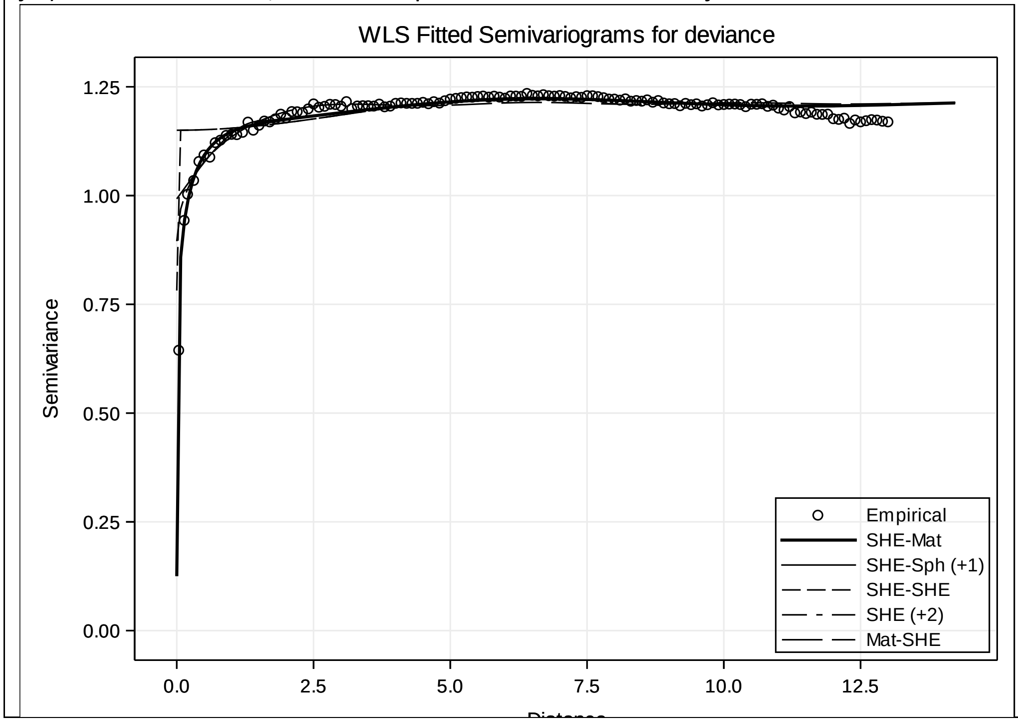

k.1) Fences, 3rd order spatial detrend

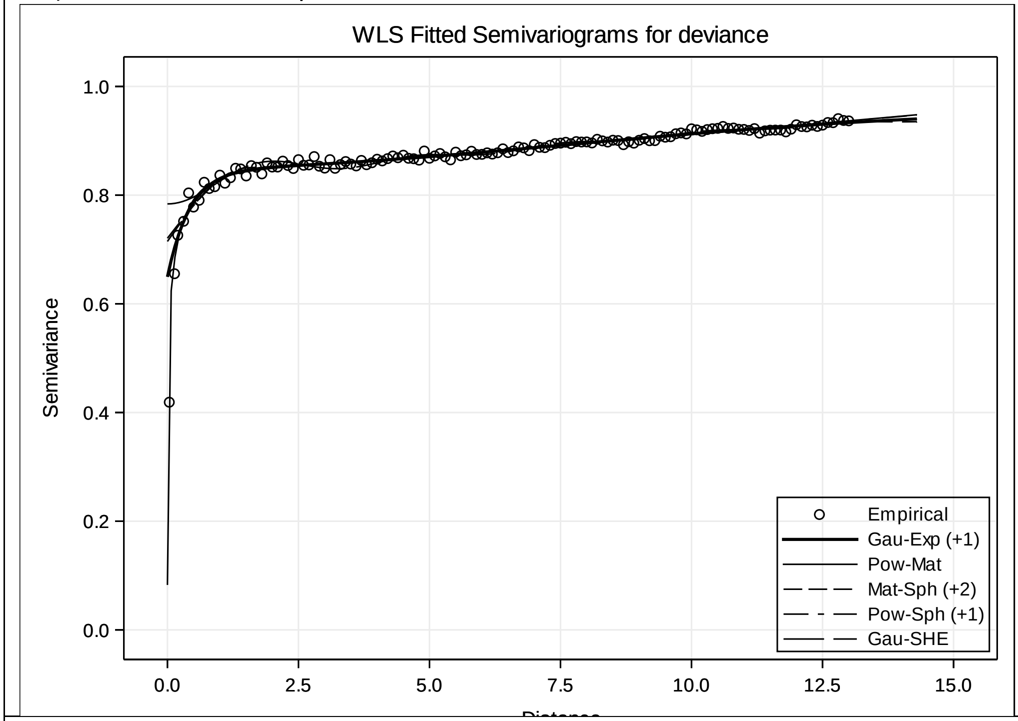

k.2) Fences, 3rd order spatial detrend + rater adjustment

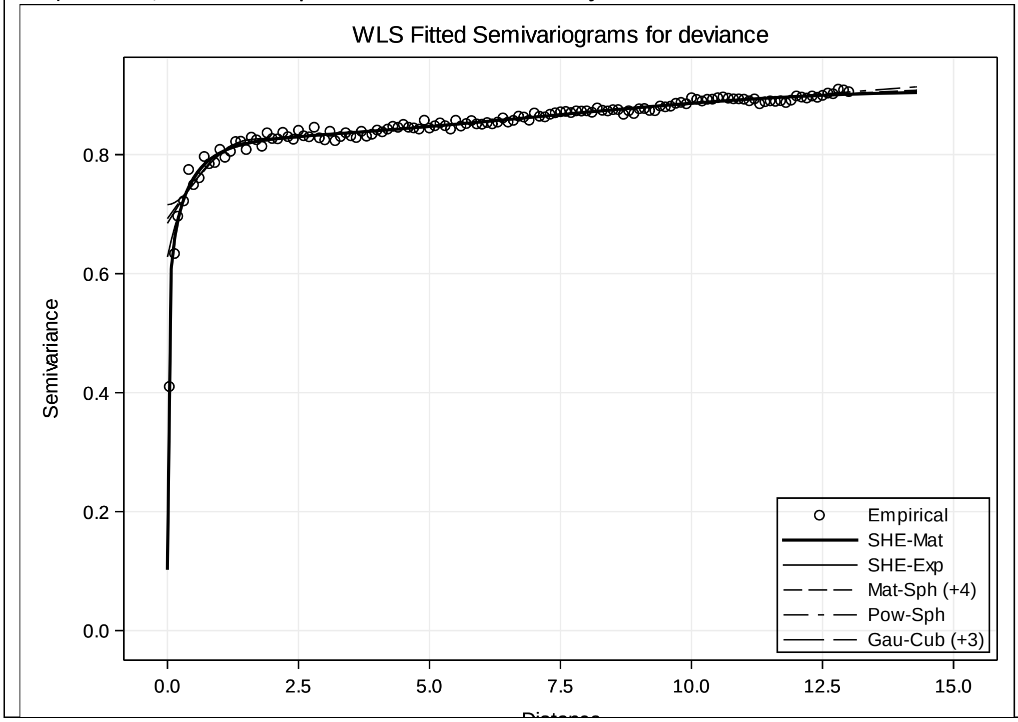

I.1) Sidewalk Present, 3rd order spatial detrend

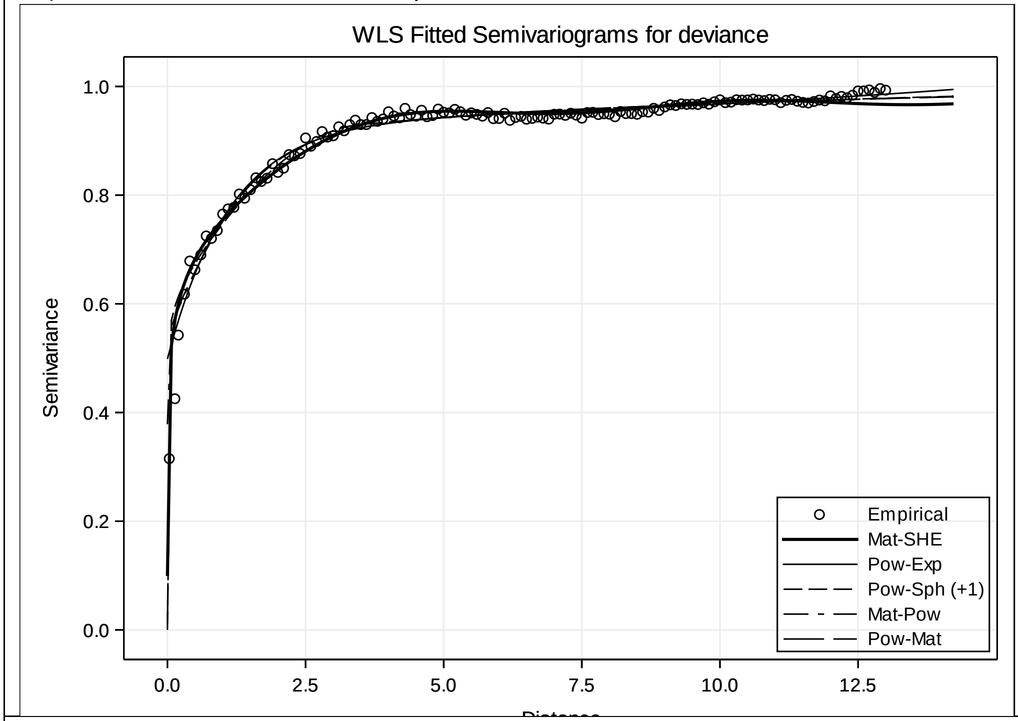

I.2) Sidewalk Present, 3rd order spatial detrend + rater adjustment

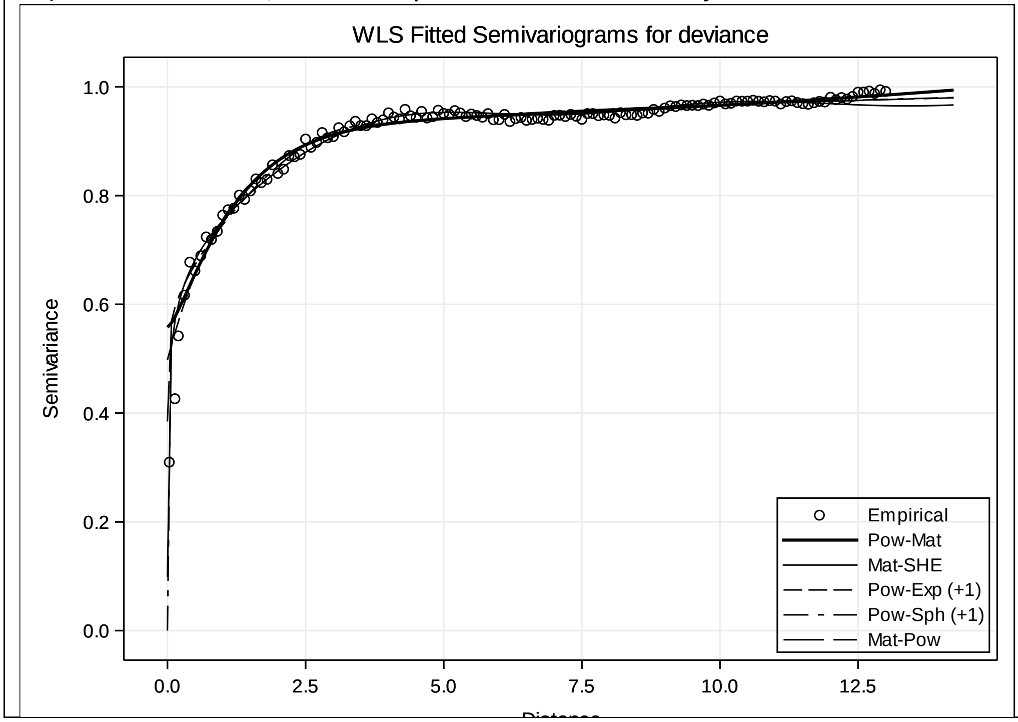

m.1) Sidewalk Complete, 3rd order spatial detrend

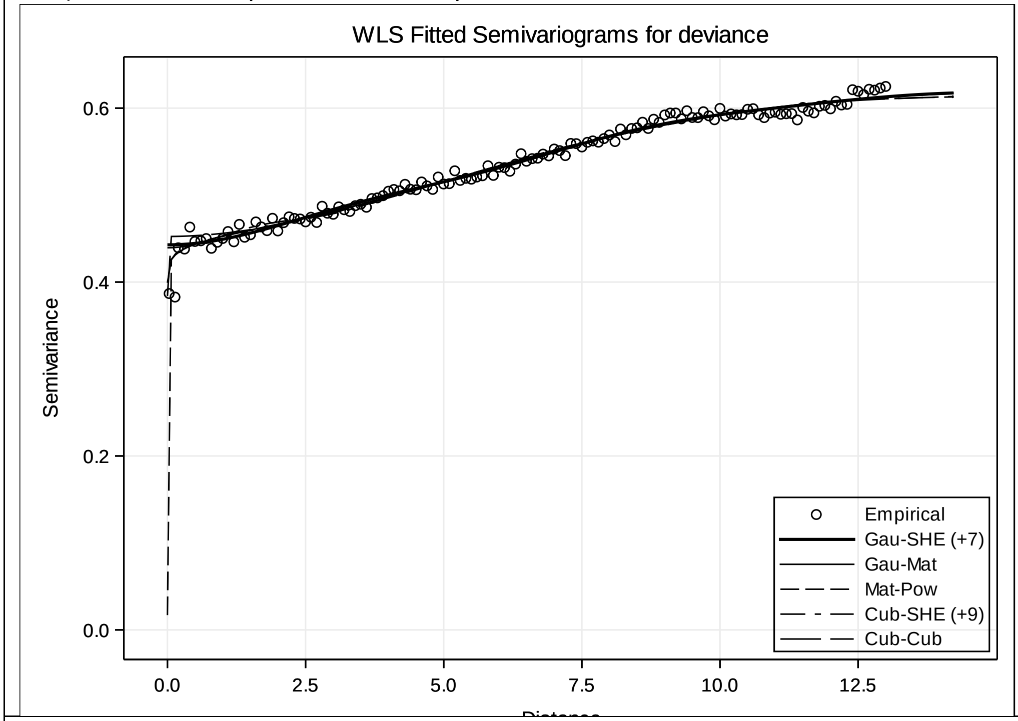

m.2) Sidewalk Complete, 3rd order spatial detrend + rater adjustment

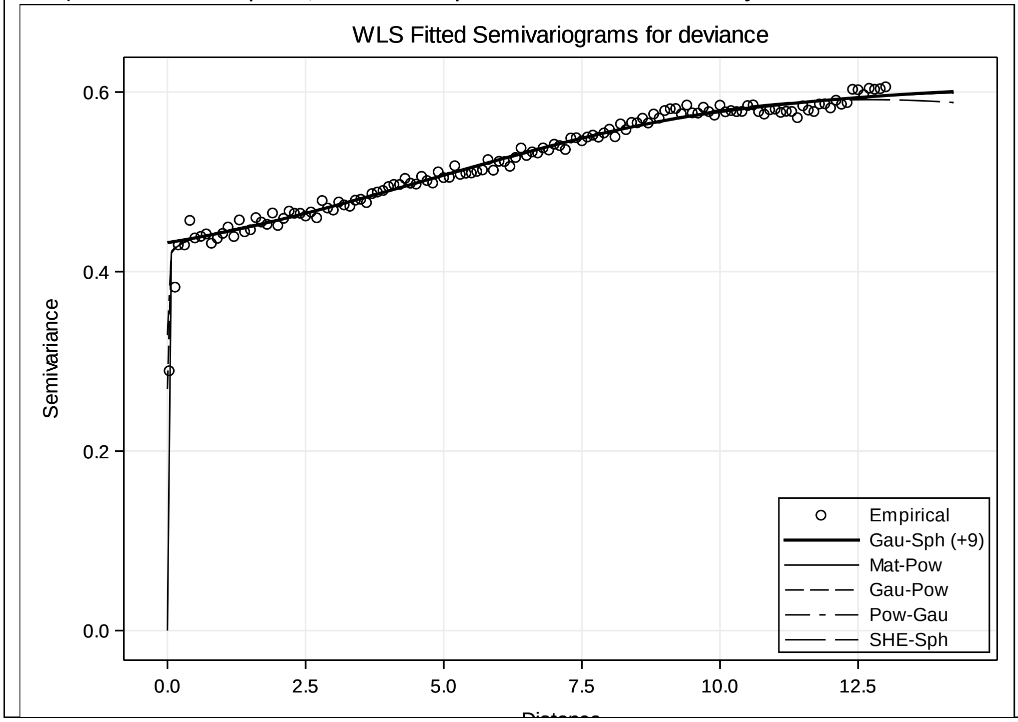

n.1) Sidewalk Condition, 3rd order spatial detrend

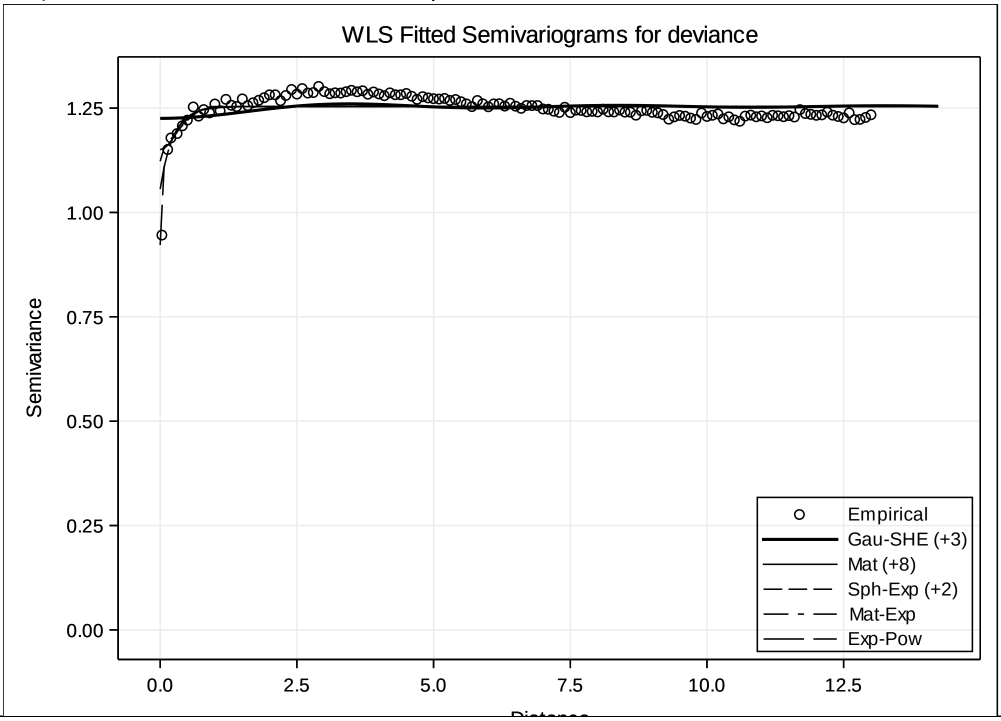

n.2) Sidewalk Condition, 3rd order spatial detrend + rater adjustment

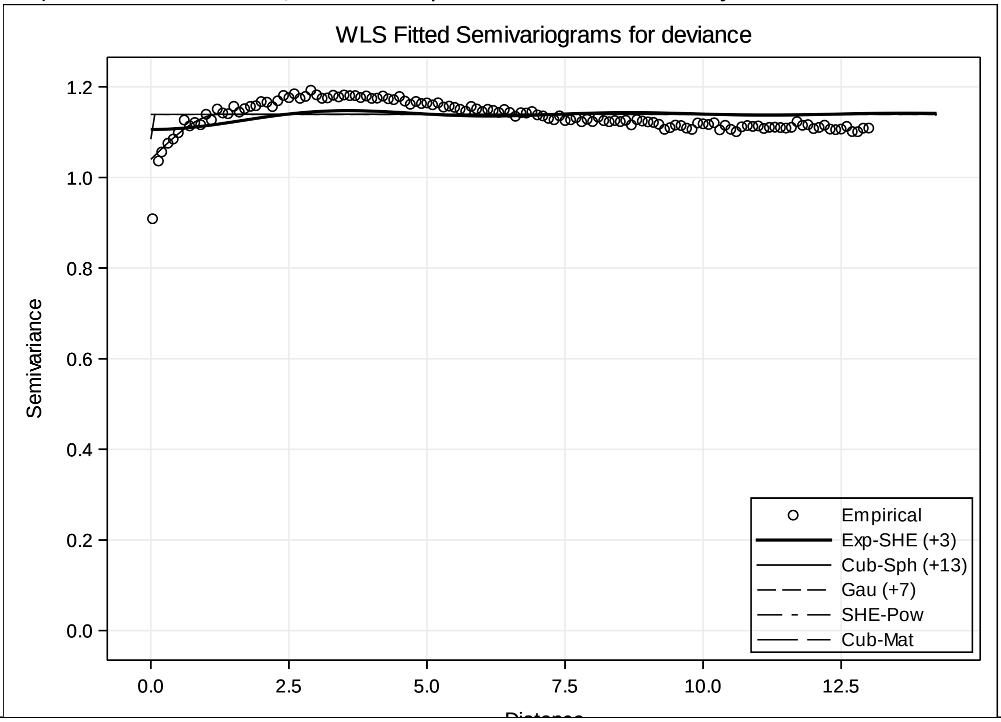

o.1) Sidewalk Width, 3rd order spatial detrend

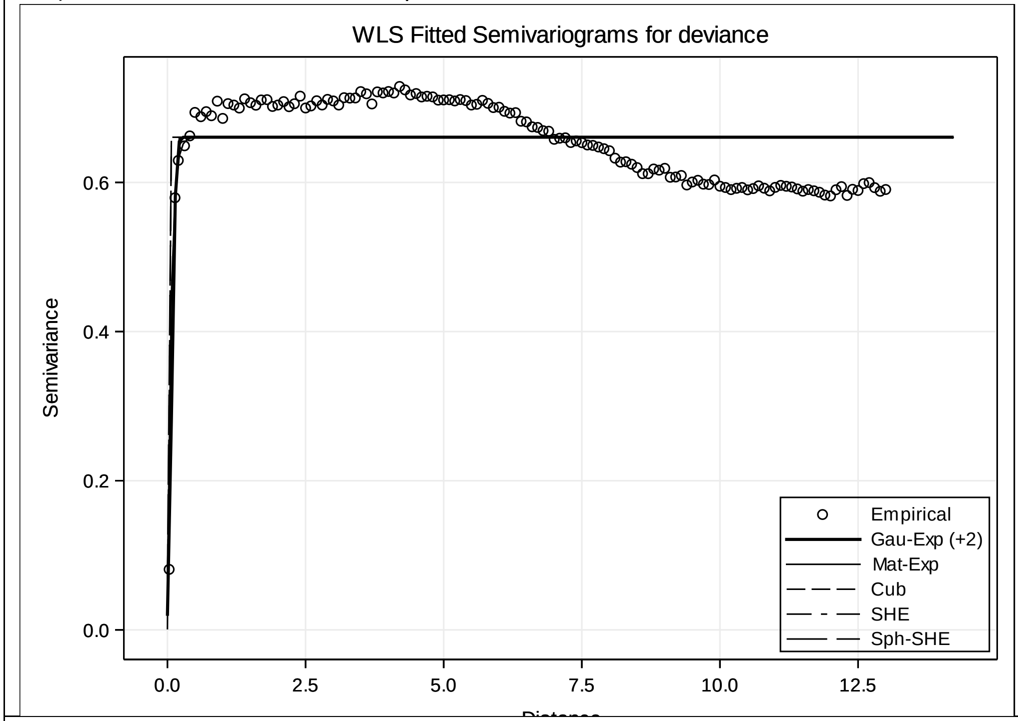

o.2) Sidewalk Width, 3rd order spatial detrend + rater adjustment

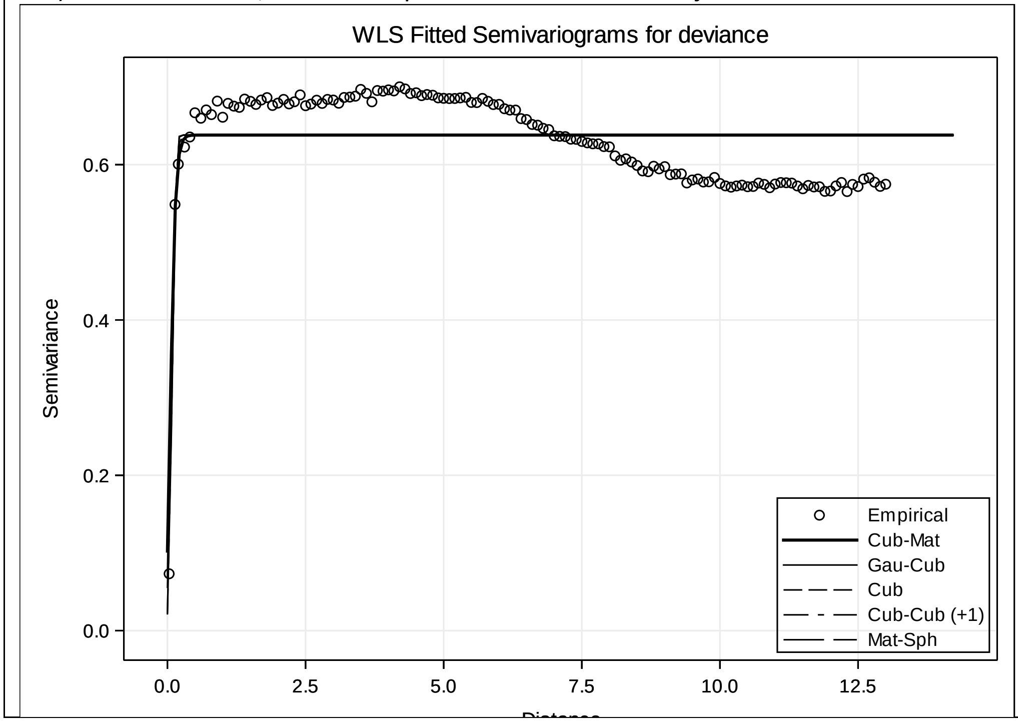

p.1) Sidewalk to Curb Distance, 3rd order spatial detrend

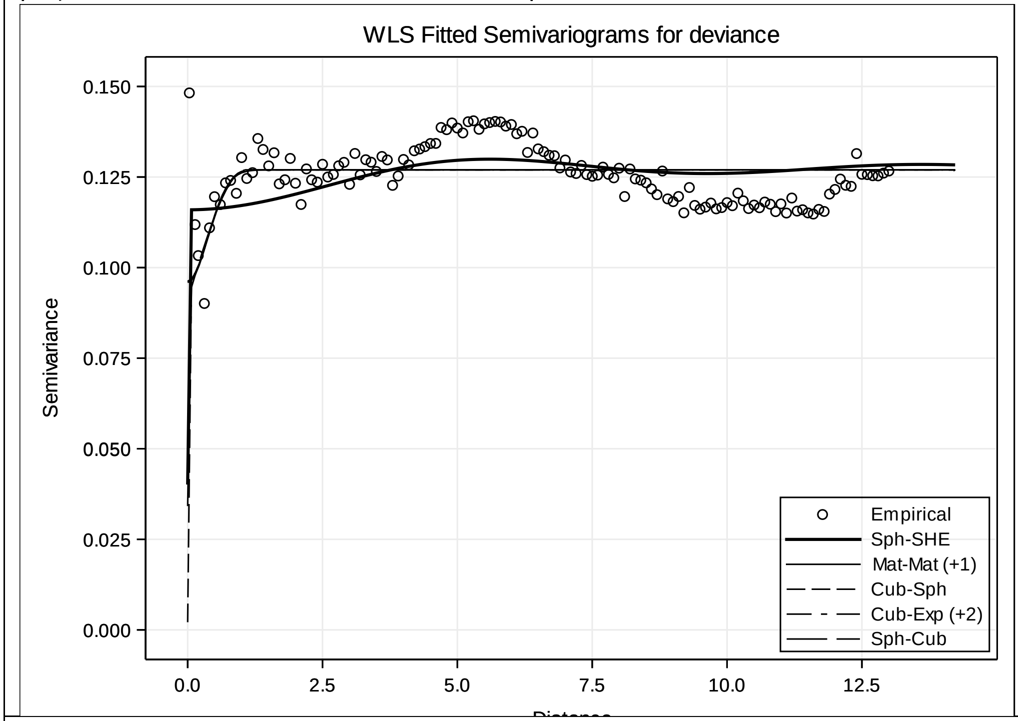

p.2) Sidewalk to Curb Distance, 3rd order spatial detrend + rater adjustment

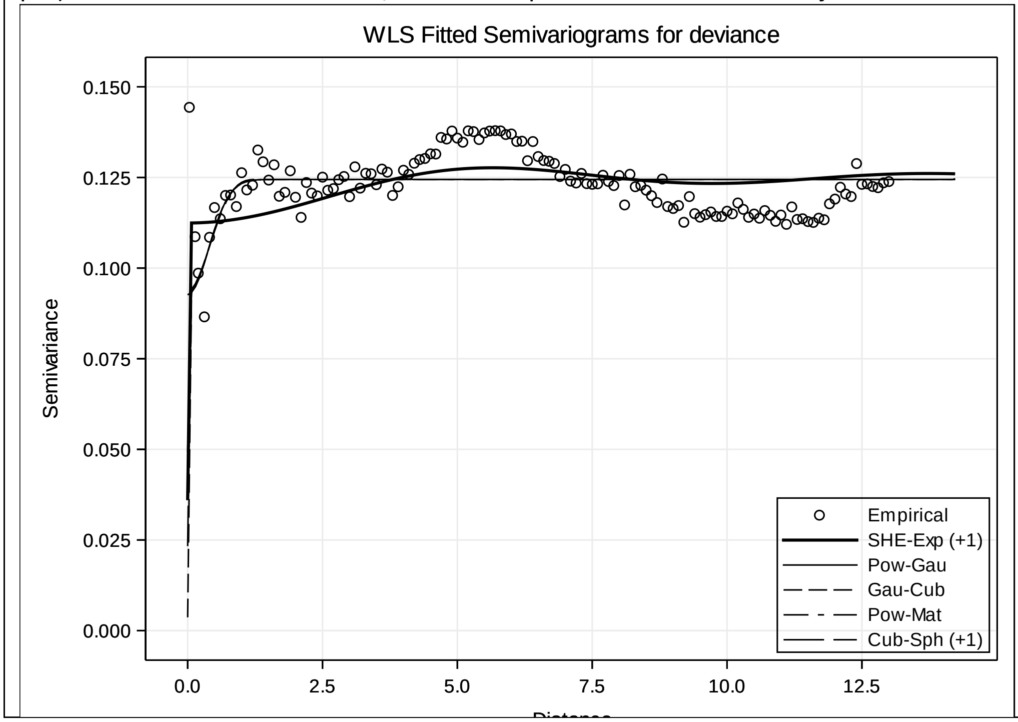

q.1) Car Obstruction, 3rd order spatial detrend

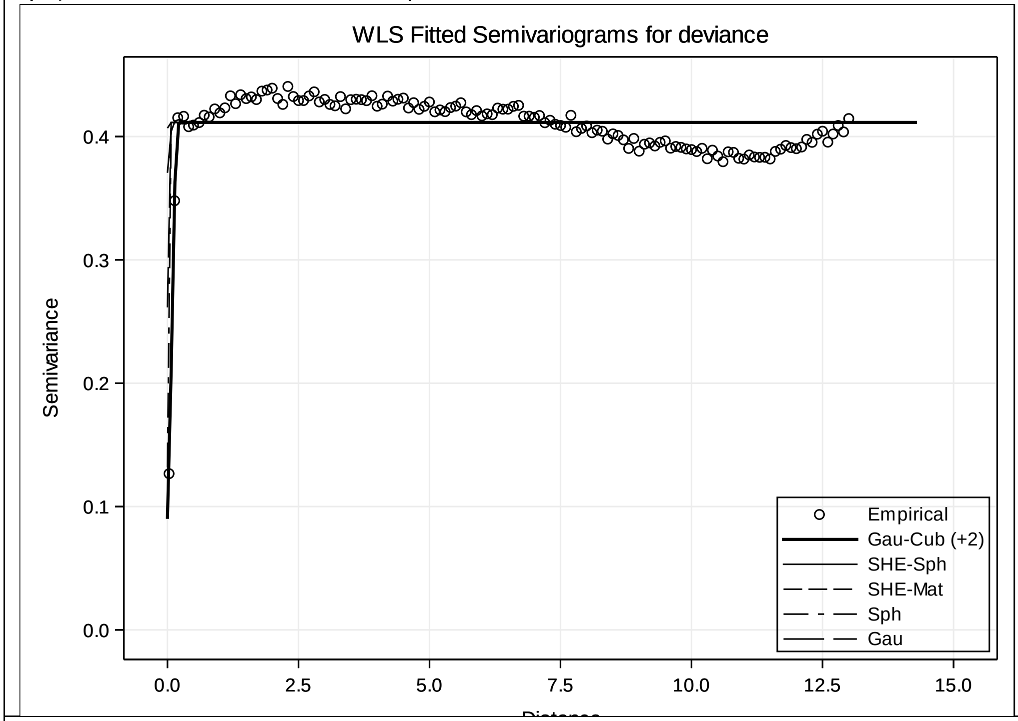

q.2) Car Obstruction, 3rd order spatial detrend + rater adjustment

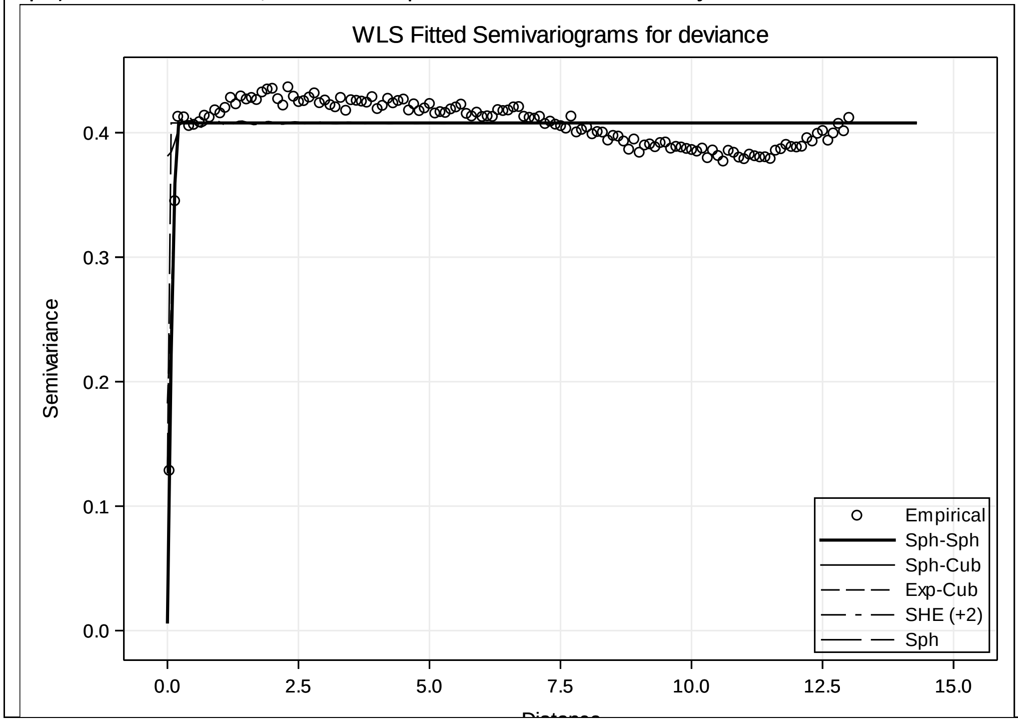

r.1) Garbage Can Obstruction, 3rd order spatial detrend

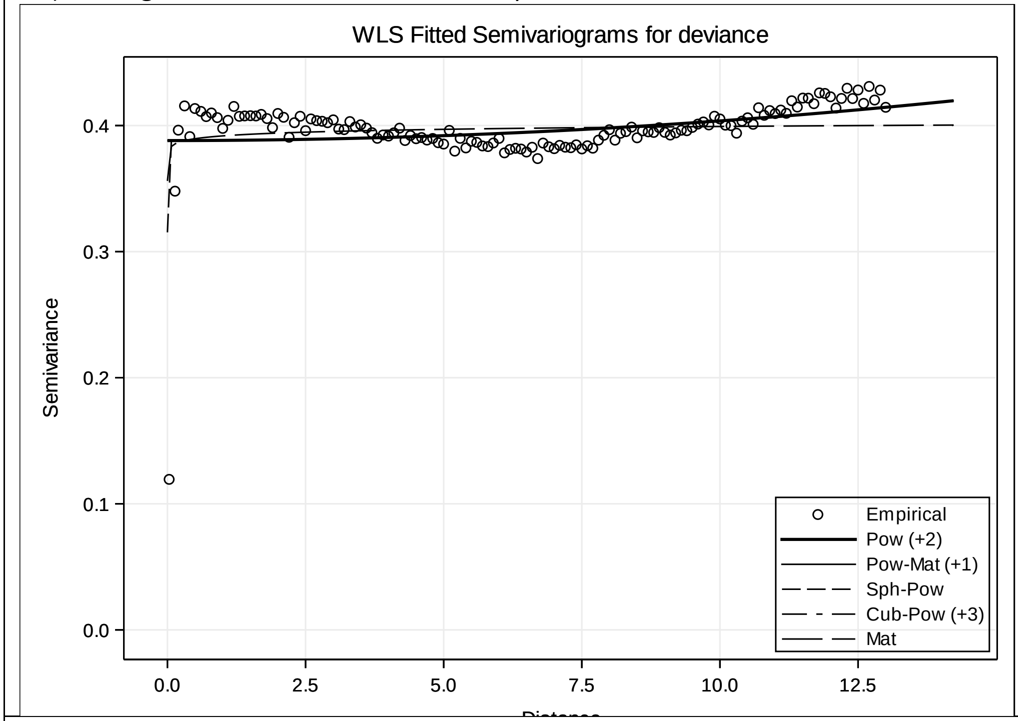

r.2) Garbage Can Obstruction, 3rd order spatial detrend + rater adjustment

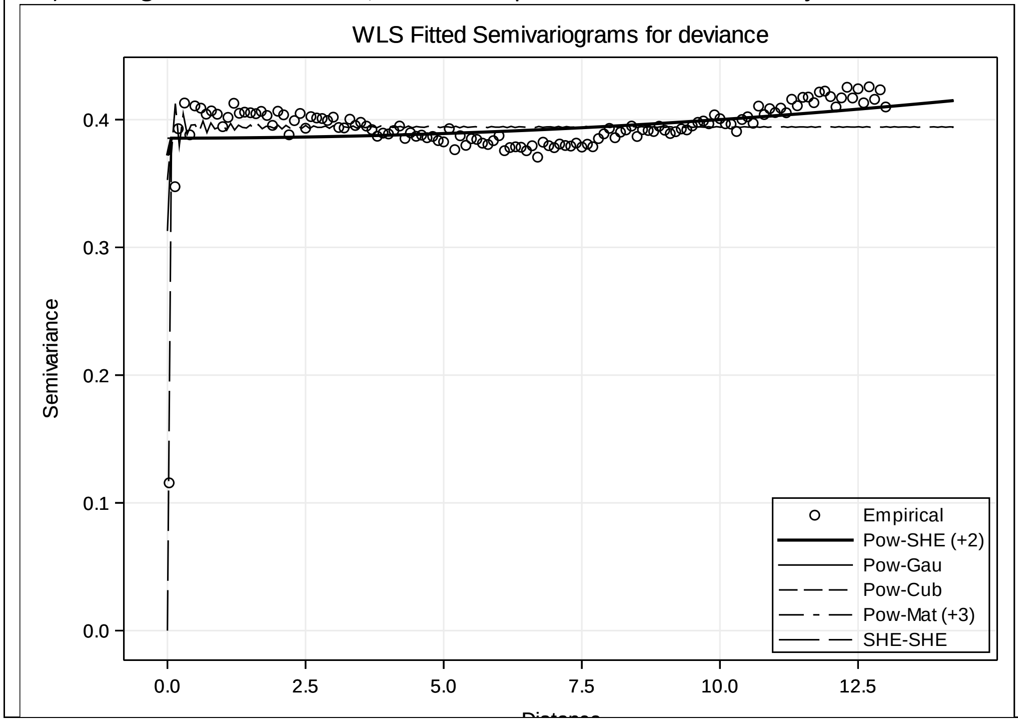

s.1) Pole/Sign Obstruction, 3rd order spatial detrend

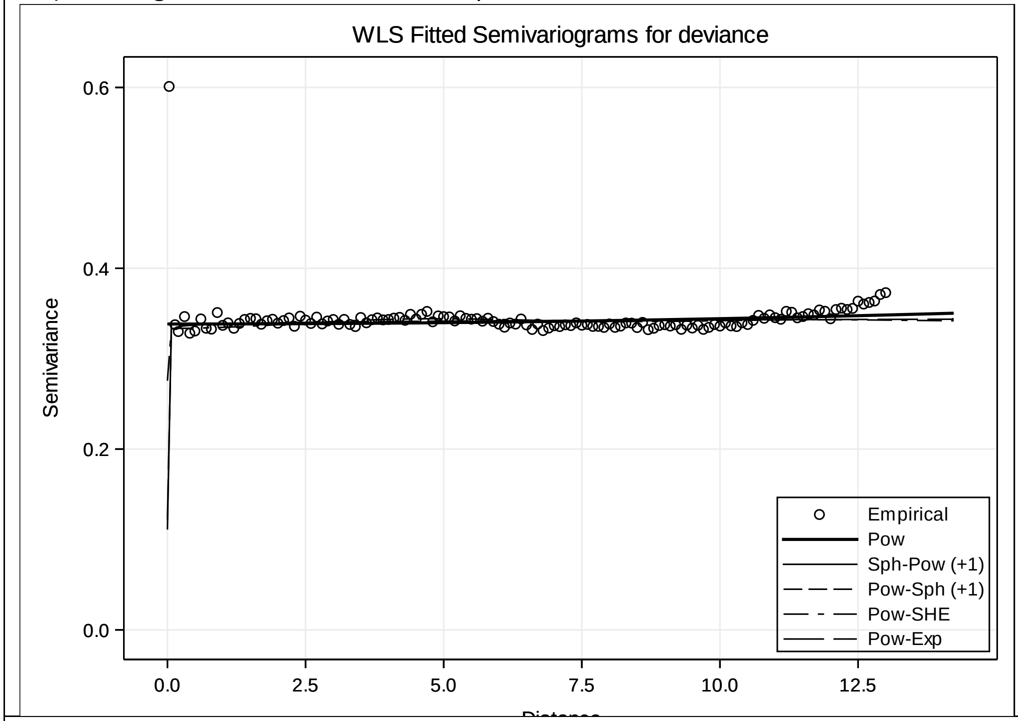

s.2) Pole/Sign Obstruction, 3rd order spatial detrend + rater adjustment

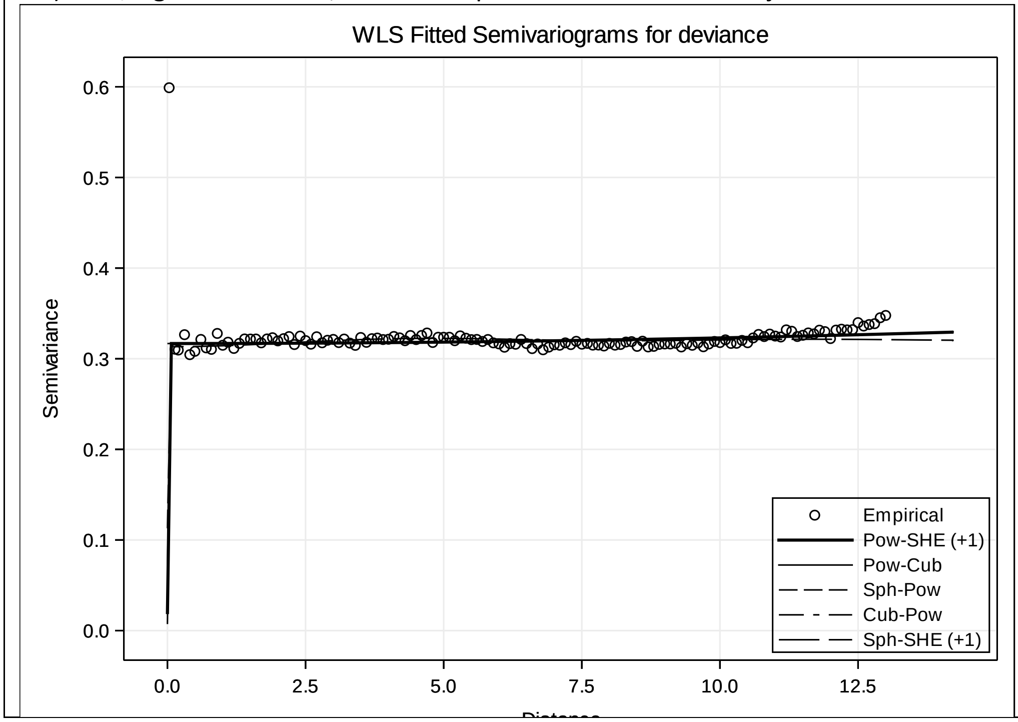

t.1) Other Obstruction, 3rd order spatial detrend

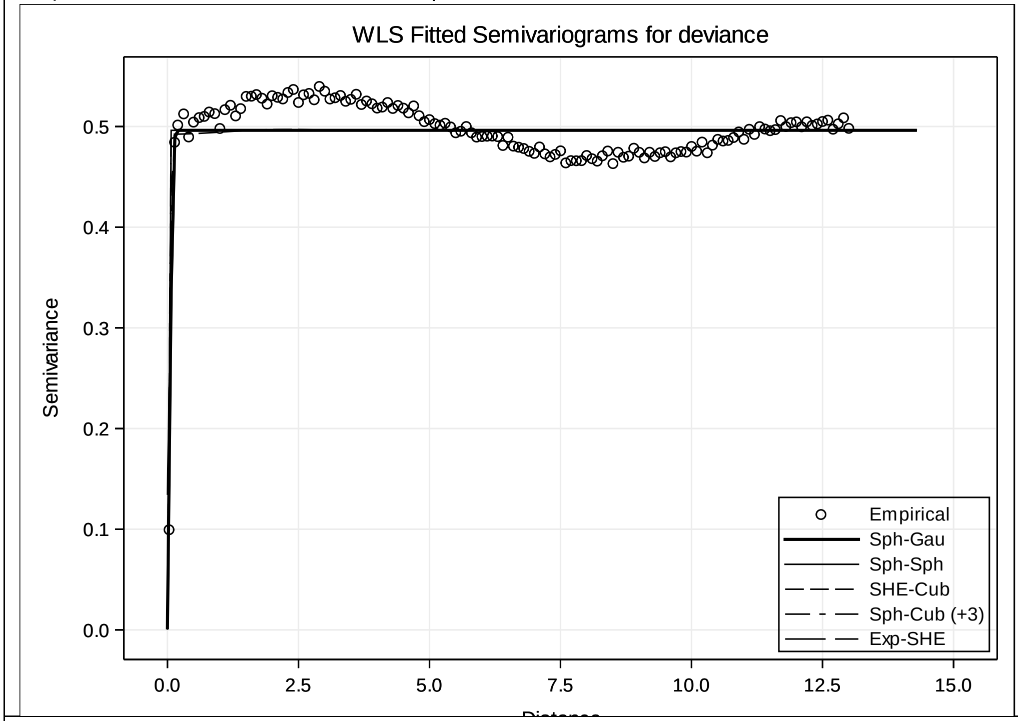

t.2) Other Obstruction, 3rd order spatial detrend + rater adjustment

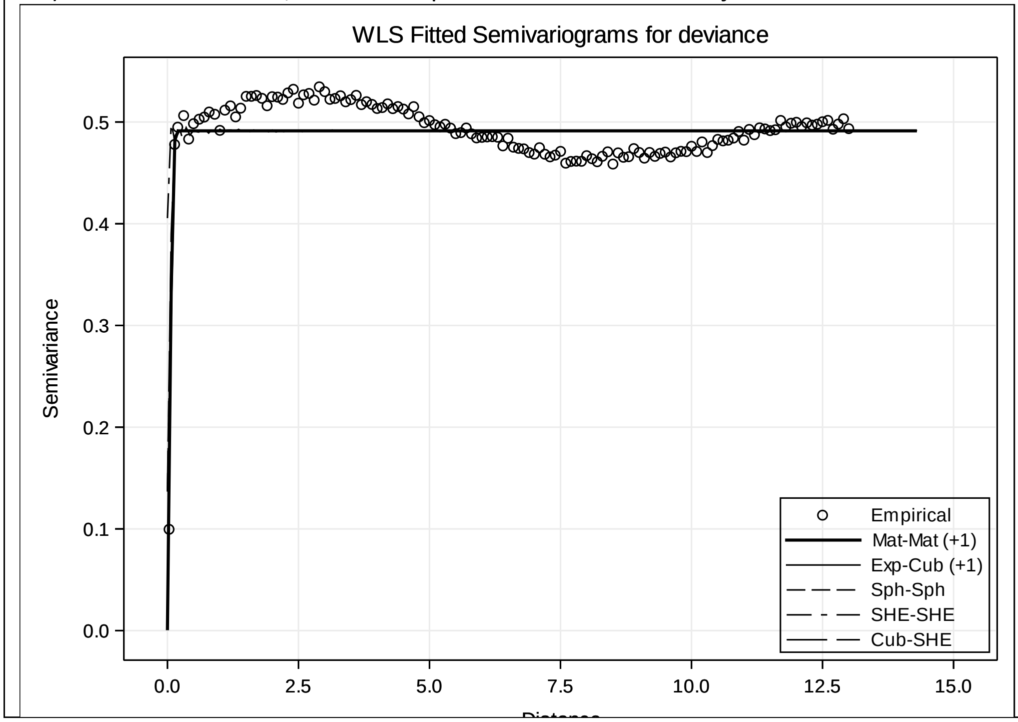

u.1) Curb Cuts, 3rd order spatial detrend

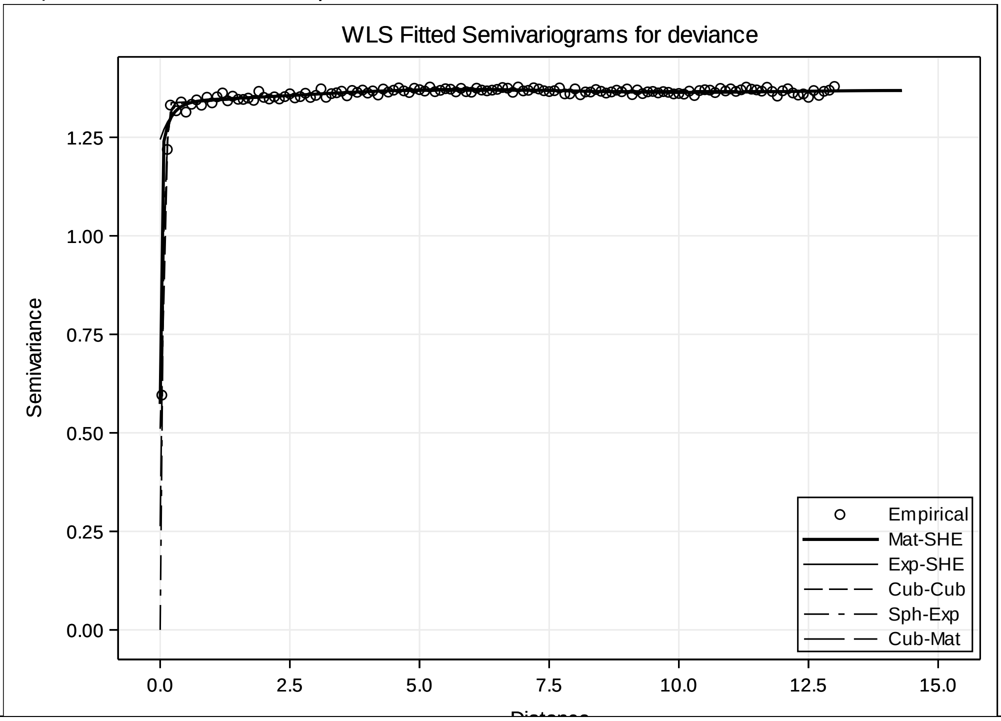

u.2) Curb Cuts, 3rd order spatial detrend + rater adjustment

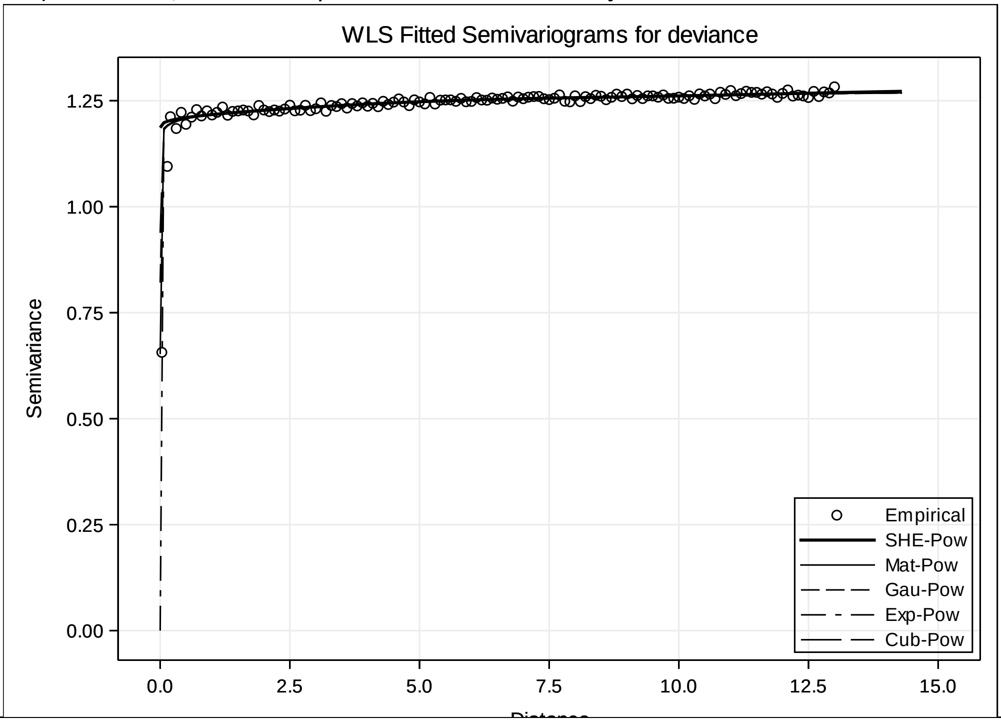

v.1) Clear Intersection, 3rd order spatial detrend

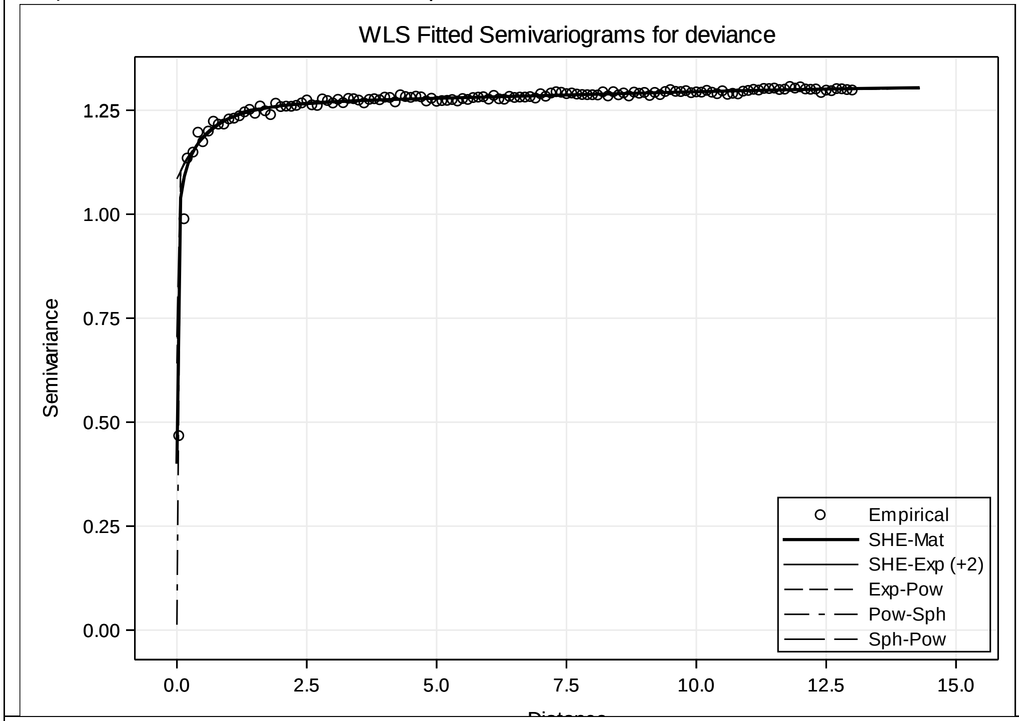

v.2) Clear Intersection, 3rd order spatial detrend + rater adjustment

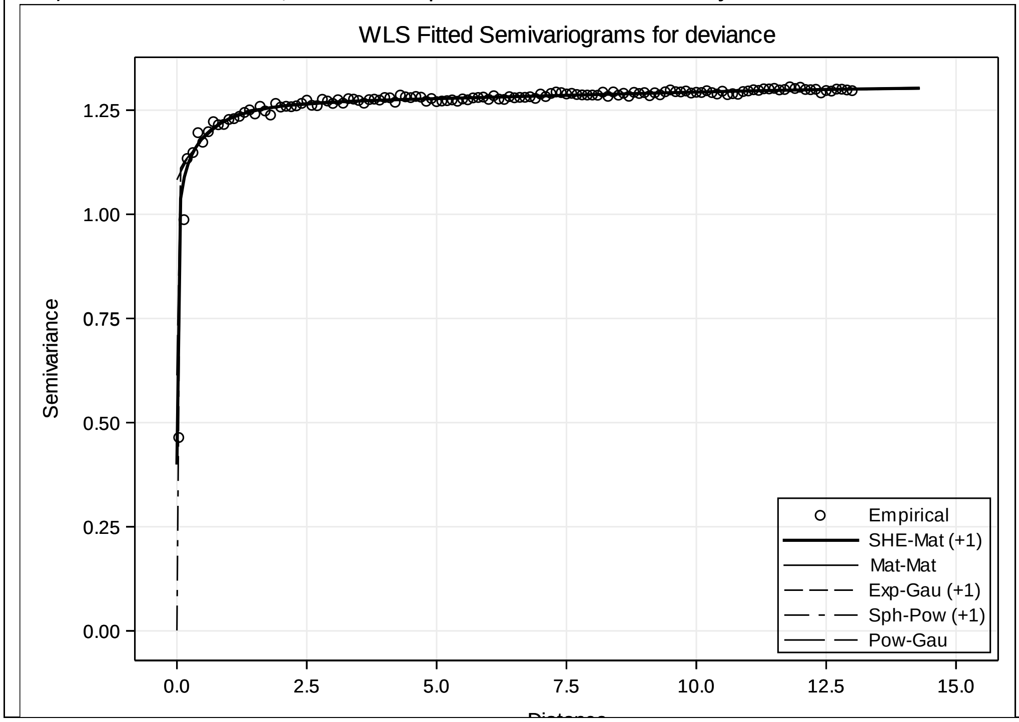

w.1) Pedestrian Crossing Sign, 3rd order spatial detrend

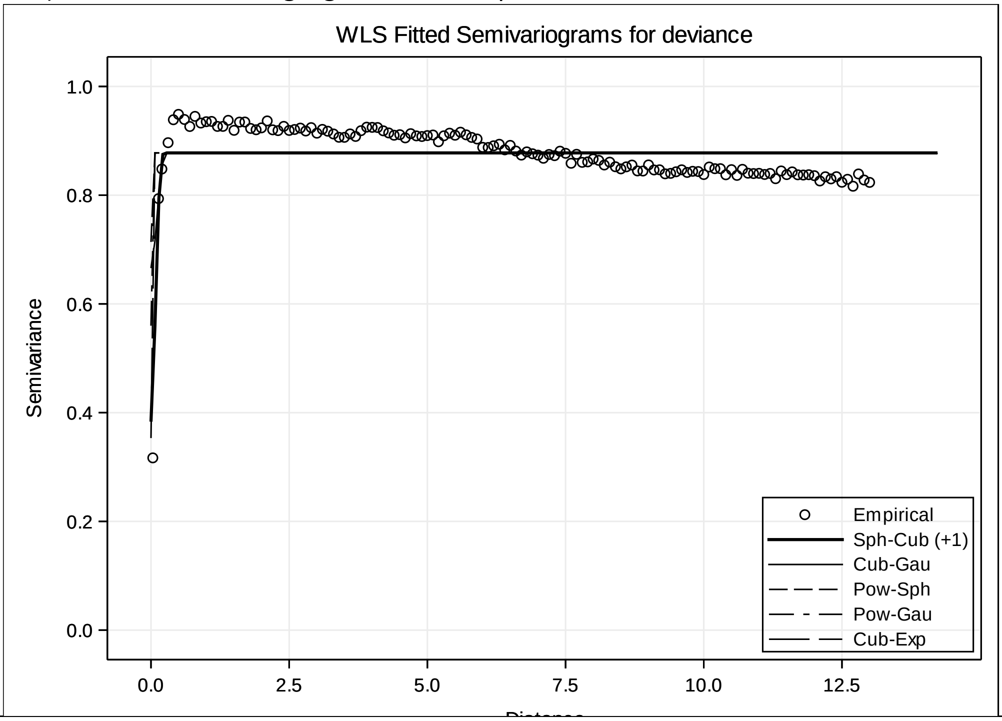

w.2) Pedestrian Crossing Sign, 3rd order spatial detrend + rater adjustment

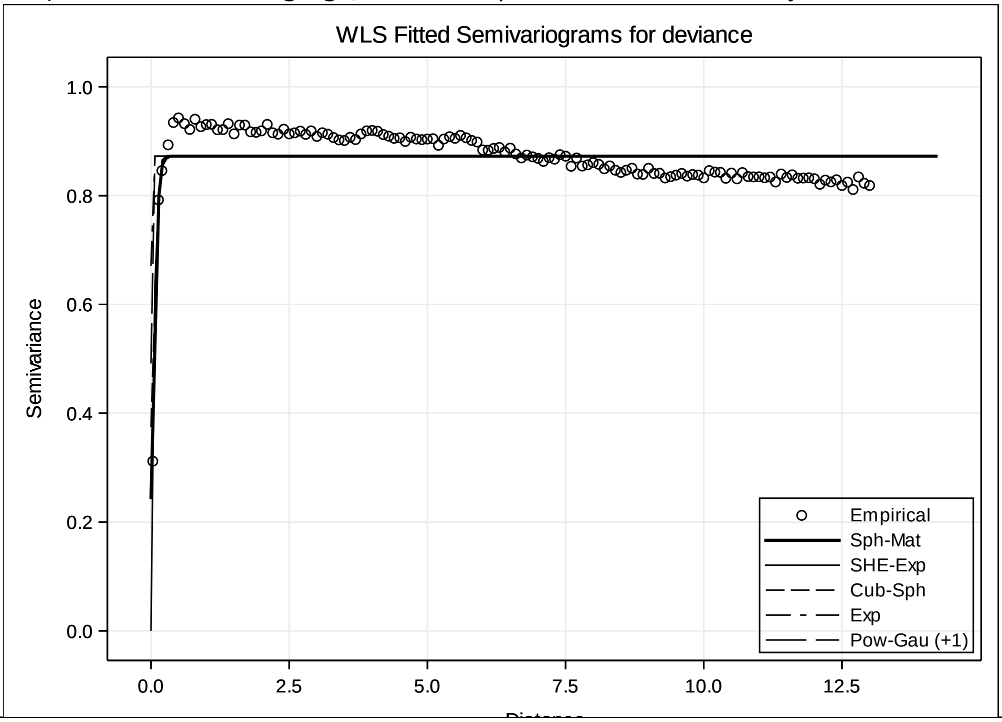

x.1) Pedestrian Signal, 3rd order spatial detrend

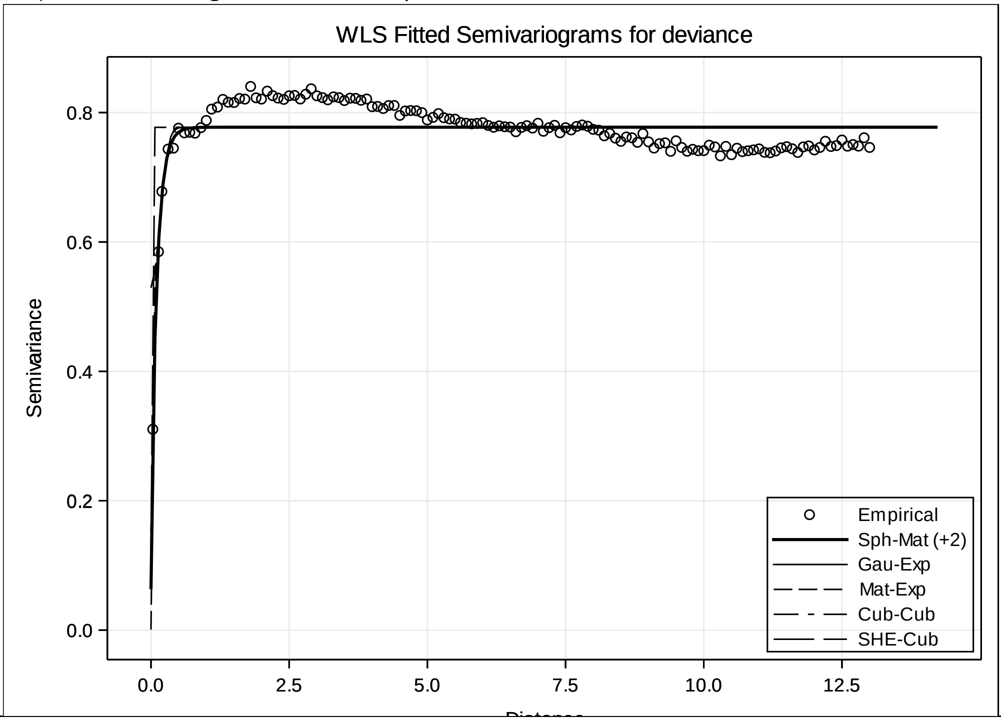

x.2) Pedestrian Signal, 3rd order spatial detrend + rater adjustment

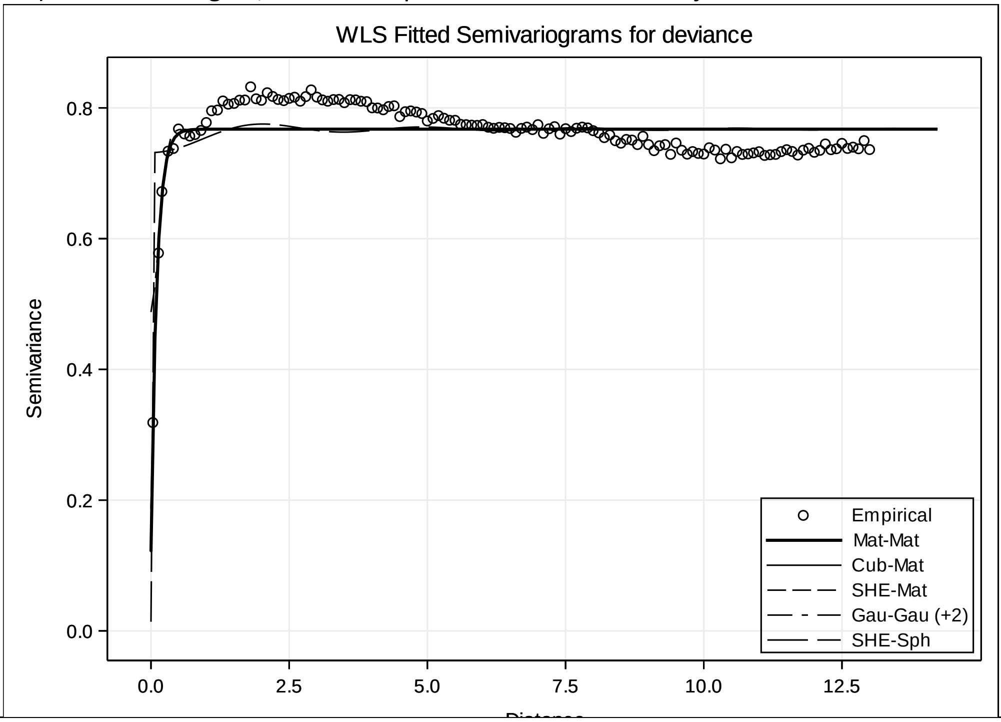

y.1) Pedestrian Crossing Marks, 3rd order spatial detrend

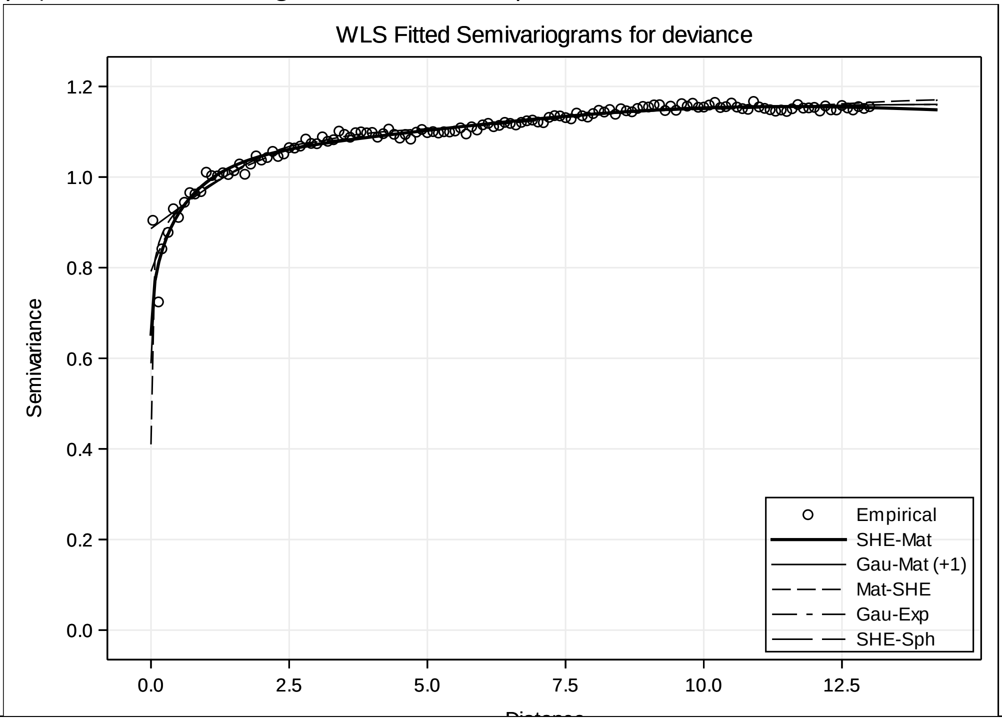

y.2) Pedestrian Crossing Marks, 3rd order spatial detrend + rater adjustment

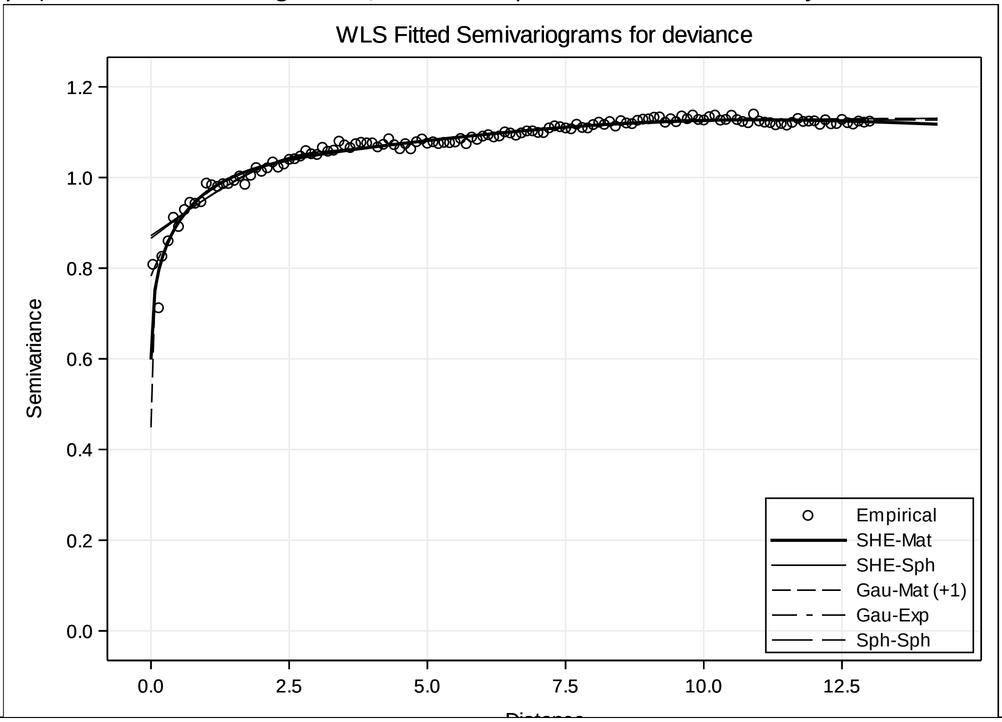

z.1) Type of Pedestrian Crosswalk, 3rd order spatial detrend

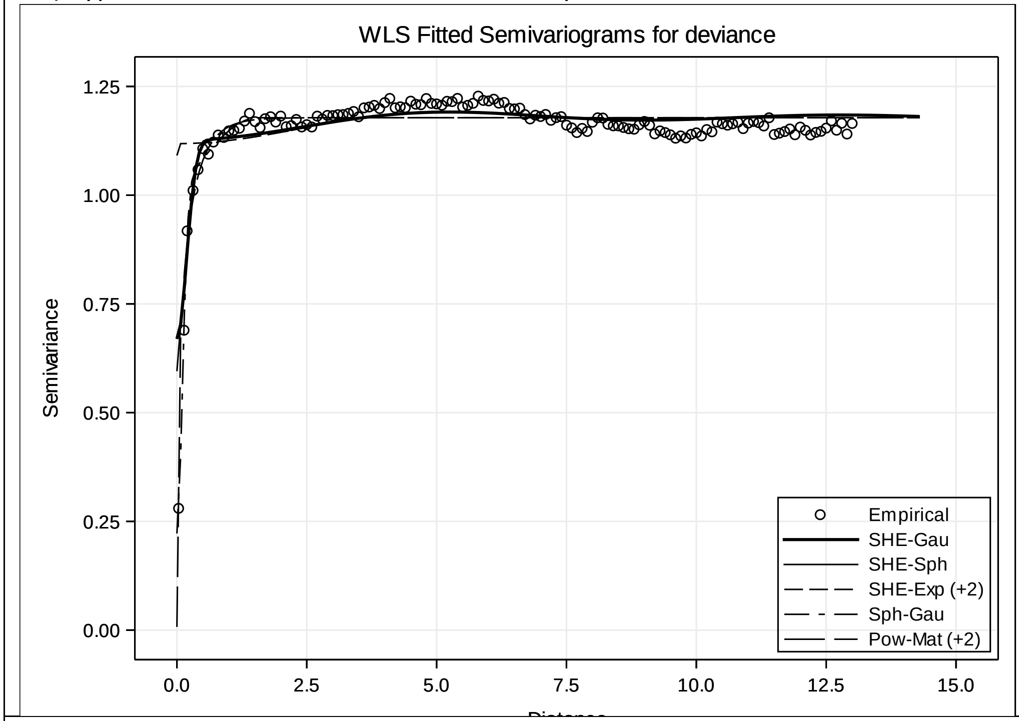

z.2) Type of Pedestrian Crosswalk, 3rd order spatial detrend + rater adjustment

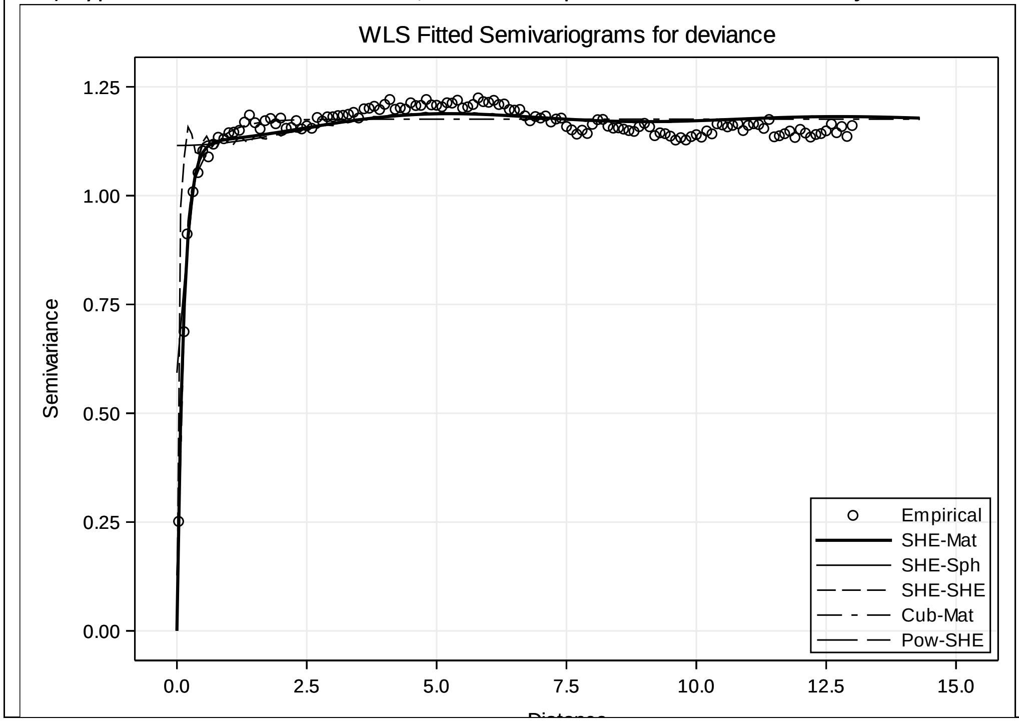

aa.1) Traffic Signal Type, 3rd order spatial detrend

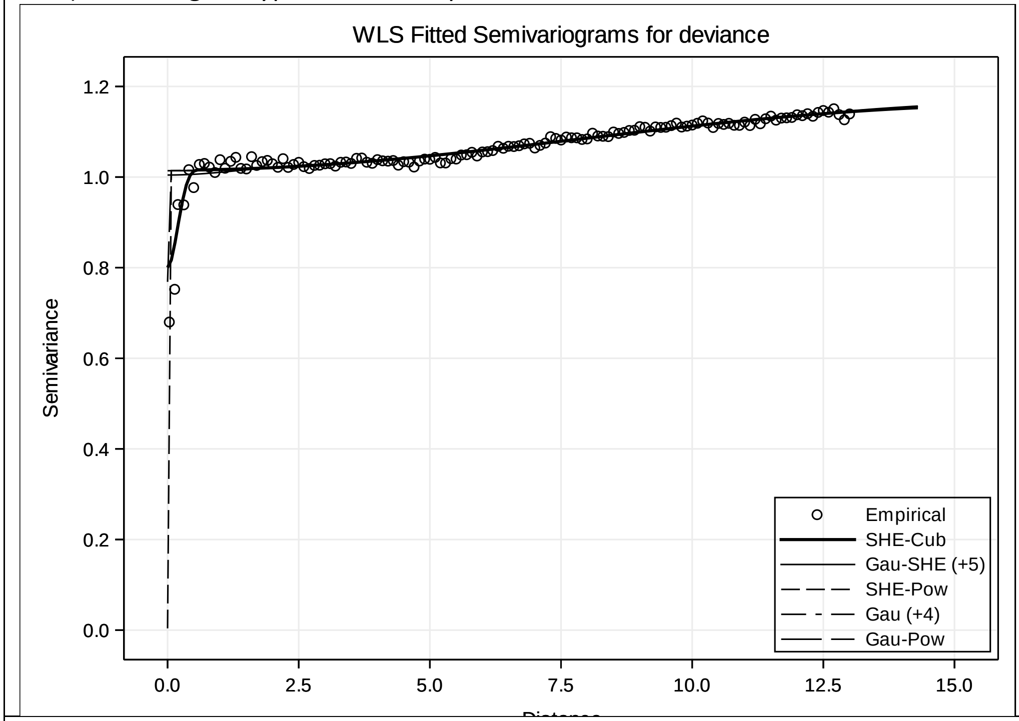

aa.2) Traffic Signal Type, 3rd order spatial detrend + rater adjustment

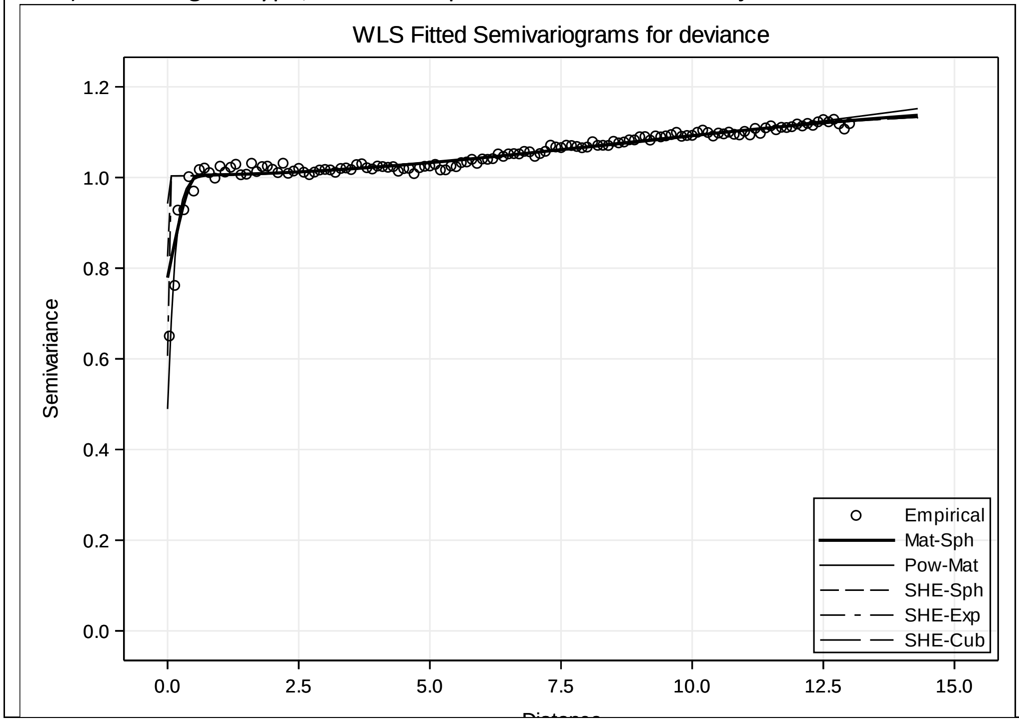

ab.1) One-way Street, 3rd order spatial detrend

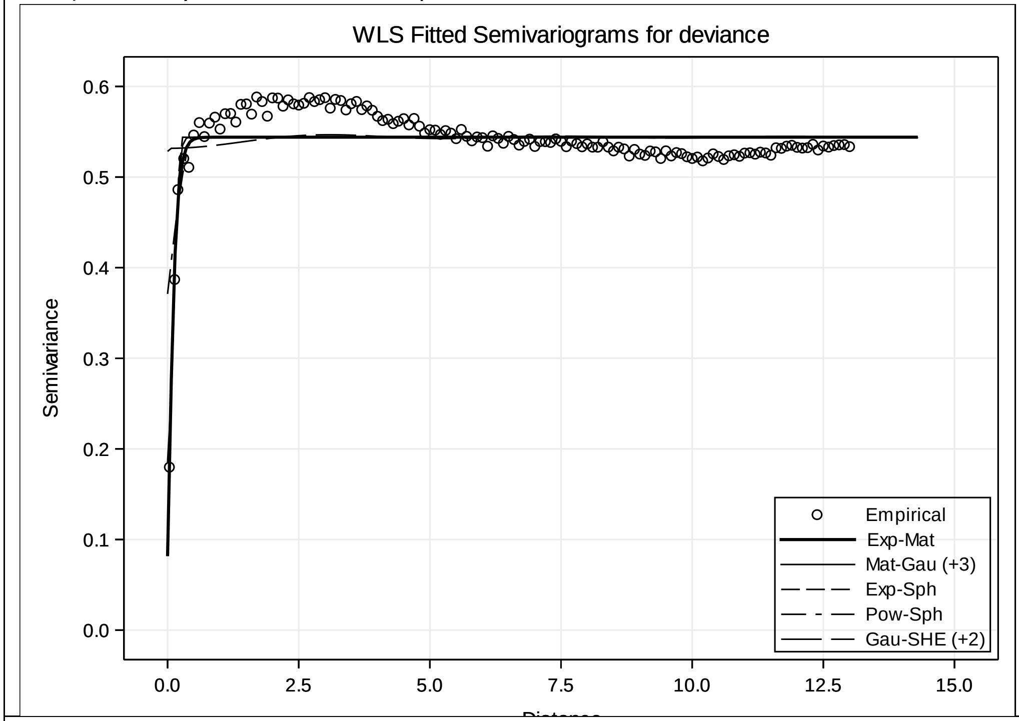

ab.2) One-way Street, 3rd order spatial detrend + rater adjustment

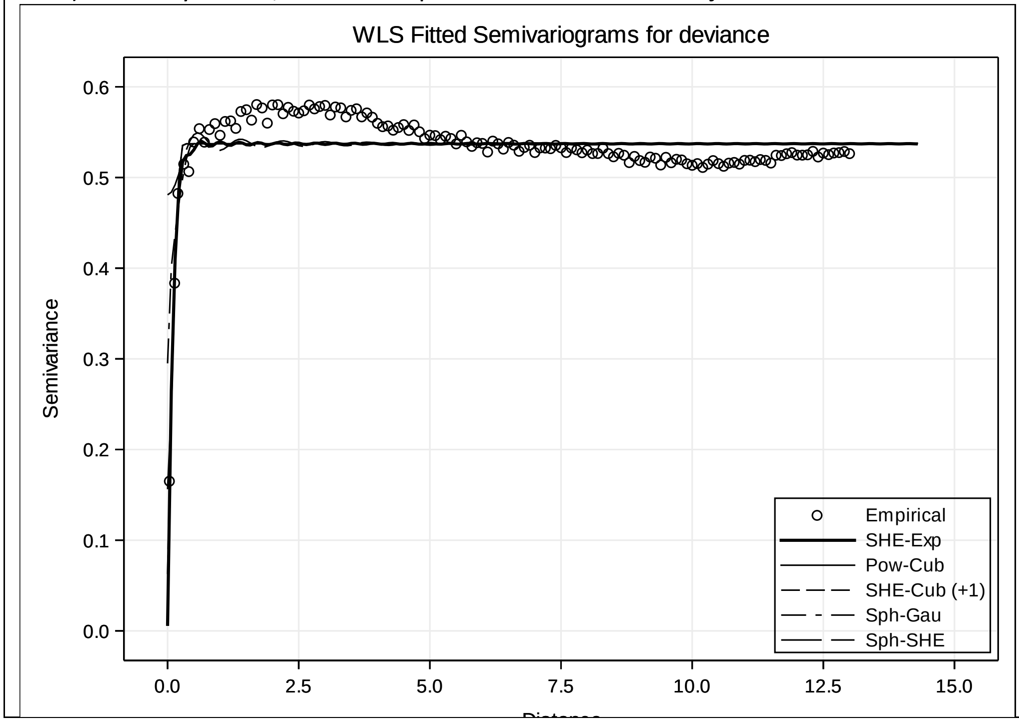

ac.1) Number of Lanes, 3rd order spatial detrend

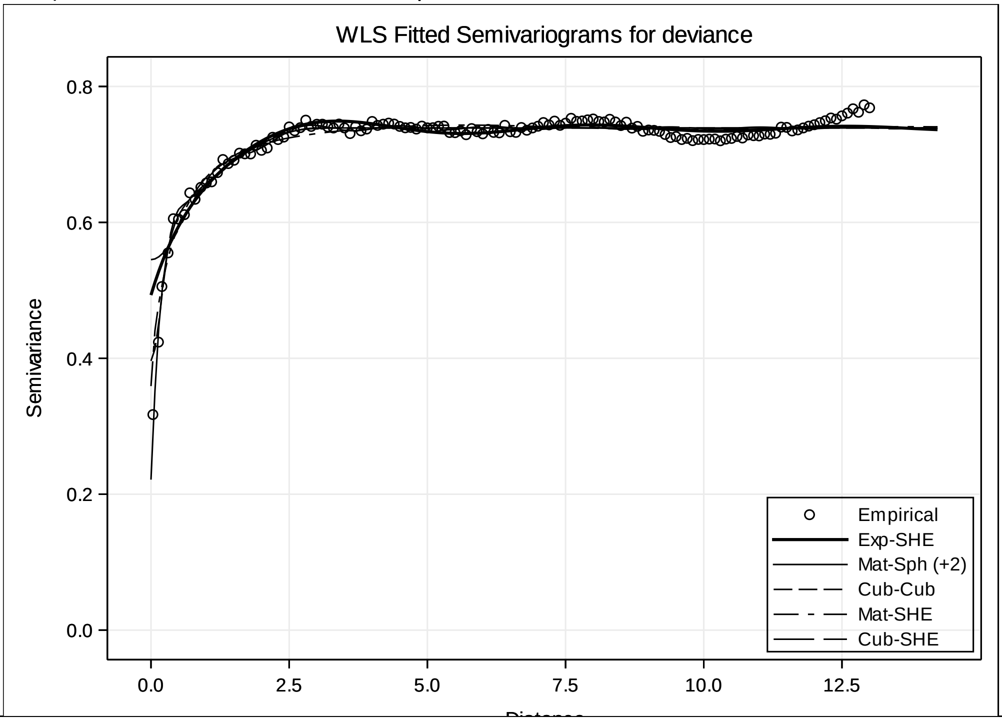

ac.2) Number of Lanes, 3rd order spatial detrend + rater adjustment

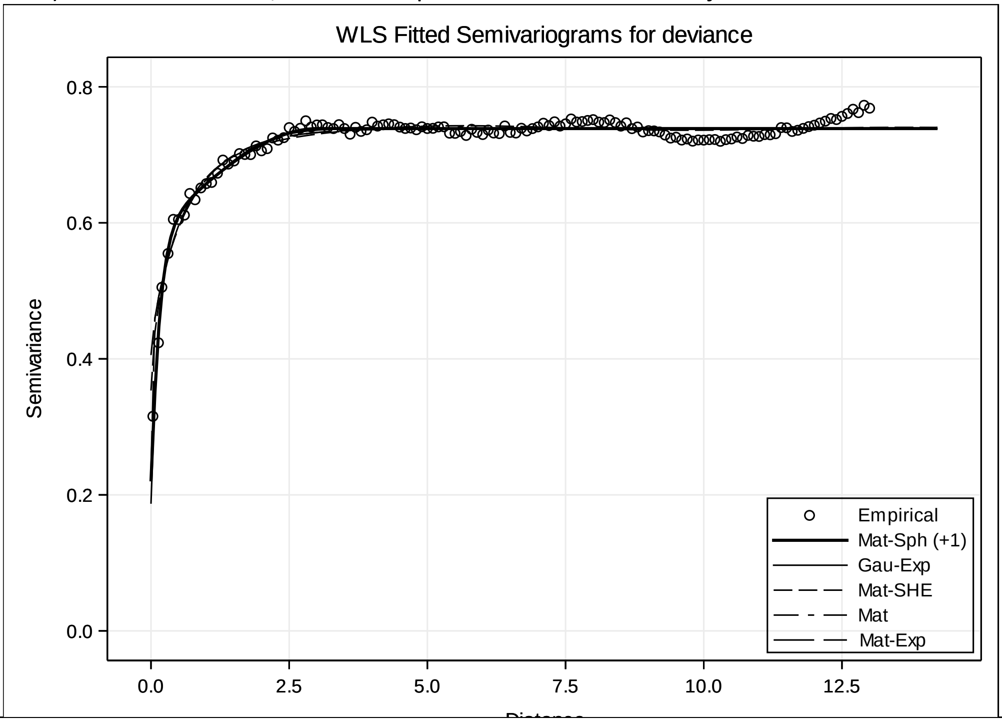

ad.1) Presence of Highway, 3rd order spatial detrend

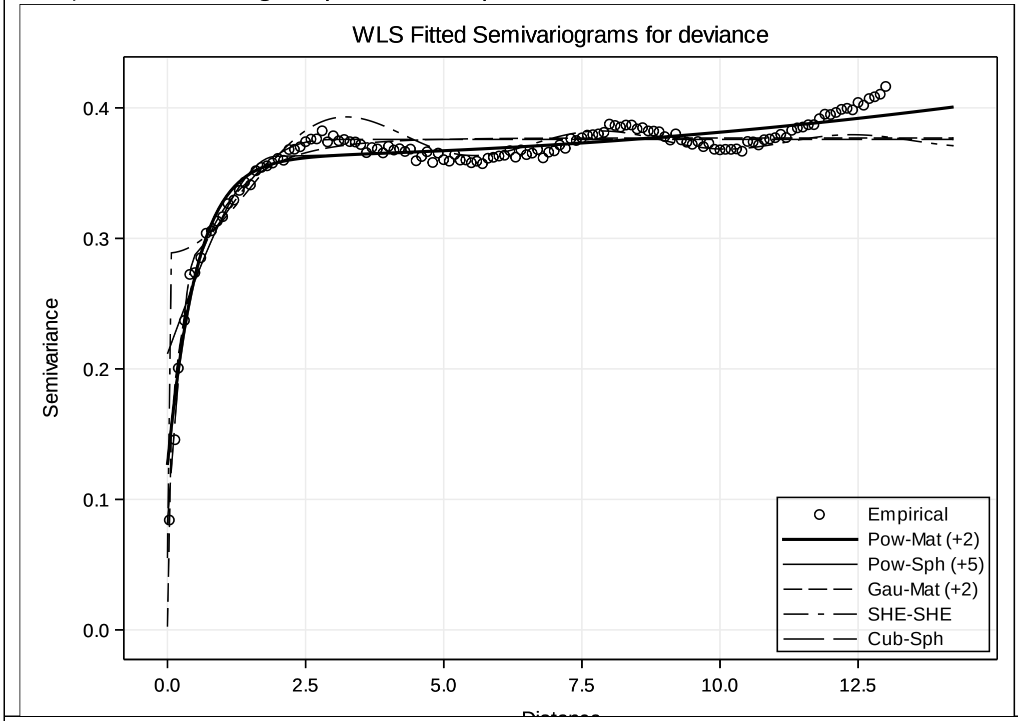

ad.2) Presence of Highway, 3rd order spatial detrend + rater adjustment

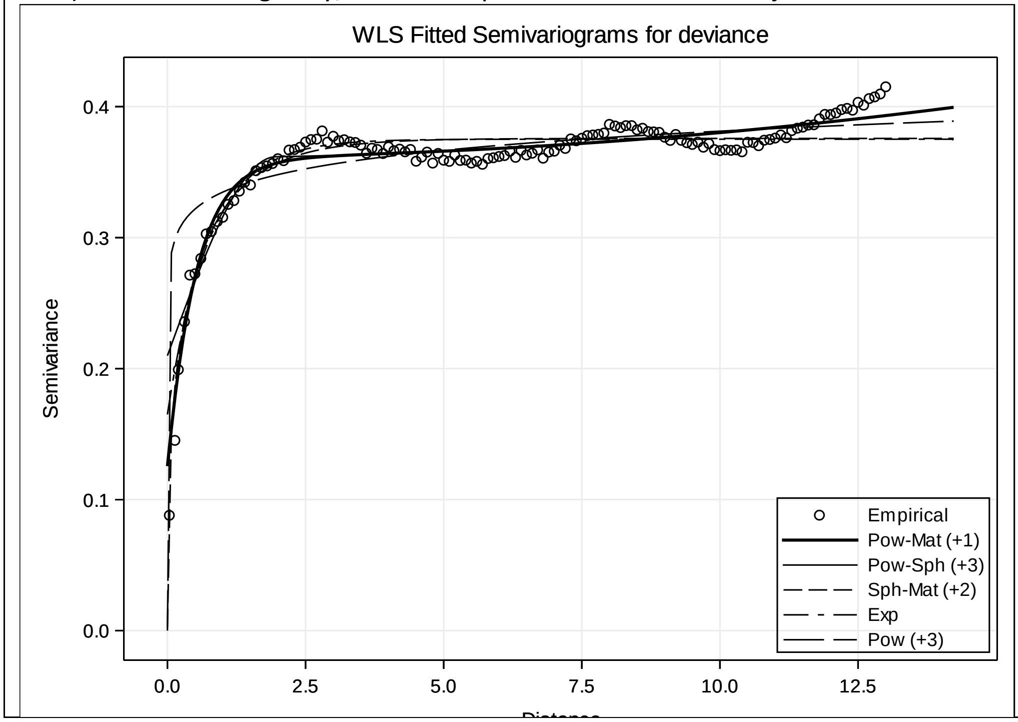

ae.1) Highway is Barrier, 3rd order spatial detrend

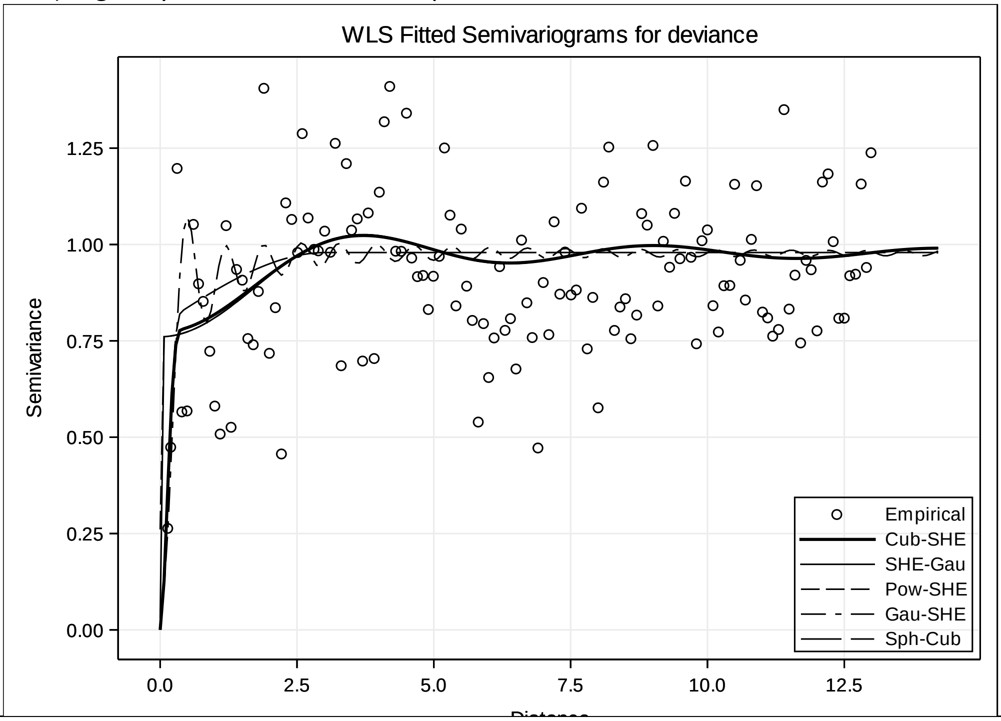

ae.2) Highway is Barrier, 3rd order spatial detrend + rater adjustment

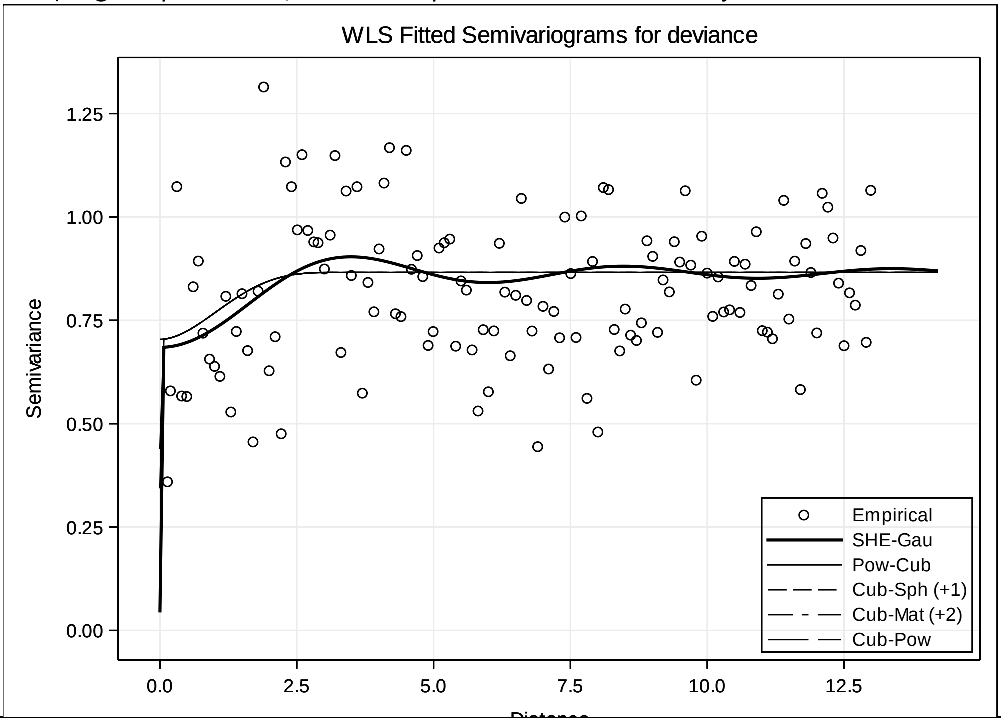

Figure S2.a-ae 1) (3rd order spatial detrending only) and 2)(3rd order spatial detrending + rater adjustment) experimental and best-fit theoretical, directional semivariograms of neighborhood audit item responses from approximately 8000 locations, Essex County NJ.

a.1) Garbage, 3rd order spatial detrend

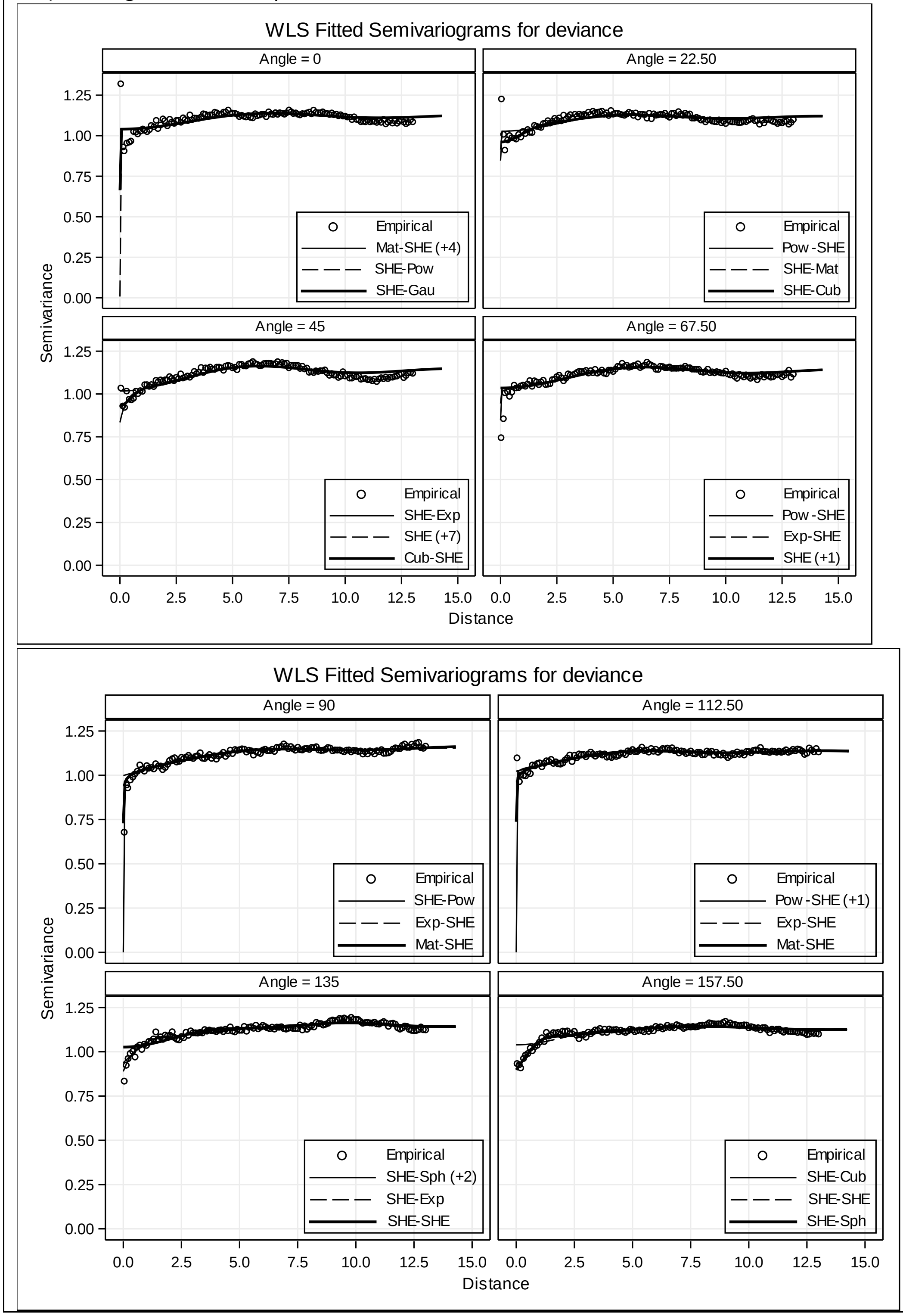

a.2) Garbage, 3rd order spatial detrend + rater adjustment

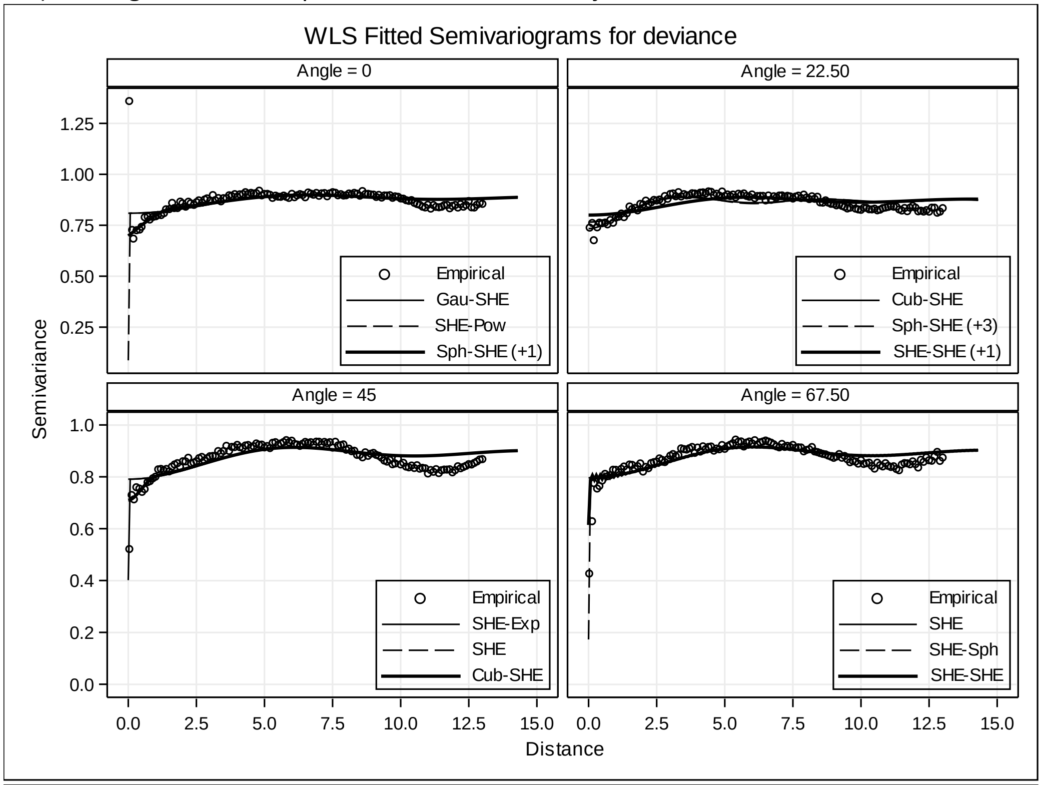

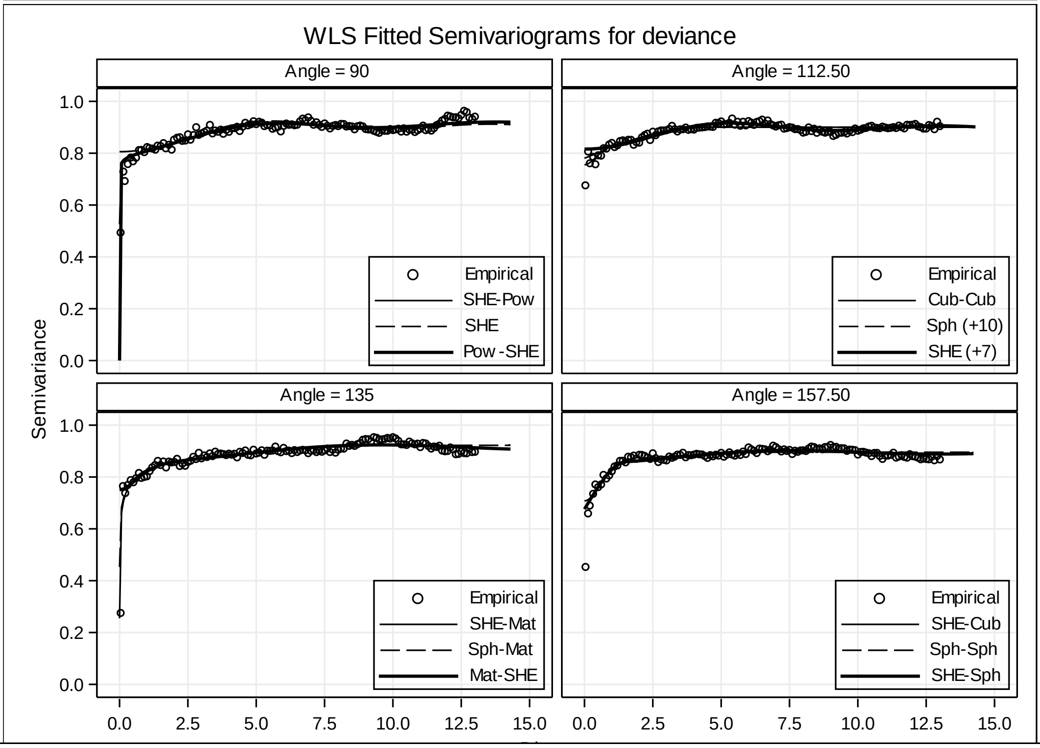

b.1) Abandoned Cars, 3rd order spatial detrend – Non-estimable due to sparse data

b.2) Abandoned Cars, 3rd order spatial detrend + rater adjustment-
Non-estimable due to sparse data

c.1) Building Conditions ≥ Moderate, 3rd order spatial detrend

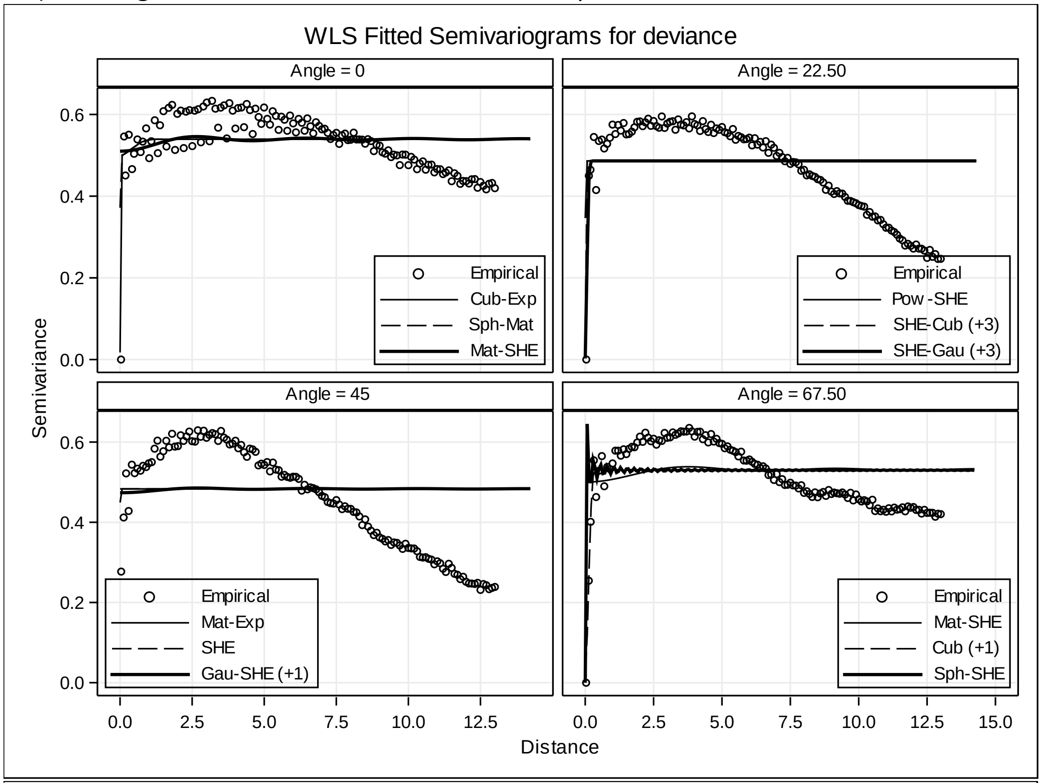

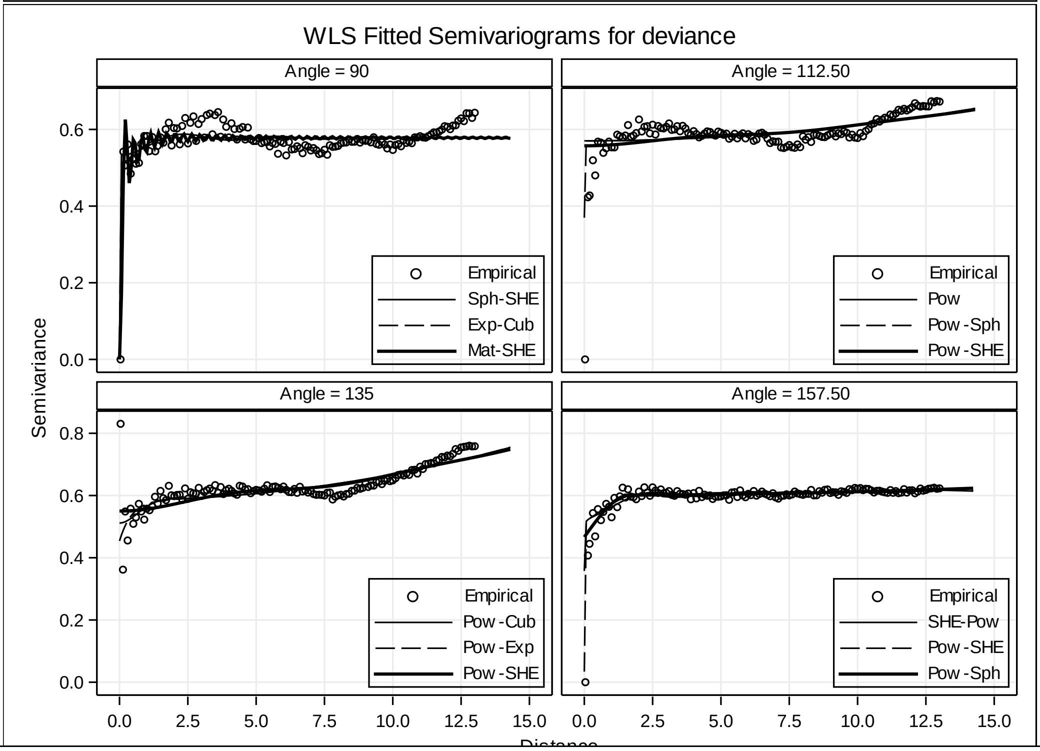

c.2) Building Conditions ≥ Moderate, 3rd order spatial detrend + rater adjustment

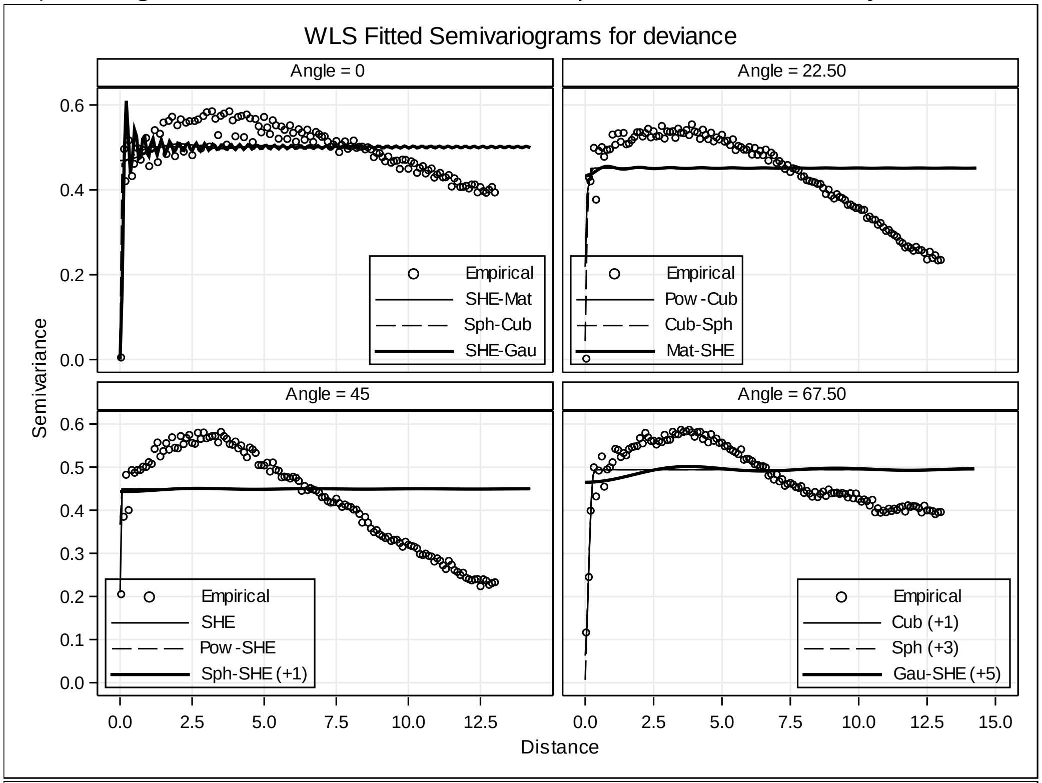

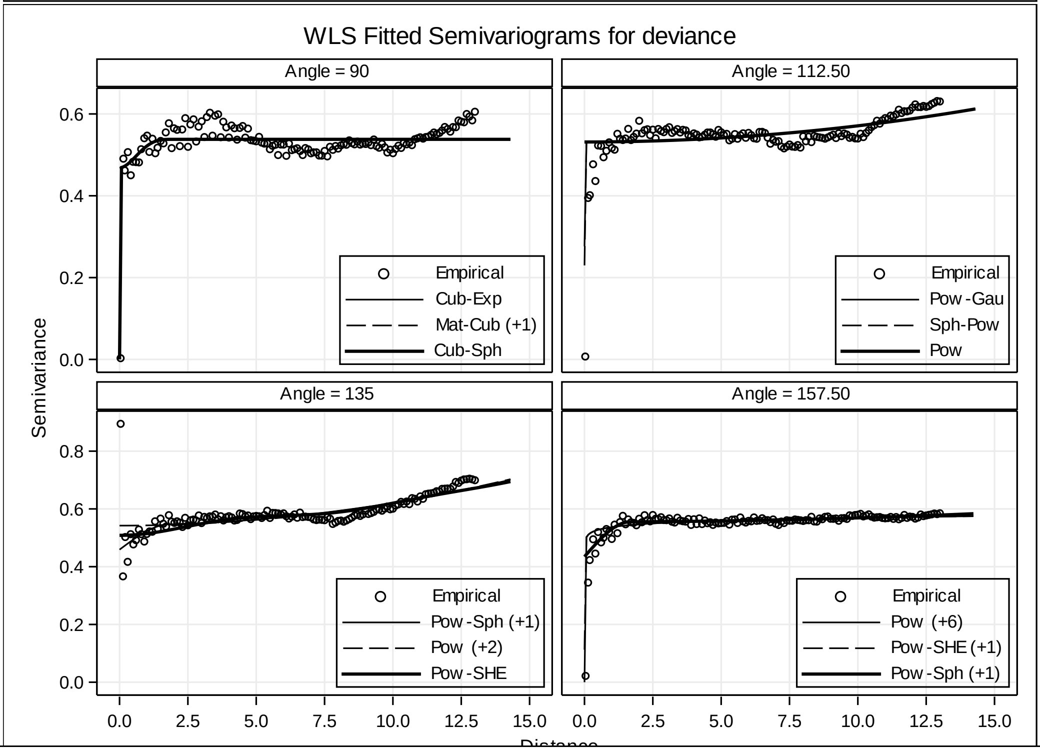

d.1) Yard Conditions ≥ Moderate, 3rd order spatial detrend

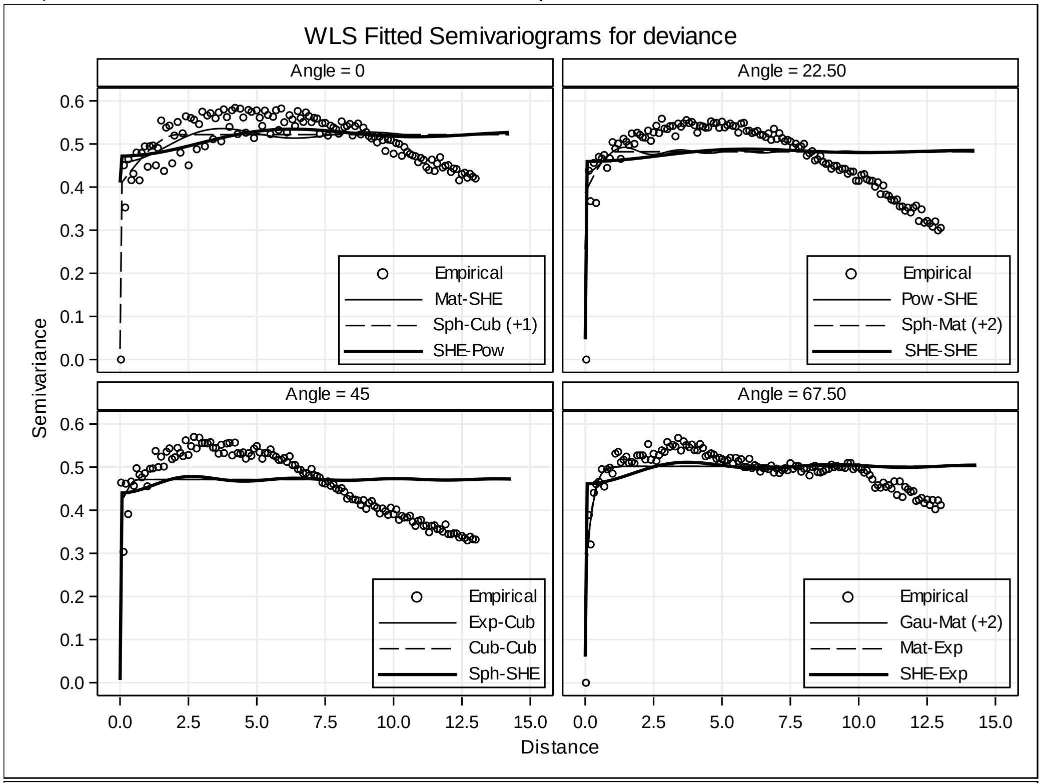

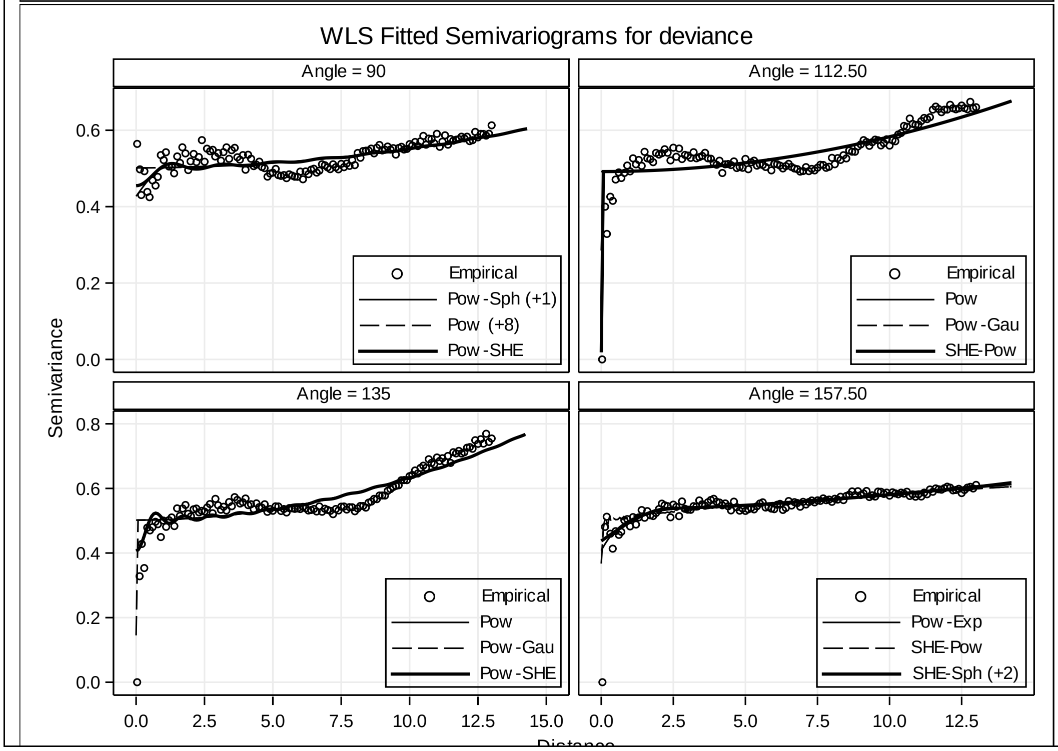

d.2) Yard Conditions ≥ Moderate, 3rd order spatial detrend + rater adjustment

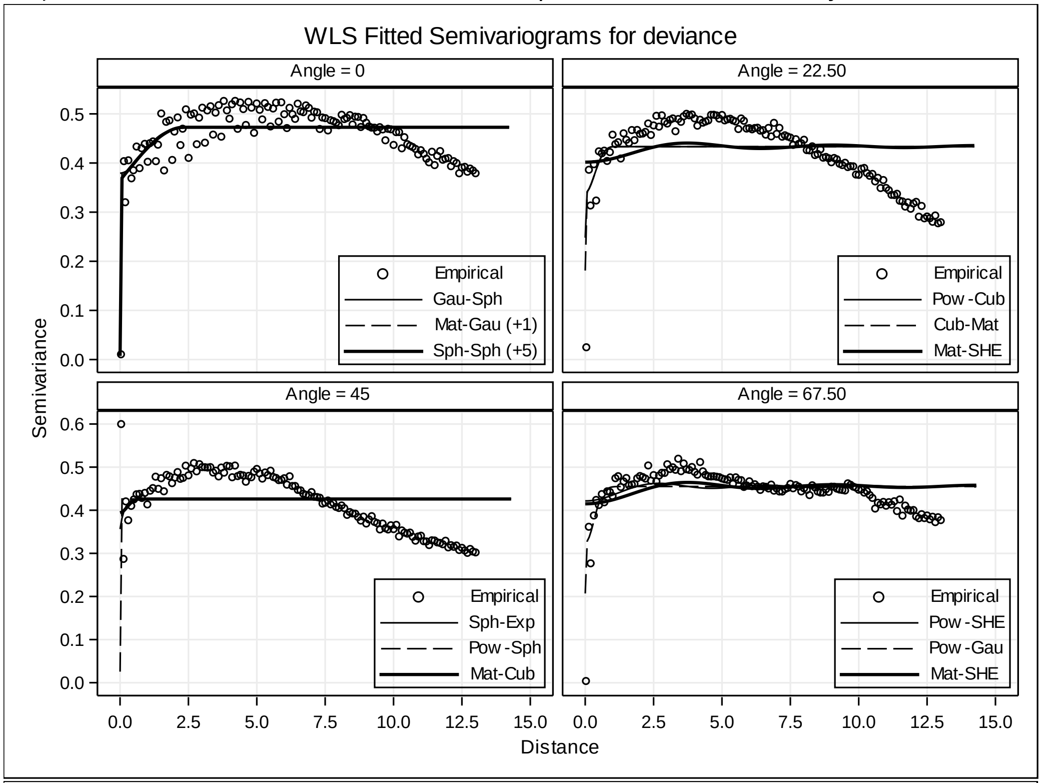

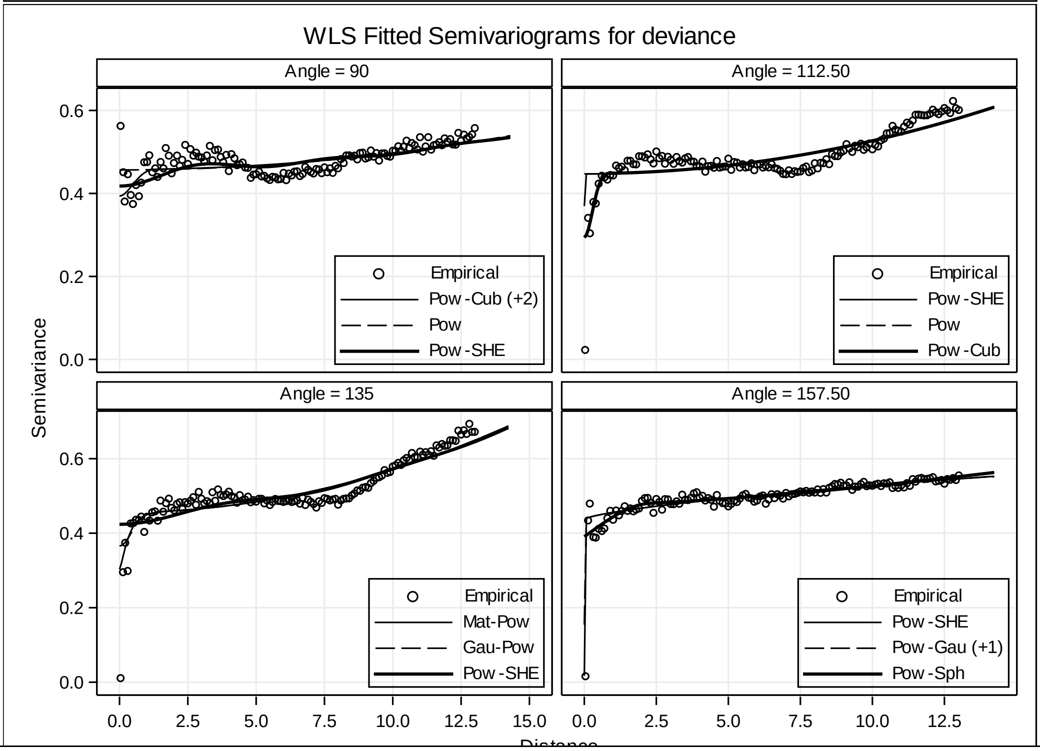

e.1) Dumpsters, 3rd order spatial detrend

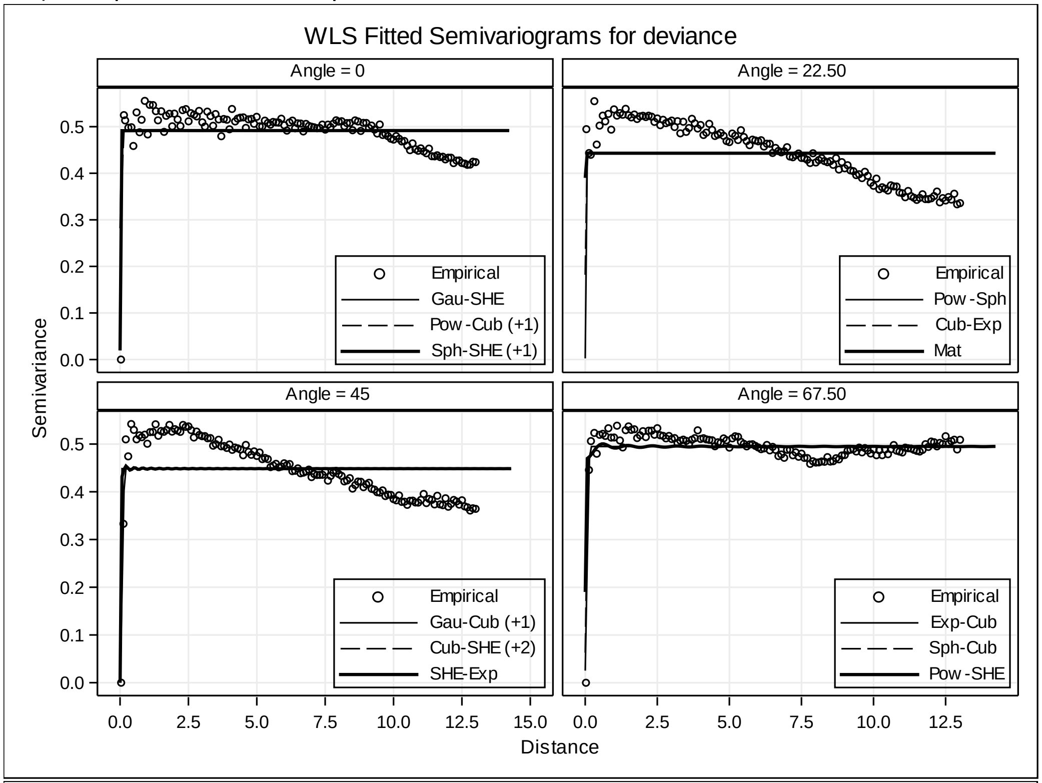

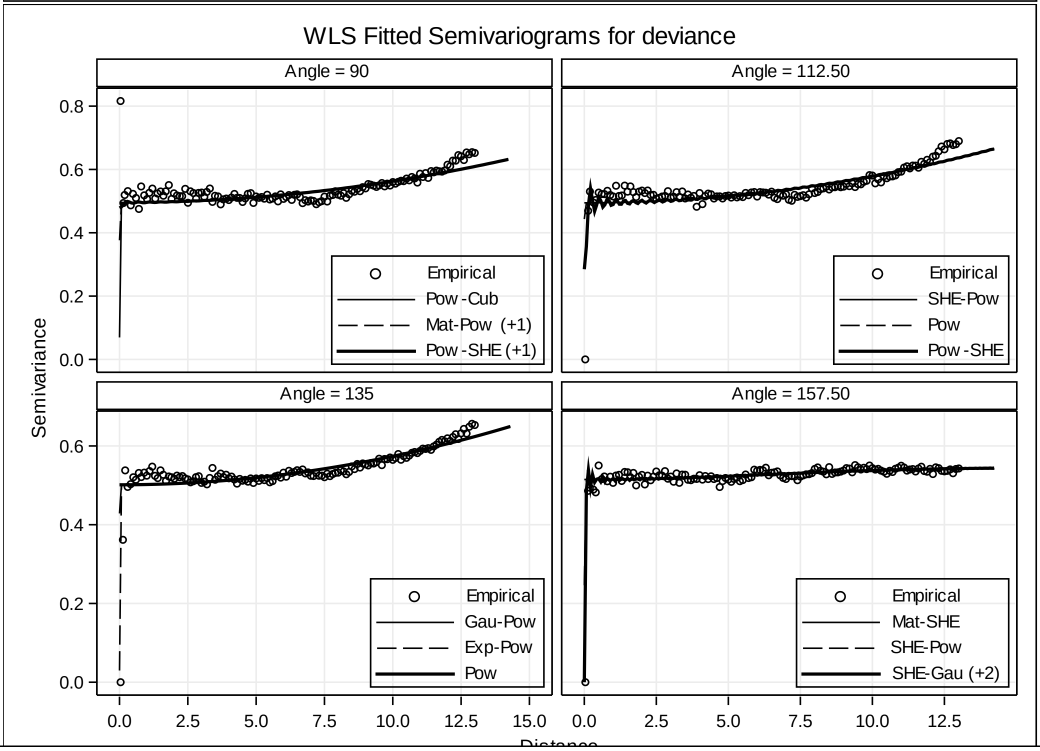

e.2) Dumpsters, 3rd order spatial detrend + rater adjustment

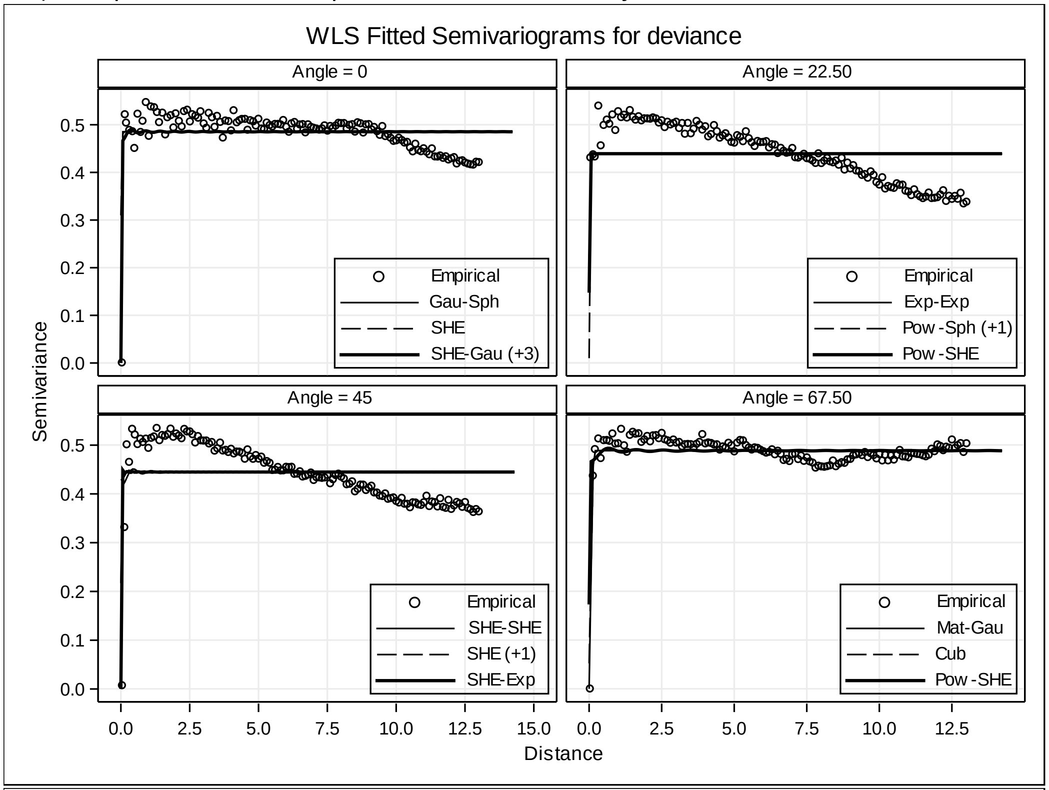

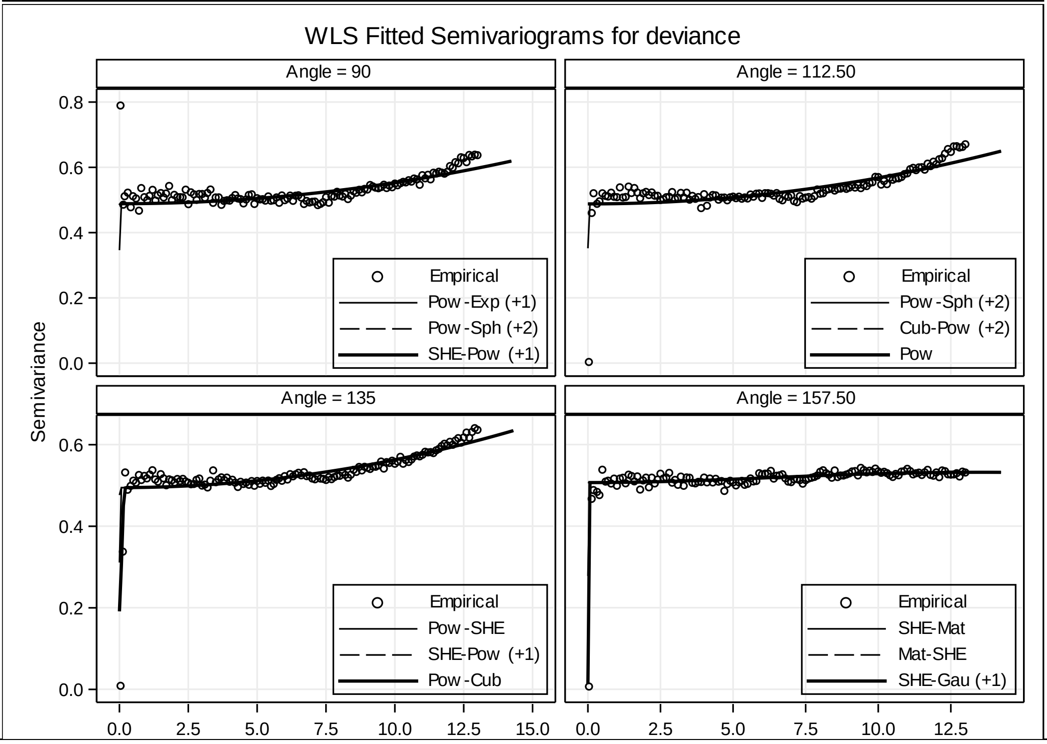

f.1) Graffiti, 3rd order spatial detrend

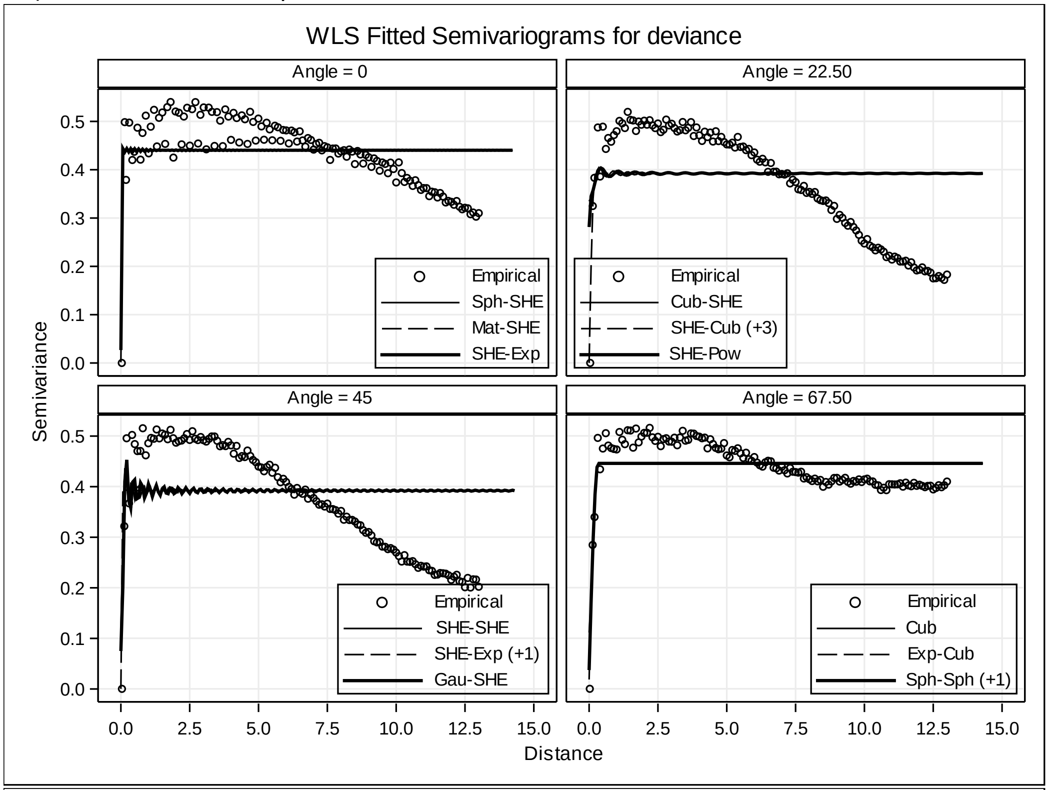

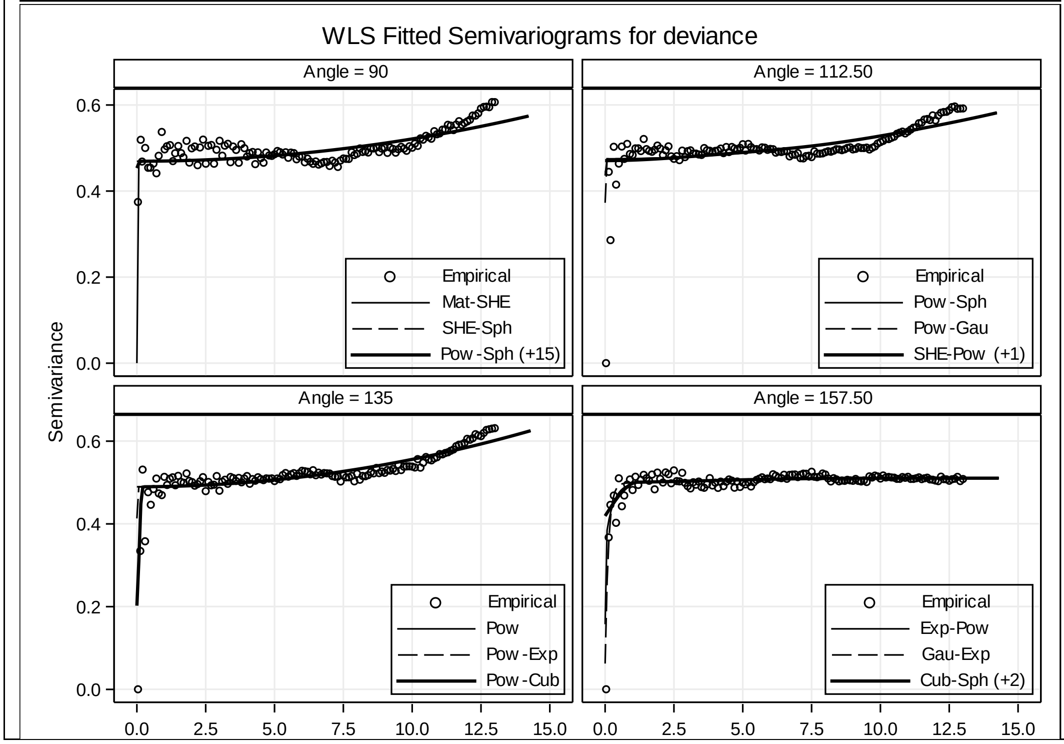

f.2) Graffiti, 3rd order spatial detrend + rater adjustment

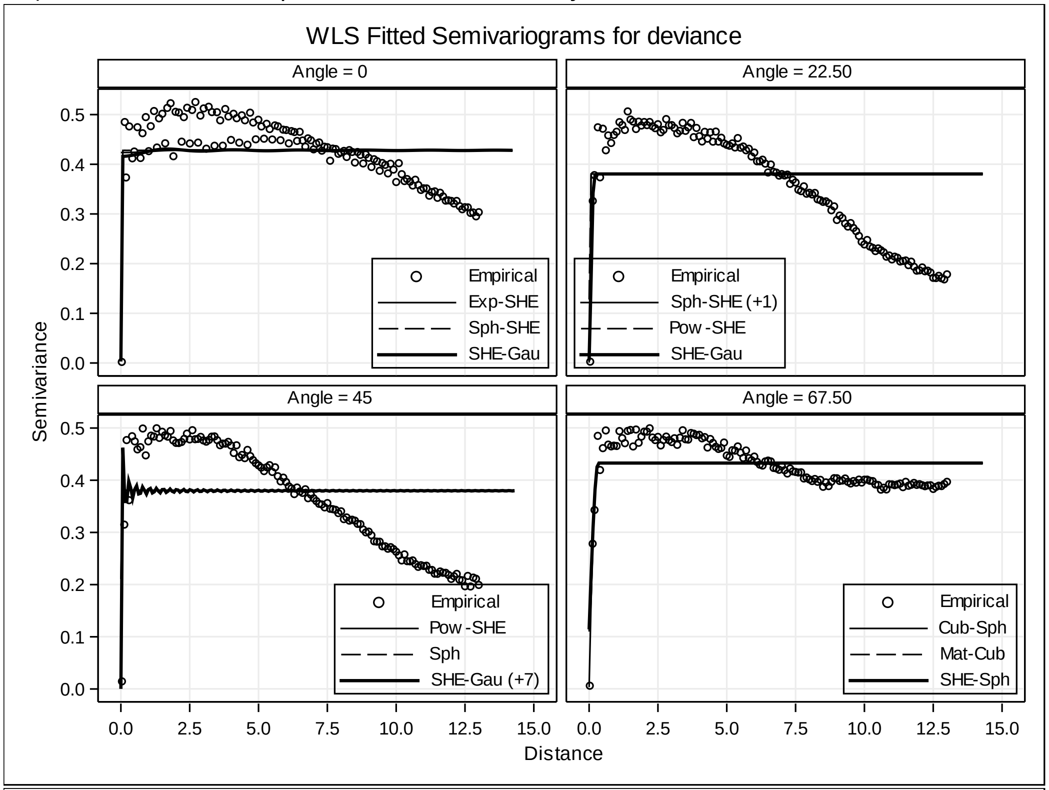

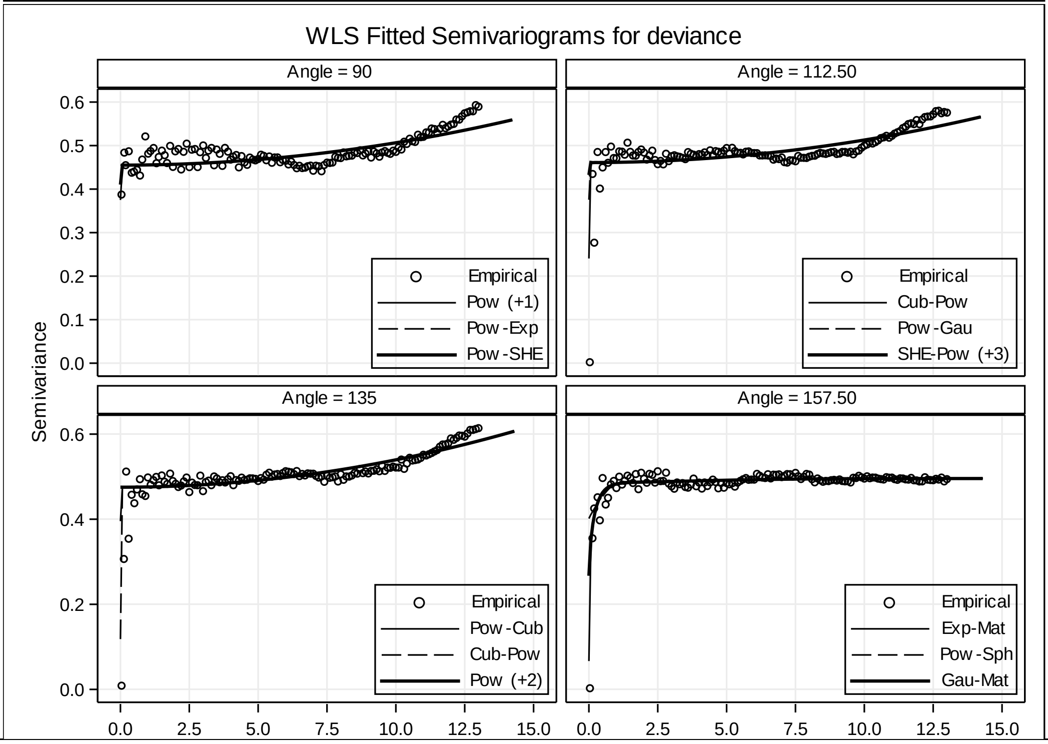

g.1) Boarded/burned Buildings, 3rd order spatial detrend

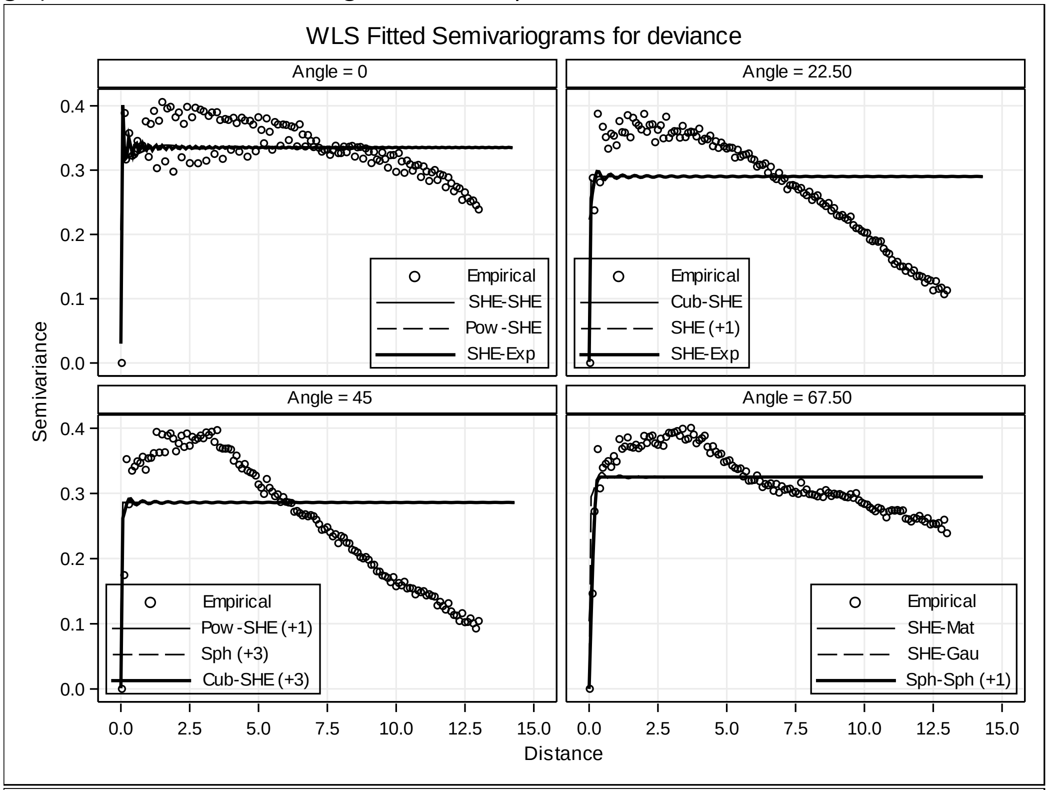

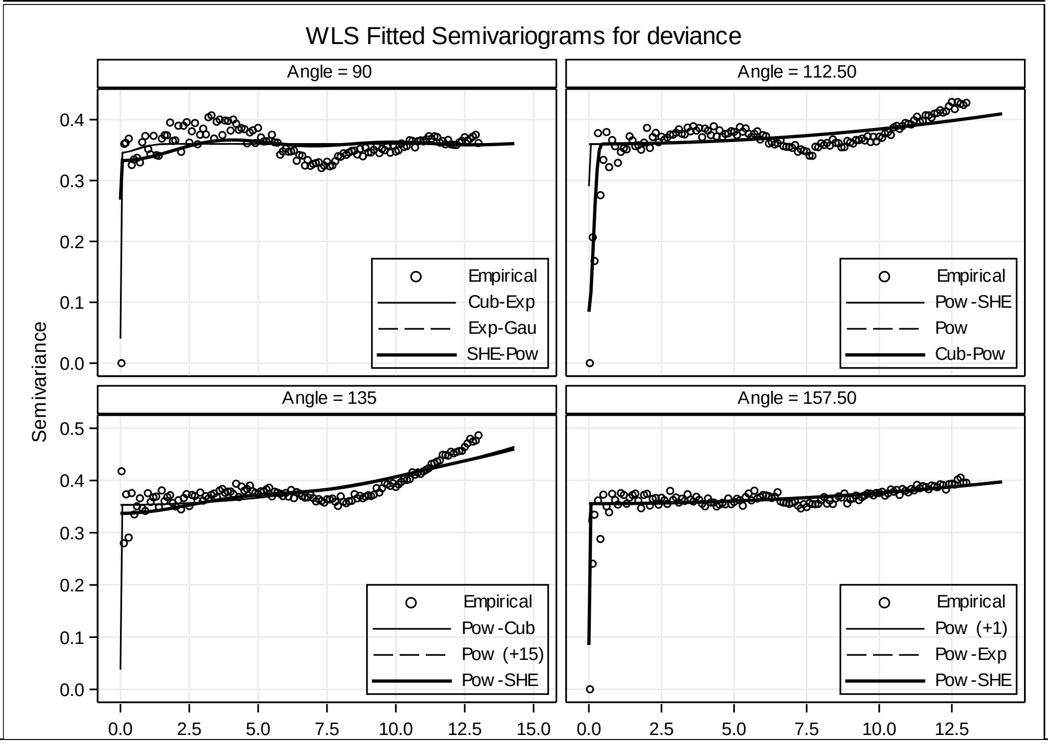

g.2) Boarded/burned Buildings, 3rd order spatial detrend + rater adjustment

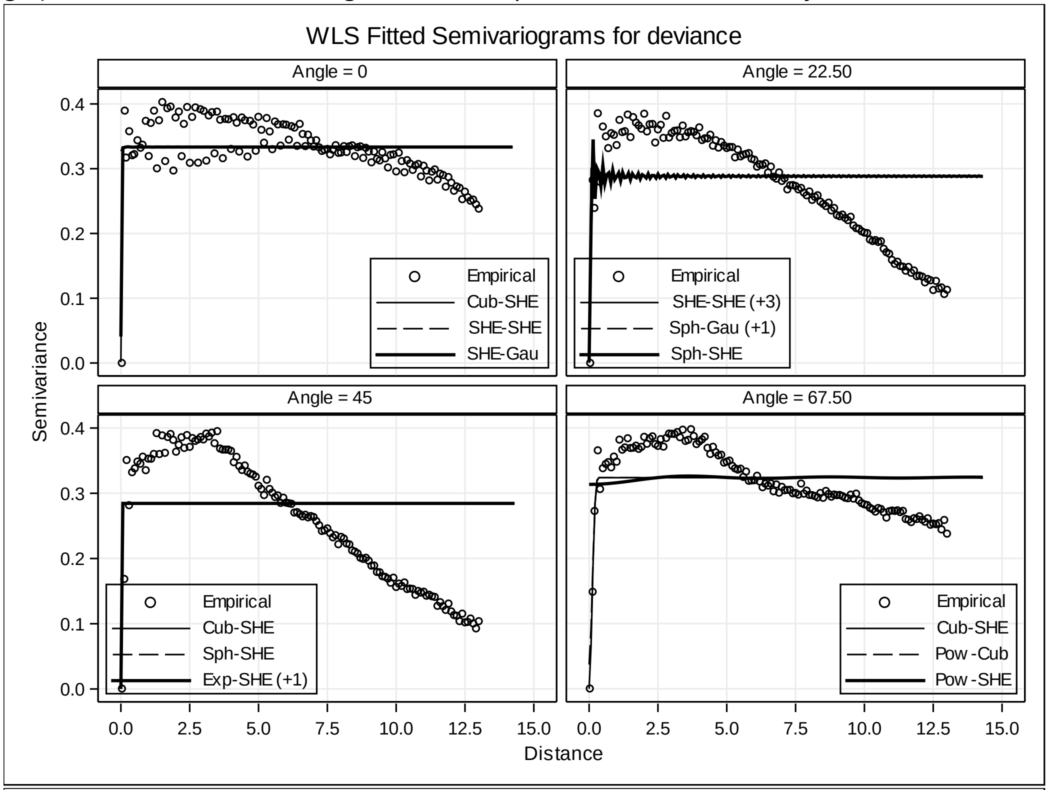

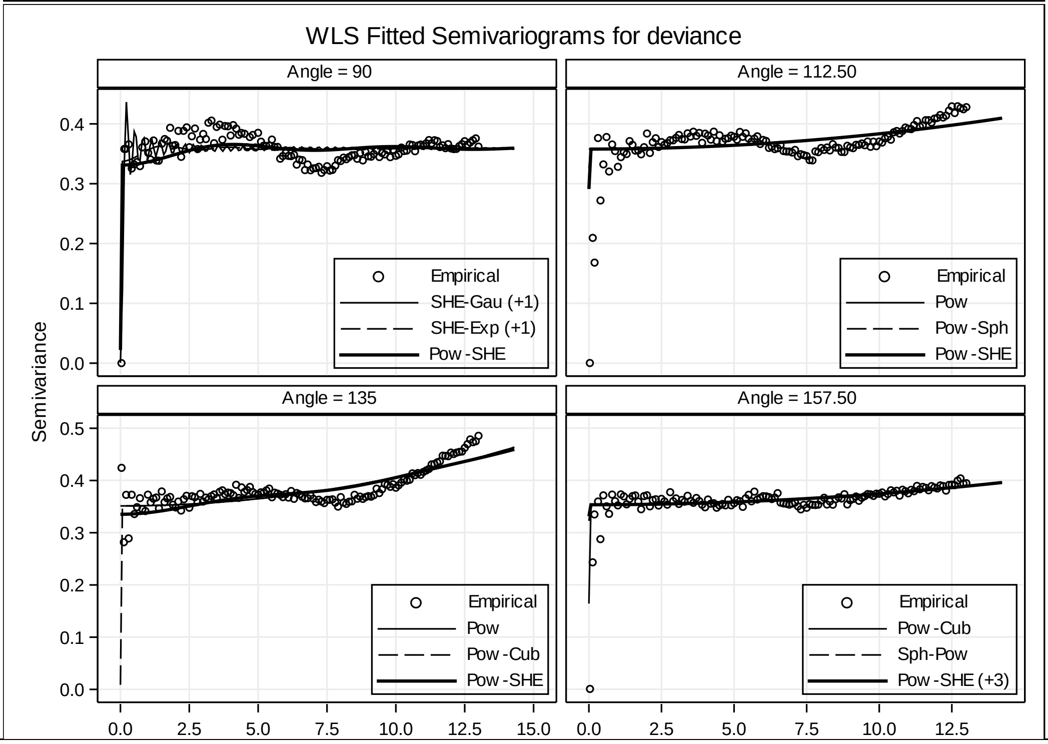

h.1) Outdoor Seating, 3rd order spatial detrend

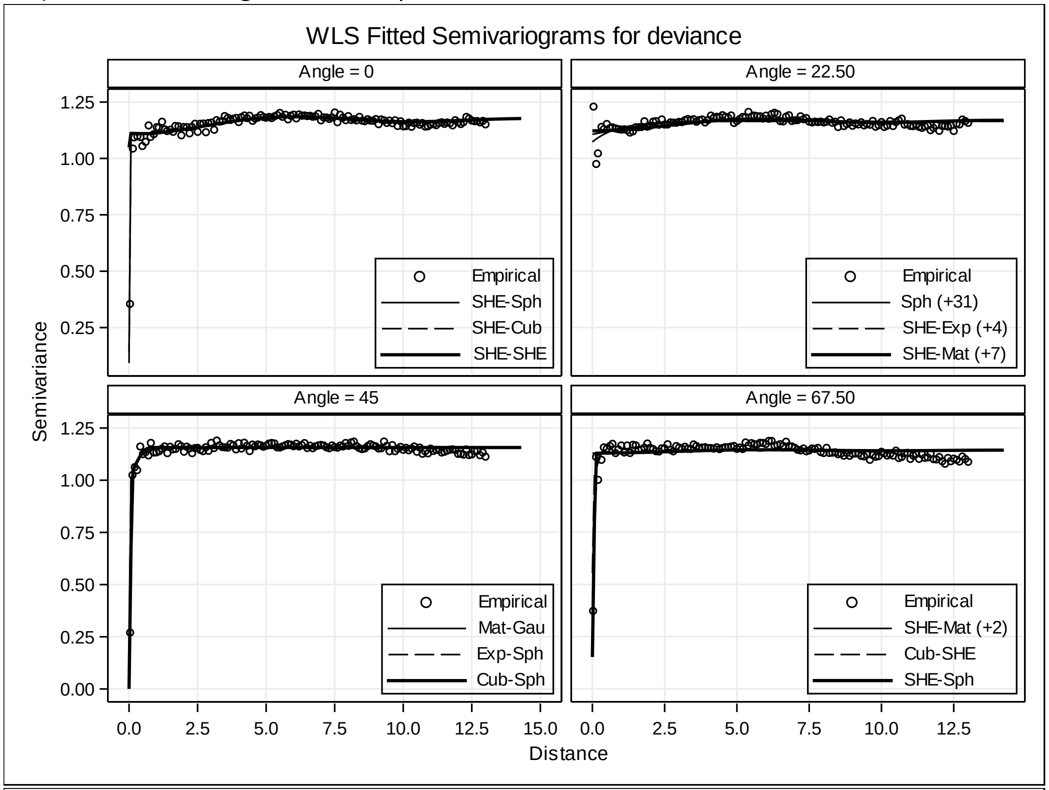

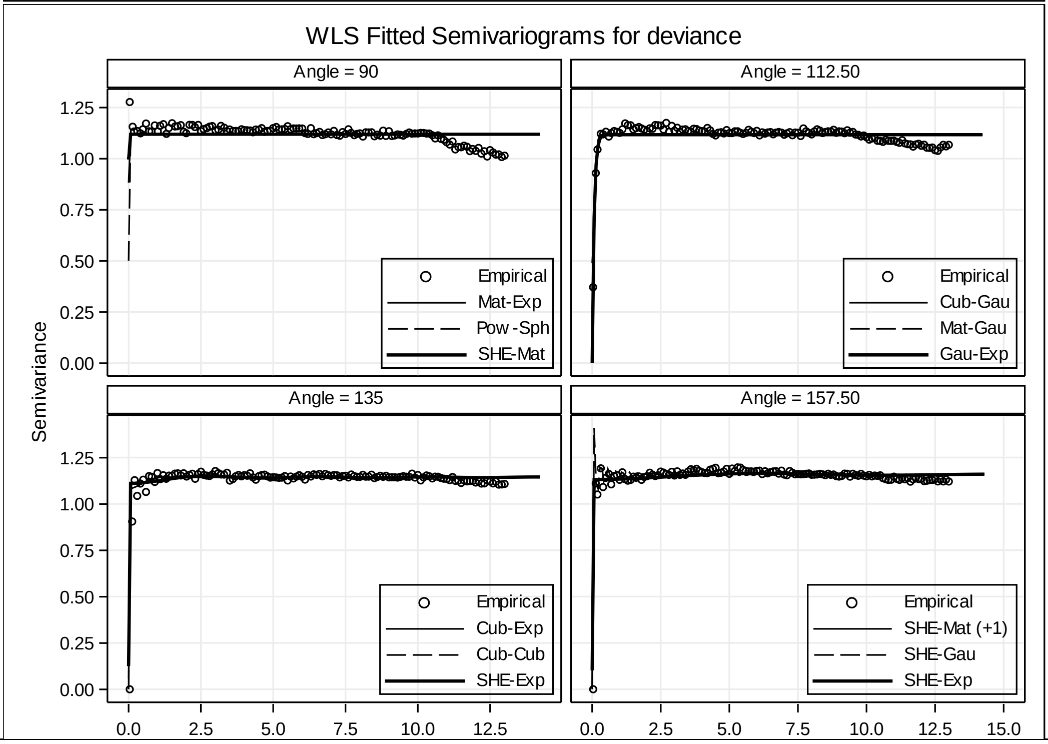

h.2) Outdoor Seating, 3rd order spatial detrend + rater adjustment

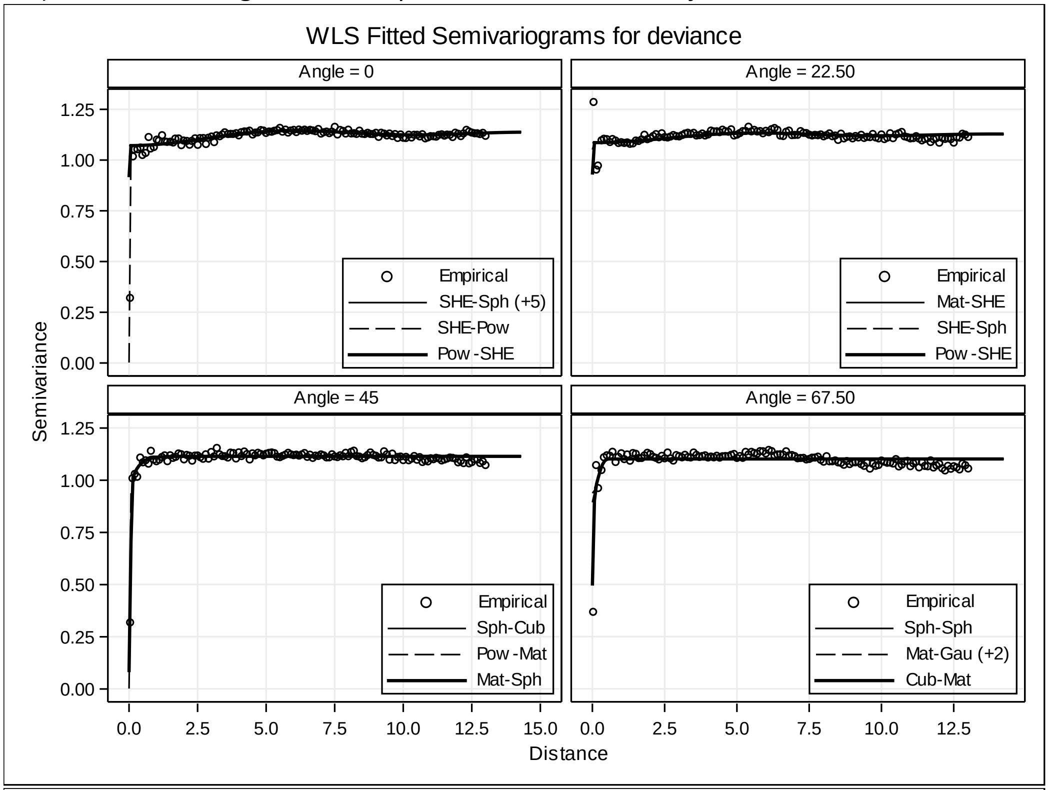

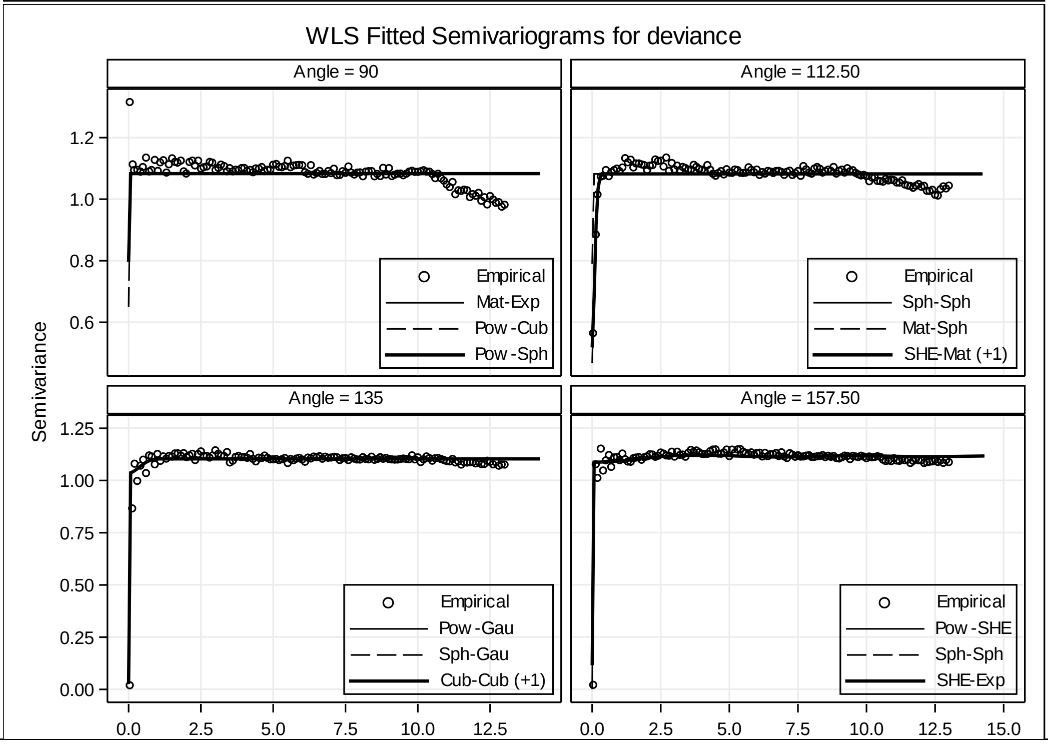

i.1) Team Sports, 3rd order spatial detrend

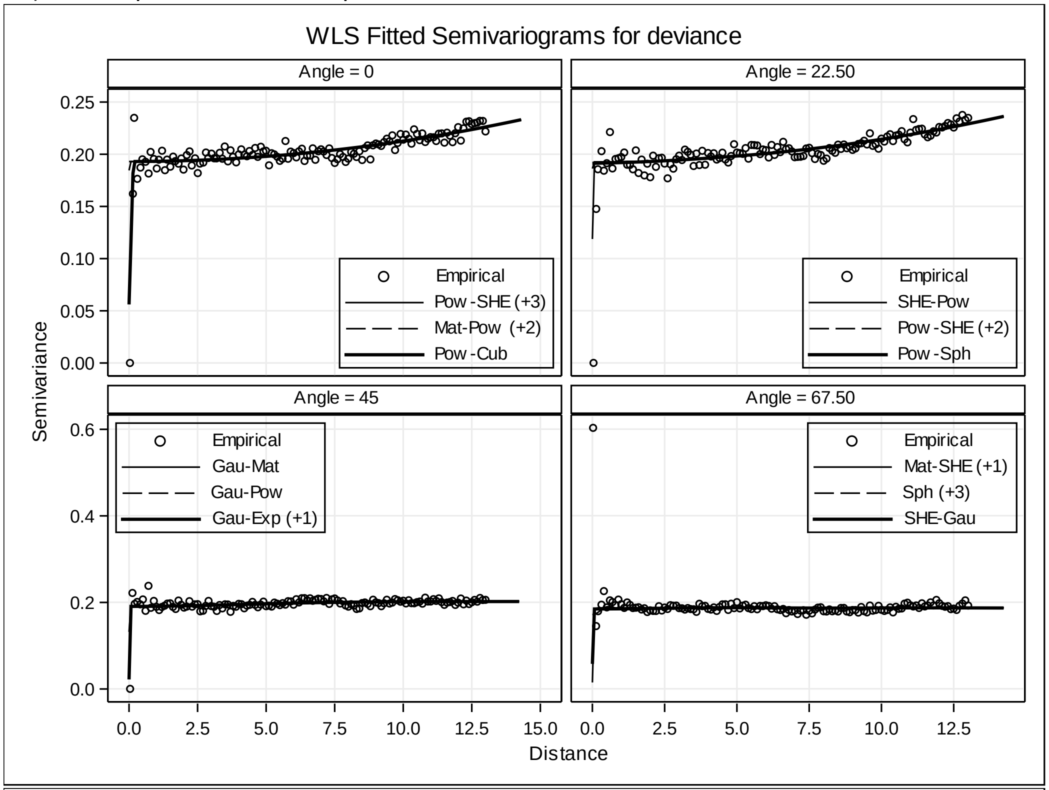

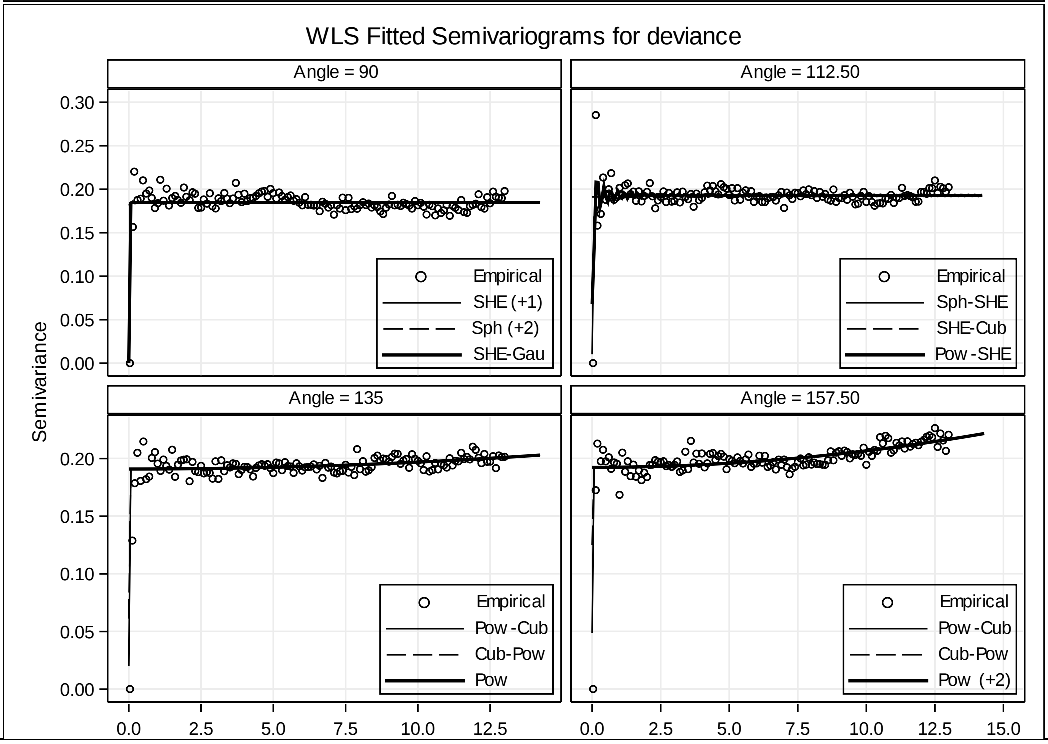

i.2) Team Sports, 3rd order spatial detrend + rater adjustment

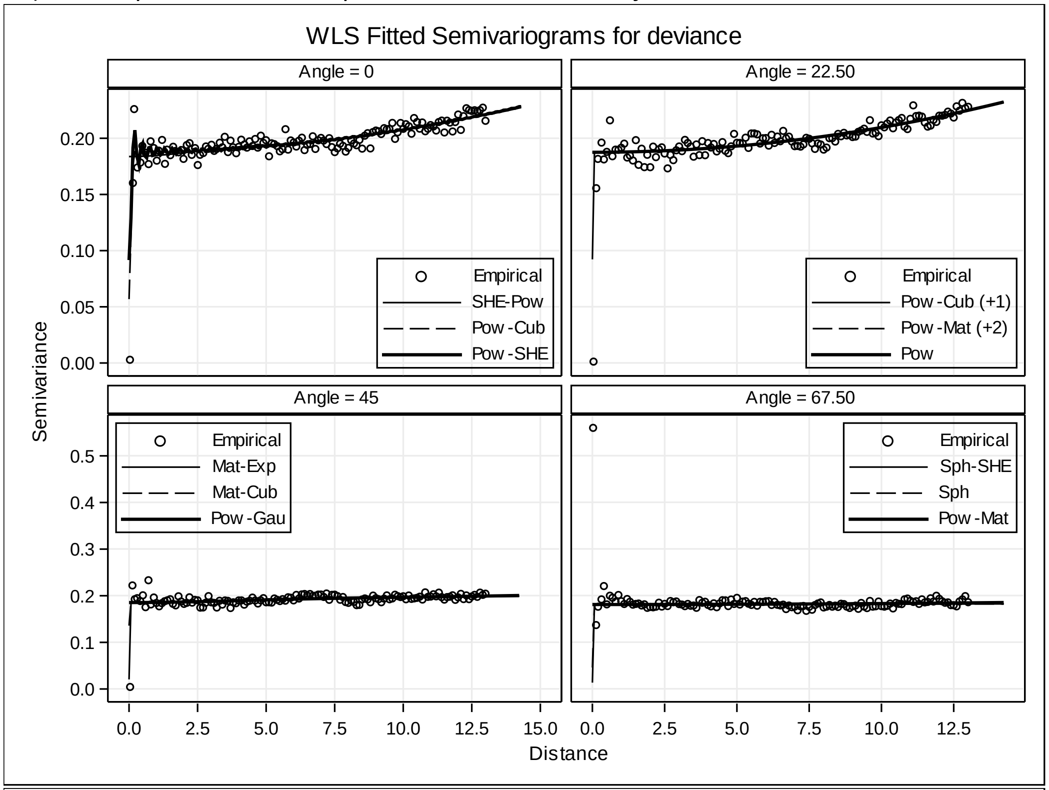

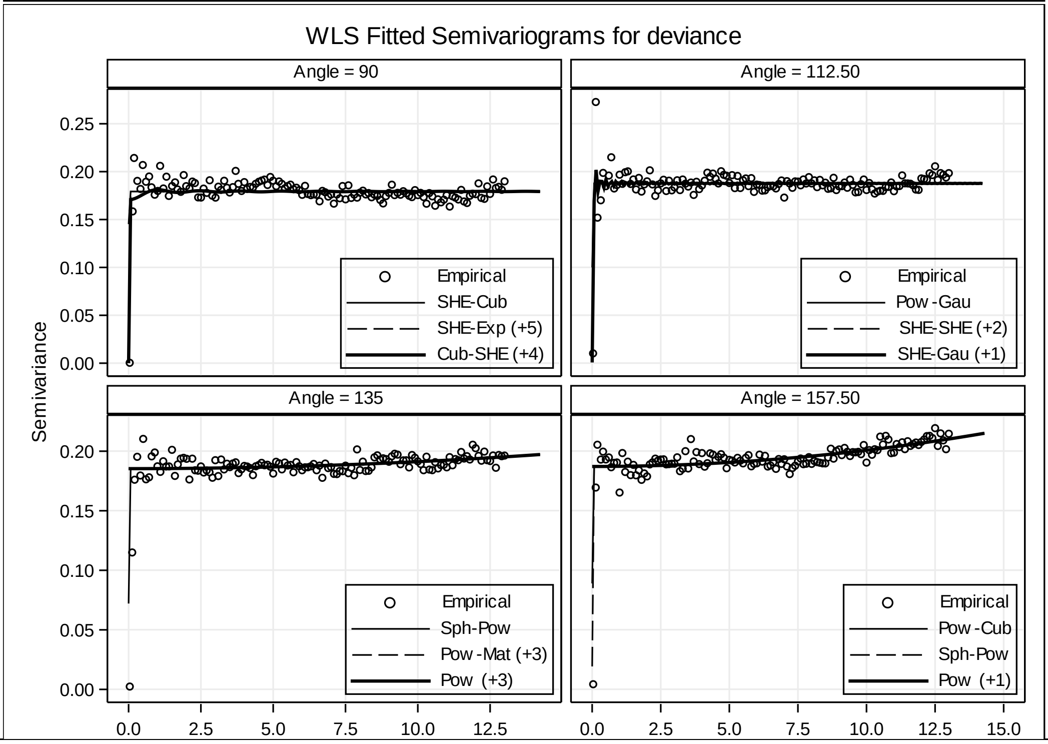

j.1) Yard Decorations, 3rd order spatial detrend

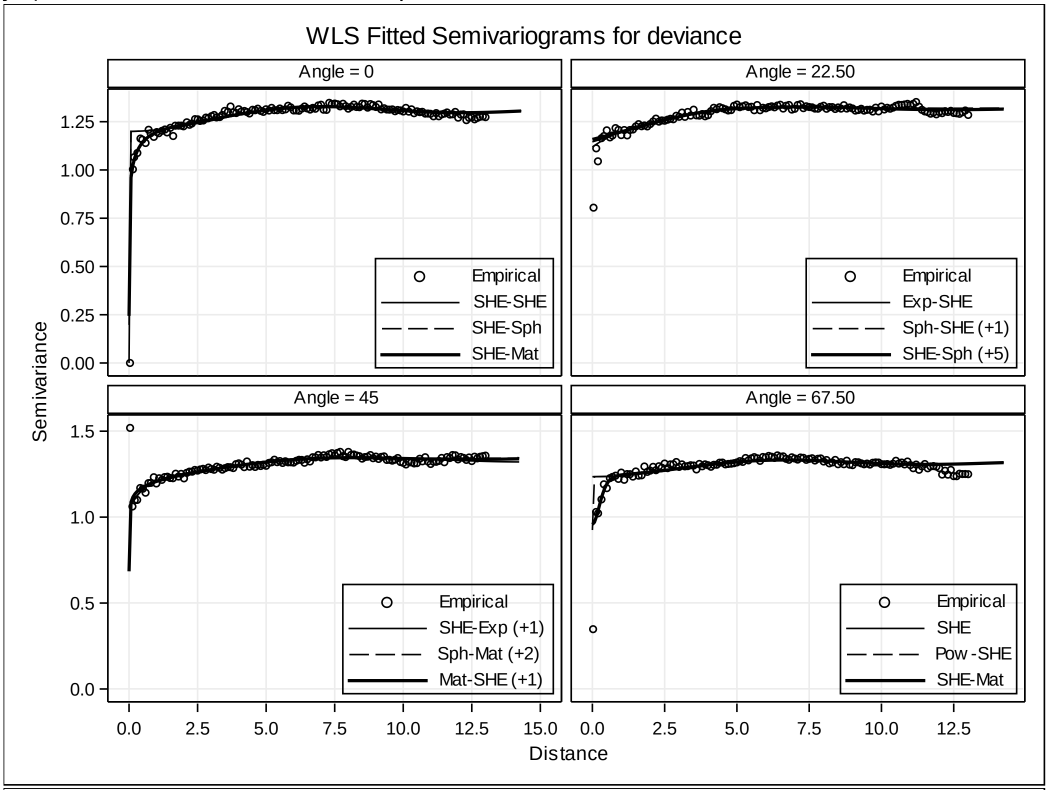

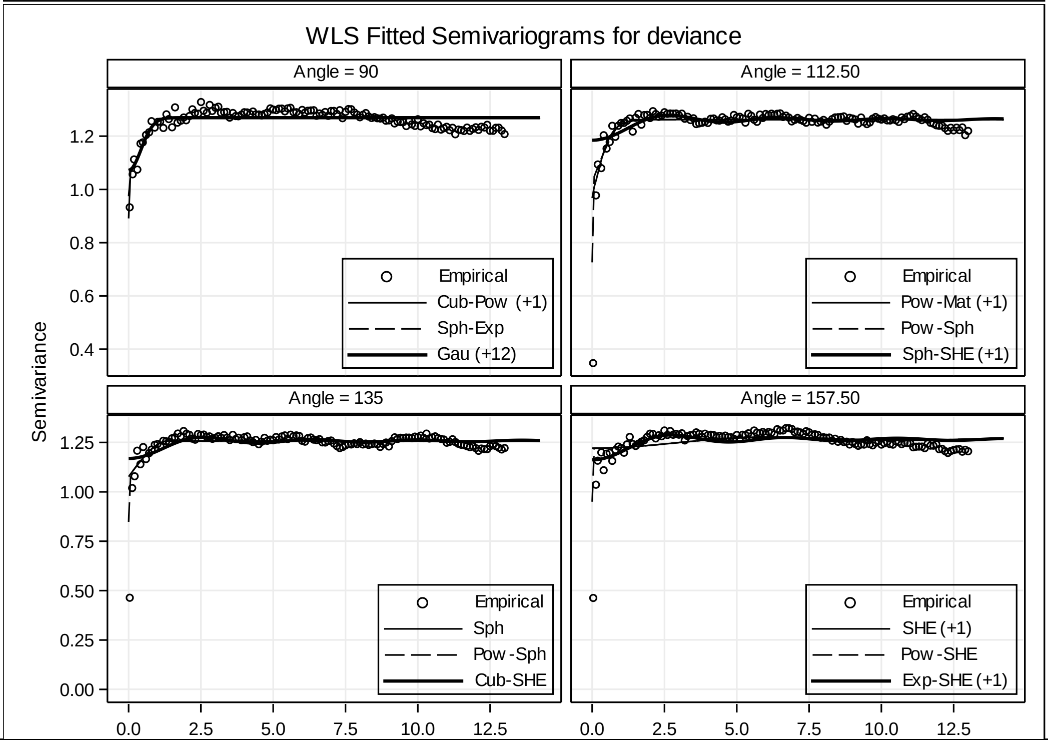

j.2) Yard Decorations, 3rd order spatial detrend + rater adjustment

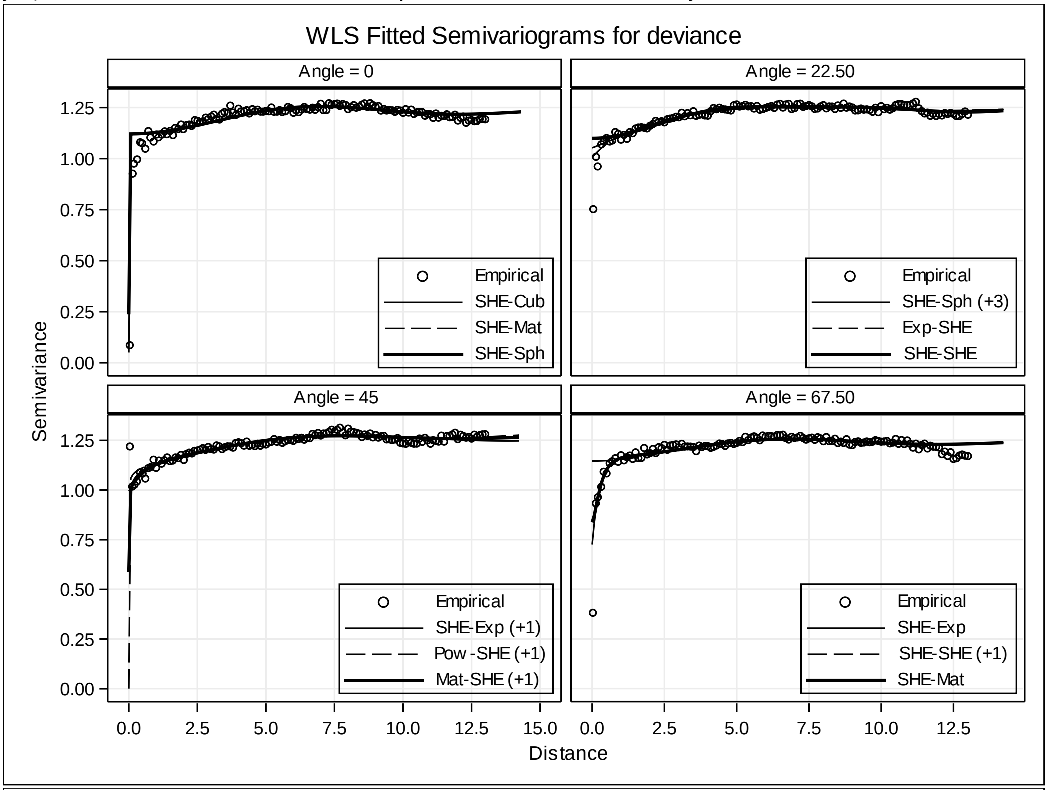

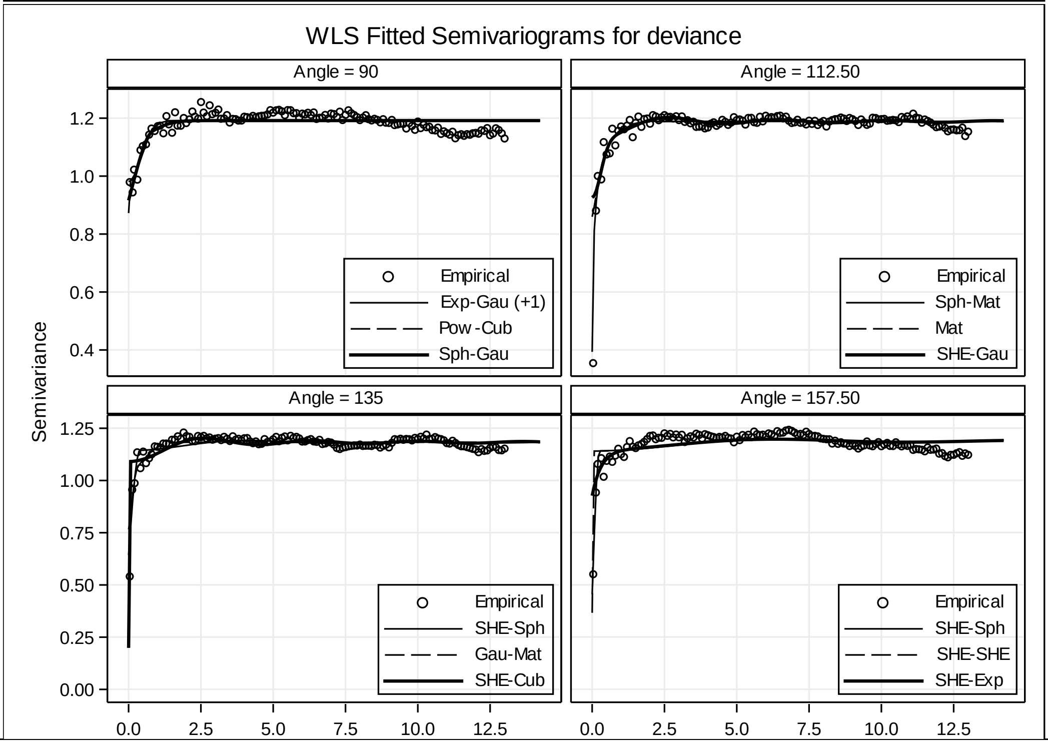

k.1) Fences, 3rd order spatial detrend

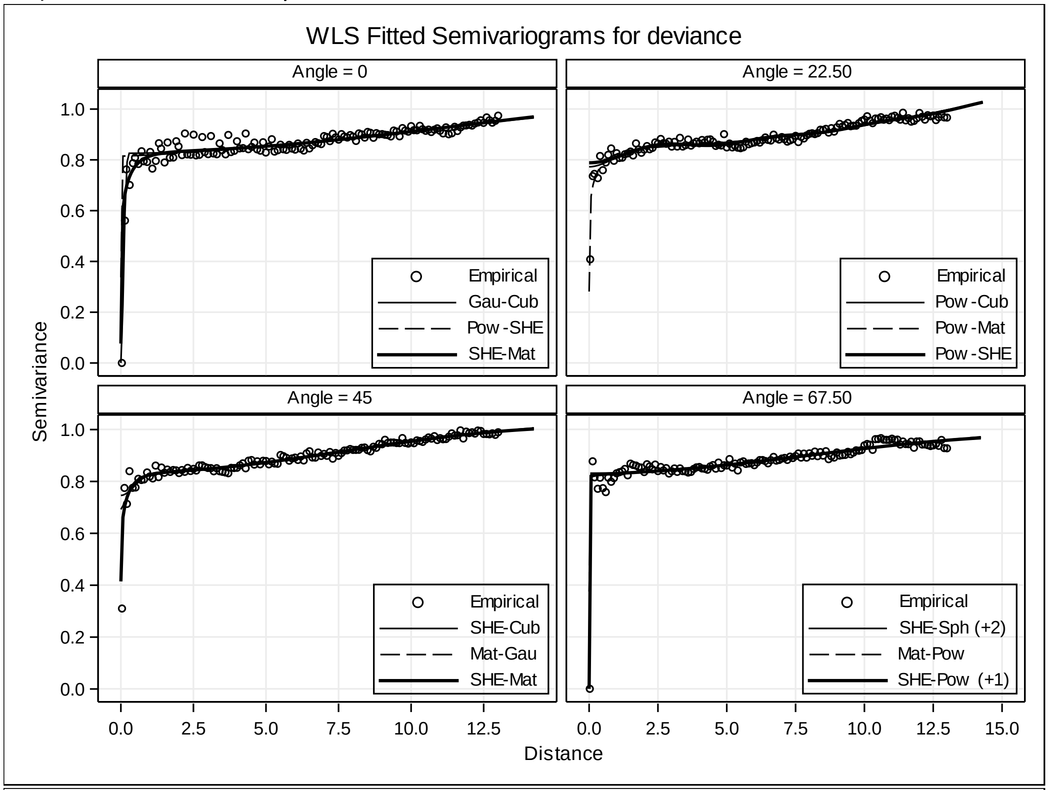

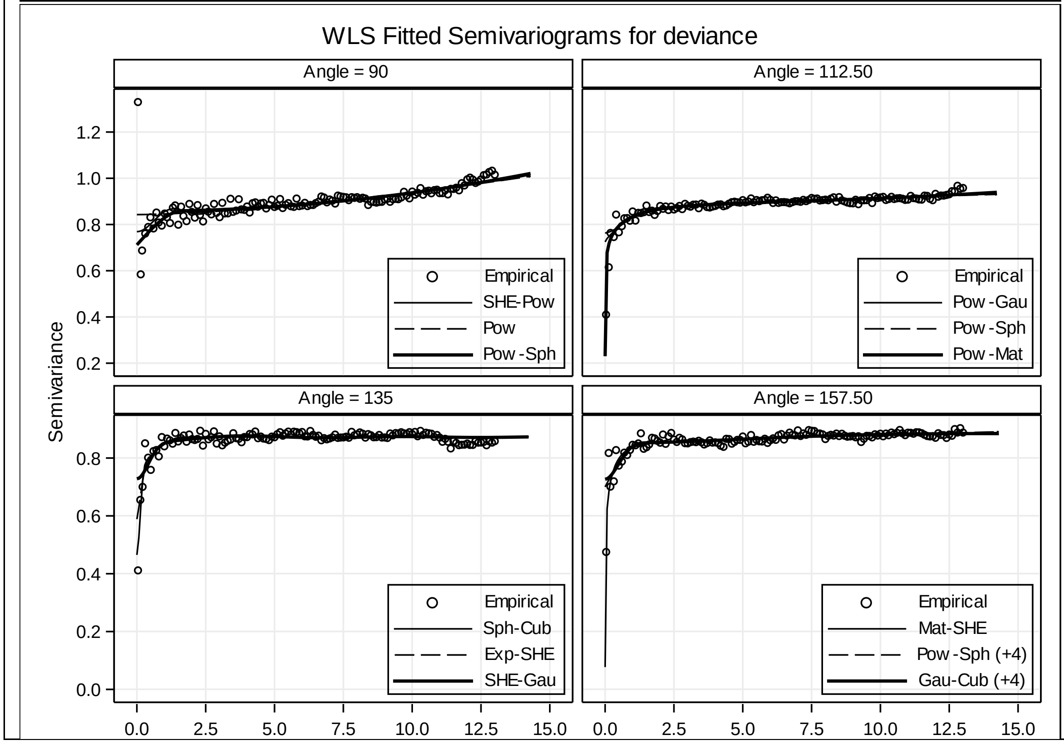

k.2) Fences, 3rd order spatial detrend + rater adjustment

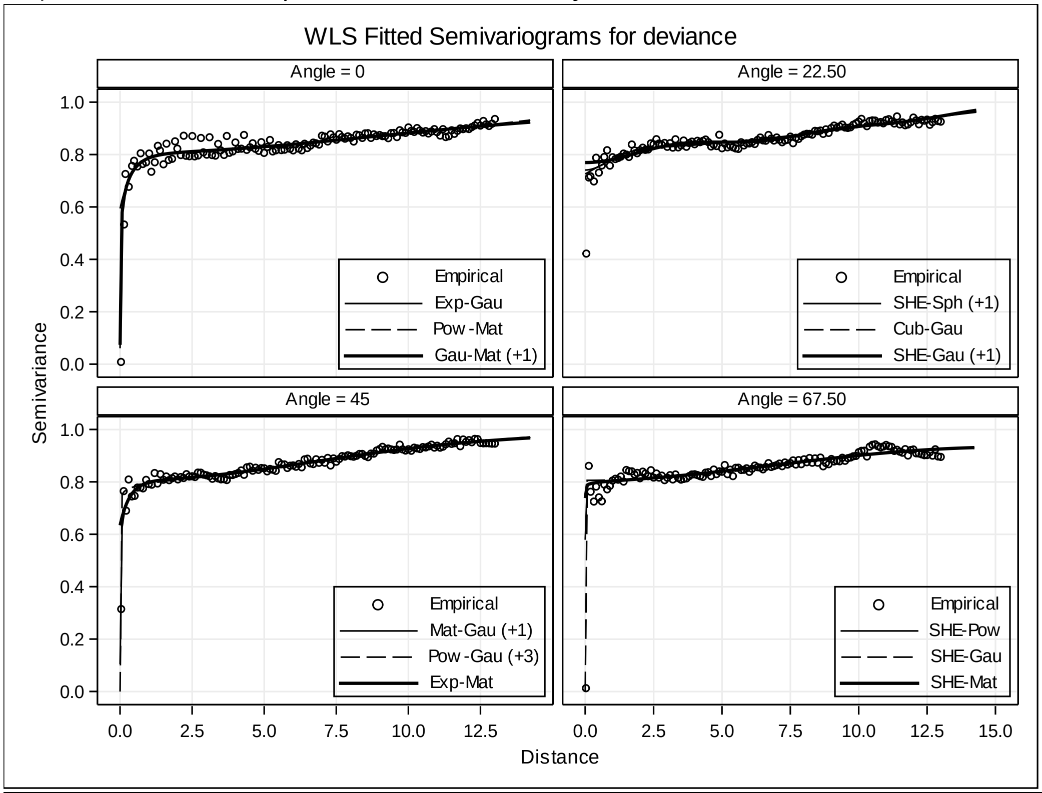

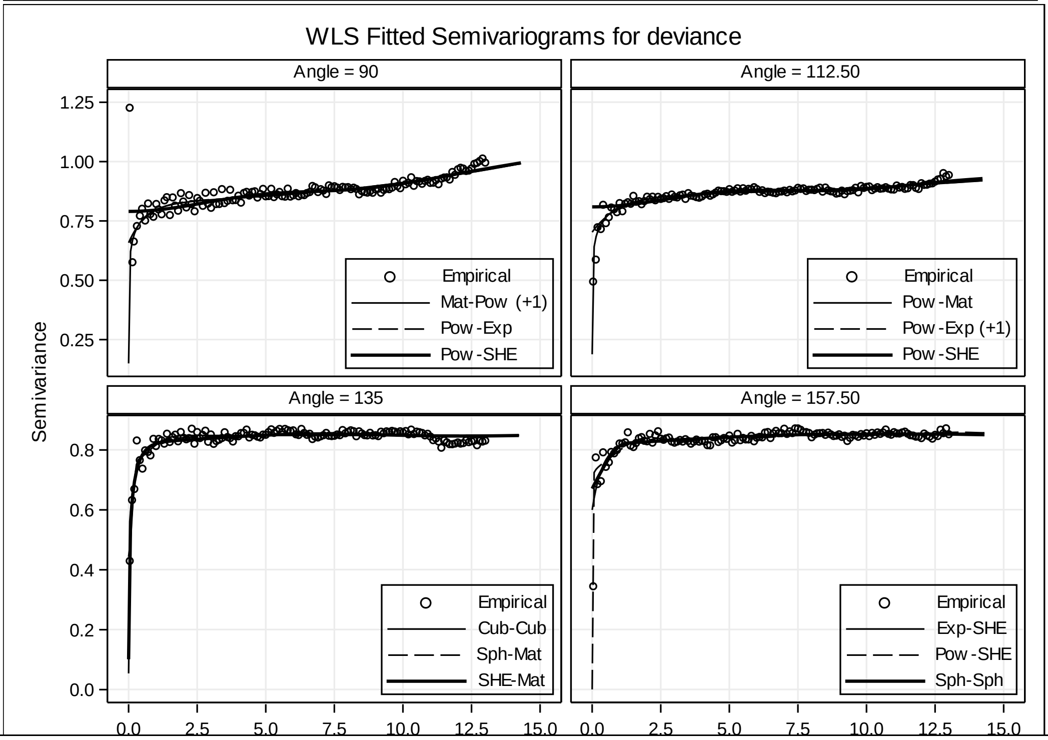

l.1) Sidewalk Present, 3rd order spatial detrend

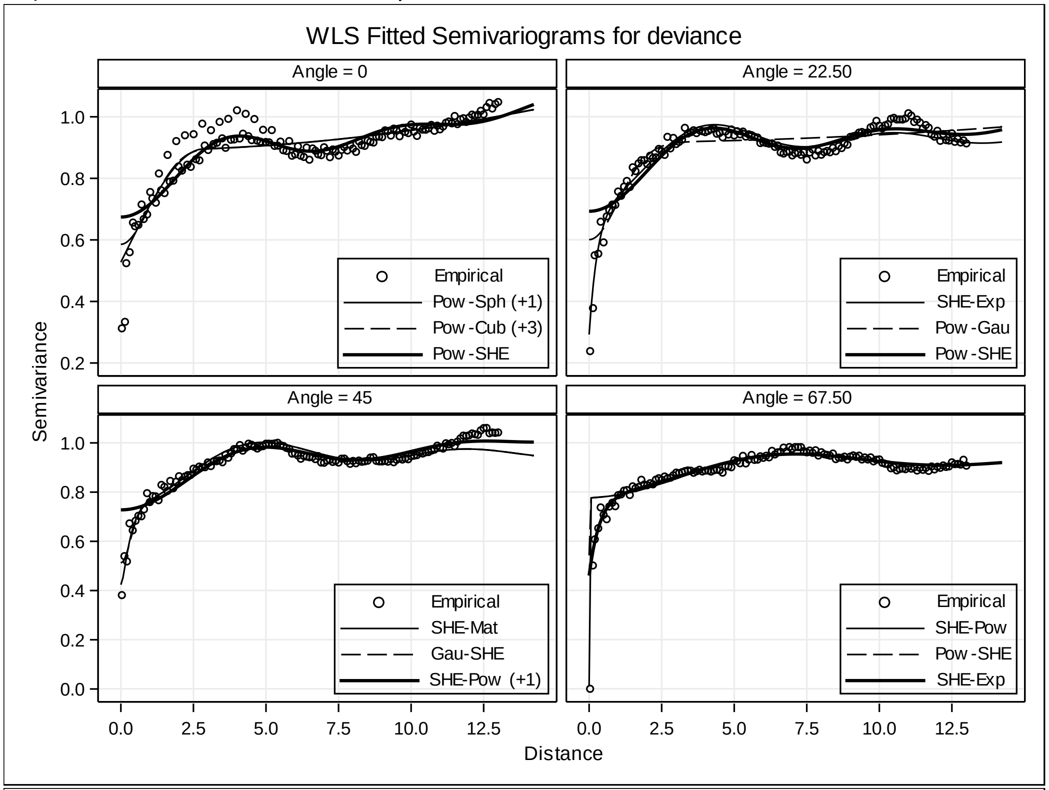

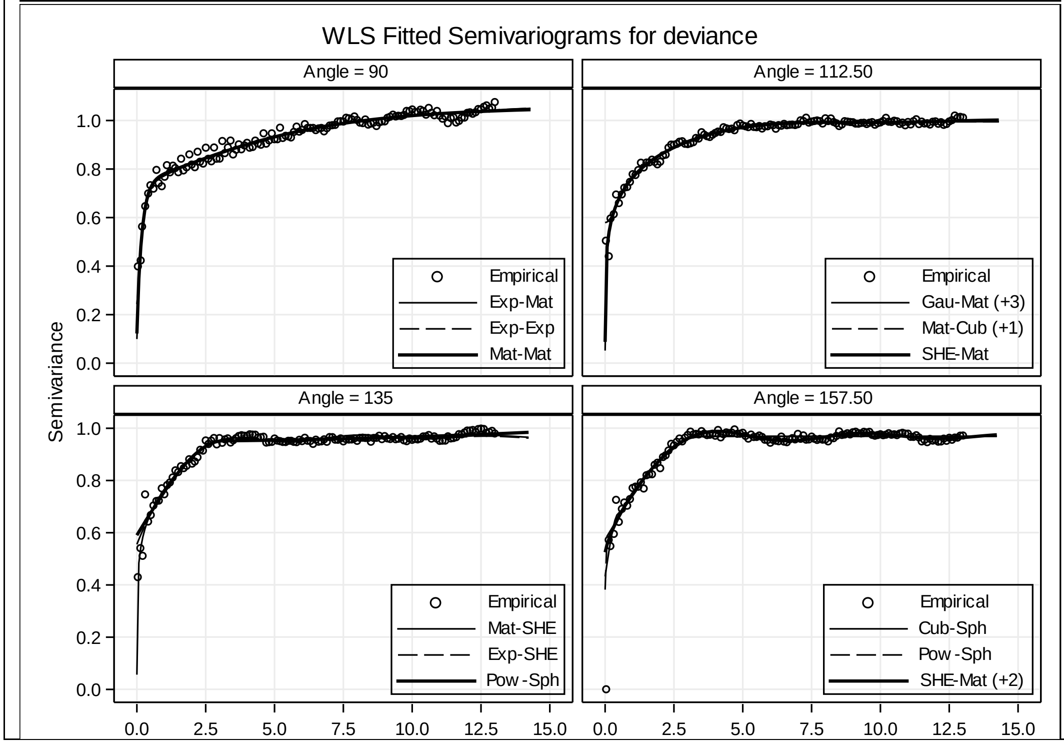

l.2) Sidewalk Present, 3rd order spatial detrend + rater adjustment

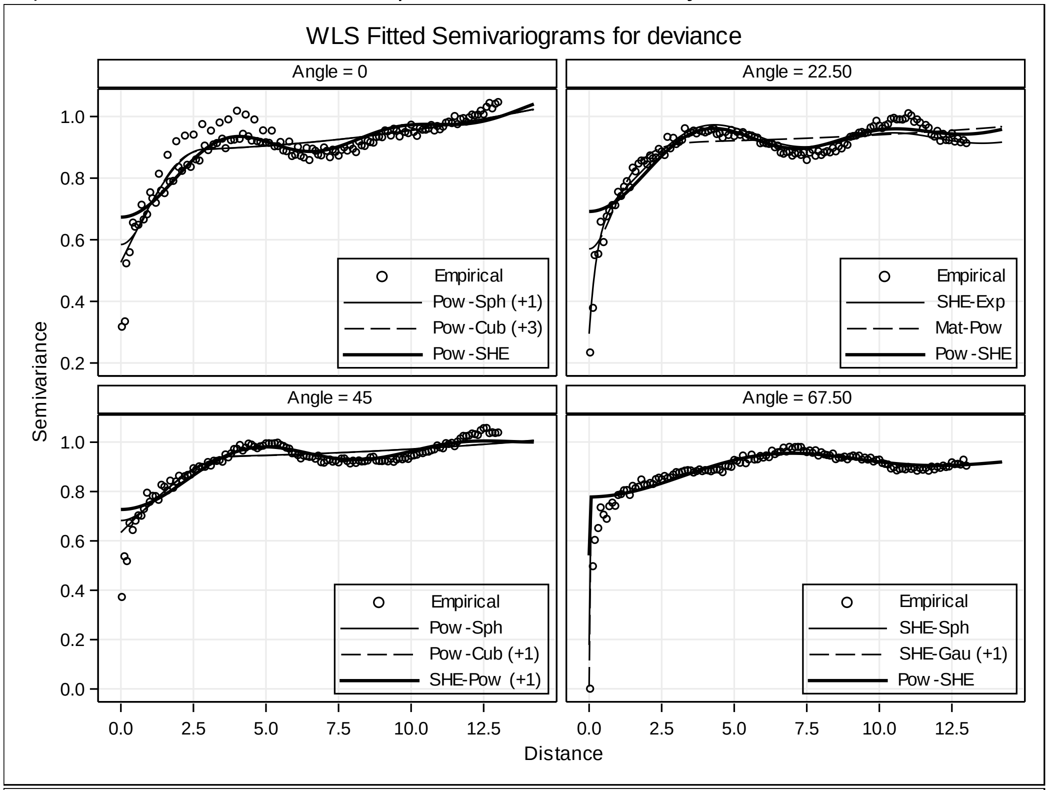

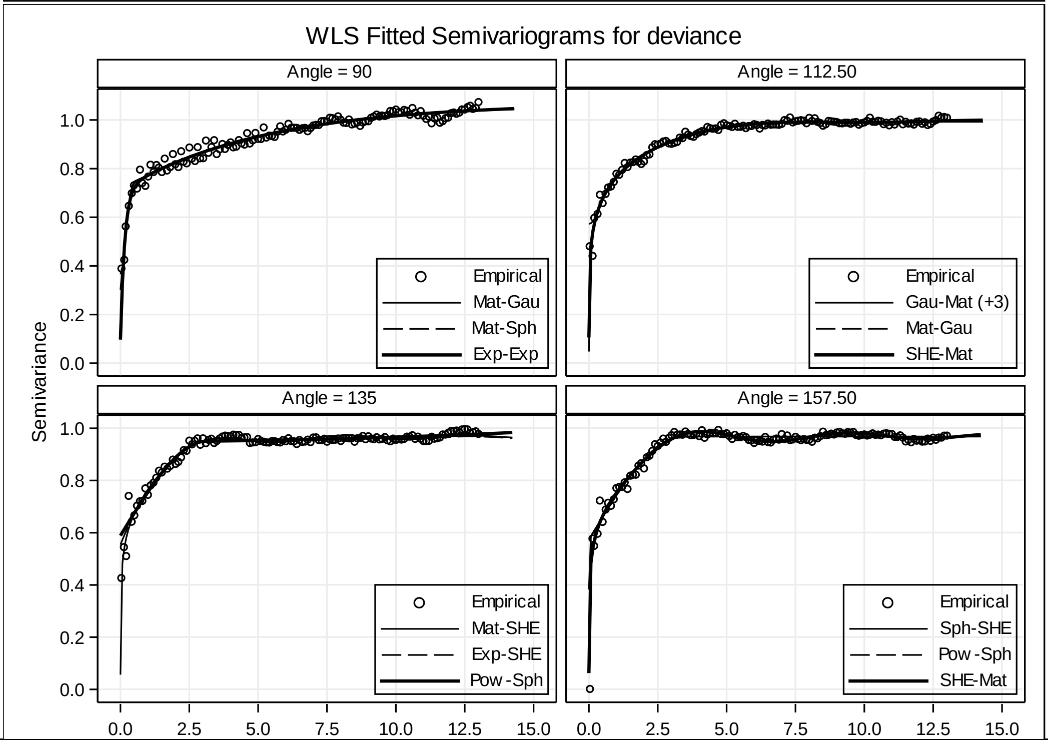

m.1) Sidewalk Complete, 3rd order spatial detrend

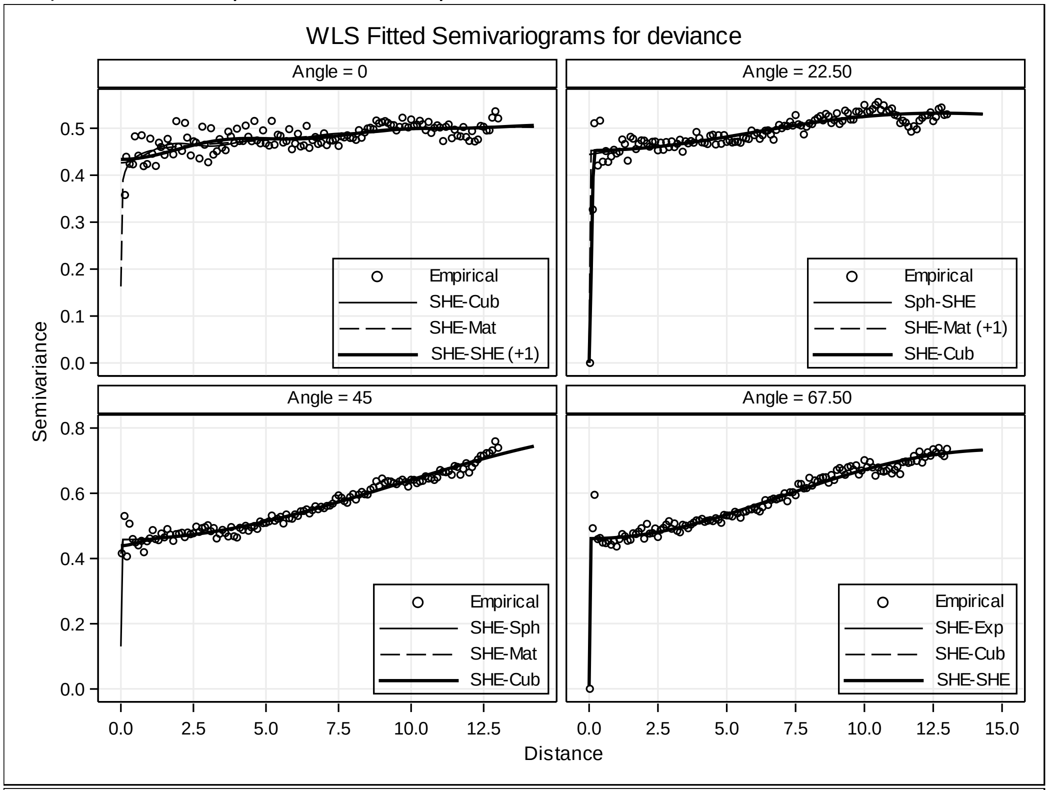

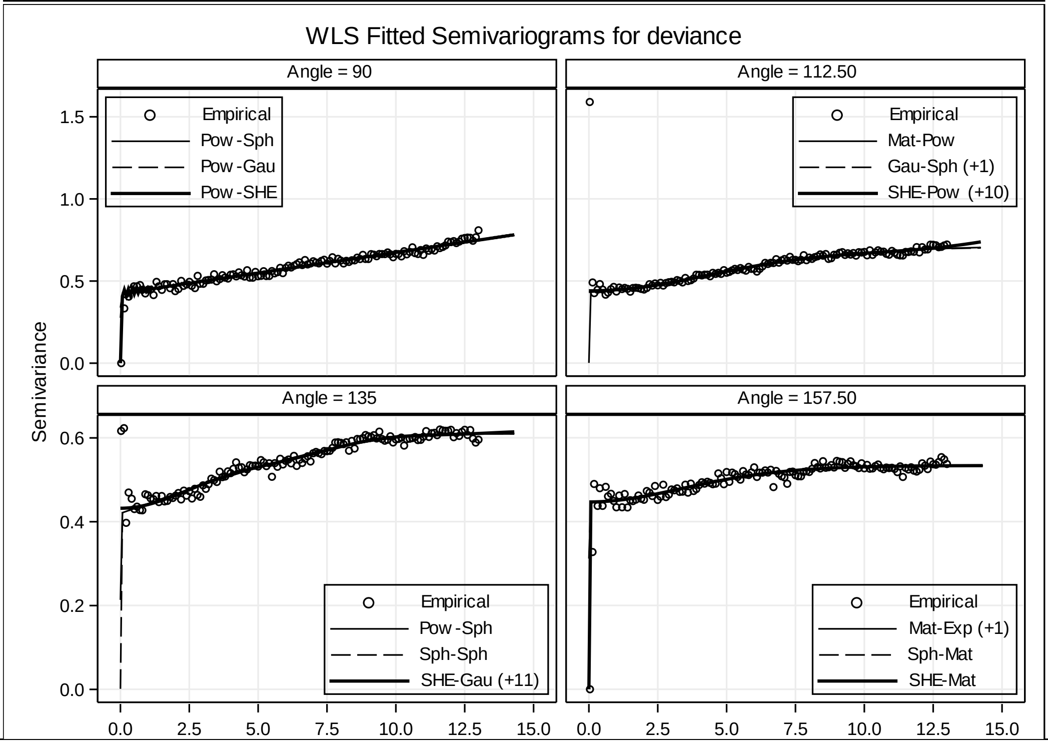

m.2) Sidewalk Complete, 3rd order spatial detrend + rater adjustment

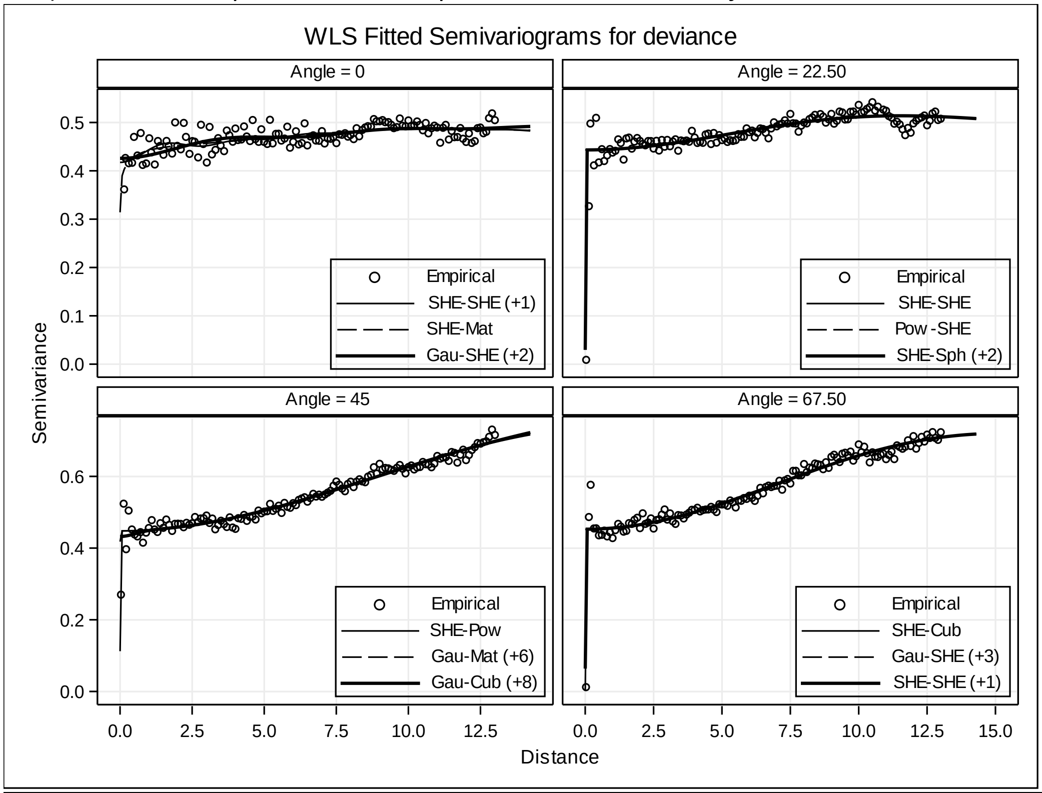

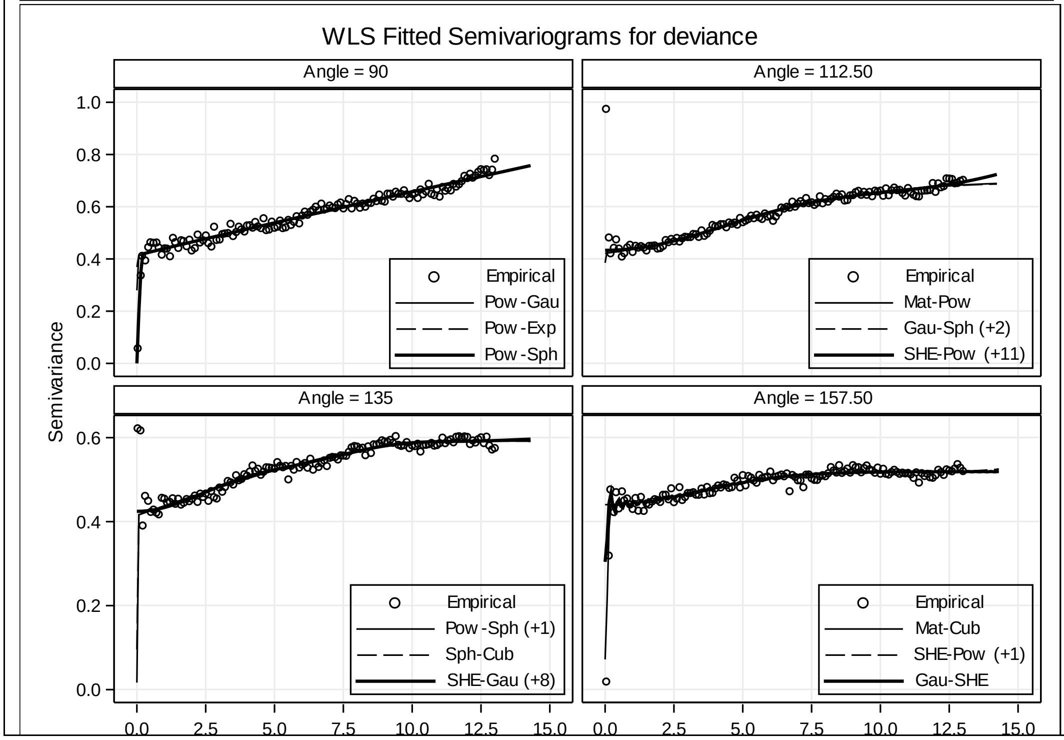

n.1) Sidewalk Condition, 3rd order spatial detrend

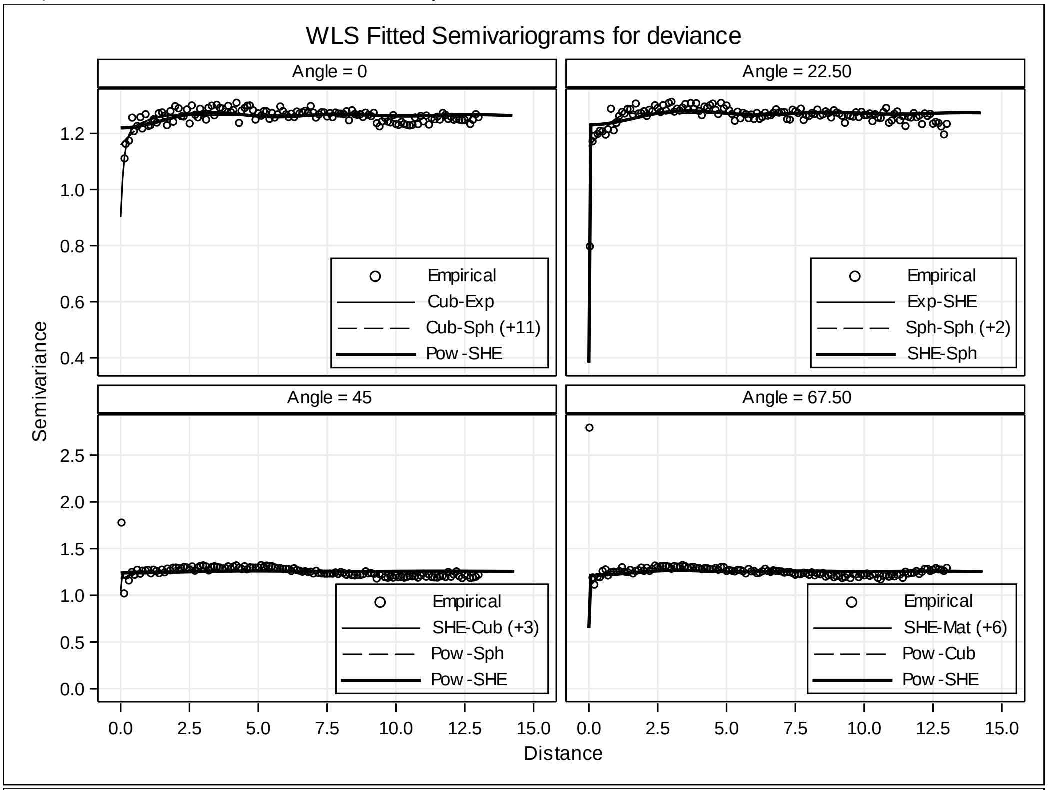

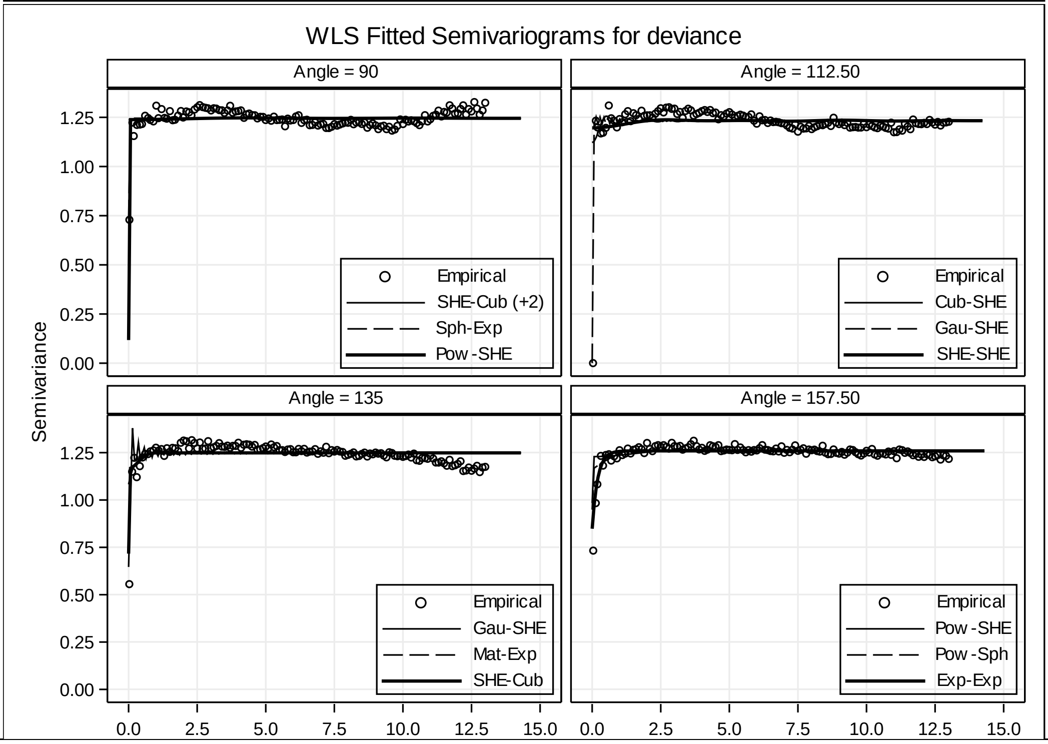

n.2) Sidewalk Condition, 3rd order spatial detrend + rater adjustment

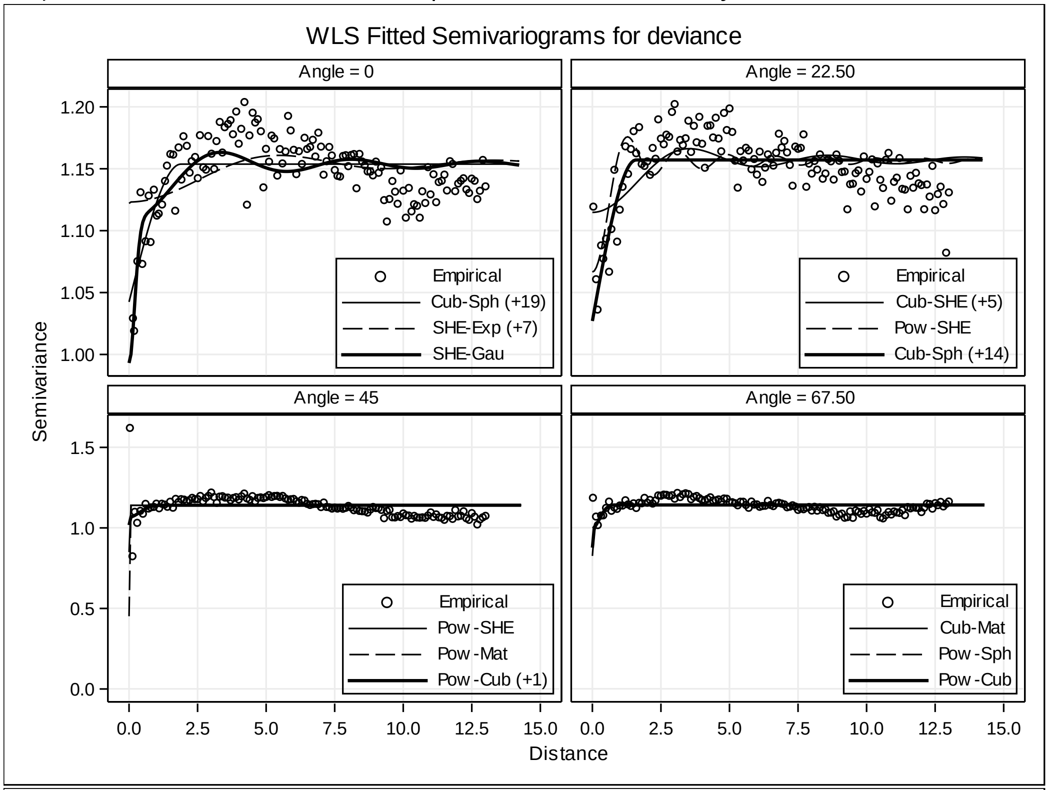

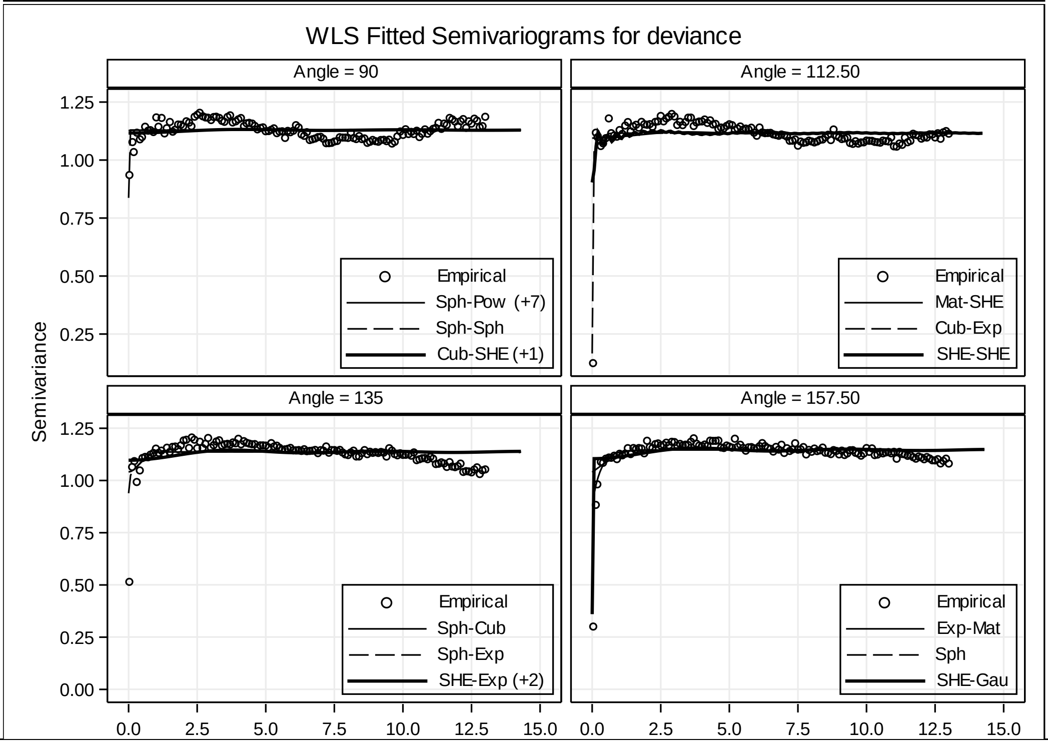

o.1) Sidewalk Width, 3rd order spatial detrend

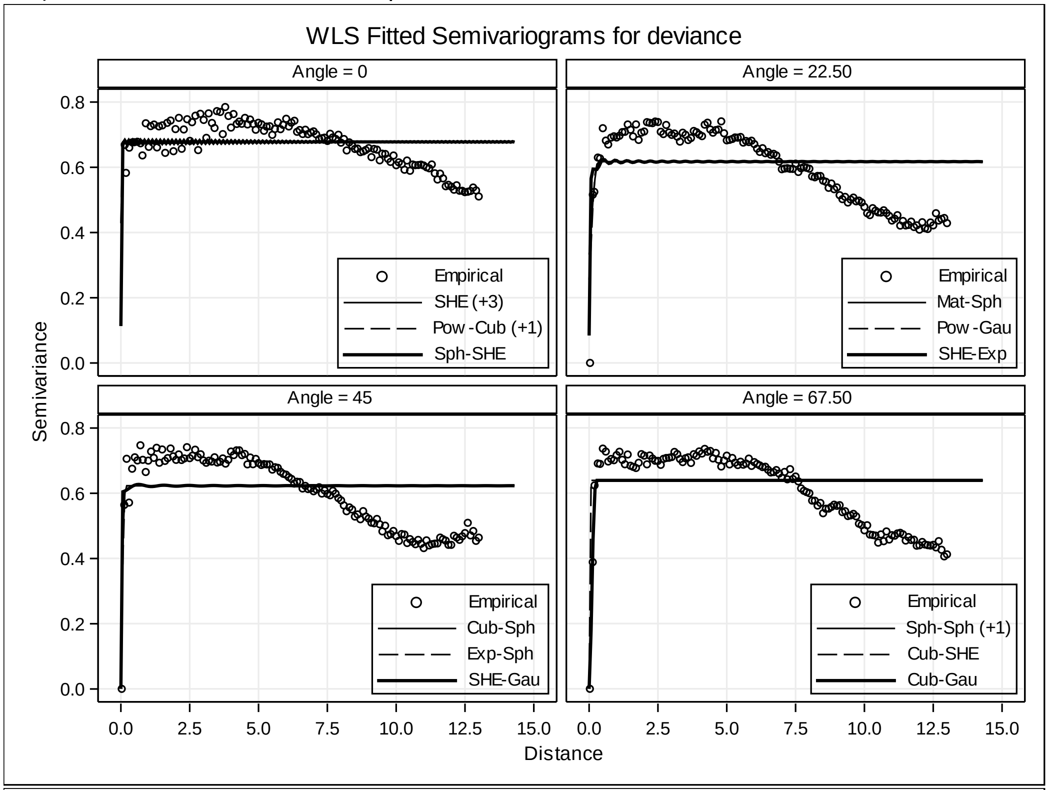

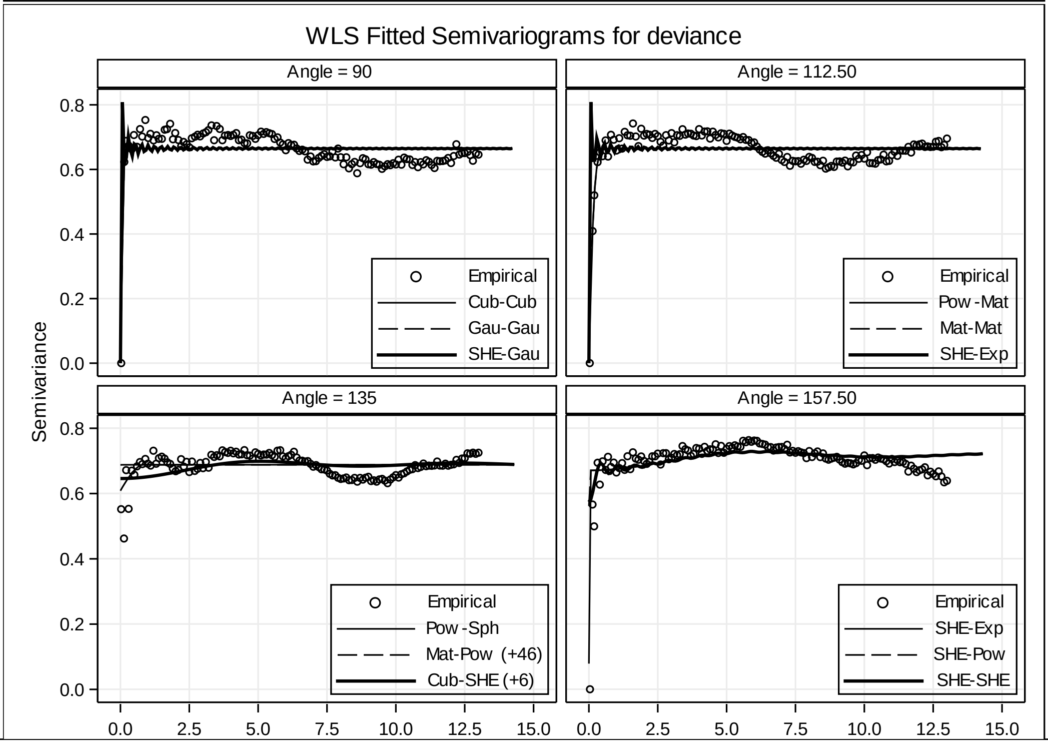

o.2) Sidewalk Width, 3rd order spatial detrend + rater adjustment

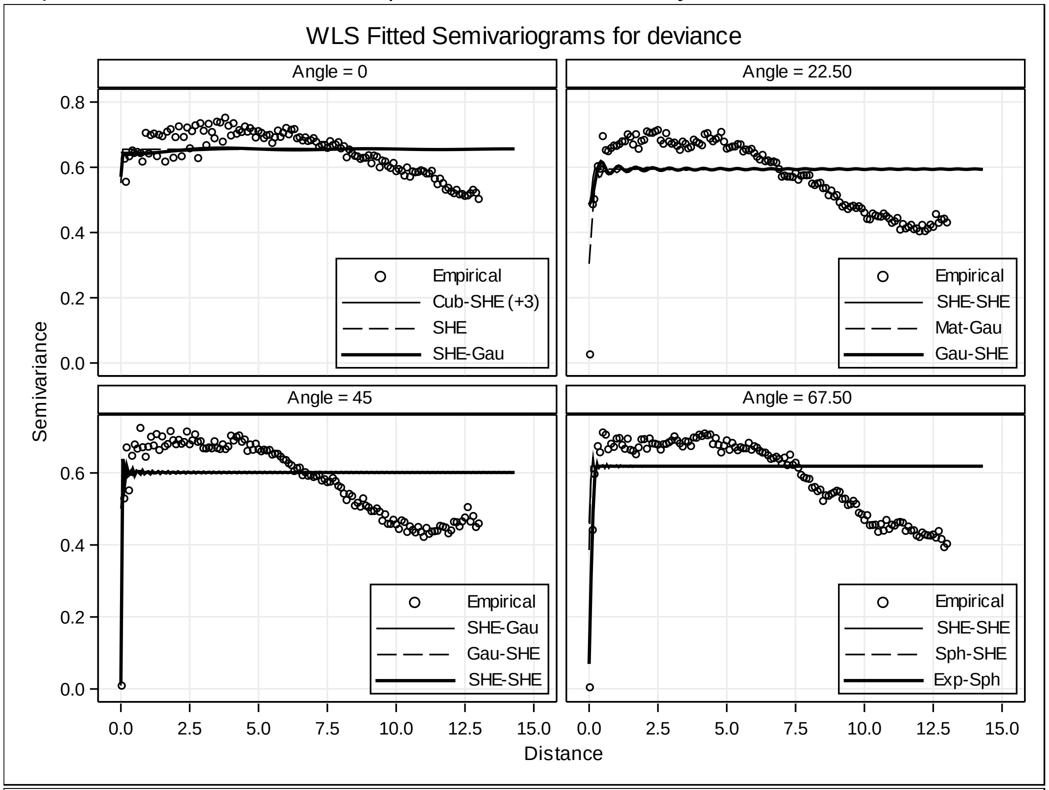

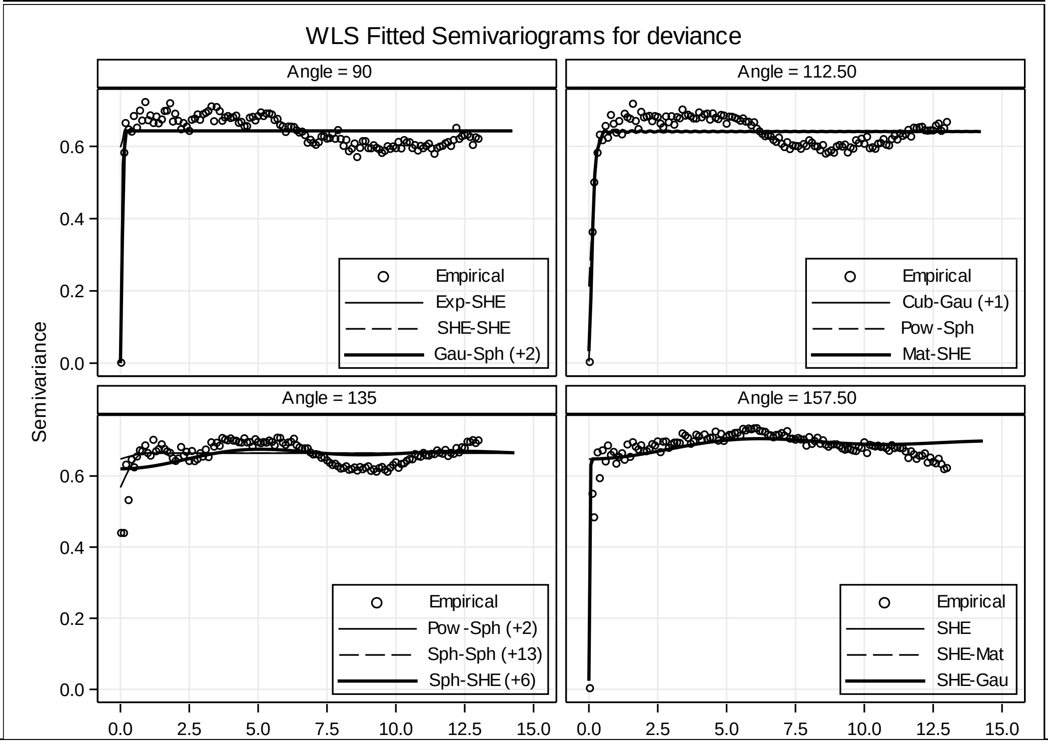

p.1) Sidewalk to Curb Distance, 3rd order spatial detrend

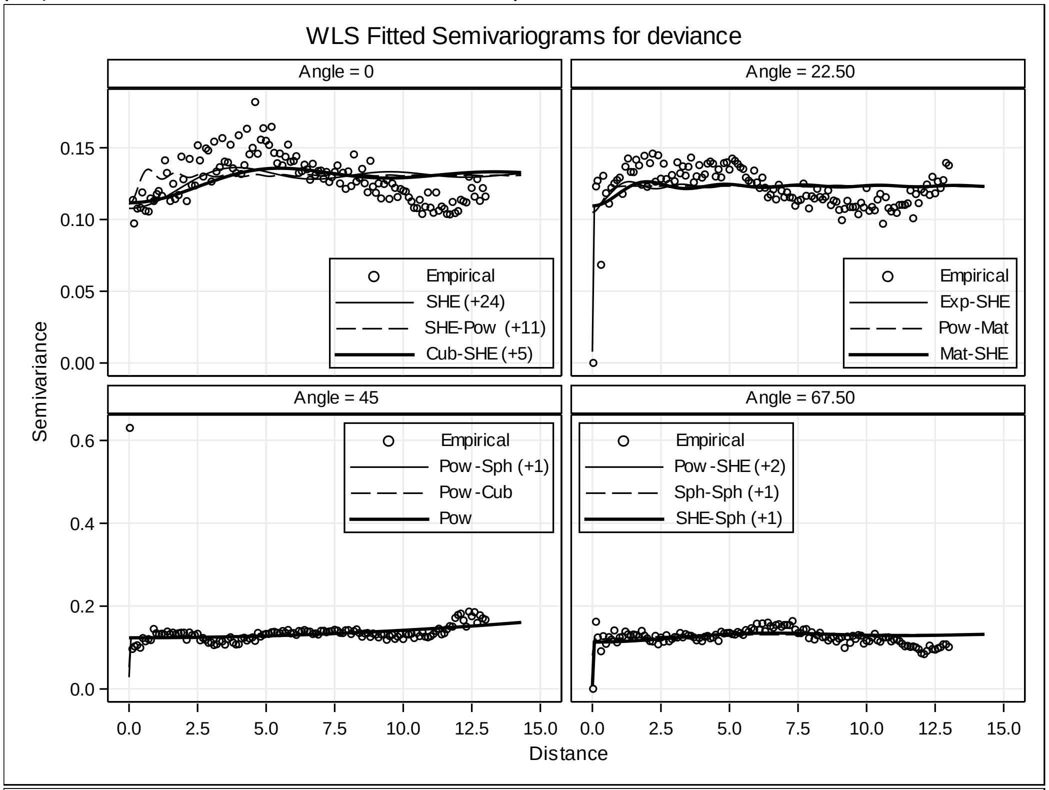

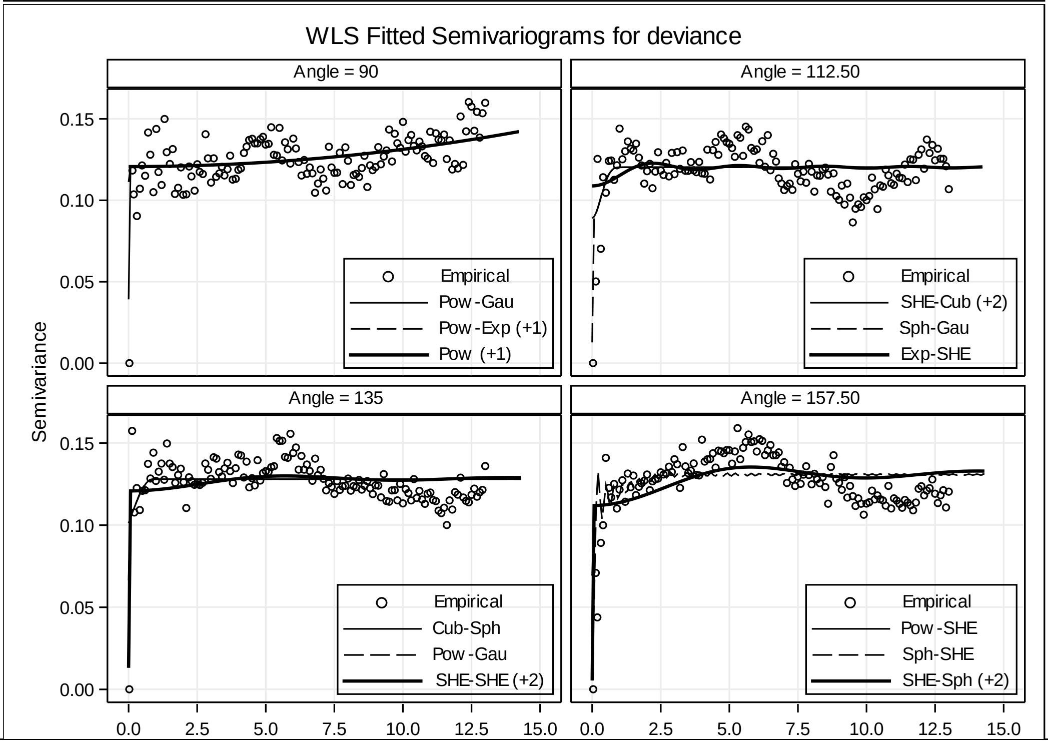

p.2) Sidewalk to Curb Distance, 3rd order spatial detrend + rater adjustment

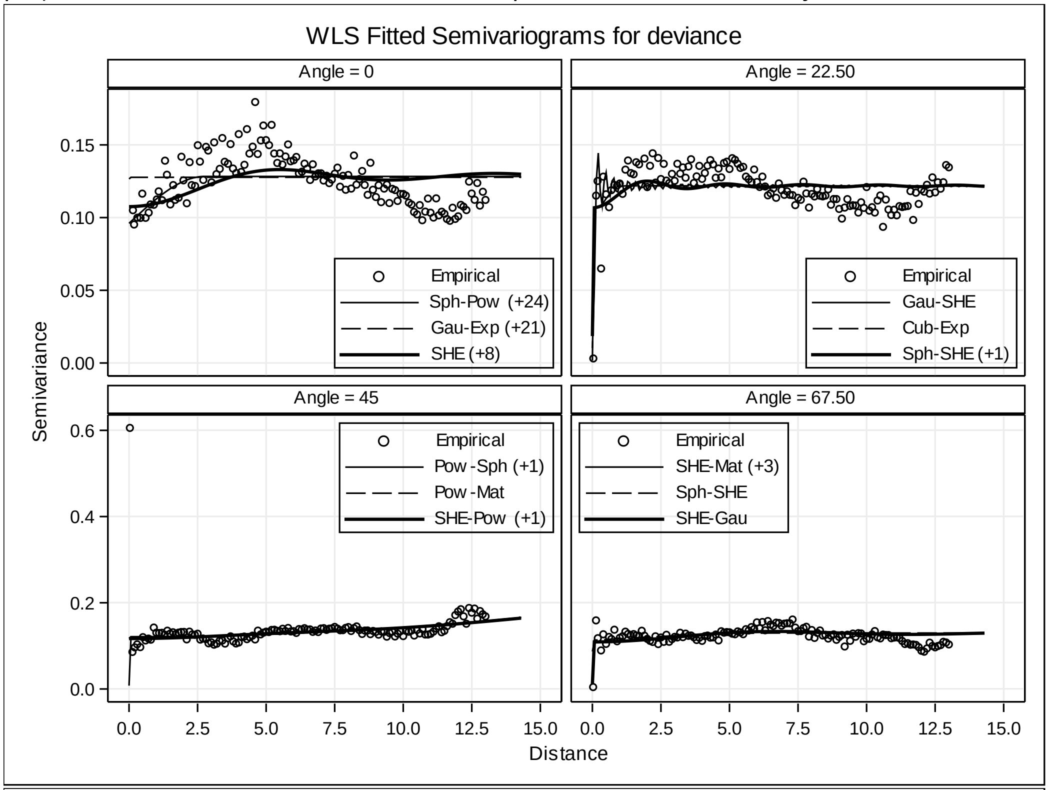

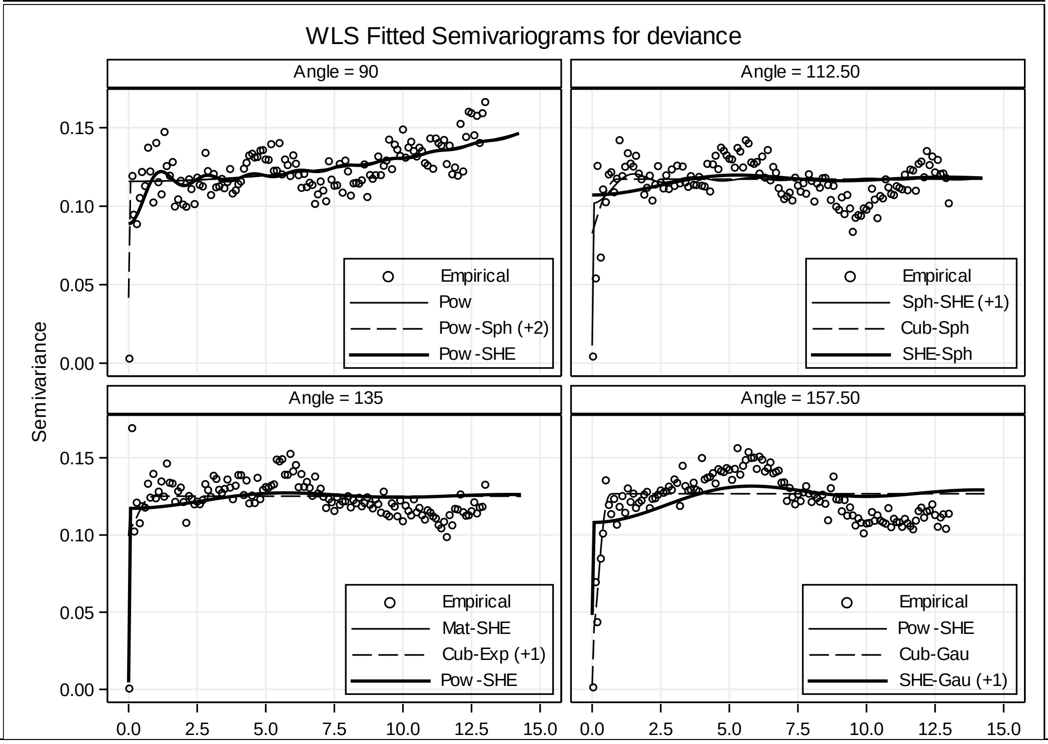

q.1) Car Obstruction, 3rd order spatial detrend

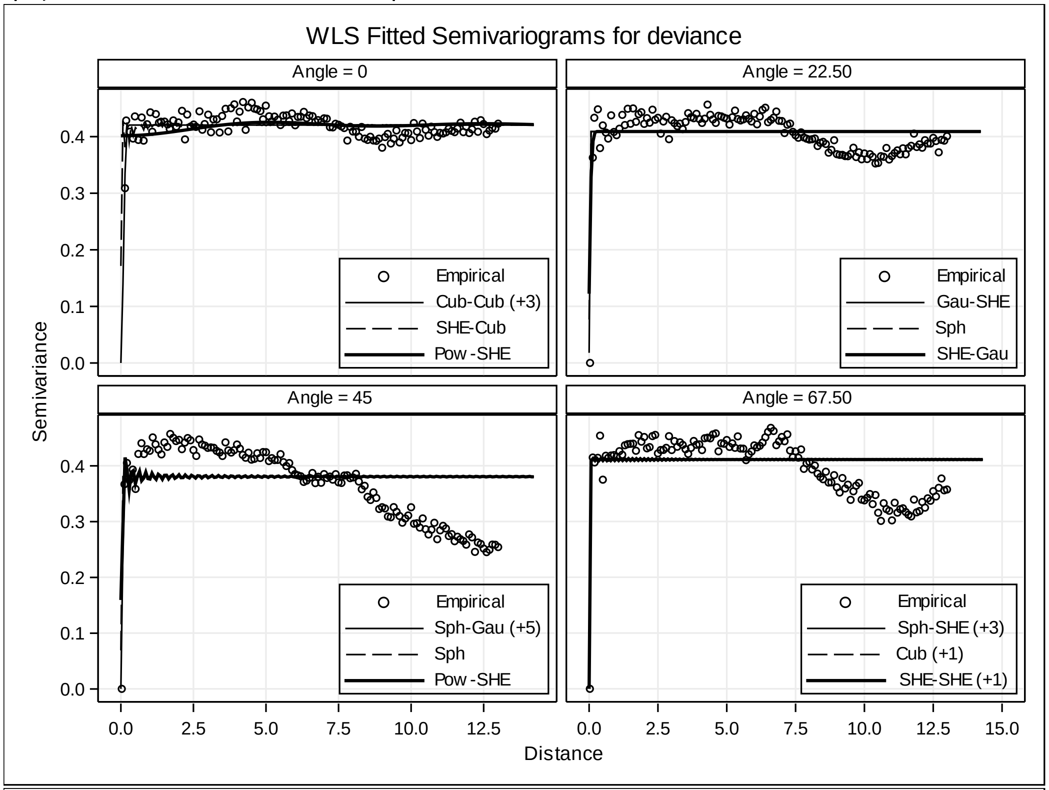

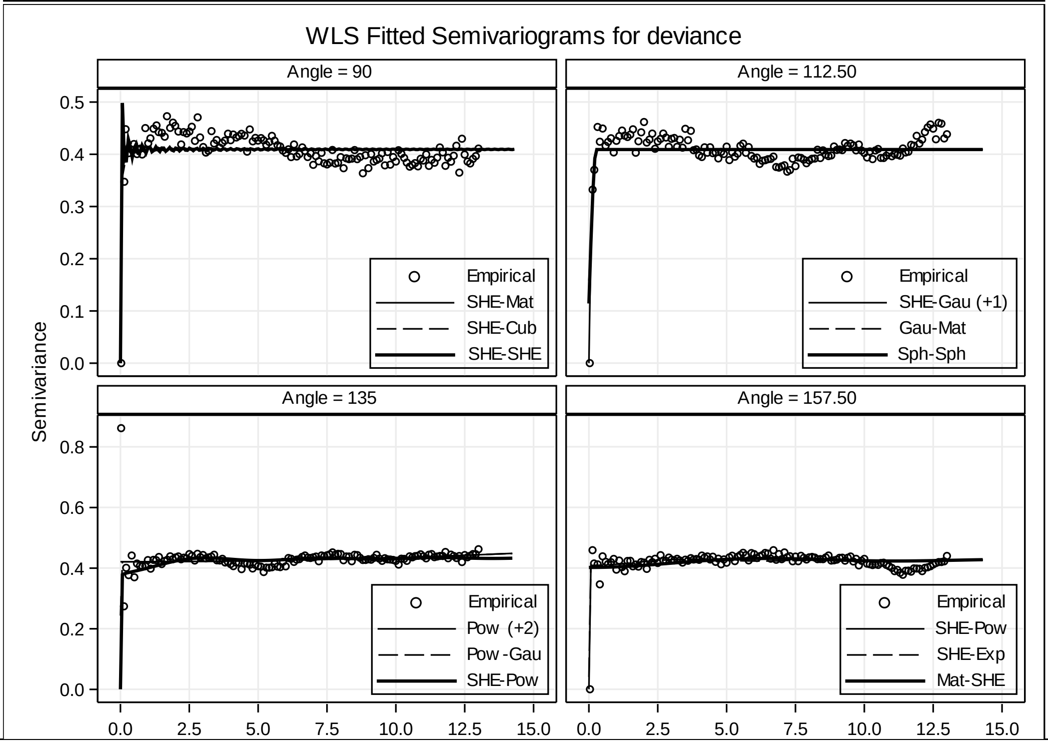

q.2) Car Obstruction, 3rd order spatial detrend + rater adjustment

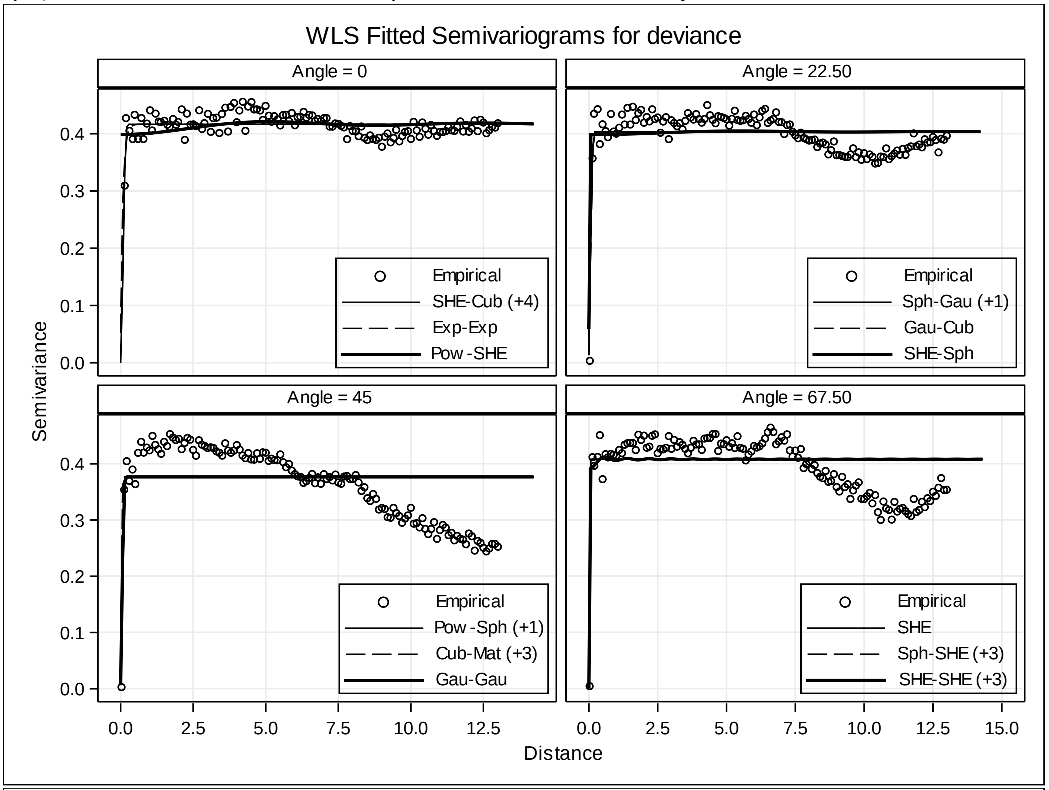

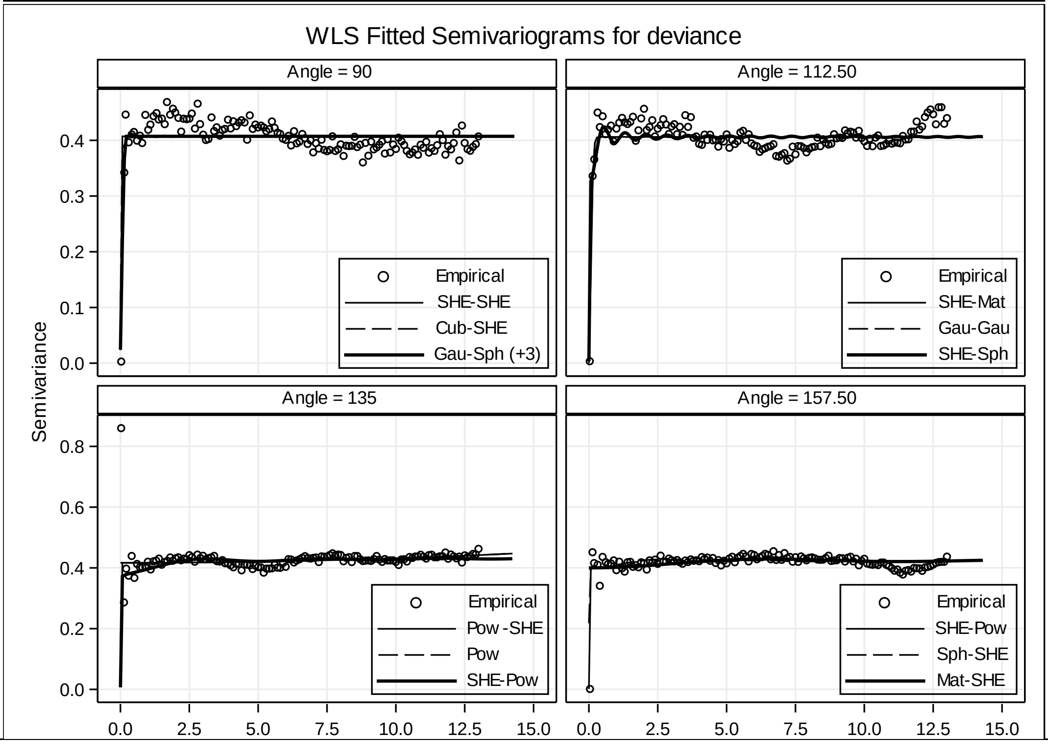

r.1) Garbage Can Obstruction, 3rd order spatial detrend

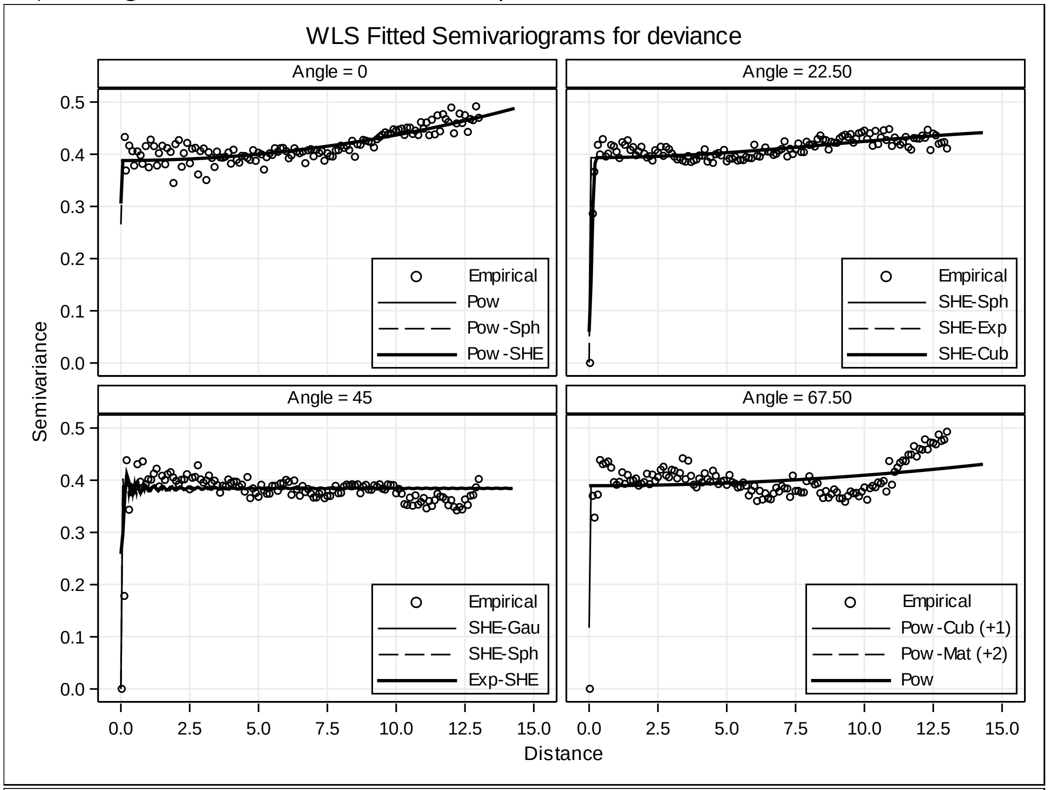

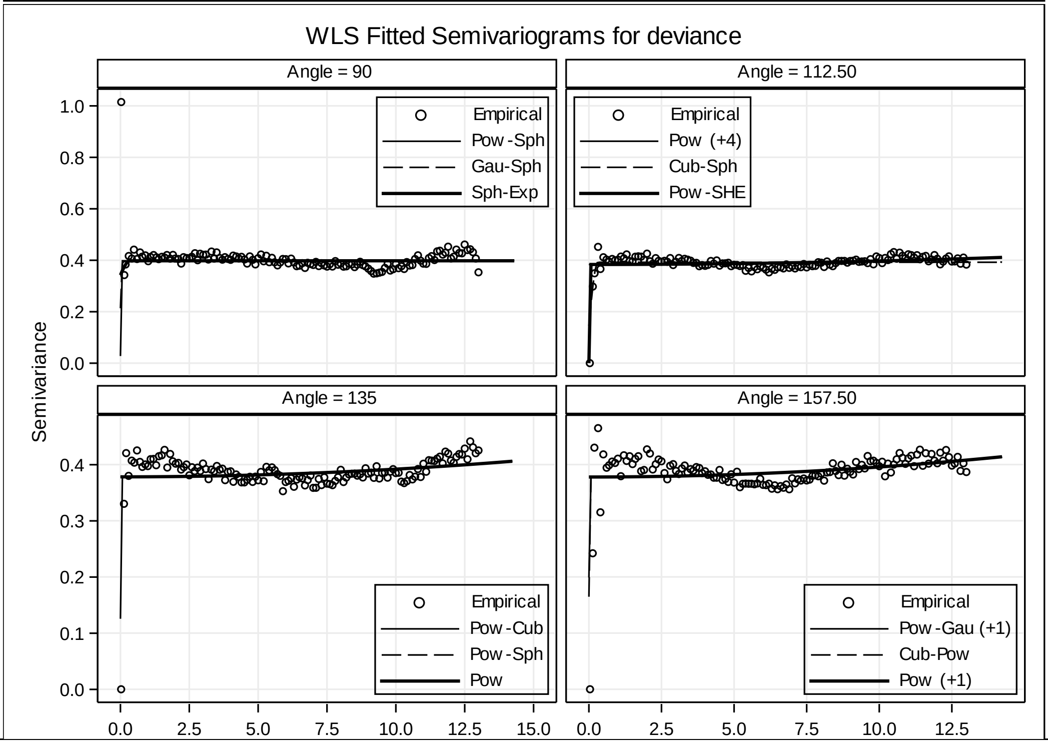

r.2) Garbage Can Obstruction, 3rd order spatial detrend + rater adjustment

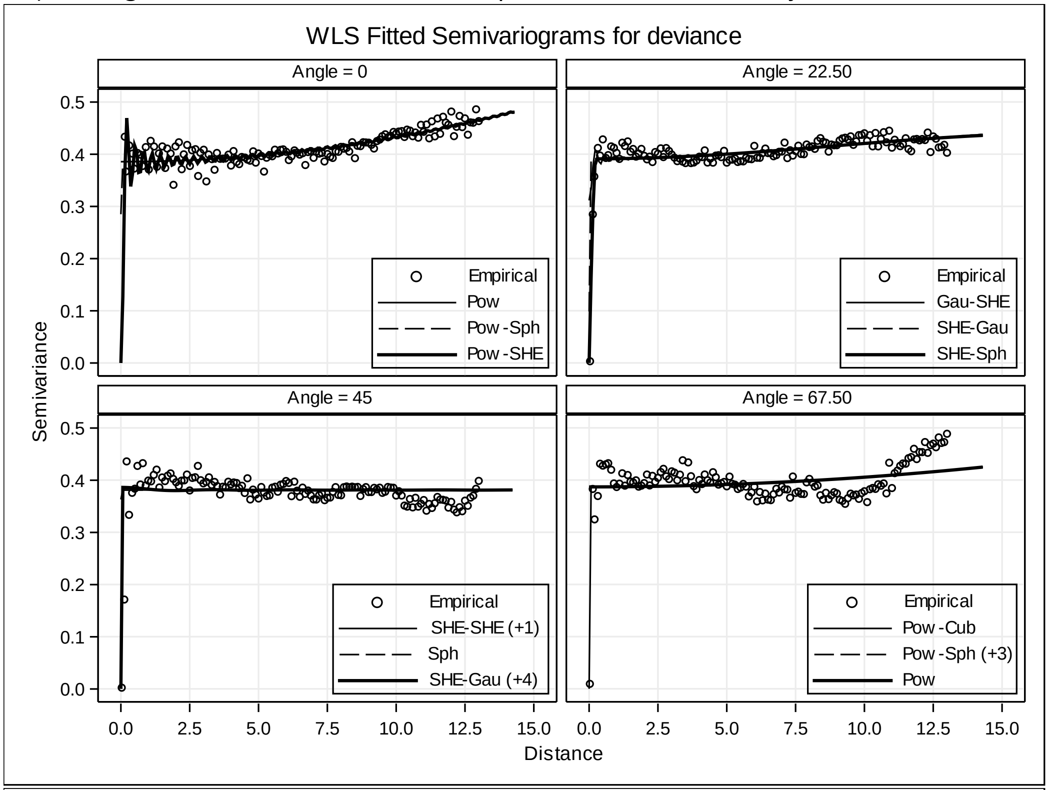

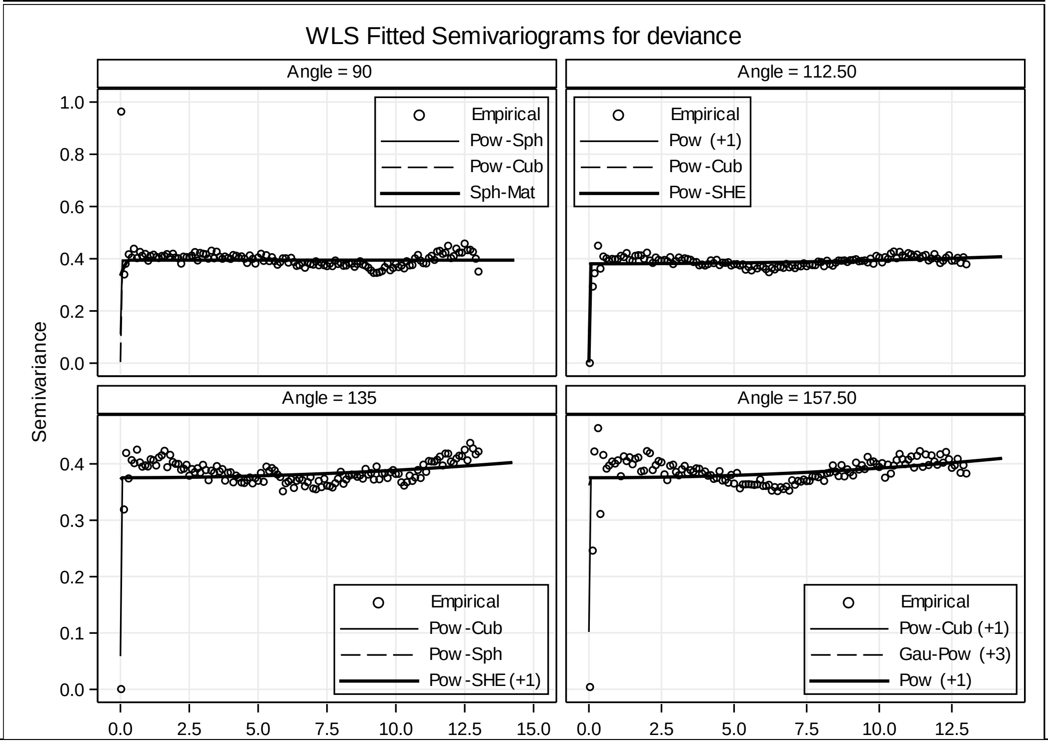

s.1) Pole/Sign Obstruction, 3rd order spatial detrend

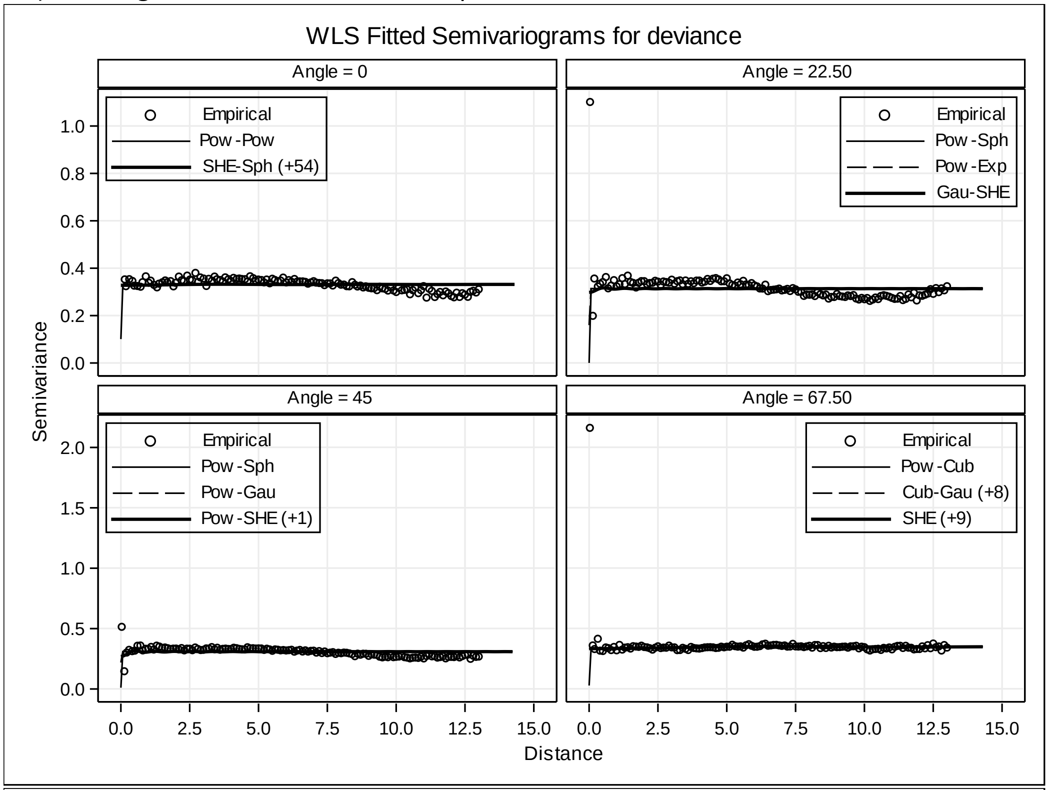

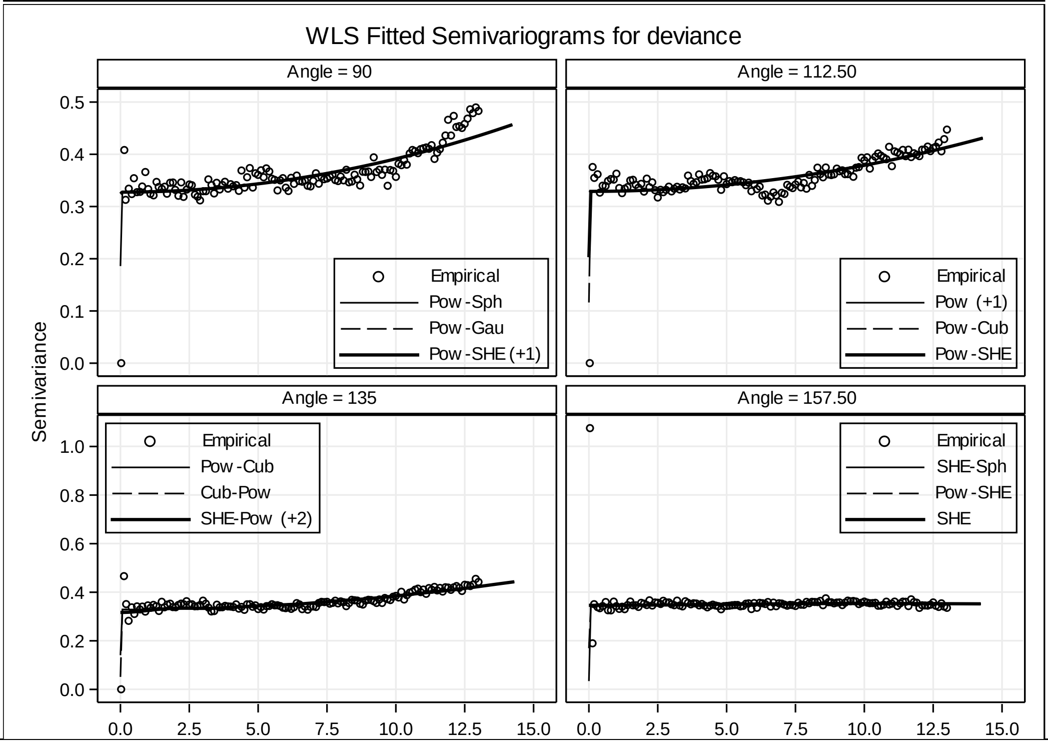

s.2) Pole/Sign Obstruction, 3rd order spatial detrend + rater adjustment

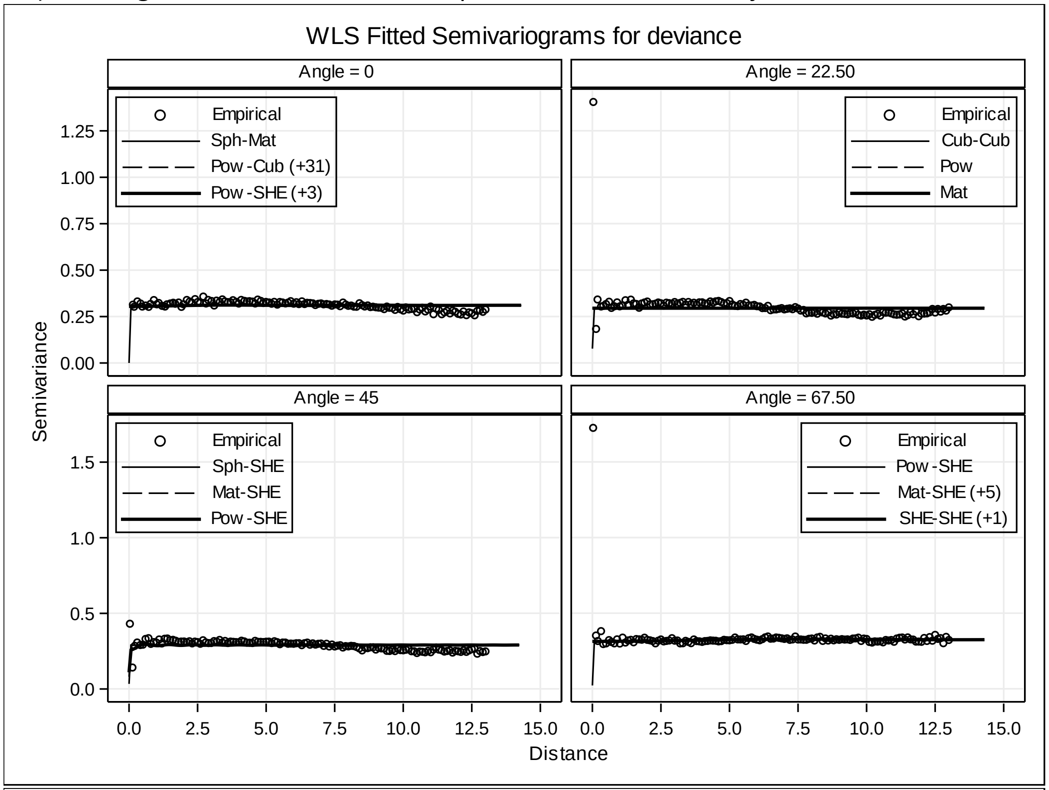

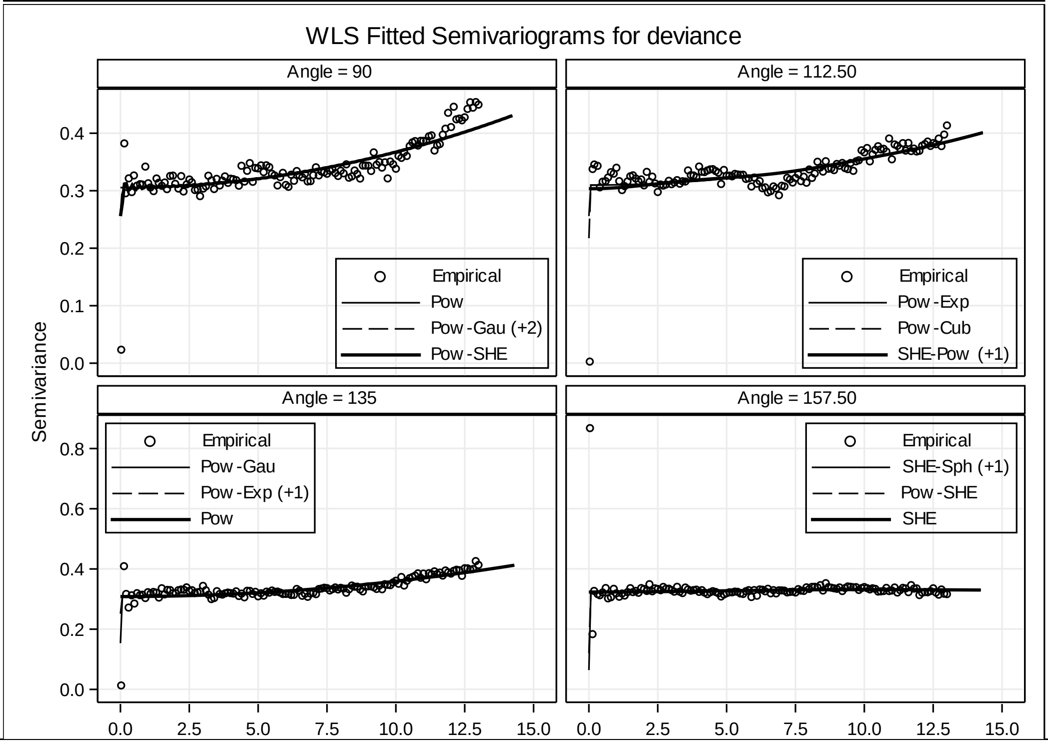

t.1) Other Obstruction, 3rd order spatial detrend

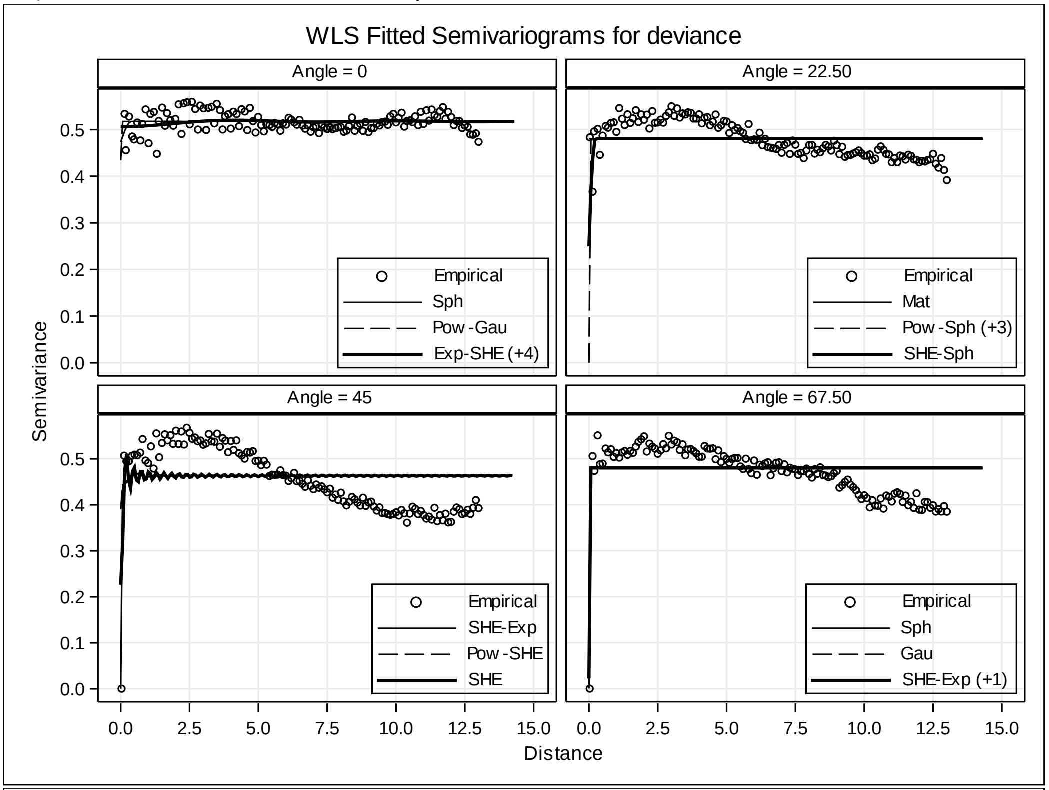

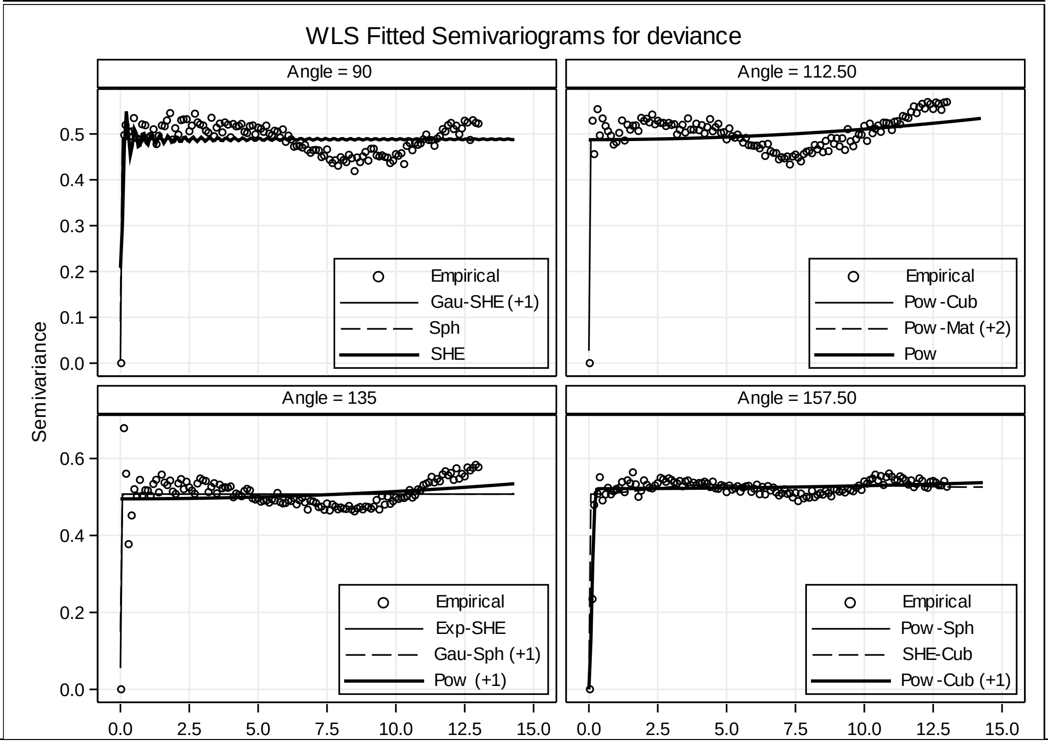

t.2) Other Obstruction, 3rd order spatial detrend + rater adjustment

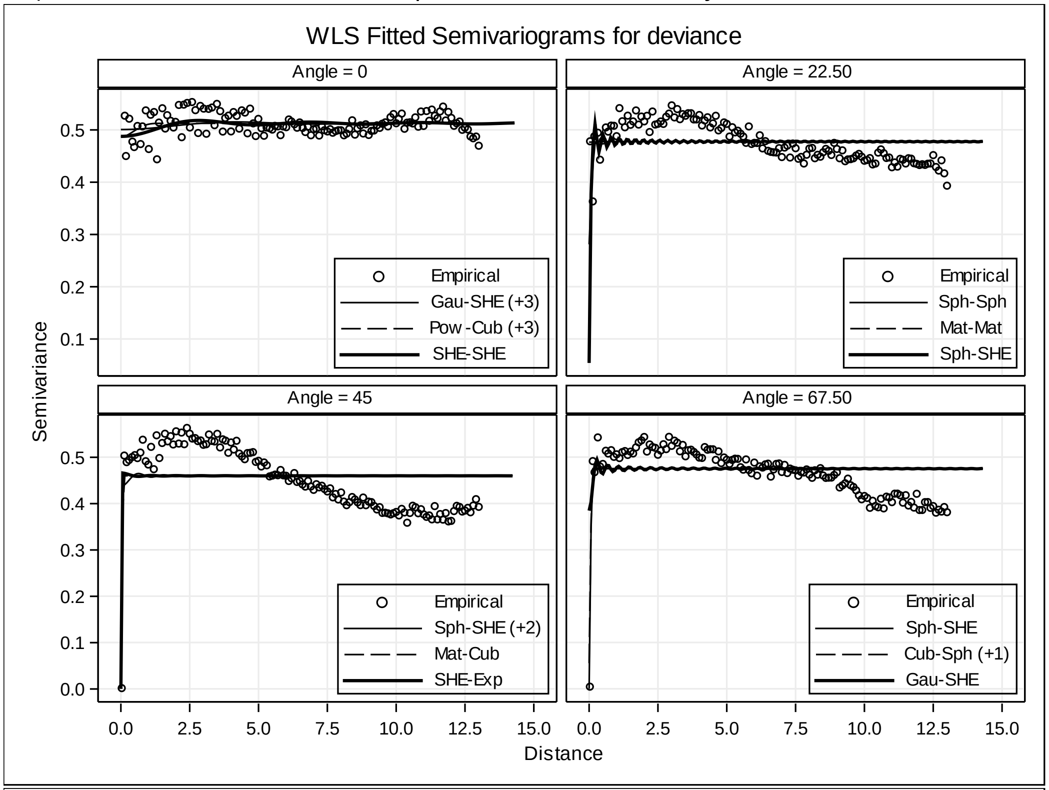

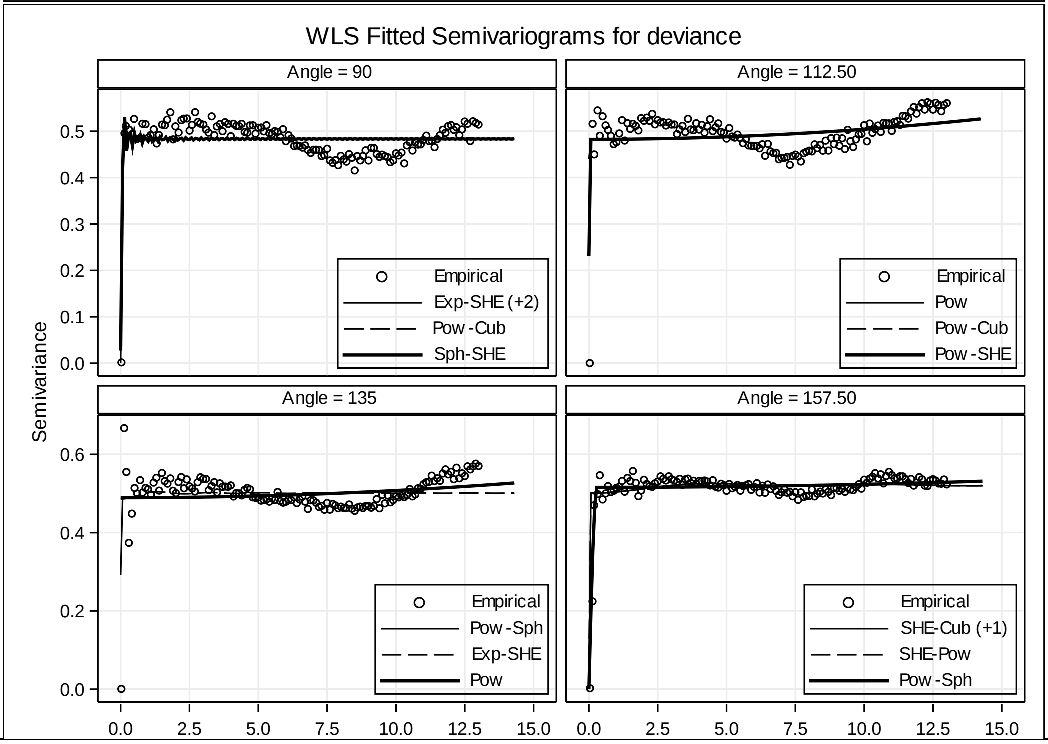

u.1) Curb Cuts, 3rd order spatial detrend

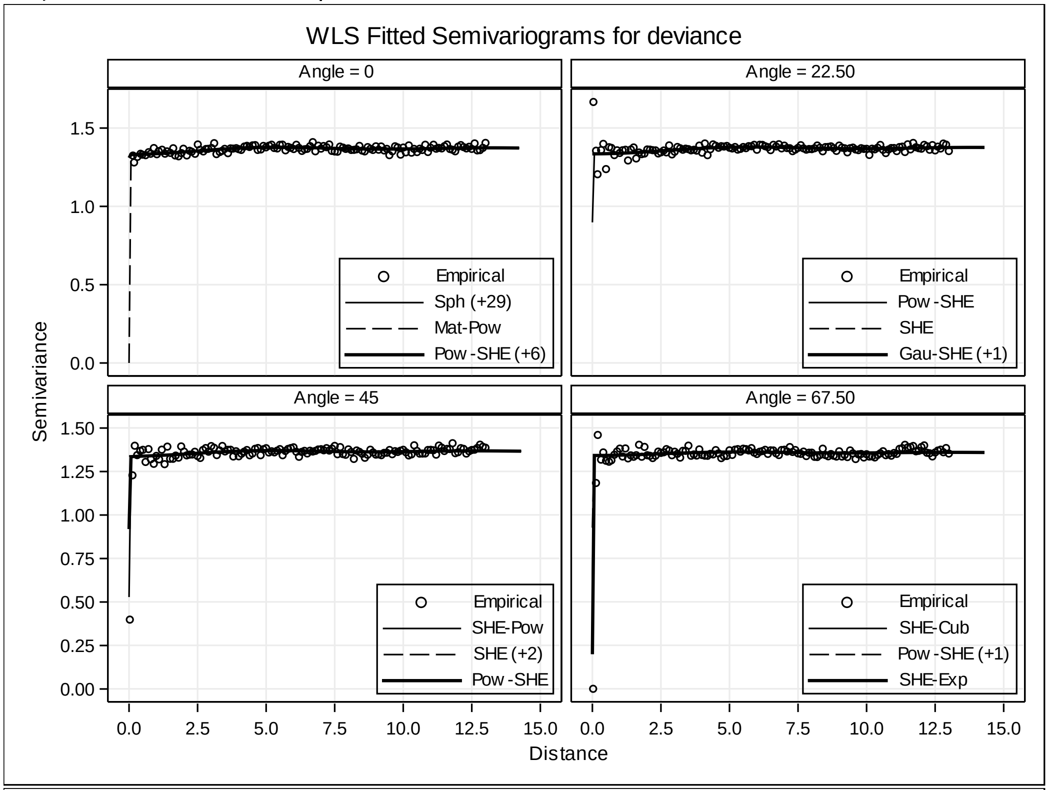

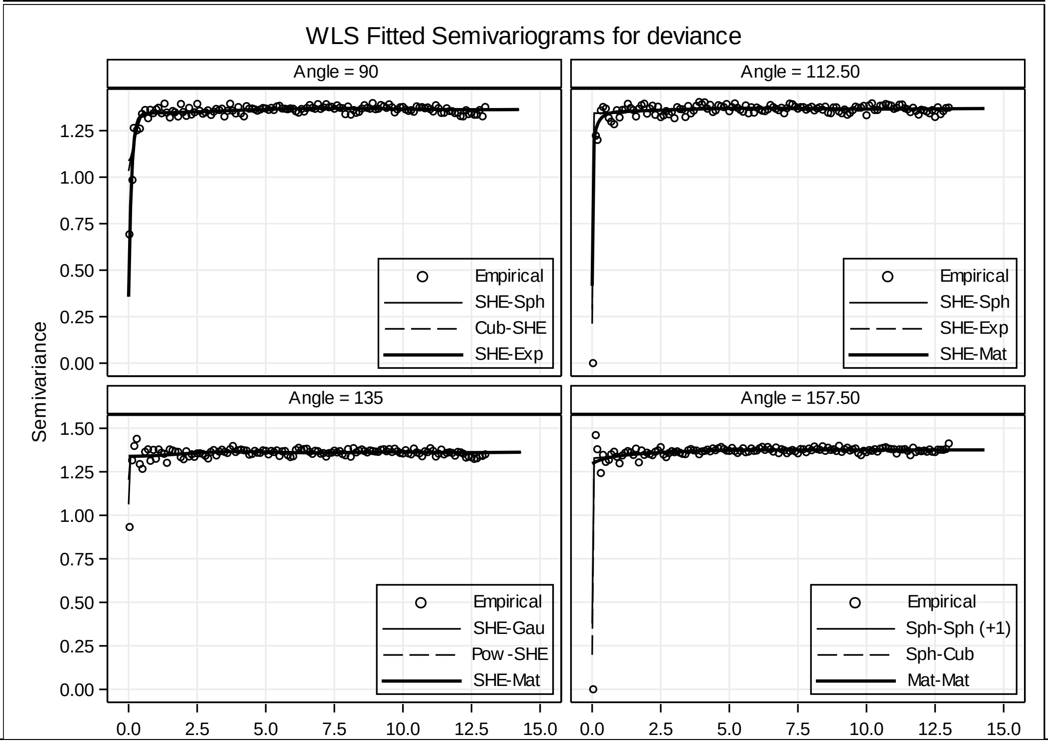

u.2) Curb Cuts, 3rd order spatial detrend + rater adjustment

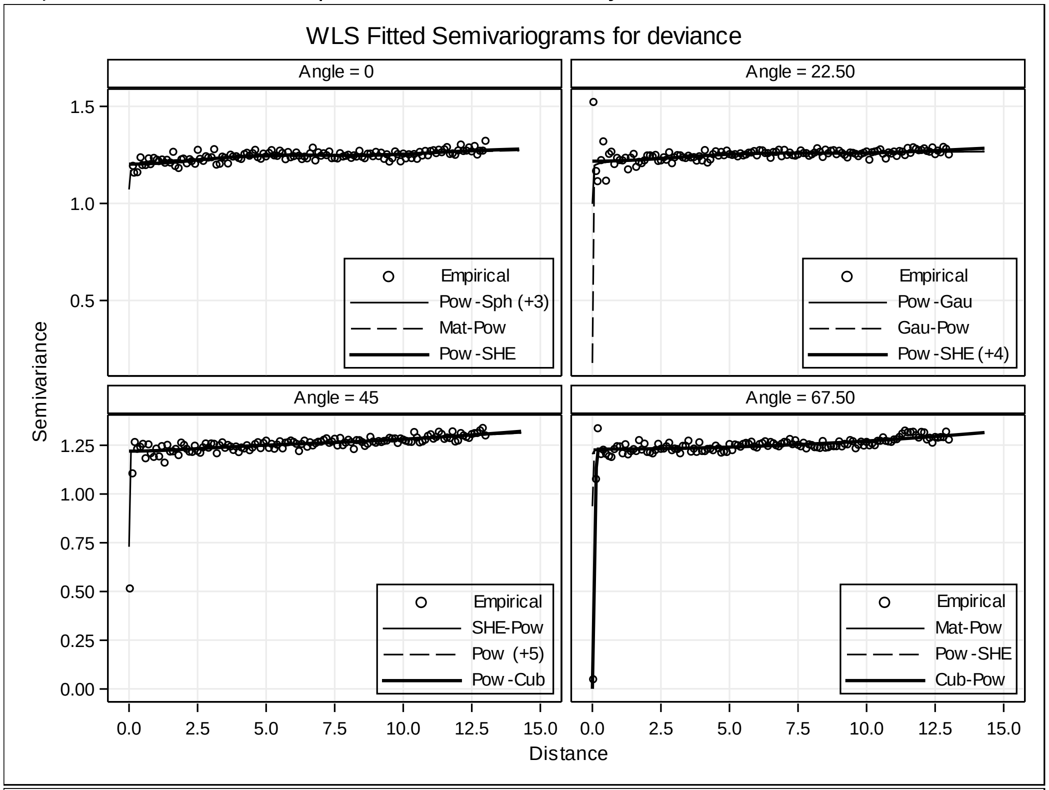

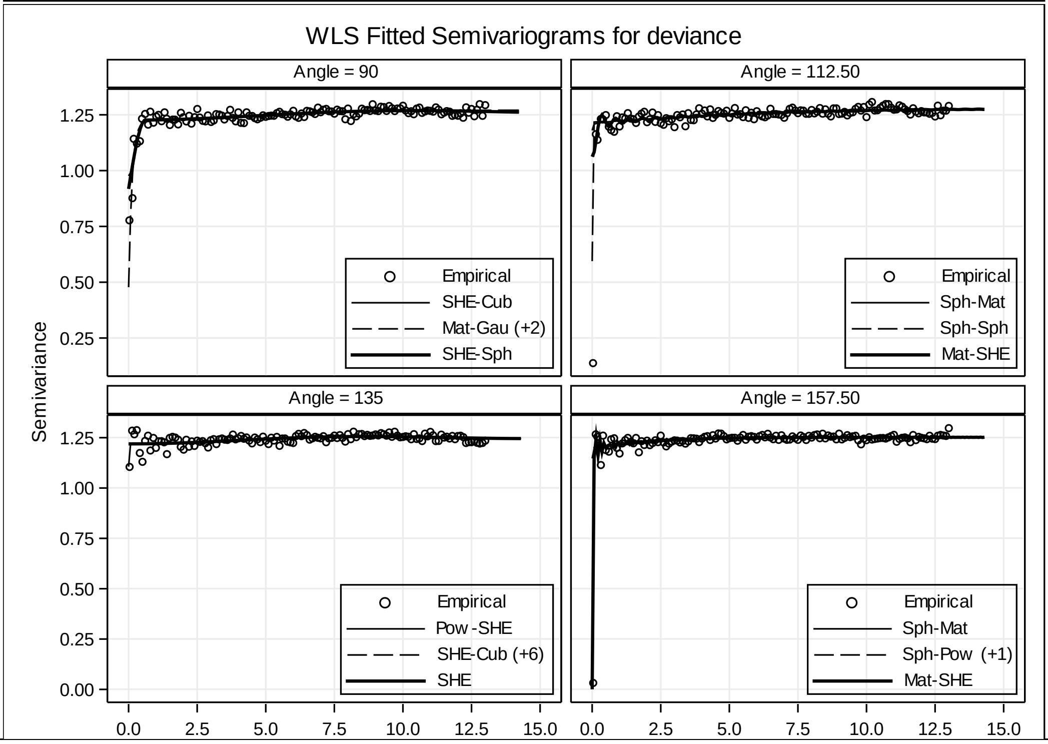

v.1) Clear Intersection, 3rd order spatial detrend

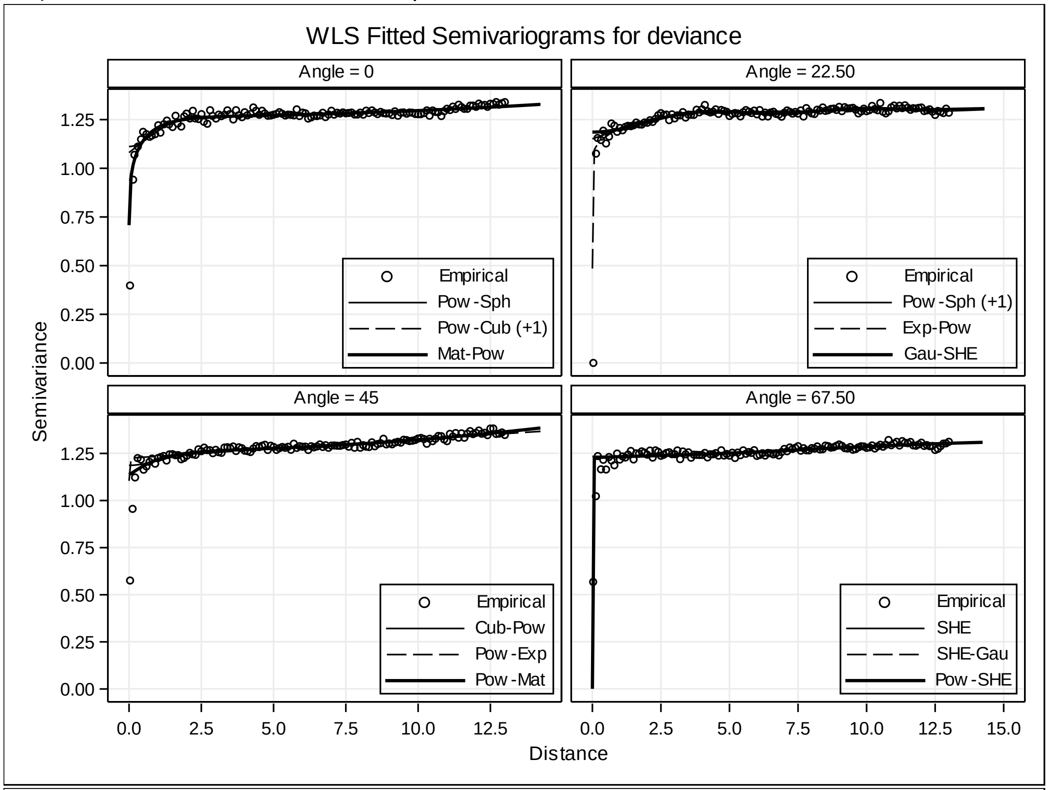

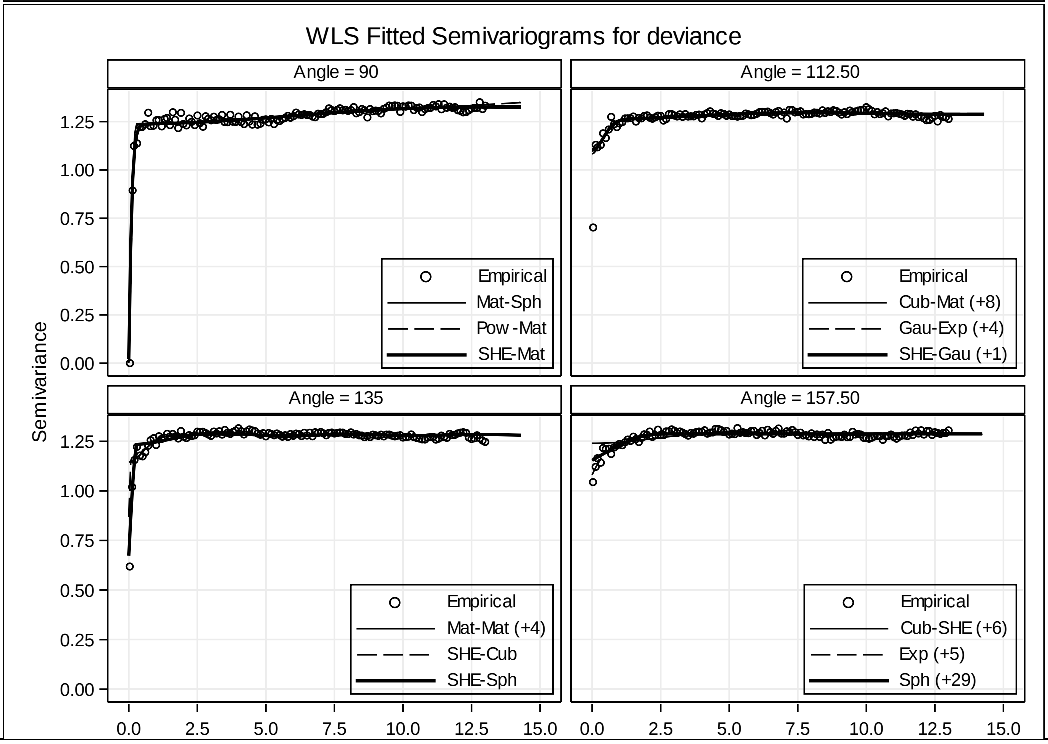

v.2) Clear Intersection, 3rd order spatial detrend + rater adjustment

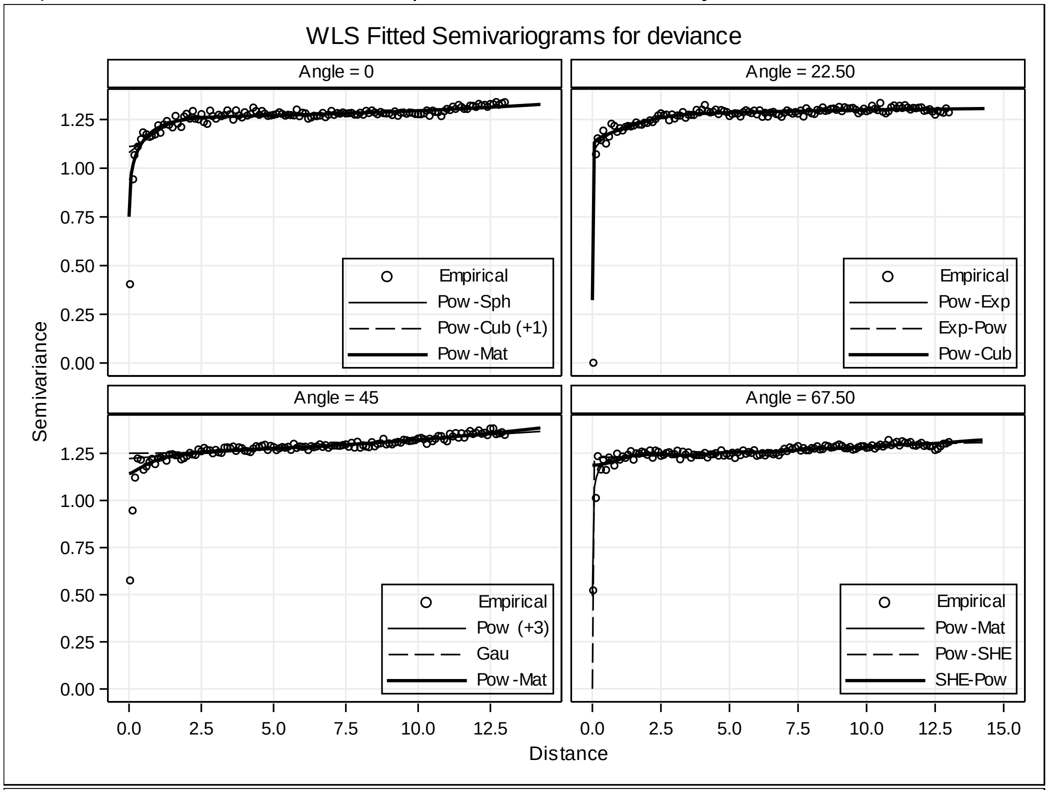

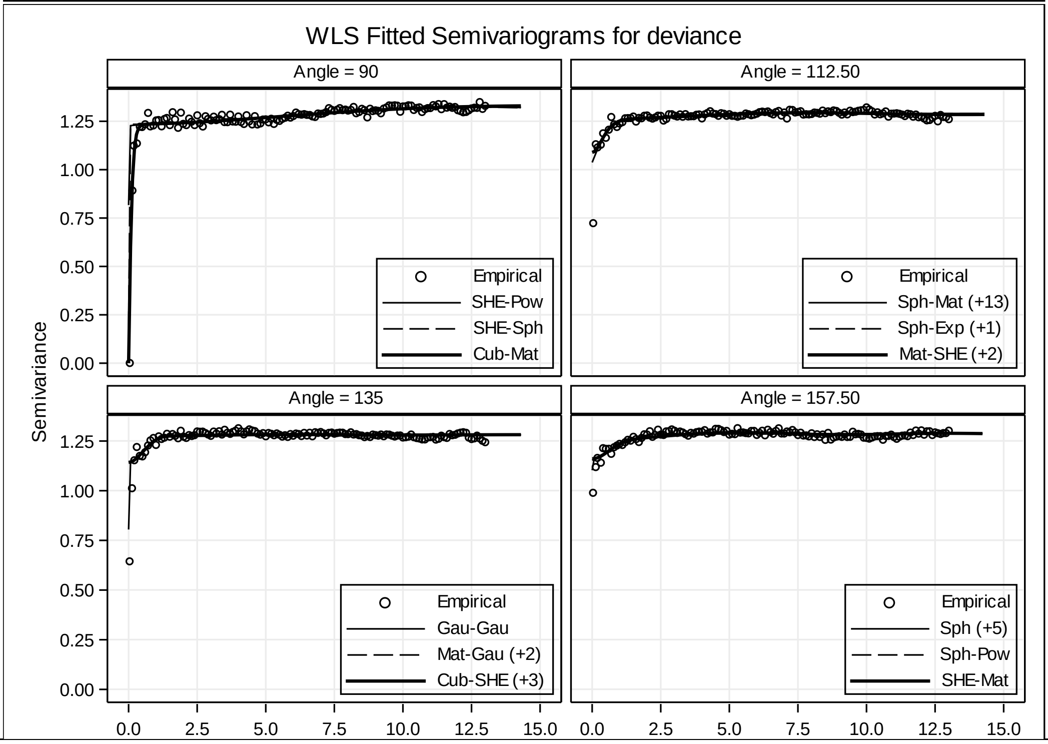

w.1) Pedestrian Crossing Sign, 3rd order spatial detrend

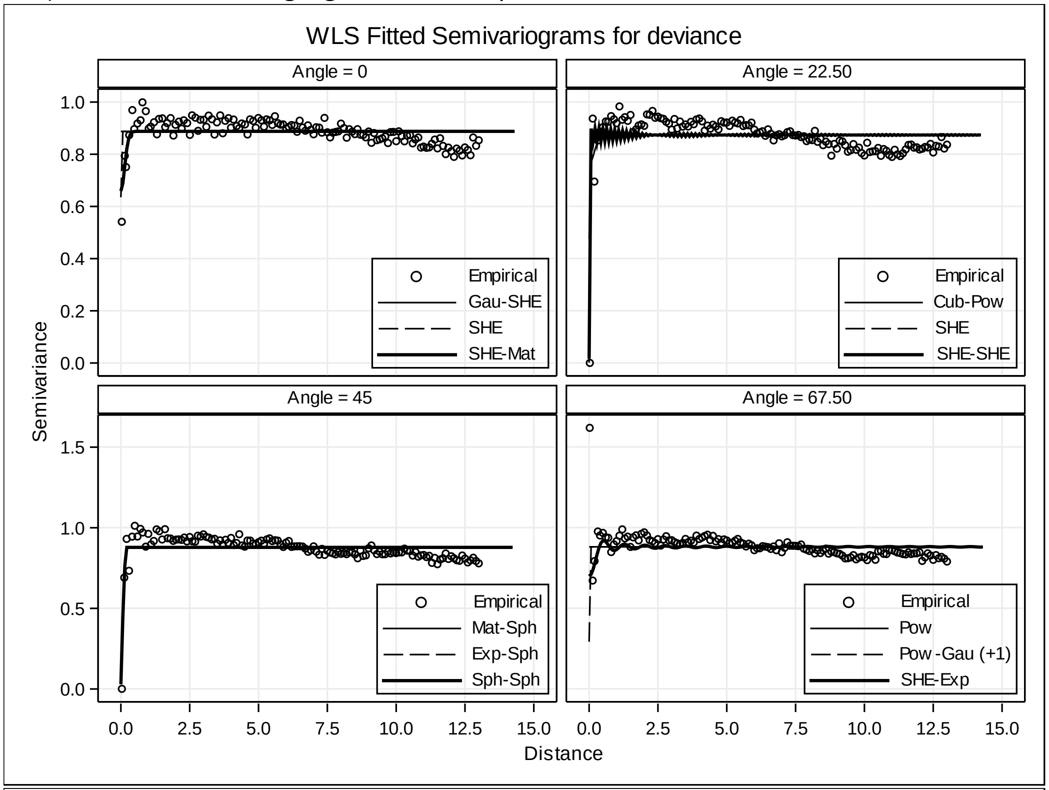

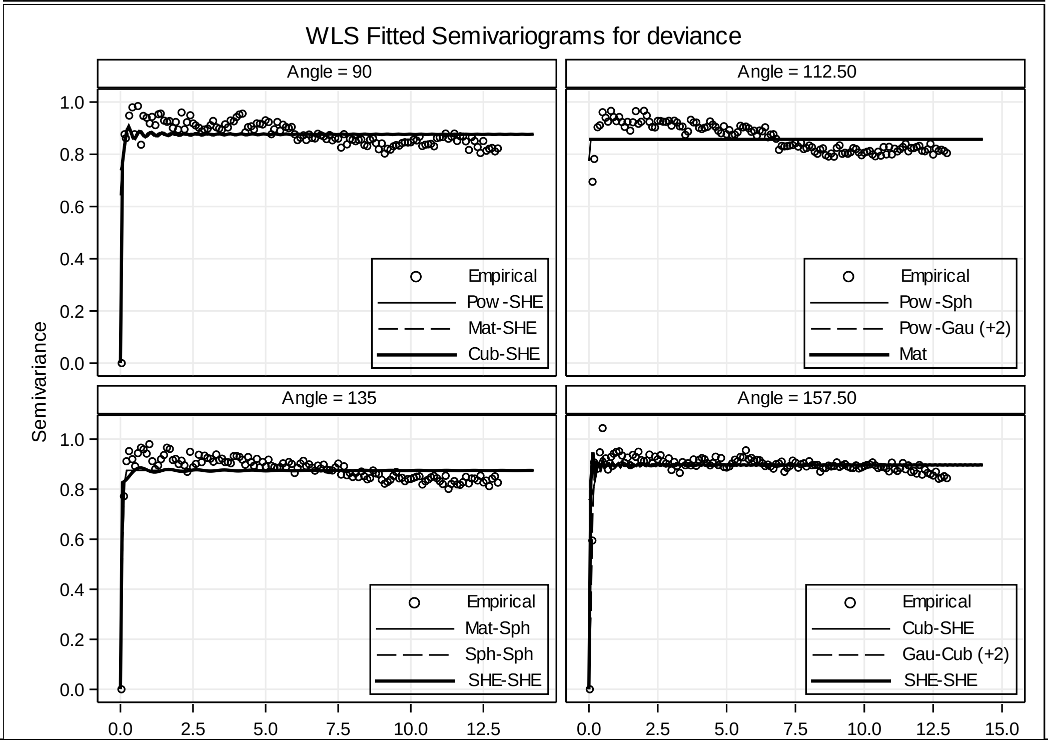

w.2) Pedestrian Crossing Sign, 3rd order spatial detrend + rater adjustment

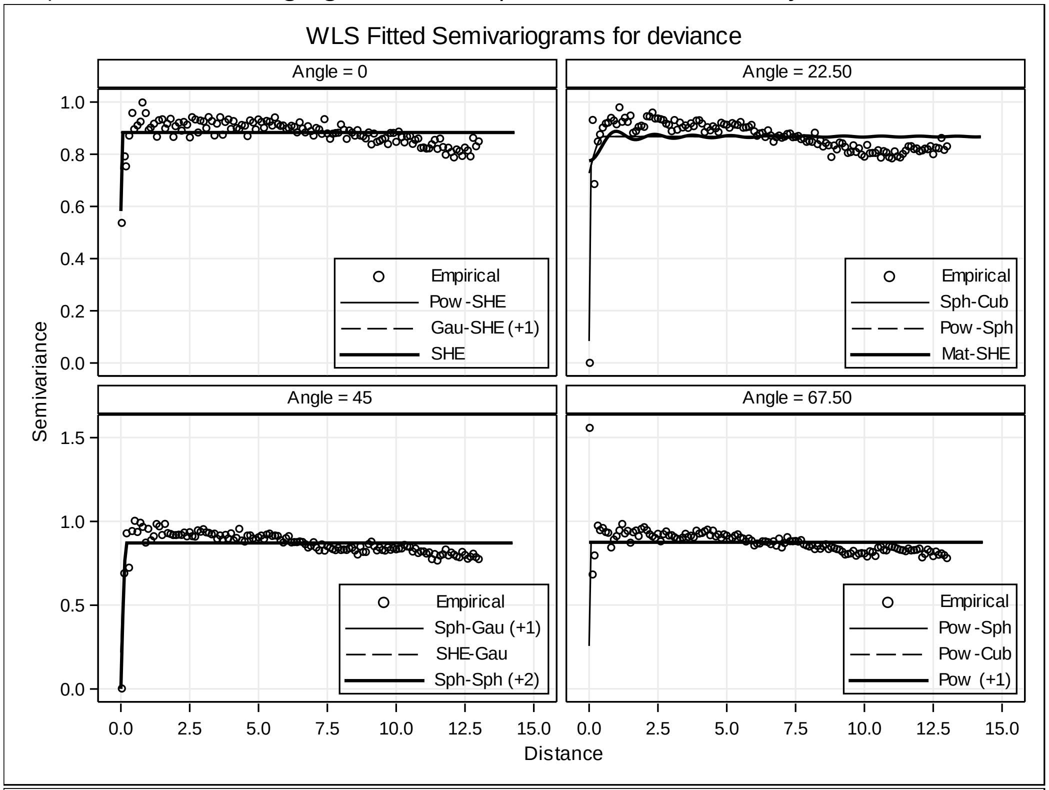

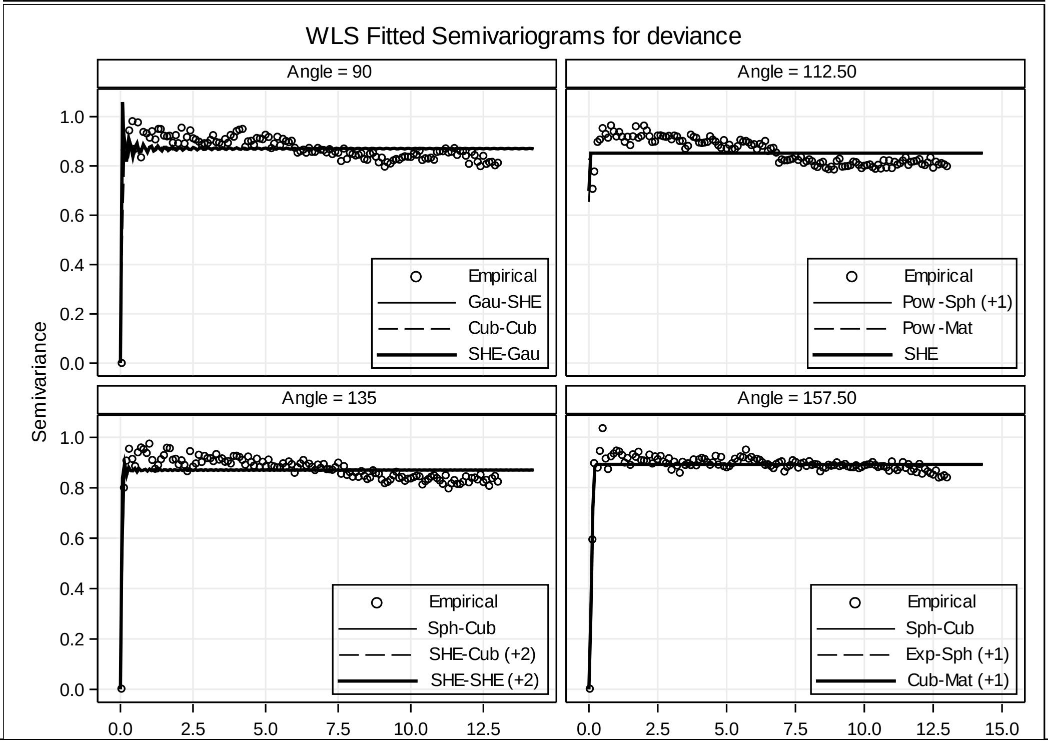

x.1) Pedestrian Signal, 3rd order spatial detrend

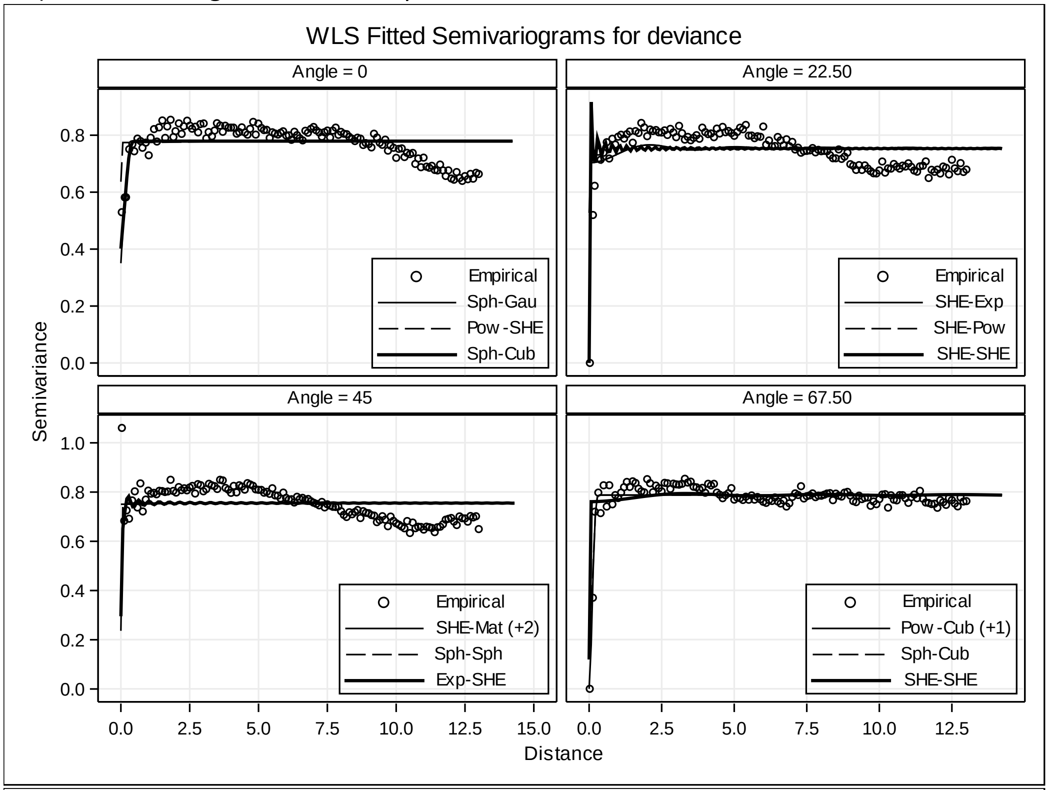

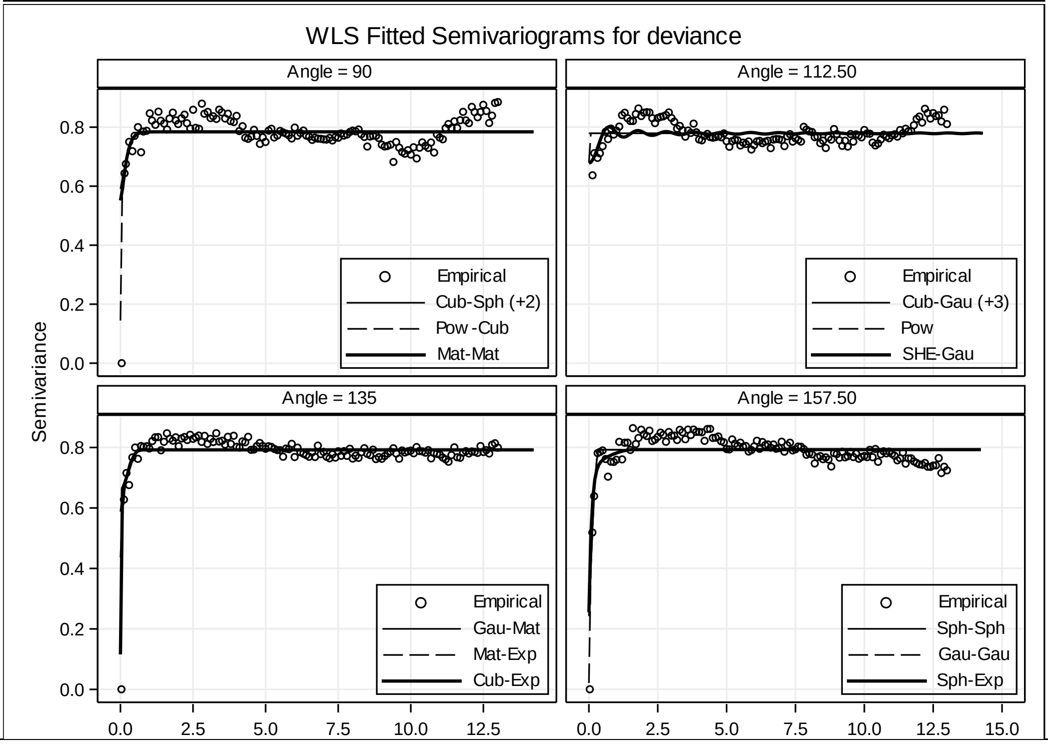

x.2) Pedestrian Signal, 3rd order spatial detrend + rater adjustment

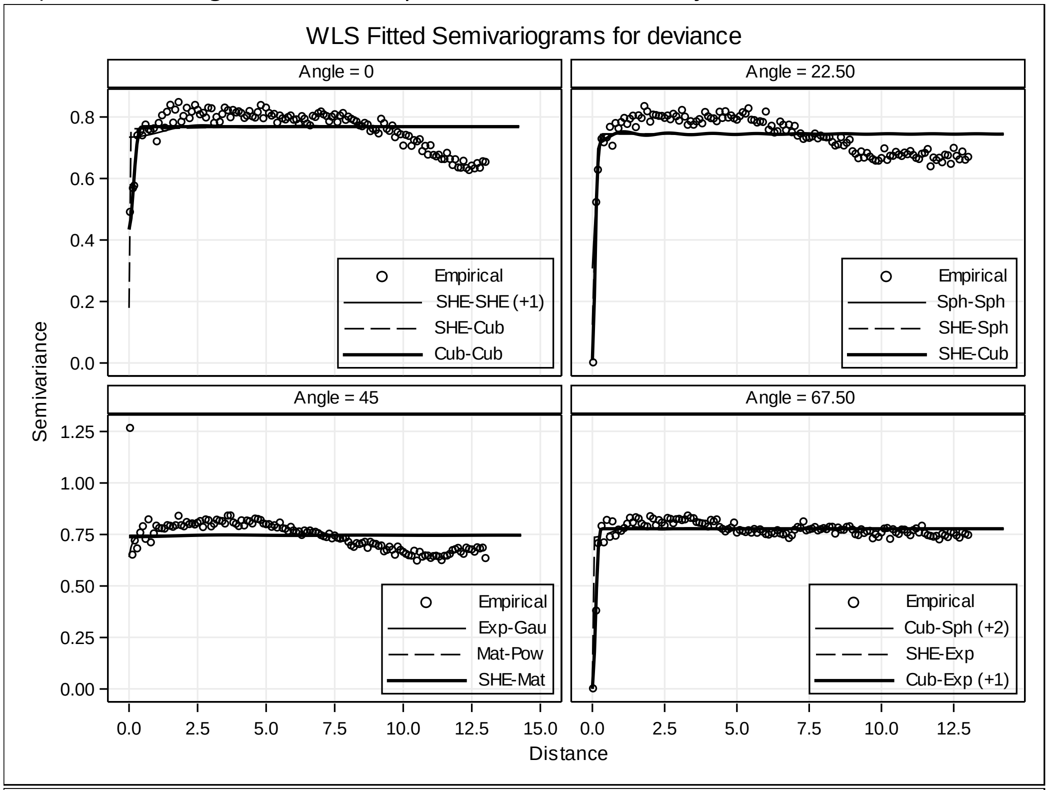

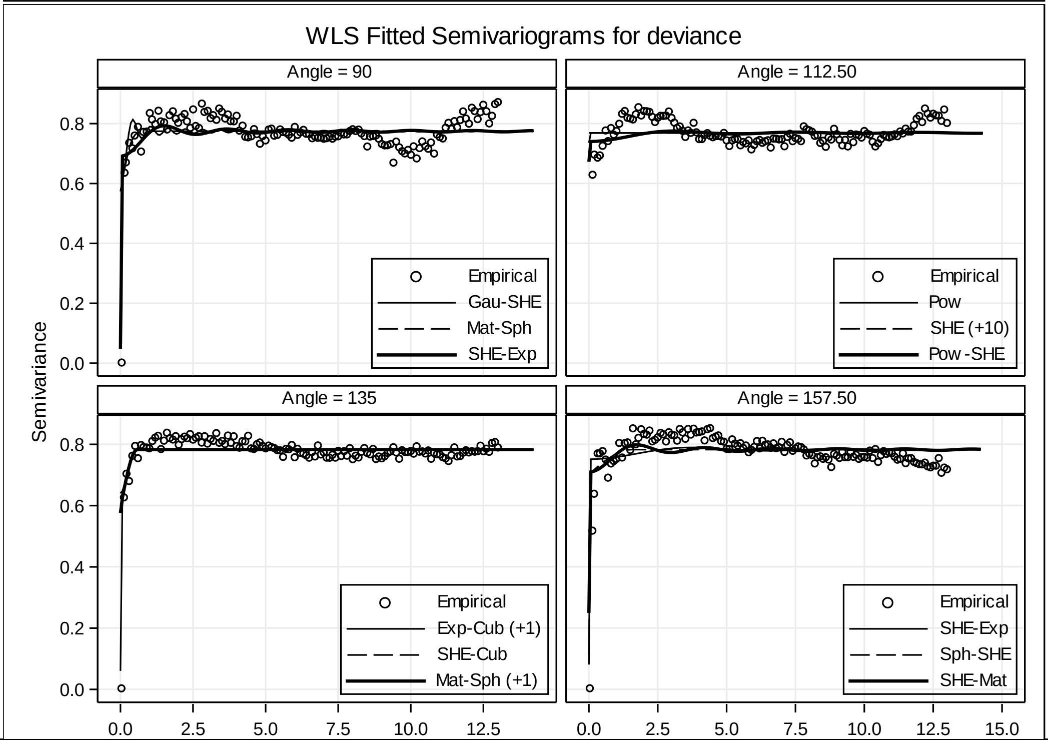

y.1) Pedestrian Crossing Marks, 3rd order spatial detrend

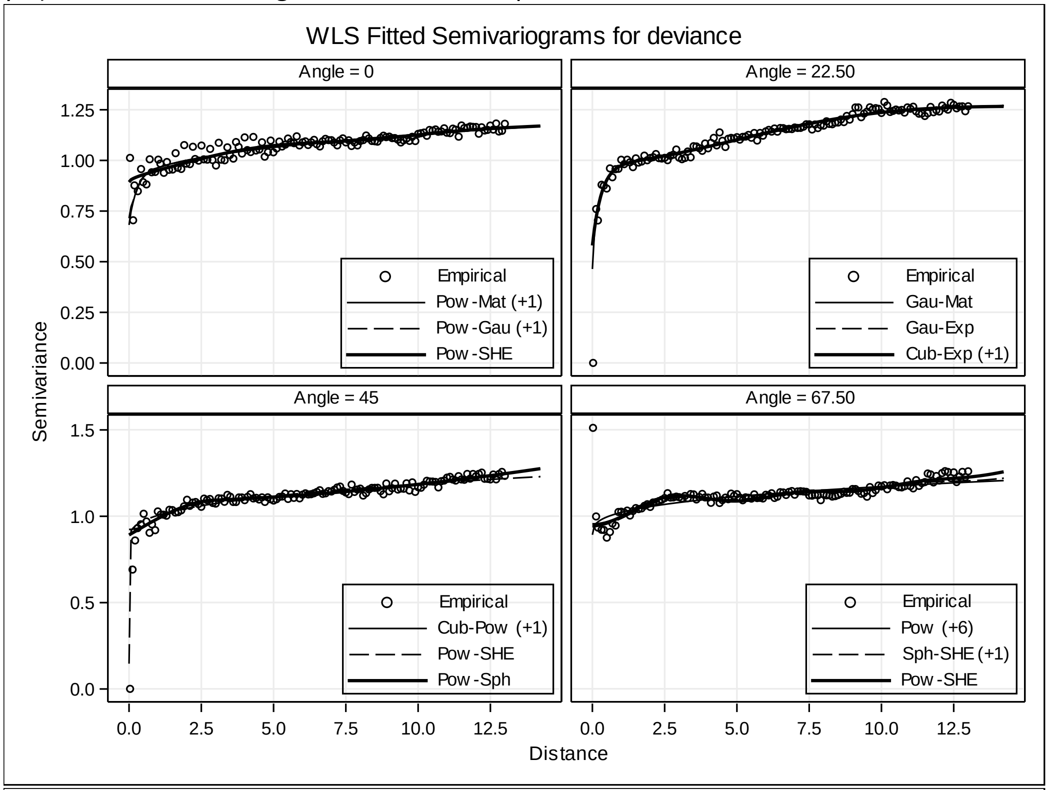

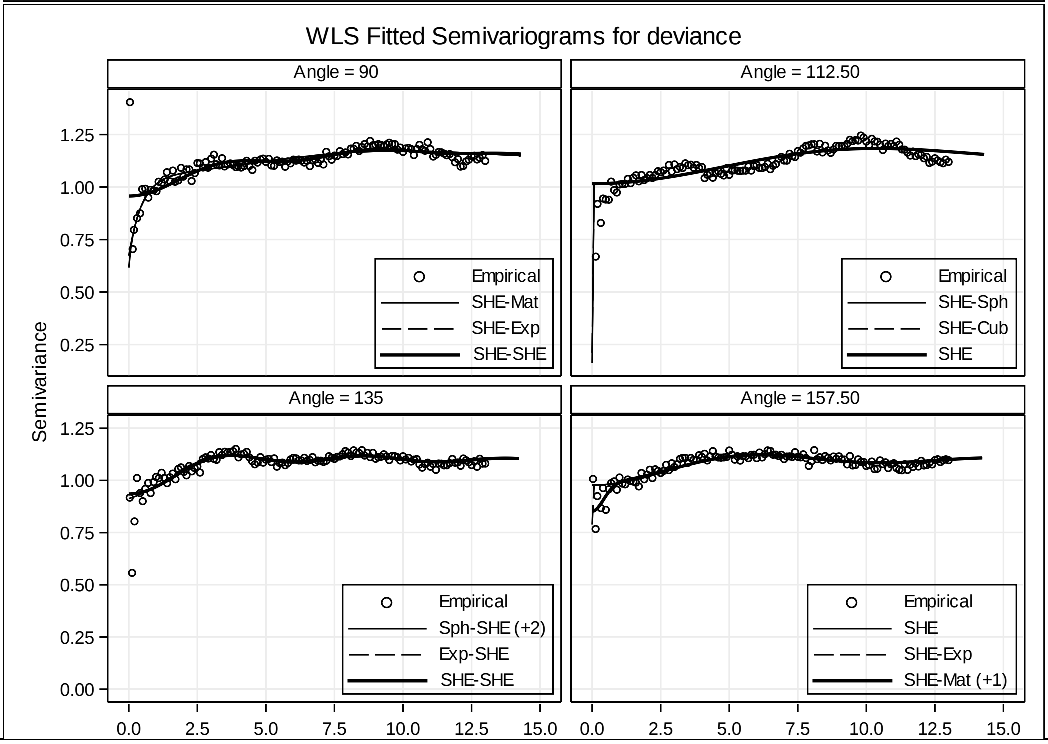

y.2) Pedestrian Crossing Marks, 3rd order spatial detrend + rater adjustment

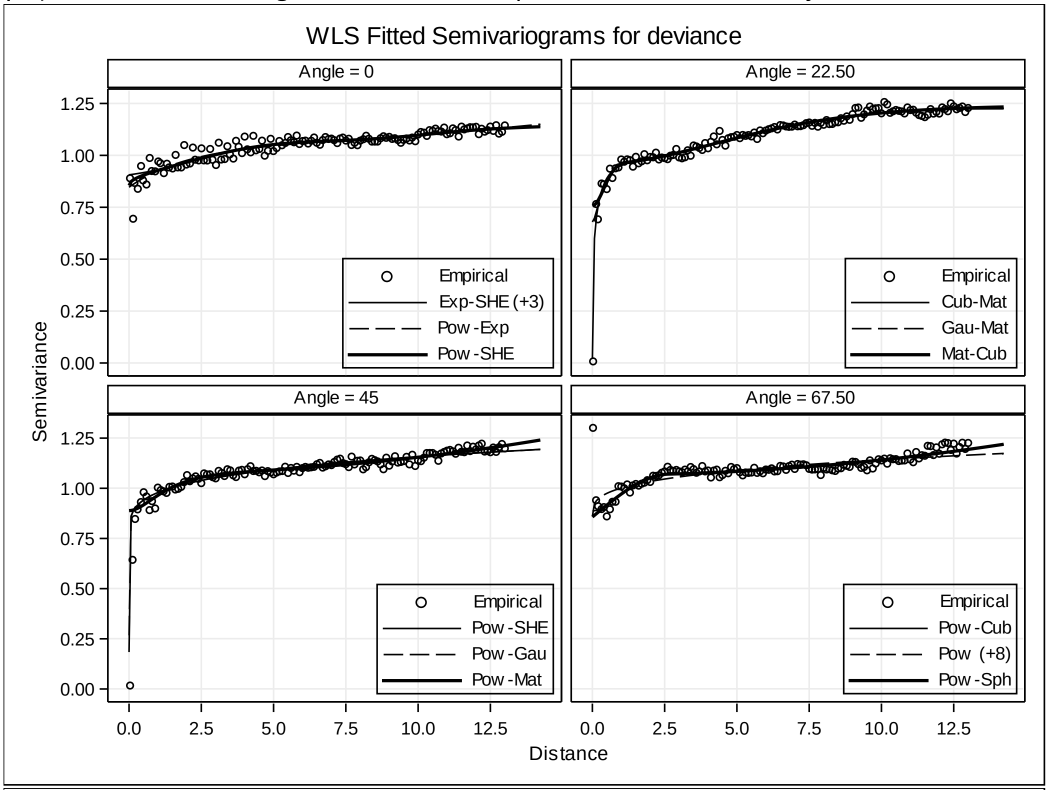

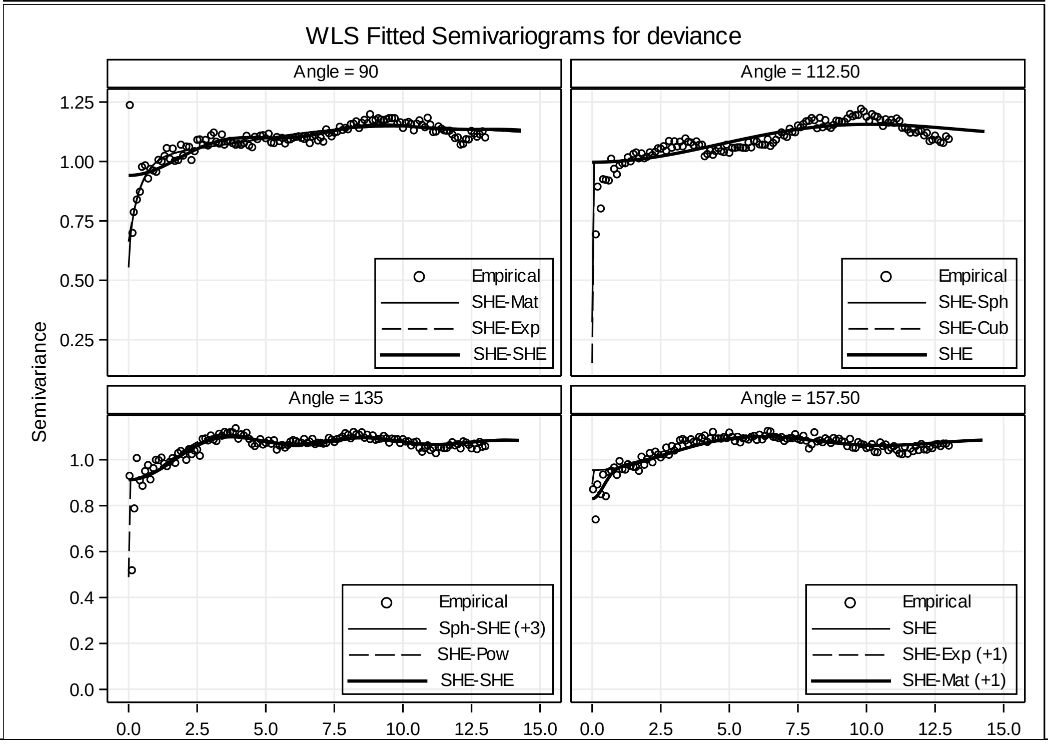

z.1) Type of Pedestrian Crosswalk, 3rd order spatial detrend

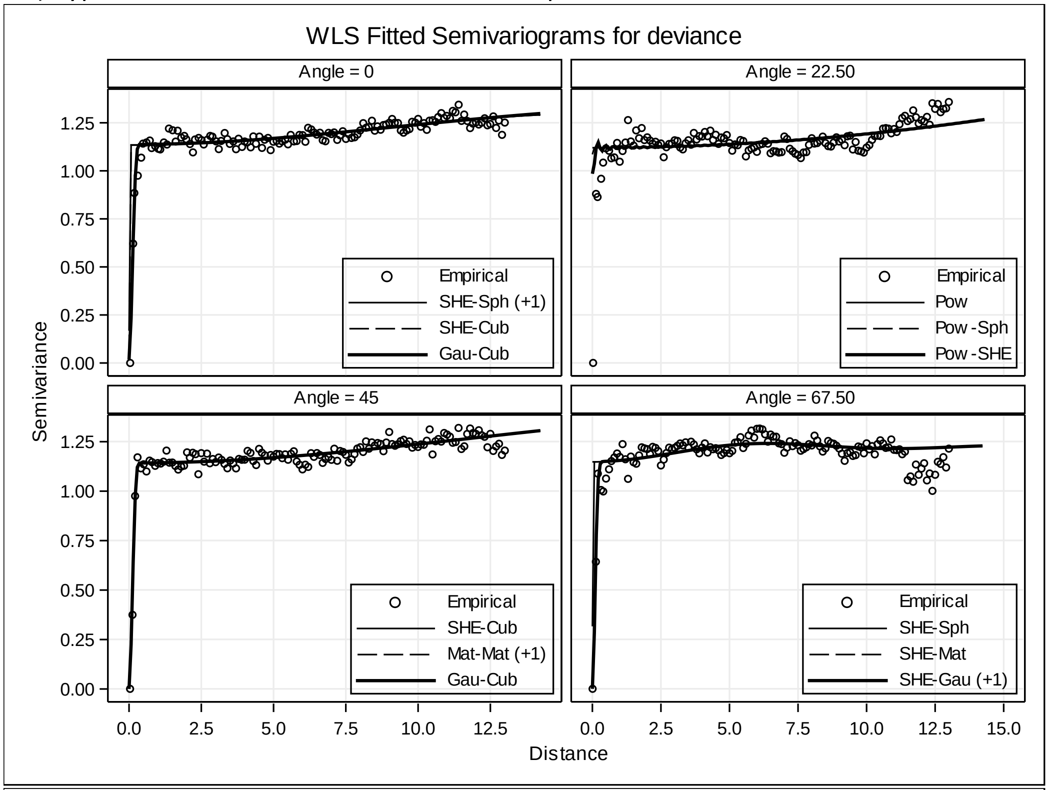

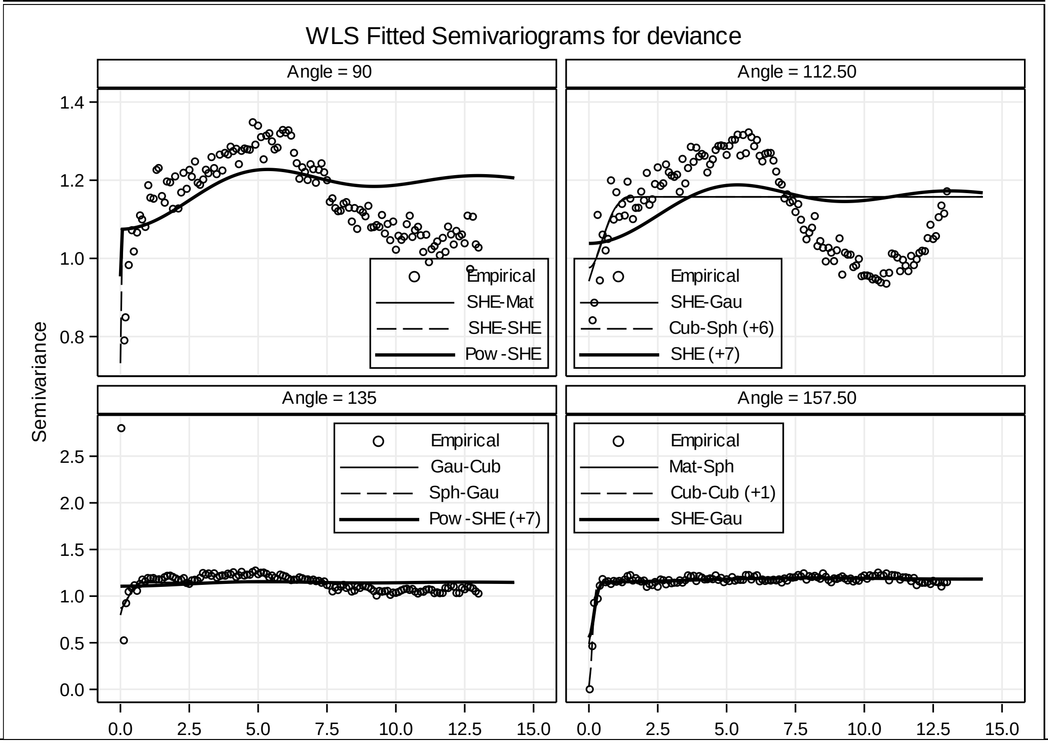

z.2) Type of Pedestrian Crosswalk, 3rd order spatial detrend + rater adjustment

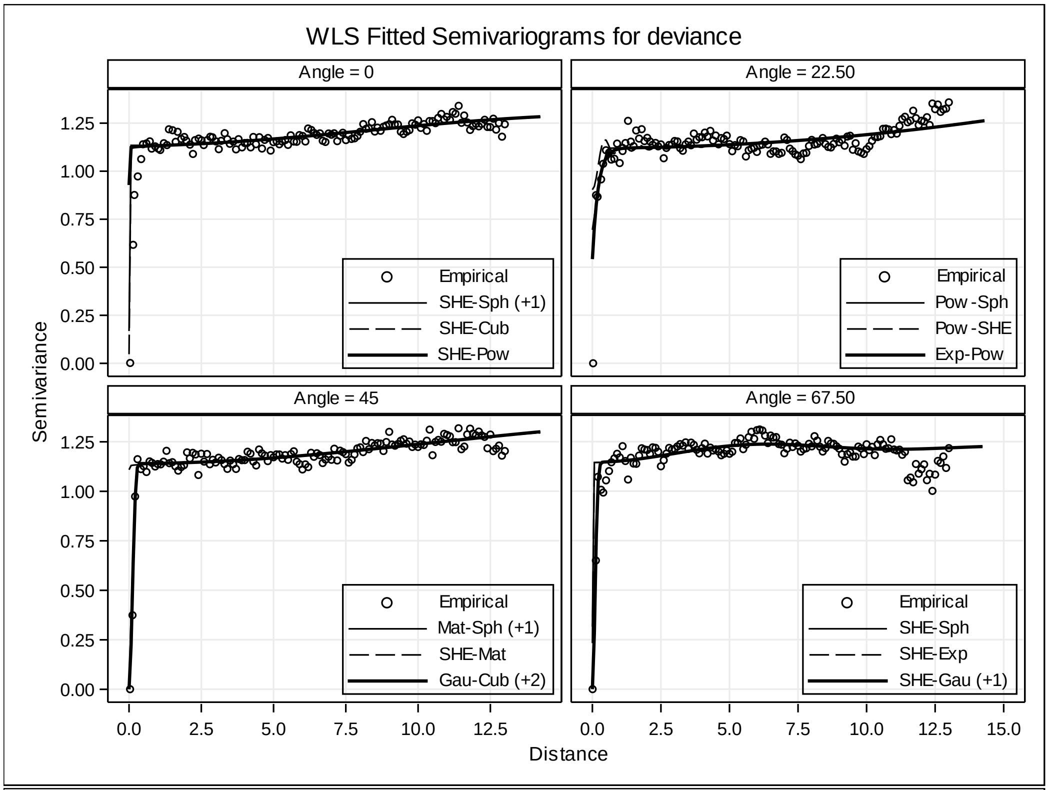

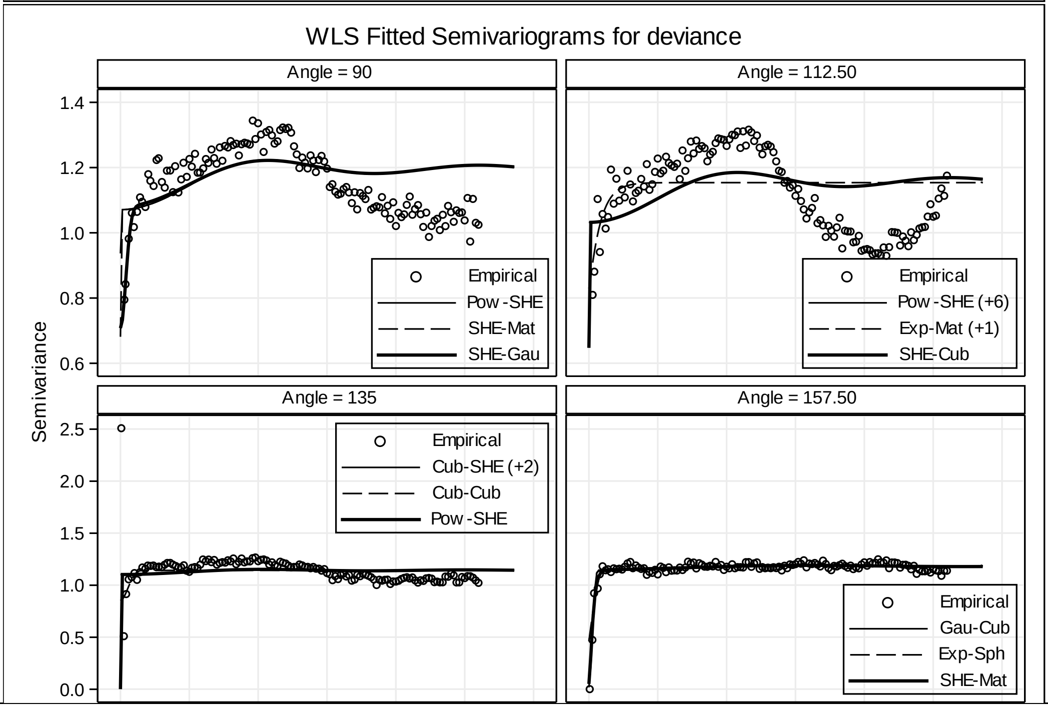

aa.1) Traffic Signal Type, 3rd order spatial detrend

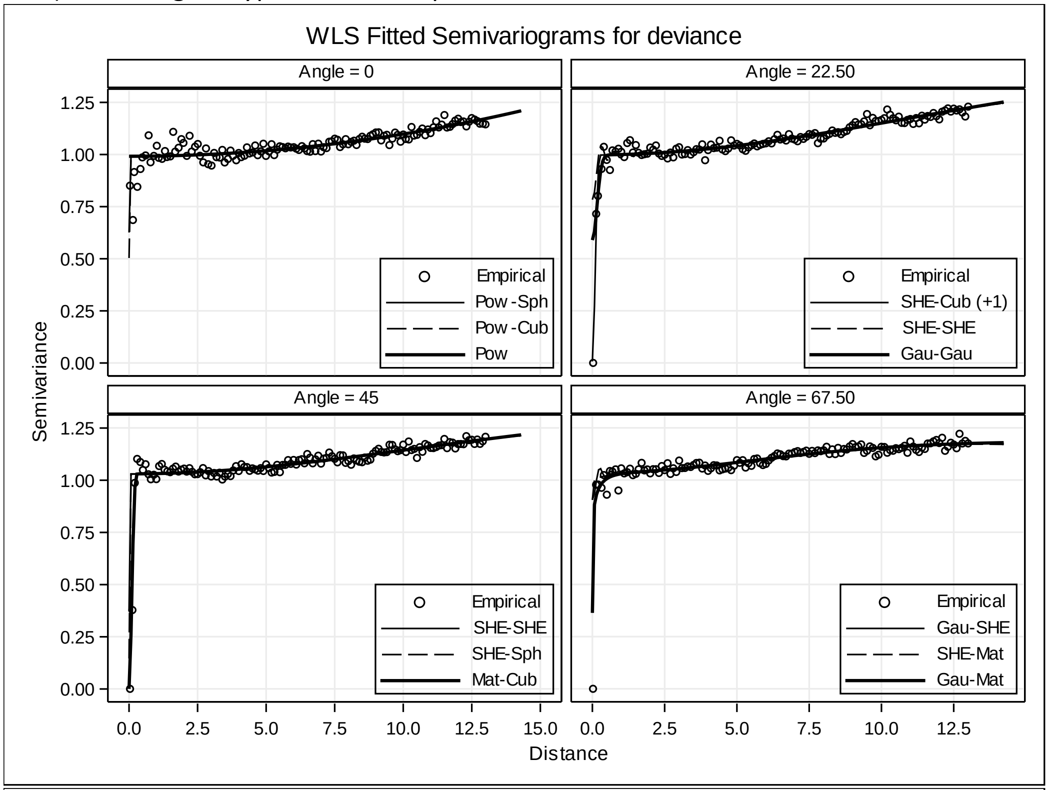

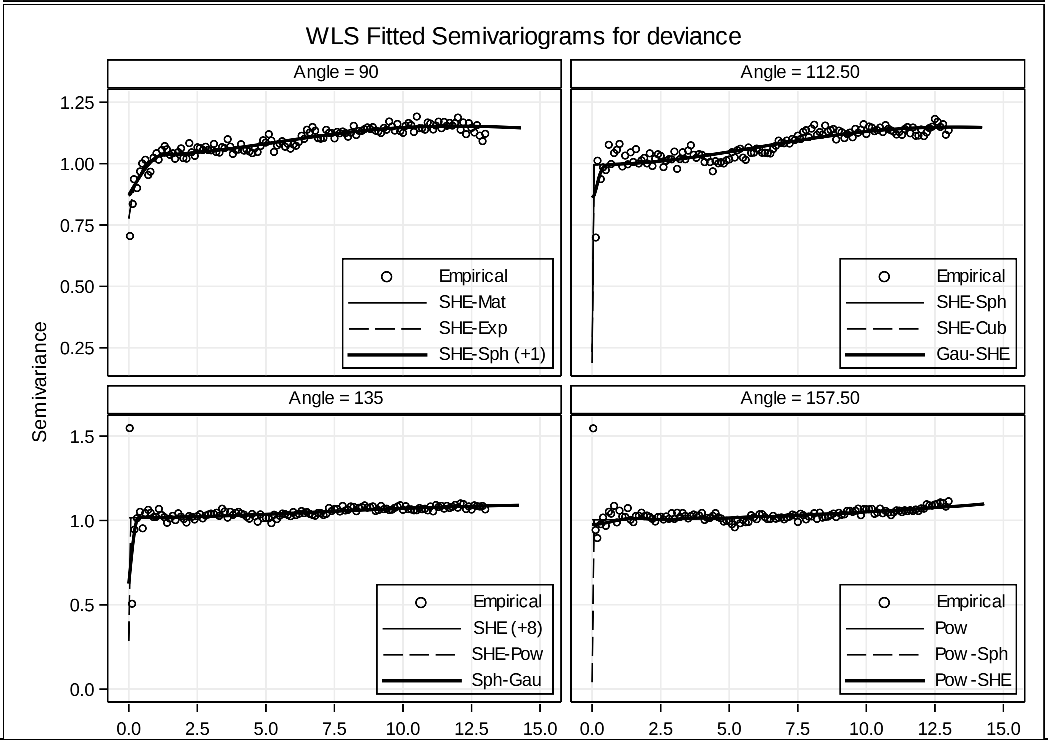

aa.2) Traffic Signal Type, 3rd order spatial detrend + rater adjustment

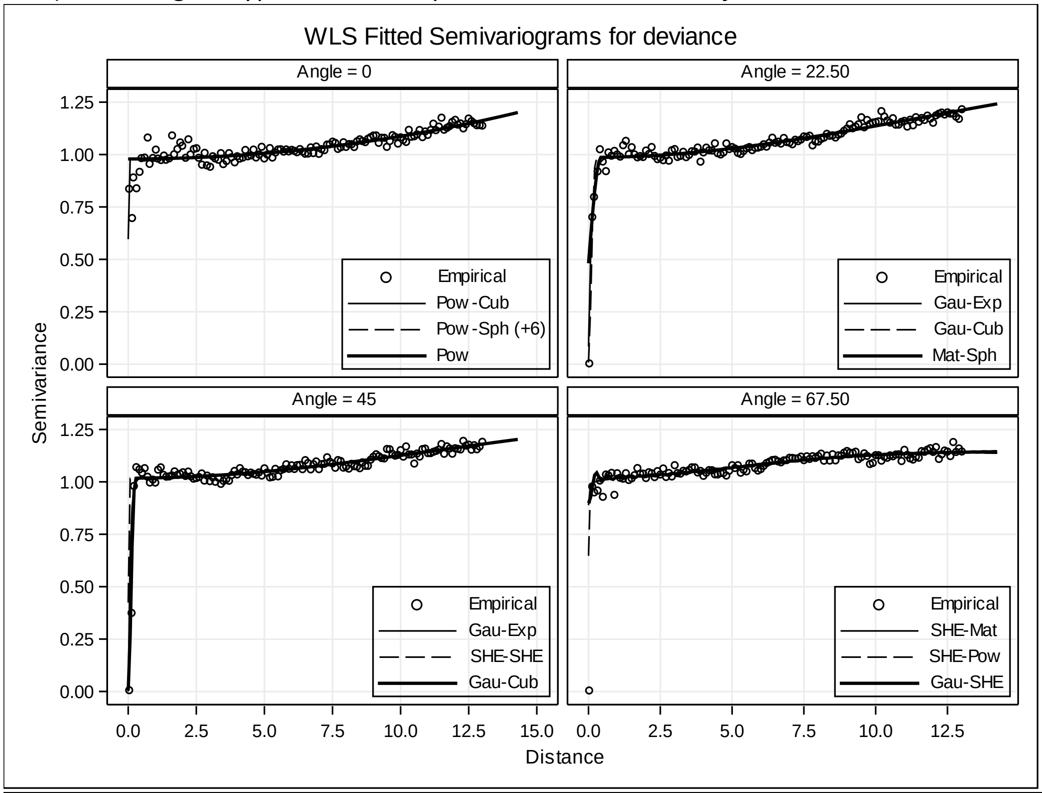

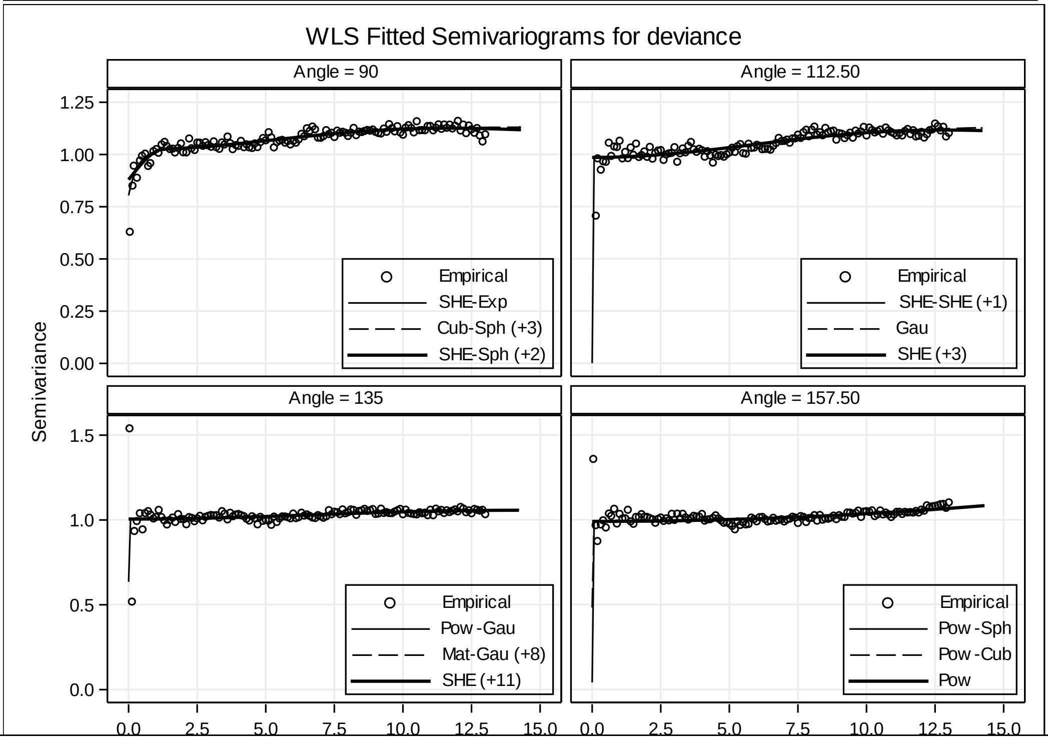

ab.1) One-way Street, 3rd order spatial detrend

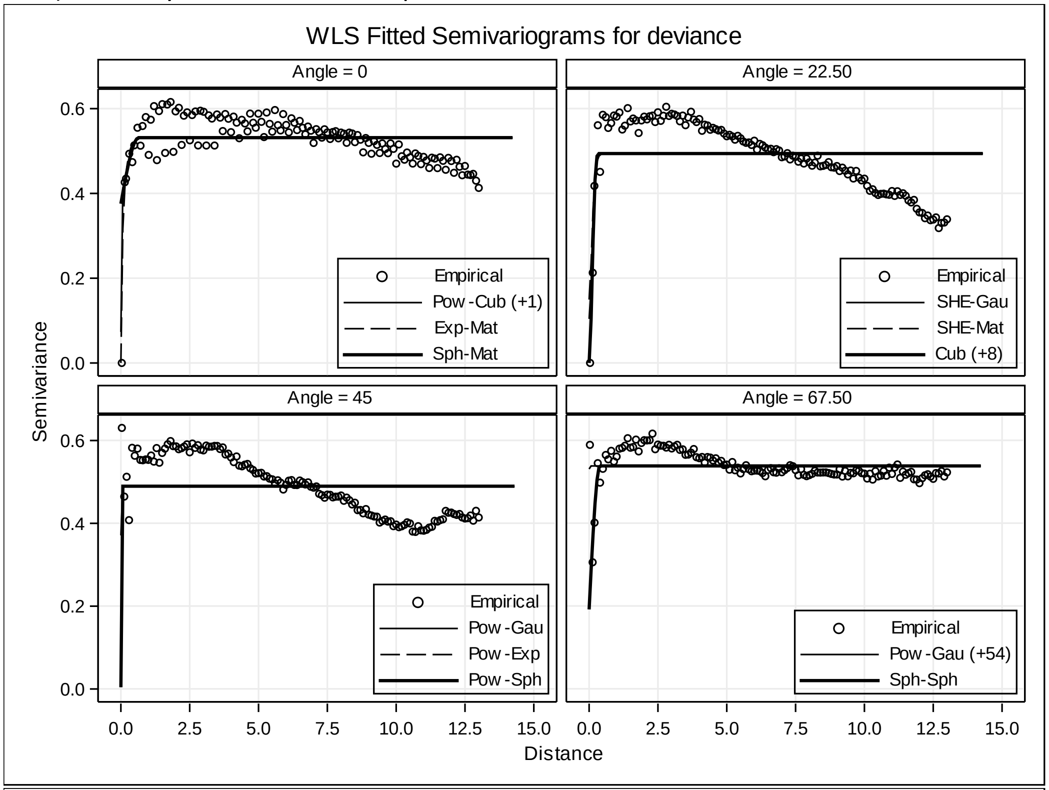

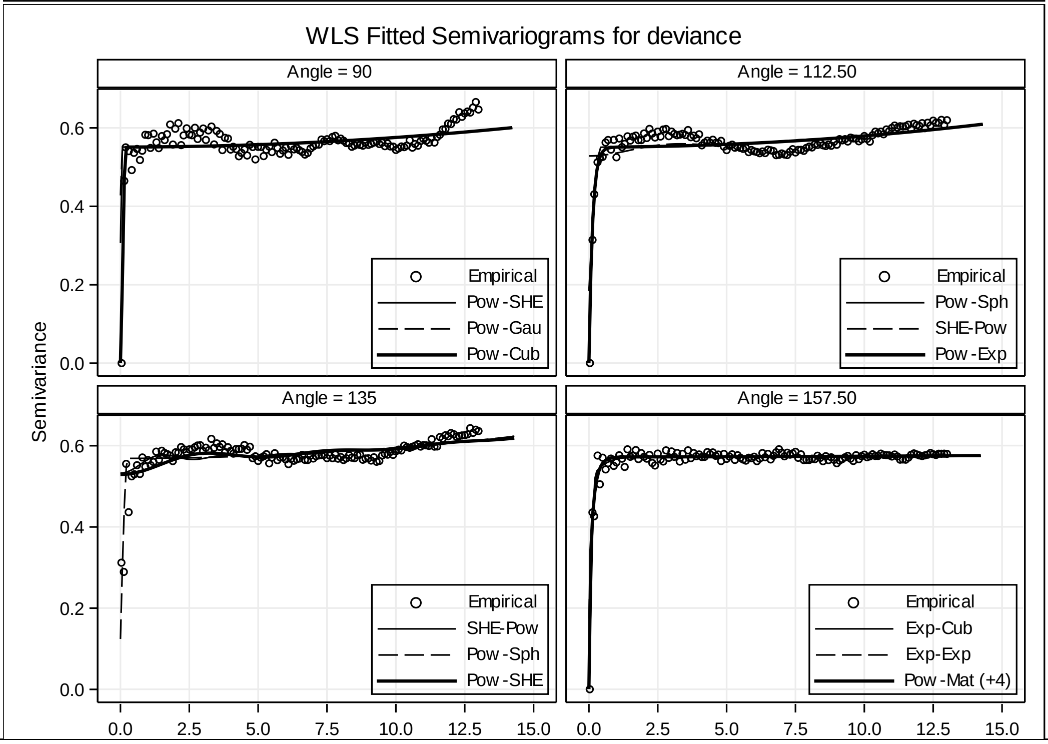

ab.2) One-way Street, 3rd order spatial detrend + rater adjustment

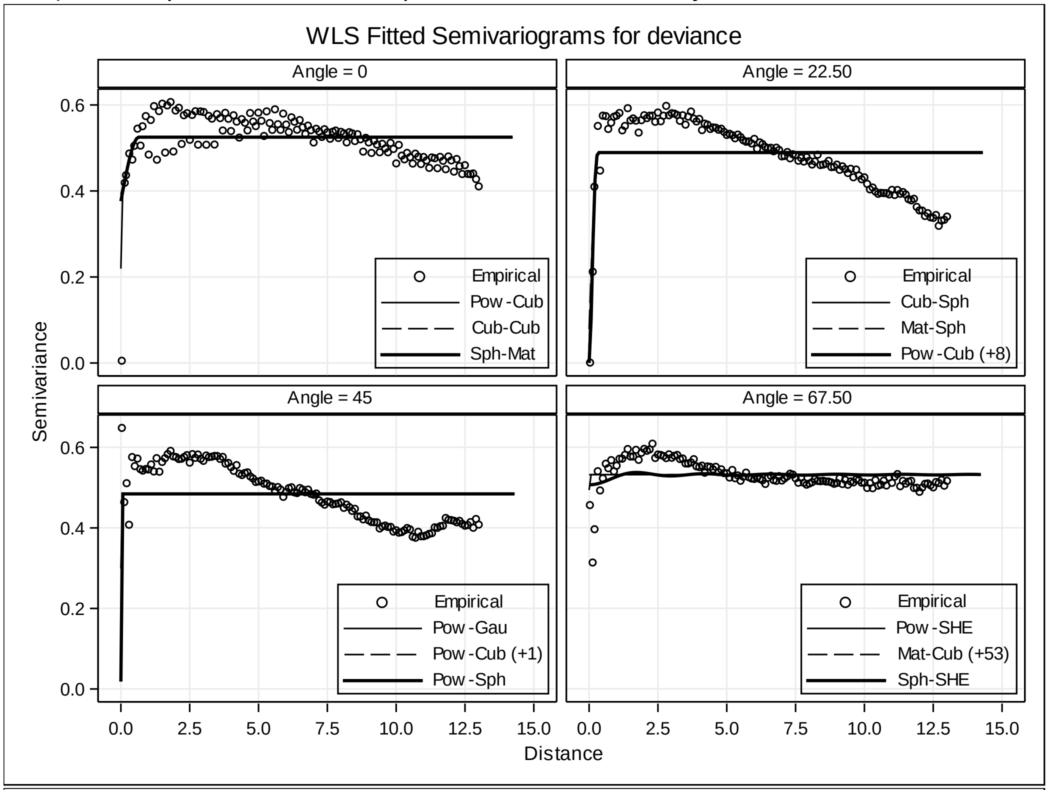

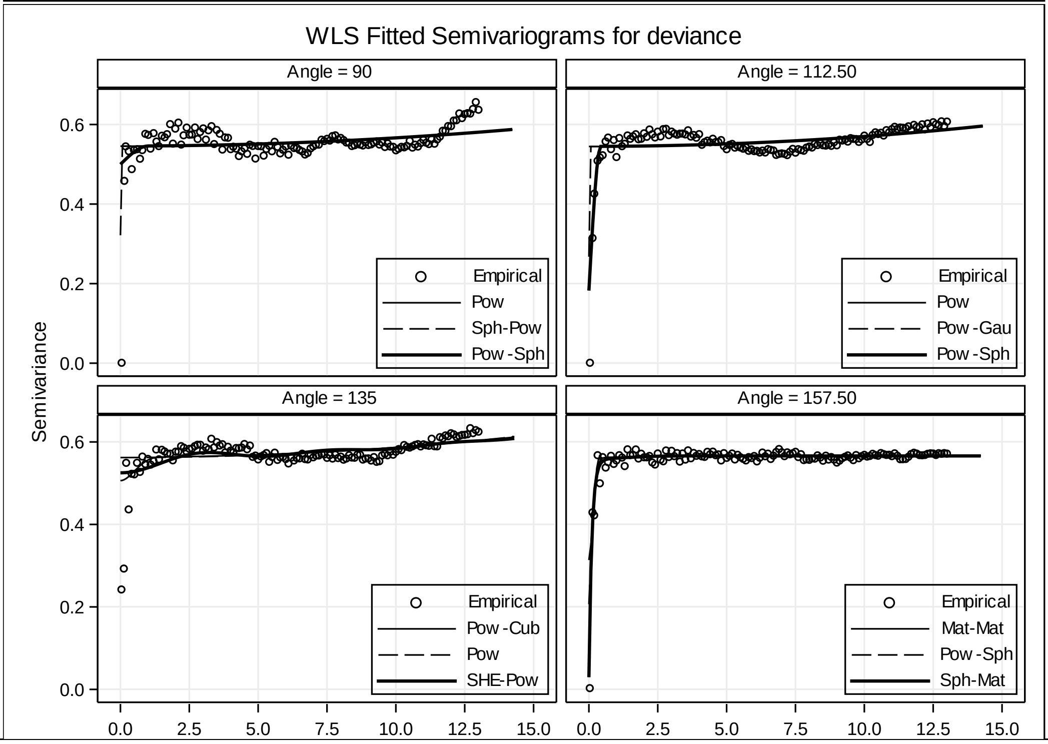

ac.1) Number of Lanes, 3rd order spatial detrend

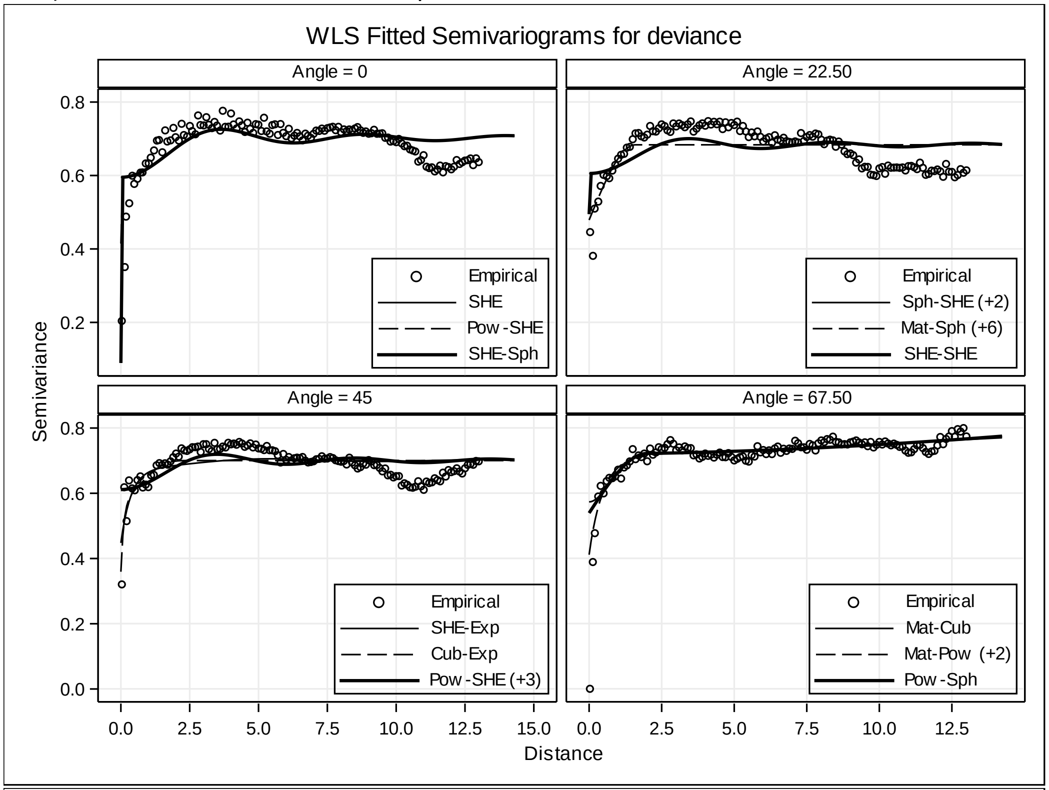

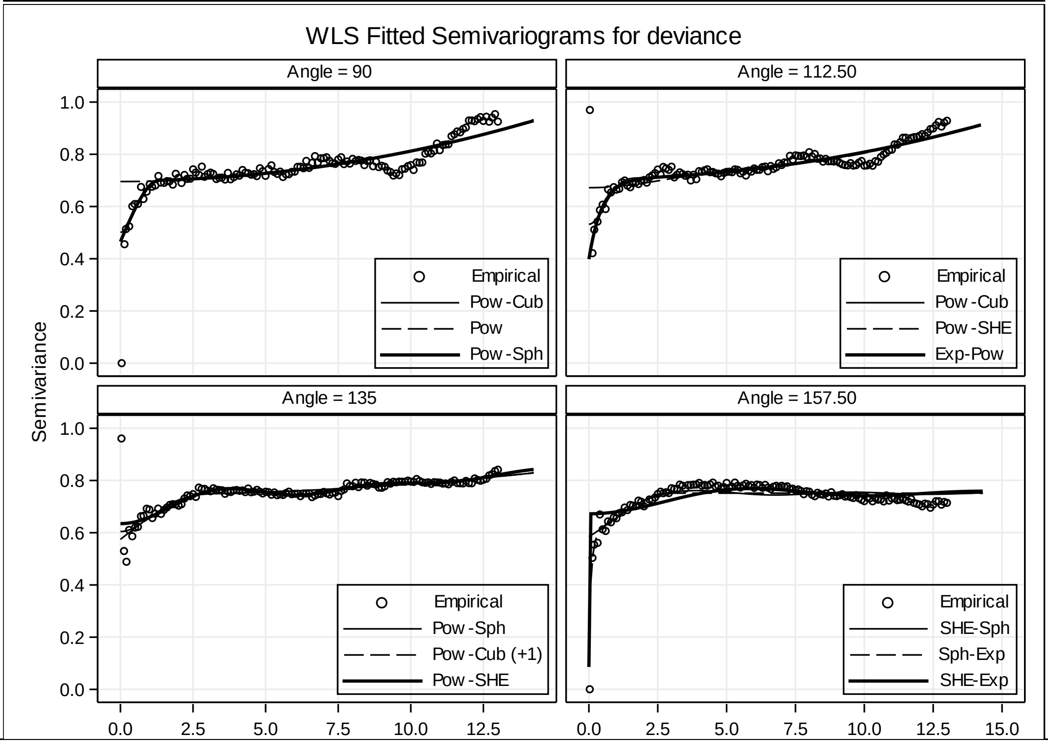

ac.2) Number of Lanes, 3rd order spatial detrend + rater adjustment

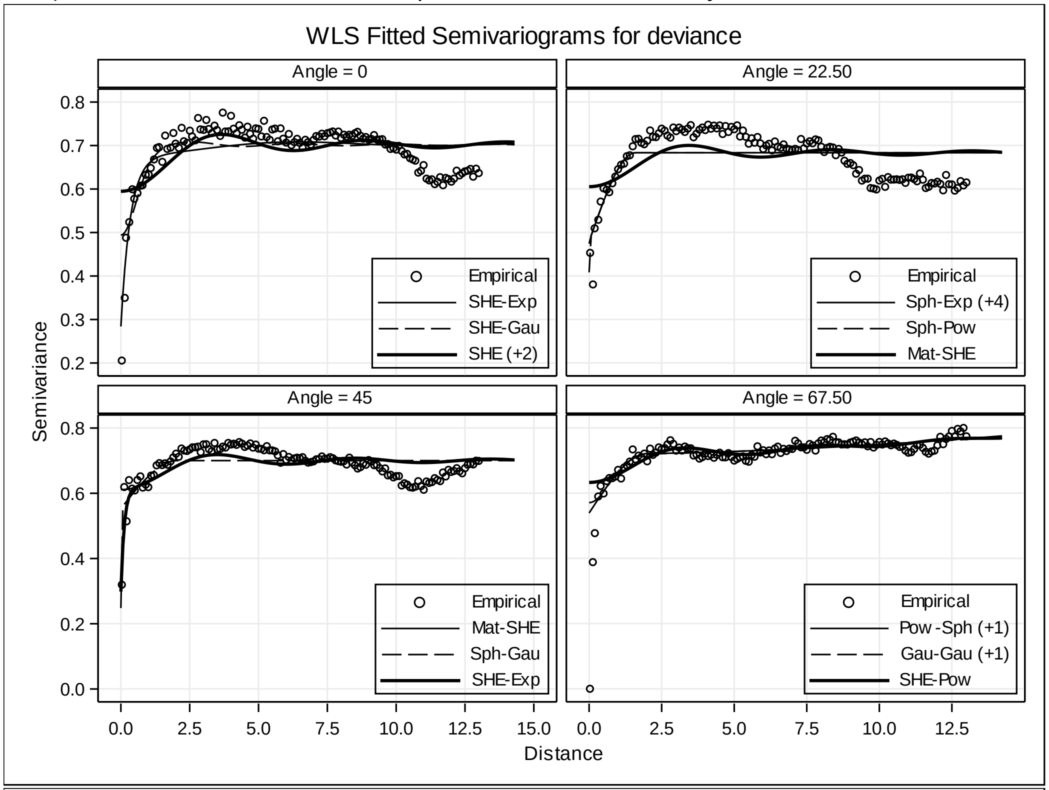

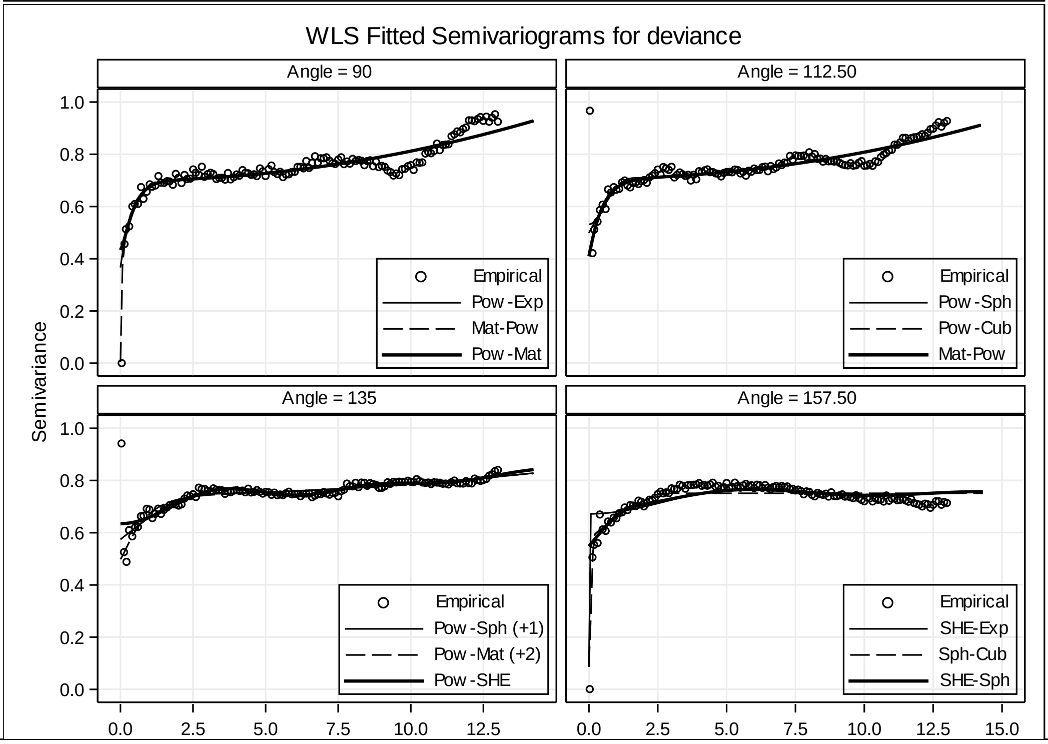

ad.1) Presence of Highway, 3rd order spatial detrend

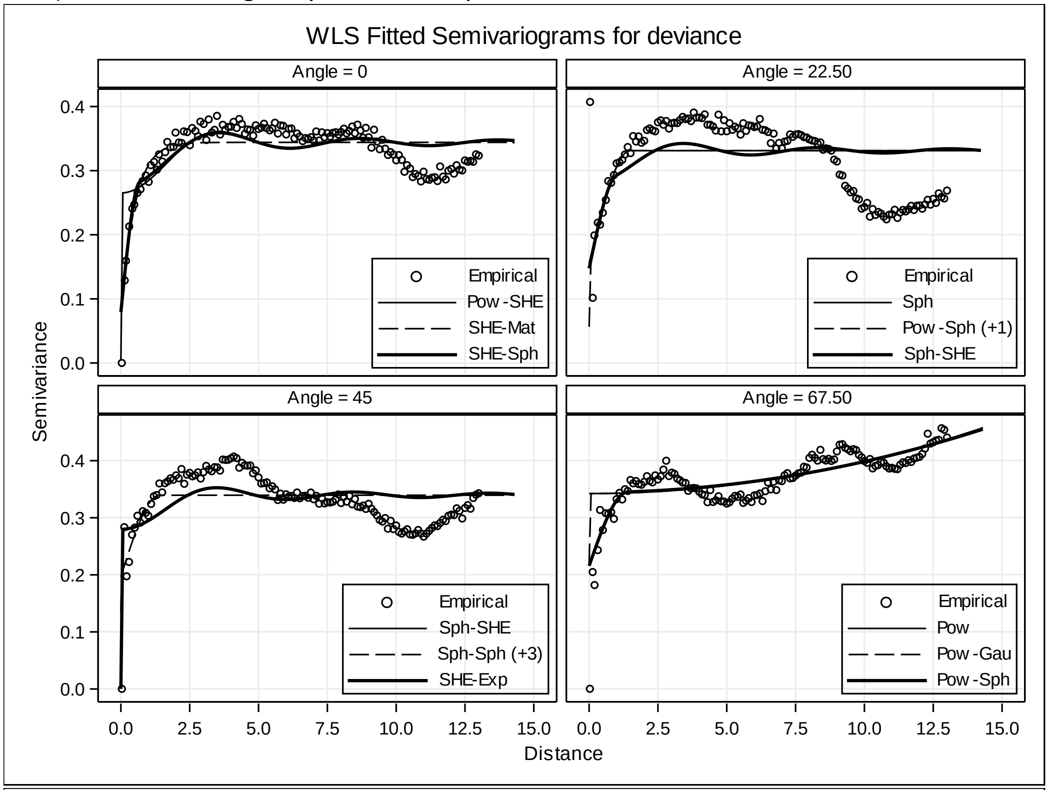

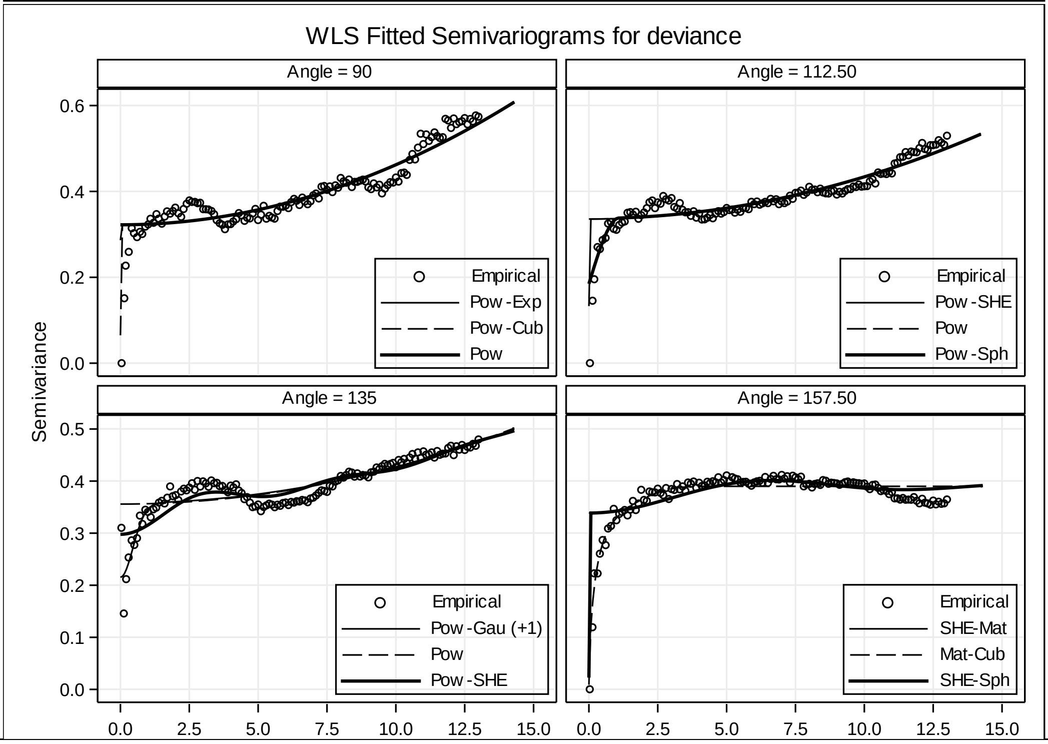

ad.2) Presence of Highway, 3rd order spatial detrend + rater adjustment

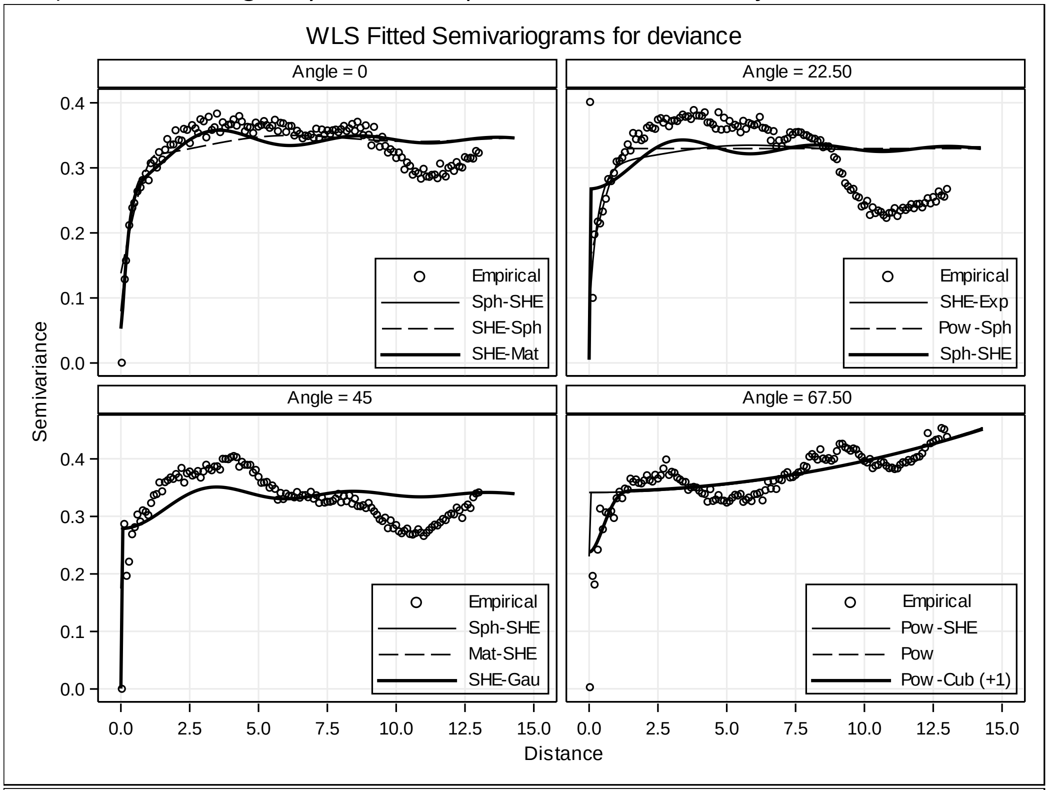

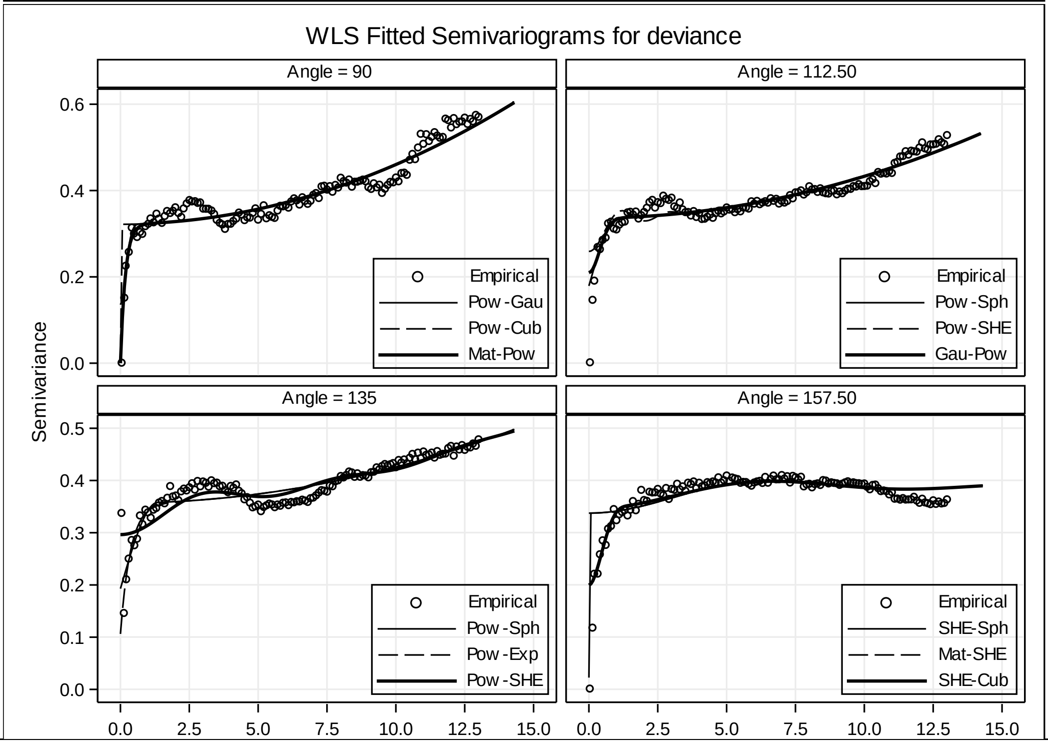

ae.1) Highway is Barrier, 3rd order spatial detrend

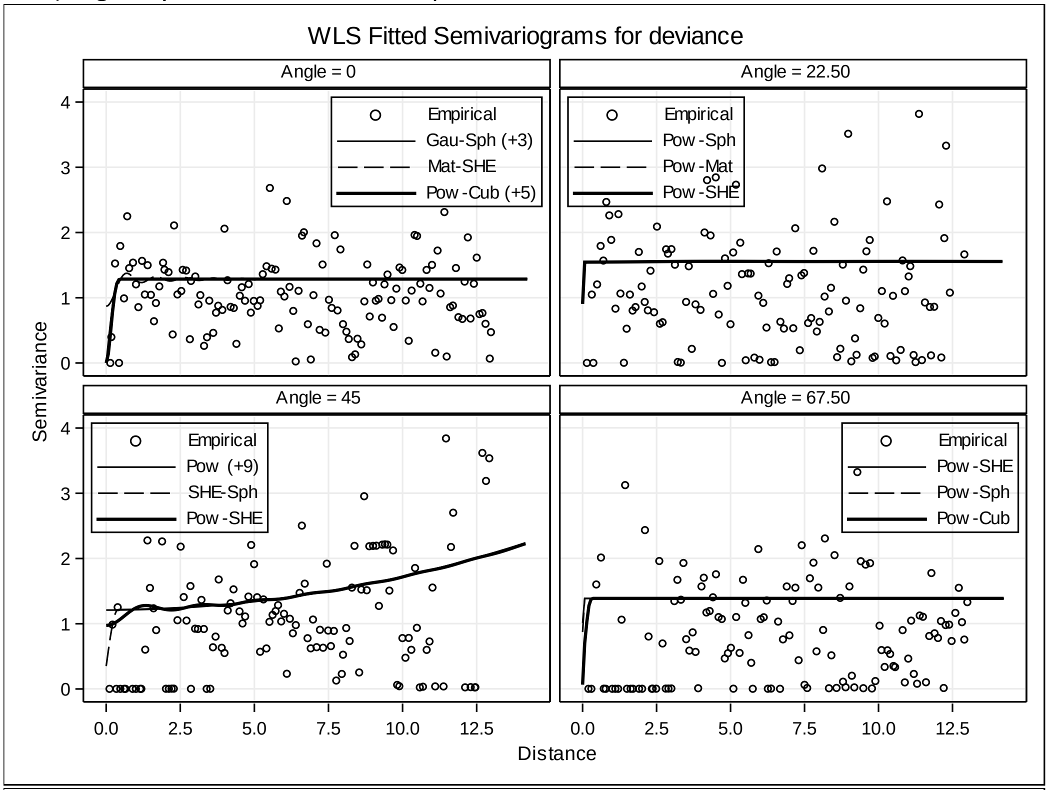

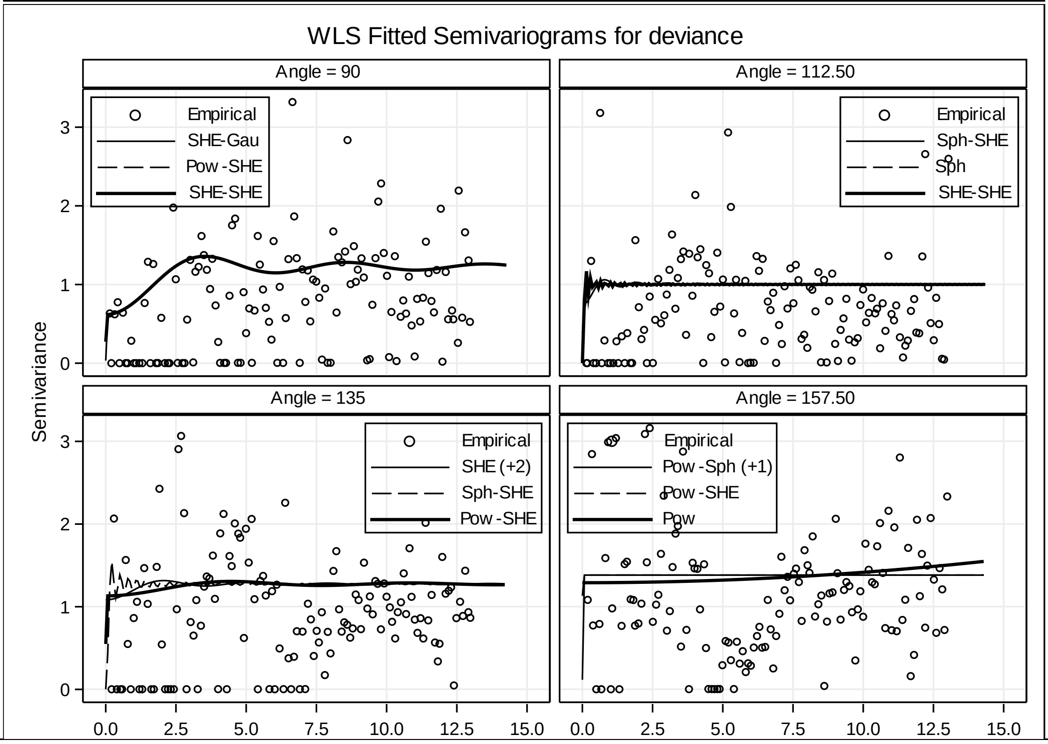

ae.2) Highway is Barrier, 3rd order spatial detrend + rater adjustment

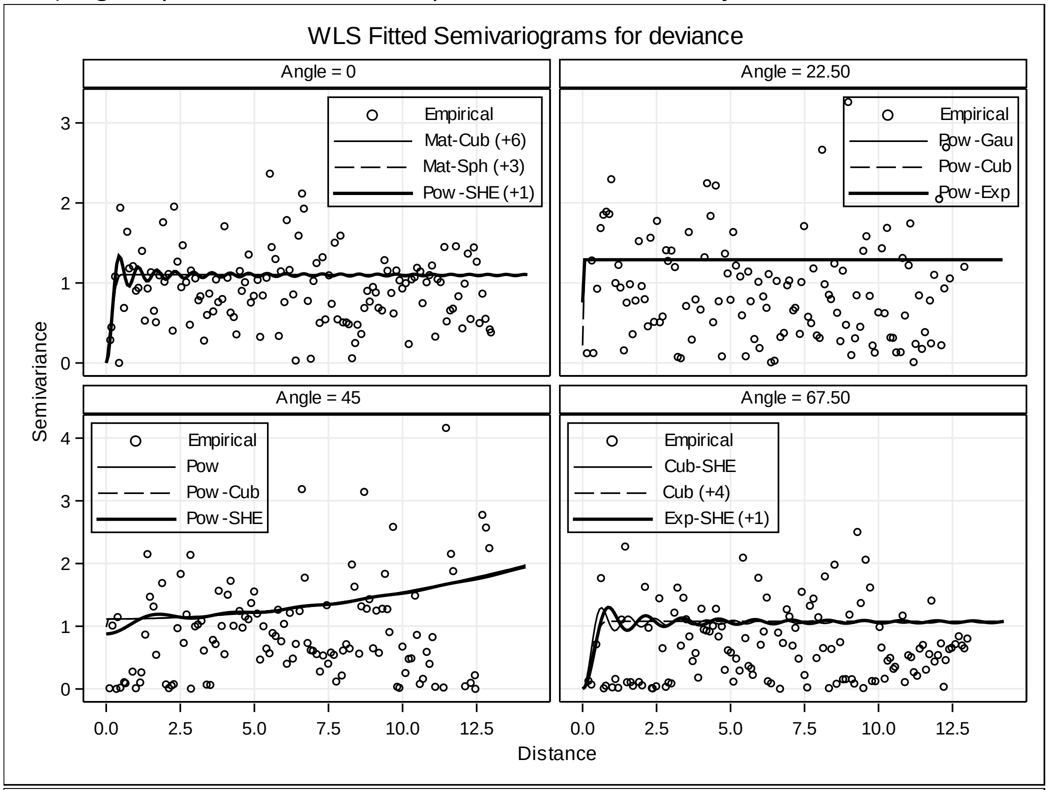

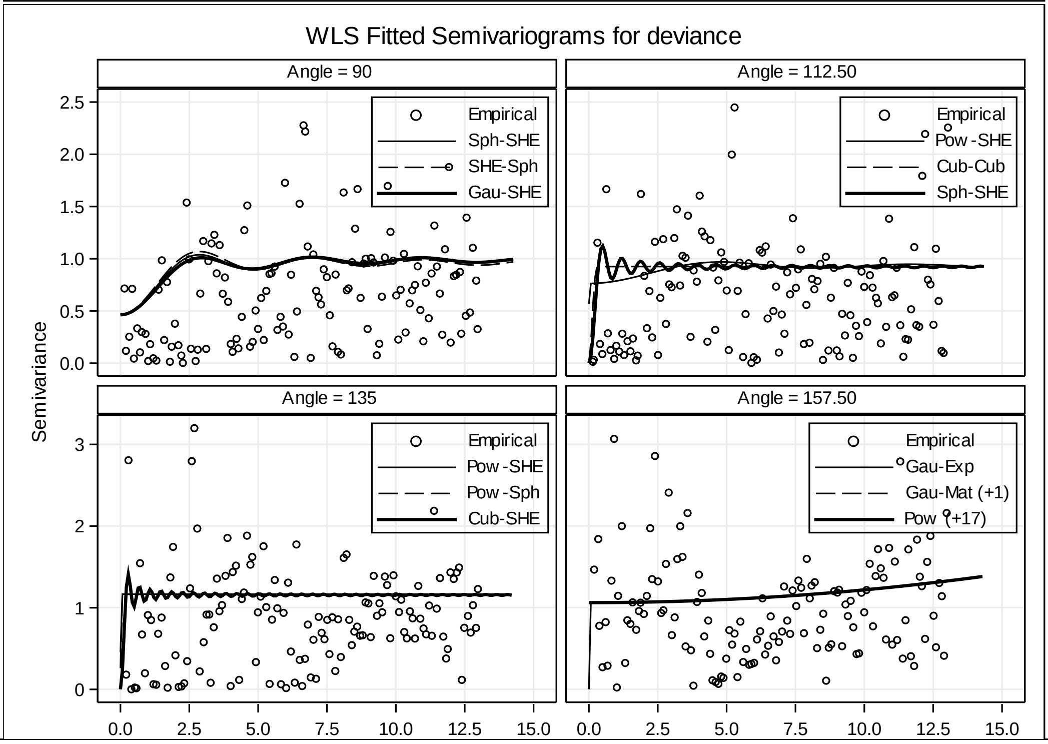

1. Cressie NAC: **Statistics for spatial data**, vol. Rev. New York: J. Wiley; 1993.
2. Waller LA, Gotway CA: **Applied spatial statistics for public health data**. Hoboken, N.J.: John Wiley & Sons; 2004.
3. Burrough PA: **Multiscale Sources of Spatial Variation in Soil .1. The Application of Fractal Concepts to Nested Levels of Soil Variation**. *J Soil Sci* 1983, **34**(3):577-597.
